# The role of ribosomal protein networks in ribosome dynamics

**DOI:** 10.1093/nar/gkae1308

**Published:** 2025-01-10

**Authors:** Youri Timsit, Grégoire Sergeant-Perthuis, Daniel Bennequin

**Affiliations:** Aix Marseille Univ, Université de Toulon, CNRS, IRD, MIO UM110, 163 avenue de Luminy 13288 Marseille, France; Research Federation for the Study of Global Ocean Systems Ecology and Evolution, FR2022/Tara GOSEE, 3 Rue Michel-Ange, 75016 Paris, France; Laboratory of Computational and Quantitative Biology (LCQB), Sorbonne Université, 4 Place Jussieu, 75005 Paris, France; Institut de Mathématiques de Jussieu - Paris Rive Gauche (IMJ-PRG), UMR 7586, CNRS, Université Paris Diderot, 8, Pace Aurélie Nemours, 75013 Paris, France

## Abstract

Accurate protein synthesis requires ribosomes to integrate signals from distant functional sites and execute complex dynamics. Despite advances in understanding ribosome structure and function, two key questions remain: how information is transmitted between these distant sites, and how ribosomal movements are synchronized? We recently highlighted the existence of ribosomal protein networks, likely evolved to participate in ribosome signaling. Here, we investigate the relationship between ribosomal protein networks and ribosome dynamics. Our findings show that major motion centers in the bacterial ribosome interact specifically with r-proteins, and that ribosomal RNA exhibits high mobility around each r-protein. This suggests that periodic electrostatic changes in the context of negatively charged residues (Glu and Asp) induce RNA–protein ‘distance-approach’ cycles, controlling key ribosomal movements during translocation. These charged residues play a critical role in modulating electrostatic repulsion between RNA and proteins, thus coordinating ribosomal dynamics. We propose that r-protein networks synchronize ribosomal dynamics through an ‘electrostatic domino’ effect, extending the concept of allostery to the regulation of movements within supramolecular assemblies.

## Graphical Abstract

**Graphical Abstract:**
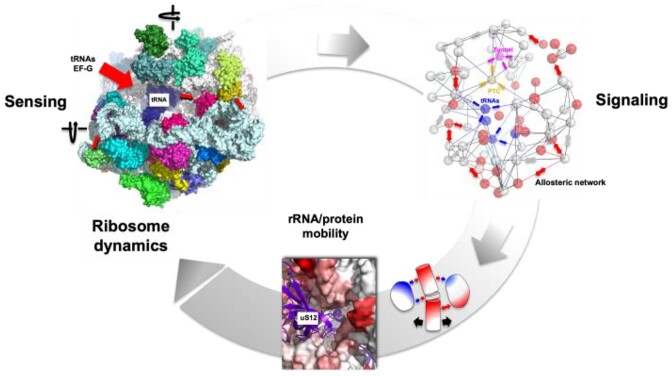

## Introduction

The ribosomes, ribonucleoprotein particles composed of a large (LSUs) and a small subunits (SSUs) perform translation – messenger RNA (mRNA)-coded protein synthesis – in the three domains of life (1–4). They represent a fascinating system for several reasons, including the coordinated assembly of their components (5–7), their ability to integrate multiple signals to modulate translation (8–10) and the valuable insights they provide into evolution and early life forms (11–14). One of their most intriguing features is the complex dynamics that occur during translation (15). Translation consists of four phases – initiation, elongation, termination and recycling where the precise coordination of ribosome movements (16,17) directs and controls the passage of mRNA, translation factors and transfer RNA (tRNA) (18–20), while enabling the release of the newly synthesized peptide through the exit tunnel (21–26). Numerous experimental (21,27,28) and theoretical studies (29,30) have revealed that all of these components undergo coordinated movements combining large-scale and fine-tuned conformational changes. This dynamic behavior is driven by the subtle modulation of intermolecular interactions, which have evolved to maintain a ribosome energy landscape conducive to dynamic motion and to control steric hindrances along the tRNA passage route (30–32).

Elongation begins with decoding, during which the ribosome dynamically monitors the correct codon–anticodon pairing between the mRNA and the tRNA-A complexed with EF-Tu, delivered to the A site (33,34). This occurs through a two-step process—initial selection and proofreading—that ensures the high fidelity of decoding (Supplementary Text 1A) (30,35,36). A peptide bond is then formed between the peptide chain of the P-site tRNA (P) and the amino acid of the A-site tRNA (37). This results in the formation of a peptidyl-tRNA, which occupies the ribosomal A-site.

**Figure 1.**
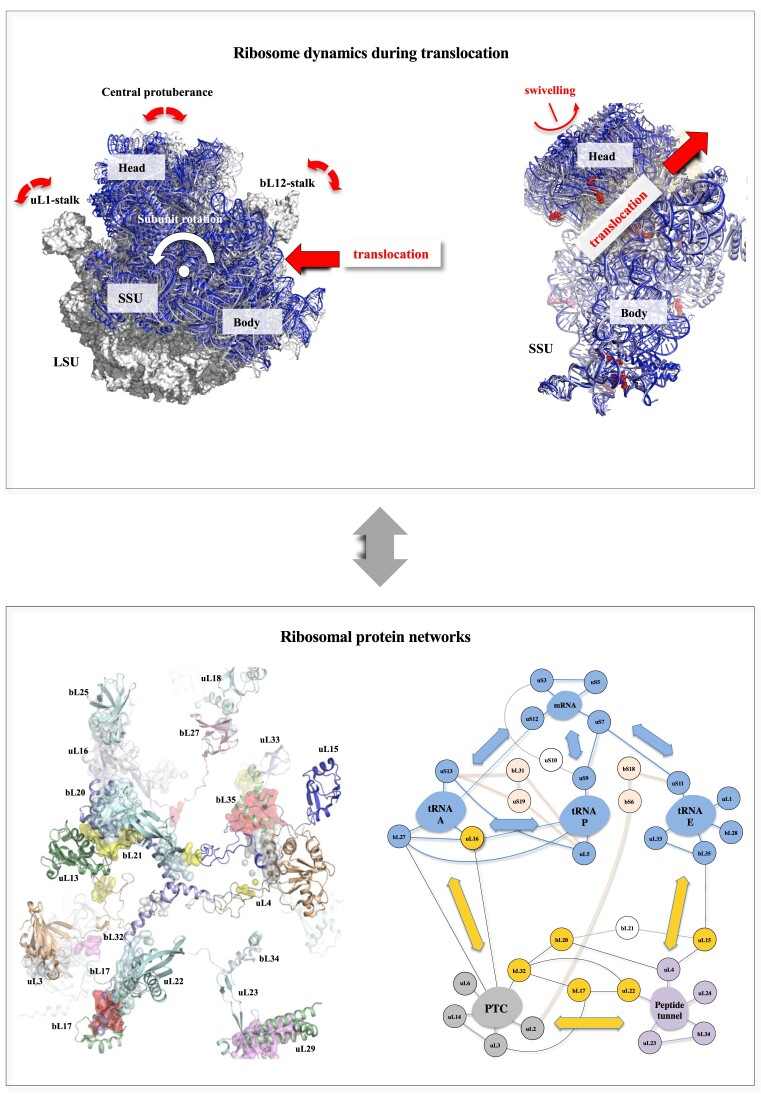
The relationship between ribosome dynamics during translocation and ribosomal protein networks. The aim of this study is to understand how r-protein network may contribute to coordinate ribosome dynamics. Top left panel: Summary of the main ribosomal motions during the translocation. The LSU in the classic unrotated state is represented with a white surface (PDB_id: 7n1p). The subunit rotation (the counter clockwise rotation of the 30S subunit relatively to the 50S subunit) is illustrated by a set of cartoons of the SSU during the six steps of the translocation described in the study of Rundlet *et al.* (2021) [48] with different colours. The colour code is maintained throughout the manuscript: white: step 1 (7n1p); light blue: step 2 (7n30); slate: step 3 (7n2u); blue: step 4 (7n2v); deep blue: step 5 (7n2c) and wheat: step 6 (7n31). Top right panel: Superimposition of the SSU observed in the six steps to highlight the swivelling motion. The direction of the translocation is represented by red arrows. Bottom left panel: Summary of the main properties of the r-protein networks. The r-proteins of the LSU of the bacterial ribosome of *T. thermophilus* (PDB_id: 4v8i) are represented by cartoons. The network interactions are represented by coloured surfaces. Bottom right panel: Schematic representation of the connectome of the bacterial ribosome where r-proteins are grouped into module around functional centers. The connections between the different functional modules are represented by arrows (adapted from Timsit *et al.*, 2021 [102].

Subsequently, ribosomal dynamics play a critical role in the next phase: the translocation of the peptidylated tRNA-A and the deacylated tRNA-P into the P and E sites, respectively (Figure 1A, and Supplementary Figures S1 and S2 and Supplementary Text 1B). Ribosomes perform a complex choreography to ensure that tRNAs and mRNAs move in perfect synchrony, preventing the loss of the reading frame. Intersubunit rotations (38–40), 30S head/body swivelling (41) and 50S stalk movements, in combination with more subtle shifts in ribosomal RNA (rRNA) helices, ensure the directional movement of the substrates (21,42,43). The tRNA translocation occurs in two steps: initially, it moves spontaneously on the LSU, followed by a catalyzed movement on the SSU by EF-G.GTP. First, peptide bond formation triggers the spontaneous movement of the acceptor stems of the peptidyl-tRNA and deacylated tRNA from the A and P sites to the P and E sites on the LSU, respectively. The spontaneous tRNA movements in their hybrid A/P and P/E states are coupled with subunit rotation, creating a ratchet-like motion (44–47). Hybrid state ribosomes serve as intermediates in translocation and are the preferred substrate for EF-G-catalyzed translocation. EF-G and GTP hydrolysis then convert the thermally driven motions of the ribosome and tRNAs into forward translocation along the 30S subunit. Recent advancements in time-resolved cryo-electron microscopy have enabled detailed observation of these translocation steps (48,49). While the exact role of GTP hydrolysis remains incompletely understood, these studies converge on the idea that the ribosome behaves like a ‘Brownian machine,’ driven by GTP hydrolysis, with rRNA largely responsible for its dynamics (17,50,51). The prevailing view is that translation factors serve to rectify and constrain the ribosome’s intrinsic dynamics (17,48,49). These dynamics are influenced by rRNA architecture, which is fine-tuned by its sequence to organize both flexible and rigid regions.

The comparison of snapshots of distinct elongation intermediates has revealed the existence of major Centers of Motion (CMs), which can be viewed as hinges around which rRNA helices exhibit mobility with varying amplitudes (52–55). Equivalent hinges have been identified in both *Escherichia coli* and *Saccharomyces cerevisiae* rRNA, demonstrating their conservation across kingdoms. Most CMs are composed of weak non-canonical base pairs, such as G.U, G.A or A.A pairs, found in the 16S and 23S rRNA (56–58) (Supplementary Figure S3). These CMs are often located in the A 3WJ family (59,60) and exhibit specific tertiary RNA–RNA interactions that confer flexibility (Supplementary Figure S4). The precise arrangement of CMs along the 16S and 23S rRNAs defines the points of articulation around which ribosomal domains move relative to each other during translocation (Supplementary Figure S5). They play a crucial role in the movements of the uL1 and bL12 stalks, as well as the central protuberance of the LSU (Supplementary Figure S6A). The highly mobile SSU contains additional CMs that play a subtler role (Supplementary Figure S6B and C). These CMs govern finer movements that reorganize the internal structure of the head and body during translocation (Supplementary Figure S7 and Supplementary Text 1B).

Despite extensive data on ribosome dynamics, our understanding of the regulatory mechanisms underlying these processes remains incomplete. For example, how does the ribosome not only transmit but also integrate multiple signals from distant functional sites so rapidly to synchronize its dynamics? Two fundamental and intrinsically linked questions remain unresolved: (i) how are the ribosome’s movements synchronized? and (ii) how does information transit between the ribosome’s distant functional sites? A number of studies have revealed RNA-mediated communication (61–65) between remote ribosomal functional sites, including the Peptidyl Transfer Center (PTC), tRNA binding sites (66–71) and the peptide tunnel (8,72–75). However, due to a generally rRNA-centric view of ribosome function and dynamics, the role of r-proteins in these processes has often been overlooked. It is widely accepted that r-proteins, which constitute approximately one-third of the ribosome (76–79), are critical for its assembly and stability primarily through their positive charges and extensions (80–83). However, r-proteins warrant further attention for several reasons. First, some r-proteins have been shown to play active roles in allosteric communication between functional sites (84–93). Additionally, r-proteins such as uS3, uS4 and uS5 have been implicated in mRNA helicase activity (23,94). Molecular dynamics studies have also suggested that r-proteins are involved in the elongation process. Specifically, the triad comprising uL16, uL5 and uL1 has been shown to control the trajectory of tRNA through the ribosome and reduce energetic barriers to tRNA interactions (95). Furthermore, the roles uS5 (54) and uS12 (96) in head swivelling and elongation have also been highlighted. Despite these findings, with the exception of a single study on the role of a protein in 16S rRNA assembly dynamics (97), there is a clear gap in our understanding of the involvement of r-proteins in mature ribosome dynamics during translocation. It is currently believed that r-proteins are largely set in motion passively by the rRNA helices to which they are firmly anchored.

Although network theory has been crucial in understanding the diverse interactions between nucleotides and RNA structural modules (66,98,99), the unique features of r-protein networks merit special attention (Figure 1, and Supplementary Figures S8–S9; and Supplementary Text 2) (100–102). These networks, primarily composed of conserved, tiny interactions between r-protein extensions, span the entire ribosome, interconnecting the r-proteins themselves and the functional centers around which they are organized into modules. As these networks become increasingly complex during the evolutionary transition from prokaryotes to eukaryotes, reflecting the growing complexity of eukaryotic ribosomal functions, it has been proposed that they play a role in ribosomal signaling and the coordination of ribosomal dynamics (102,103). Specific motifs, particularly those involving charged and aromatic residues at protein interaction interfaces, suggest that these networks transmit signals through a distinct form of allostery, one that is driven by electrostatic changes.

The role of r-protein networks in rRNA dynamics remains incompletely understood, and the mechanisms by which they may influence rRNA movements have yet to be fully elucidated. Several key questions remain unanswered: (i) how do rRNA motions affect the r-proteins? (ii) Conversely, do r-proteins play a role in rRNA dynamics? (iii) How does r-protein network signaling contribute to overall ribosome dynamics? Our study provides initial insights into the function of r-protein networks in regulating ribosome dynamics, bridging the fields of ribosome dynamics and r-protein networks (Figure 1). We first demonstrate that the major CMs are consistently in contact with r-proteins, which link them into a complex network. To better understand the role of r-proteins bound to rRNA hinges, we conducted a systematic analysis of rRNA motion around each r-protein, based on recent cryo-electron microscopy studies (48). This analysis reveals substantial rRNA mobility around r-proteins during translocation and suggests a model in which periodic, context-induced electrostatic changes in r-proteins modulate rRNA–r-protein interactions, potentially synchronizing ribosome dynamics.

## Materials and methods

### Major rRNA CMs and r-protein binding

By comparing the structures of ribosomes at different stages of translocation, two research groups have identified the presence of major CMs within the 16S and 23S rRNA of *Thermus thermophilus* ribosome (52–55). These hinge points, which constitute articulations between rigid rRNA helices, have been identified through the alignment of the crystal structures of EF-G (52) or EF-Tu (53) bound and unbound ribosomes. They are typically associated with weak structural motifs, including non-canonical base pairs, bulges, particular tertiary RNA–RNA interactions and three-way junctions (56–59) (Supplementary Figures S3–S5). Two hinge points control the swivelling movement of the 30S head (54) and recurrent motifs are associated with the movement of the uL1 stalk during translocation (55). A synthesis of the works of these two groups lists 15 CMs in the SSU (named pX) and eight CMs in the LSU (named pLX) according to the number (X) of a base of the hinge (Supplementary Table S1 and Supplementary Figures S3–S6). We verified that these were indeed movement zones by superimposing different *T. thermophilus* and *E. coli* ribosome structures at different stages of translocation (Supplementary Figures S6–S7). We characterized the interactions of each CM with r-proteins according to distance criteria and analysed the geometry of their interactions and the structural motifs facing them. In order to identify the phylogenetically conserved amino acids in contact with the CMs, we utilized the alignments proposed by Yutin *et al.* (104) and the scripts developed in our previous article (102). The new ribosomal r-protein nomenclature is utilized for the naming of ribosomal proteins (105).

### Analysis of rRNA movements around r-proteins

To determine how rRNA moves around each protein during the early stages of translocation, we used the highest resolution cryo-electro-microscopy data available in the PDB that describe the early translocation steps (48). To measure the extent of rRNA helix displacement in the vicinity of r-proteins during the six steps defined by Rundlet *et al.* (2021), we used a procedure to extract buried information from high-resolution structures obtained at step 1–6 (PDB_id: 7n1p, 7n30, 7n2u, 7n2v, 7n2c and 7n31, respectively) (Supplementary Table S2 and Supplementary Figures S1–S2).

Previous studies on ribosome dynamics have primarily focused on the movement of rRNA domains by structurally aligning 16S or 23S rRNA regions of ribosomes at different functional stages. In contrast, to investigate the movement of rRNA around r-proteins, we compared ribosome structures at different translocation stages by aligning the structures of the r-proteins themselves. To characterize the RNA/protein mobility, we employed the following approach: we superimposed the coordinates of a given r-protein from steps 2–6 onto its corresponding coordinates from step 1, along with a surrounding layer of rRNA within a 10–15 Å radius of the protein. Only the Cα atoms of the r-proteins were used for the superposition to ensure accurate 3D alignment, with the surrounding rRNA treated as a rigid block. This allowed us to compare the position of the rRNA around the r-protein at different stages (steps 2–6) relative to its position at step 1. To quantify the major rRNA motions around the r-proteins, we measured the displacements of phosphorus atoms in the rRNA sugar-phosphate backbone at steps 2–6 relative to their initial positions at step1. We defined displacements greater than 1 Å as significant, indicating notable shifts in the phosphate groups. To apply this procedure systematically, we developed PyMOL scripts (106) that automate the analysis for each r-protein in both the SSU and LSU. These scripts calculate and list the interphosphorus distances between equivalent atoms at steps 2–6, relative to their position in step 1. Additional scripts were written to systematically represent the movements of rRNA around each protein by measuring the distance of the phosphorus atoms relative to the CM of the r-protein as well as its ionisable groups (Glu, Asp or His). Beyond the initial approach, which identifies large displacements by measuring the phosphorus atom shifts, we also observe more subtle rearrangements in the rRNA backbone and base conformations. This detailed analysis provides several types of data including the displacement of phosphate groups in relation to their original positions at step 1 (Table 2), in comparison to the COMs of r-proteins and in relation to the ionisable groups (His, Glu and Asp) of r-proteins (Supplementary Data 1 and 2).

### Calculation of the centrality of r-protein-CMs networks

Centralities are node and edge features that are computed from the structural information -the connectome- of a network. They capture the relative importance of nodes and edges with respect to diverse static and dynamic properties of a network. As in our previous study, which described the properties of the ribosomal protein networks of the three kingdoms, we calculated the centralities on the basis of their definitions in graph theory (102). Here, we consider two centralities: Betweenness centrality and Eigen-vector centrality in non-directed graphs G = (V,E) with V a set of vertices and E a collection of edges; an edge e ∈ E is a subset of E of cardinality 2. Each r-protein, CM, tRNA and functional center is associated to a node (Supplementary Table S3). When there is more than one contact between the one of these elements there is a edge between both. Let us denote for *v*,*v*_1_, *u* ∈ *V* the number of shortest paths between *v* and *v1* passing through *u* as σ*_v,v1_ (u)*. The Betweenness centrality measures how many central paths pass through the node and is defined as *C^B^(u)* = **Σ** σ*_v,v1_ (u) /* σ*_v,v1_*. Eigenvector centrality is a normalized eigenvector of the adjacency matrix of G with eigenvalue 1, *C^B^(u)* = *x(u)* where *Ax = x*. Both centralities are computed using the Python package NetworkX (107). Specifically, the largest connected component of the connectome is extracted using ‘networkx.connected_components’; ’largest’ refers to the component with the highest number of nodes. Betweenness centrality is then computed for this largest component using the function ‘networkx.algorithms.centrality.betweenness_centrality’, while eigenvector centrality is computed using ‘networkx.eigenvector_centrality’.

## Results

### The rRNA CMs are systematically bound by r-proteins

**Figure 2.**
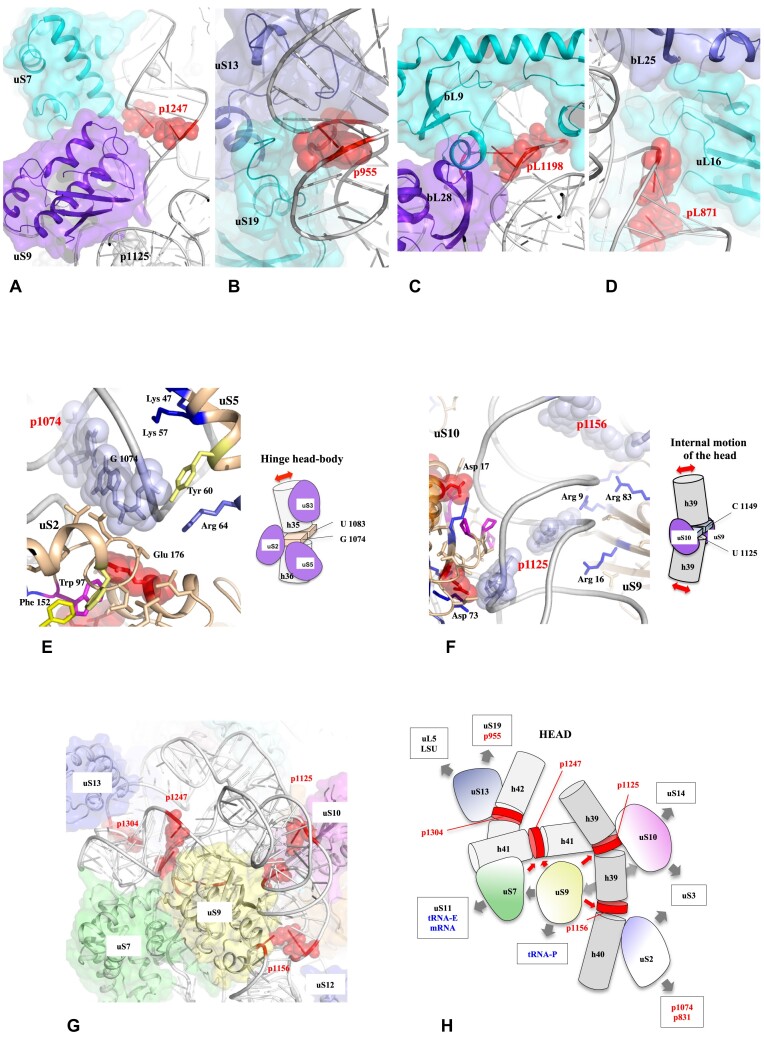
Properties of the interactions between CMs and r-proteins in the bacterial ribosome. Interactions between the CMs (red spheres) of the SSU and LSU of *T. thermophilus* ribosome (pdb id : 4v9h) and dimer of r-proteins (blue cartoons and surfaces): (**A**) uS7-uS9 and p1247. (**B**) uS13-uS19 and p955. (**C**) bL9-bL28 and pL1198. (**D**) uL16-bL25 and pL871. Asymmetry of charge distribution in CMs–r-protein interactions. (**E**) CM p1074 and uS2 and u5. (**F**) CM p1125 and uS9 and uS10. Ensemble of local CMs–r-protein interactions with the SSU head. (**G**) Interactions around the protein uS9 (**H**) Schematic representation of the network of interactions around uS9 (**G**). The CMs are represented in red and the r-proteins are represented by coloured cartoons and surfaces.

Our study firstly reveals that most of the CMs that form the main hinges between rRNA helices during ribosome translocation (52–55) are bound by r-proteins (Figure 2, Table 1, and Supplementary Table S1 and Supplementary Figure S10). Notably, of the 23 CMs of the bacterial ribosomes, 19 establish direct and specific interactions with r-proteins, while three establish an indirect interaction (contact occurring <1 helix turn from the CM). In SSU, except p1212 and p201, all the CMs are directly connected by r-proteins. In the LSU, except pL1528, the majority of CMs are also in direct contact with r-proteins. Only pL1907, which resides at the interface of the two subunits, does not interact with an r-protein. However, G1907, which forms a G•U pair with U1923, also plays a crucial role by interacting with the tRNA helix D at the P site. Conversely, the majority of SSU r-proteins and two-thirds of LSU r-proteins are involved in interactions with CMs (Table 1). Two types of direct CM-r-protein interactions are observed. About half the time, the two bases of the CMs are bound symmetrically by one or more r-proteins. In the other half, a single nucleotide (the purine) of the base pair is targeted by a protein specific interaction (Supplementary Table S1). Interestingly, CMs are also frequently observed at the interface of two r-proteins, in such way that the RNA hinge zones and r-protein dimers correspond (Figure 2A–D). Although it is most often the globular domains that interact with CMs, they are sometimes contacted by extensions of certain r-proteins such as uS9, uS13 and uS19 (Supplementary Figure S10). Our analysis indicates that in addition to their special sequences and tertiary RNA–RNA interactions (Supplementary Figures S4–S5), CMs also need to bind to proteins, probably to allow for a greater degree of control over their mobility. This remarkable finding prompted us to conduct further analysis of CM-r-protein interactions in order to gain a deeper understanding of the intriguing link between r-protein binding, rRNA flexibility and the overall ribosome dynamics.

**Table 1.** List of the r-protein–CMs interactions in the SSU (A) and the LSU (B). The conserved residues and motifs in direct or indirect interaction with the CMs and the RNA helices are indicated. WHED is noted ‘1’ when the motifs Glu, Asp (ED), Trp (W) or His (H) are in contact with the rRNA around the CM

Protein	Hel	Direct	base pair	contact type	WHED	Indirect	Base pair	Contact type	WHED
uS2	35/36	p1074	G1074-U1083	**ABA/ trp, thr > G1074**	1			**Cluster ED/ glu 170 > A 1101**	1
uS2	26/26a	p831	U831-G855	**ABA/ trp, his > U831**	1				
uS2	40	p1156	G1156-A1179	lys grip > 1158, arom > 1171					
uS3	35/36					p1074	G1074-U1083	ABA/ his, pro > C1109	1
uS3	40					p1156	G1156-A1179	idem	1
uS3	39					p1125	U1125-C1149	idem, (interface uS3-uS2 : glu 206- glu 134)	1
uS4	16/17					p441	A441-G493	glu150-lys151 > 491, his 123 > 439, αH- Cter > A439	1
uS5	35/36	p1074		cation-π, arg64-tyr60 > G1074					
uS5	28	p1394		arg 24 > 1396					
bS6									
uS7	43	p1351	U1351-G1371	lys 35 + αHel-Nter > U1351					
uS7	41	p1247	u1247-G1290	lys 35 + αHel-Nter > G1290					
uS7	42					p1304	G 1304 (A1332)	basic grip/ arg 115 + 119 + SB > A1239	1
uS8	21					p593	G593-U646	ABA/ 3 pro > > G588 and C 643	1
uS8	35/36					p1074	G 1074 - U 1083	interface uS8-uS2	
uS8	26/26a					p831	U 831-G 855	acid cluster/SB > G825	1
uS9	39	p1125	U1125-C1149	arg grip/ thr > 1149					
uS9	40	p1156	G1156-A1179	basic grip/ thr > 1179					
uS9	41	p1247	U1247-G1290	loop NS/polar/ α-hel-N-ter > G1290					
uS9	43	p1351	U1351-G1371	**asp 75, asp105, glu 110 in SB > G1371**	1				
uS9	30					p955	U955-A1225	ser/gln/arg > U1232	
uS10	39	p1125	U 1125- C 1149	Asp 73 in SB + 3 pros in loop > U1125	1				
uS10	40					p1156	A 1179 - G 1156	ABA/ his 68 > A1152	1
uS10	43					p1351	U 1351 -G 1371	ABA/ his 62/trp 61(uS14) > G1368	1
uS11									
uS12	44					p1394		asp 92 in SB/3 pros > 1491 H44	1
uS13	30	p955	U 955 - A 1225	cluster basic/ arg 78, arg 91 > A 1225					
uS13	42	p1304	G 1304> (A1332)	ABA/ tyr 23 > U 1330, His 92 > 1309	1				
uS13	41					p1247		basic grip lys13/arg 44 > C 1296	
uS14	33b/34					p1212	U 1212 extra-hel	basic grip > U 1217, cat-pi 1219	
uS14	43					p1351	U1351-G1371	composite interface SB + WH with uS10, uS9 > C1369	1
uS14	40					p1156	**A 1179 - G 1156**	composite interface SB + WH with uS10, uS9 > C1116 + 1186	1
THX	42	p1304	G 1304> (A1332)	asp 5 > P 1304	1				
THX	43	p1351	U1351-G1371	Cation-π lys3/trp 14 > U1351					
THX	41					p1247	U1247-G1290	basic grip : arg 9 and arg 10 > C1244	
uS15	21	p593	G 593-U 646	asp21, glu26 > U751; tyr69, asp74, arg77 > G752	1				
bS16	17					p441	A441- G493	ABA/ tyr 17, tyr39, pro 41, asp29 > A451	1
bS16	6					p62	U 62 - G105	SB asp 23/arg 25 > C110	1
uS17	21	p593	G593-U646	glu 24 > U646 and G585	1				
bS18	H26/H26a		U 831-G 855			p831	U 831-G 855	basic grip > U835	
uS19	30	p955	U 955 - A 1225	arg 78 > A1225, tyr 80 > U955		p955	U 955 - A 1225	ABA/ his 57, trp 34, tyr 52 > G1220	1
uS19	42					p1304	G 1304 > P (A1332)	his 69 and glu 73 and phe 74 > C 1320, trp 34 and arom cluster > 1120	
uS19	33b/34					p1212	U1212 extra-hel	trp 34, tyr 52, his 57 > 1219	1
bS20	8	p149	A 172 -A 149	asp 64, lys 65 > C 177					
bS20	6	p62	U 62 - G105	basic grip lys 14, arg 15 > G105					
bS20	10					p201	C 201-G216	asp 64, his73 > A 195	1
**protein**	**Helix**	**Direct**	**Base pair**	**Contact type**	**WHED**	**Indirect**	**Base pair**	**Contact type**	**WHED**
uL1		none							
uL2	34	pL703	U703-G728	trp 214 + lys 208, thr 10 > G728	1	pL703	U703-G728	lys 7 > A705, his 58 > A 1556 (extrahel) > G728	1
uL2	79	pL2198	A2198, C2196-G2093	3 ABA /pro > U2203, G2206, G2223	1	none			none
uL3		none			none	none			none
uL4		none			none	none			none
uL5	84	pL2298	A2298-G2318	basic grip lys74,75 > A2298	none	pL2298	A2298-G2318	ABA : asp 126 166 > A2303, Lys 36 > 2315	1
uL6	42					pL1032	A1032-G1122	arg 59 (NC) > A1035	none
bL9	79	pL2198	A2198, C2196-G2093	tyr25, tyr 29 > A2198	none	none			none
uL10		none			none	pL1032	A1032-G1122	Lys 8 (coli) > C1044	none
uL11		none			none	none			none
bL12		none			none	none			none
uL13	42	none	A1032-G1122		none	pL1032	A1032-G1122	lys66, lys 70 > 1022, 1144, 1142	1
uL14		none			none	none			none
uL15		none			none	none			none
uL16	42	pL1032	A1032-G1122	glu 116 … A 1032, glu 111 > C 1116, lys 128 > A 1029	1	none			none
uL16	38	pL871	U 871- G 906	phe 29 > G 906, phe69 > U871, grip arg 5,6 > U871, ABA/pro > G906	1	none			none
bL17		none			none	pL1528	A 1528-A1542-G1448	basic grip arg 63, arg 64 > U 1453 > G 1448 > A 1542	1
uL18	84				none	pL2298	A2298-G2318	basic grip arg 13,15 > 2296, ABA/pro > C2293	1
bL19		none			none	none			none
bL20	42	none			none	pL1032	A1032-G1122	basic cluster > H41 > G1122	none
uL22		none			none	none			none
uL23	9, 10	none			none	pL149	A149-G177	thr 35 > C143, lys 40 > G 139	none
uL24		none			none	none			none
bL25	38	pL871	U 871- G 906	non conserved residues > G 875	none				none
bL25	42	none			none	pL1032	A1032-G1122	non conserved residues > C 1119, C 1040	none
bL27	84	none			none	pL2298	A2298-G2318	lys 46 > 2334	none
bL28	79	pL2198	A2198, C2196-G2093	ile 67 > 2091 non conserved residues (arg 50) > A 1199	none				none
bL28		none			none	pL149		non conserved basic residues > G152	none
uL29		none			none	none			
uL30		none			none	none			none
bL31		none			none	none			none
bL32		none			none	none			none
uL33	84	none			none	pL2298	A2298-G2318	Lys 25, lys 27 > A 2287	none
bL34	9, 10	pL149	A149-G177	arg 29 > G178; arg 19, phe 18 > U120 > A149	none				none
bL35		none			none	none			none
bL36	42	pL1032	A1032-G1122	lys 8, val 16 + 23, arg 18 > A 1032	none	none			none

### Conserved protein motifs and charge patterns towards CMs

The systematic analysis of the r-protein motifs interacting with CMs reveals a high degree of phylogenetic conservation across species (Supplementary Table S1 and Supplementary Figure S11). These motifs have very specific electrostatic characteristics that raise interesting questions about how they interact with rRNA. For example, although protein–RNA interactions are generally mediated by basic residues (108,109), a significant number of r-proteins exhibit the presence of acidic residues in the vicinity of the CMs. This unusual trait may potentially result in the repulsion of phosphate groups (Figure 2E and F, and Supplementary Table S1 and Supplementary Figure S12). The negatively charged residues are frequently observed within one helix turn of the CMs, while basic or aromatic residues generally interact closely with the base pair of the rRNA hinge. These acidic residues are most often included in ABA (Acid, Aromatic and Basic) motifs where residues form salt-bridges (SB), cation– or anion–π interactions (110–112). They are frequently combined with strictly conserved proline, histidine or tryptophan (Table 1, and Supplementary Table S1 and Supplementary Figures S11–S12). Similarly, while it is common to observe the N-ter ends of α-helices interacting with the nucleic acid backbone, the interaction of their C-terminal ends, with a negative component, is much rarer. However, in the case of uS4, uS7, bS16, bS18 and uL5, the C-terminus is clearly directed towards the rRNA in the vicinity of CMs. Another distinctive feature is that when inserted between multiple rRNA helices, the r-proteins expose motifs with different electrostatic properties to each of them (Figure 2E and F). The specific distribution of well-conserved charged residues on either side of the CMs suggests that the electrostatic properties of the r-proteins are a primary determinant of rRNA flexibility. It seems plausible that the various categories of protein motifs serve discrete functions in CM-protein interactions.

### Properties of the network formed by the r-proteins, CMs and functional centers of the ribosome

**Figure 3.**
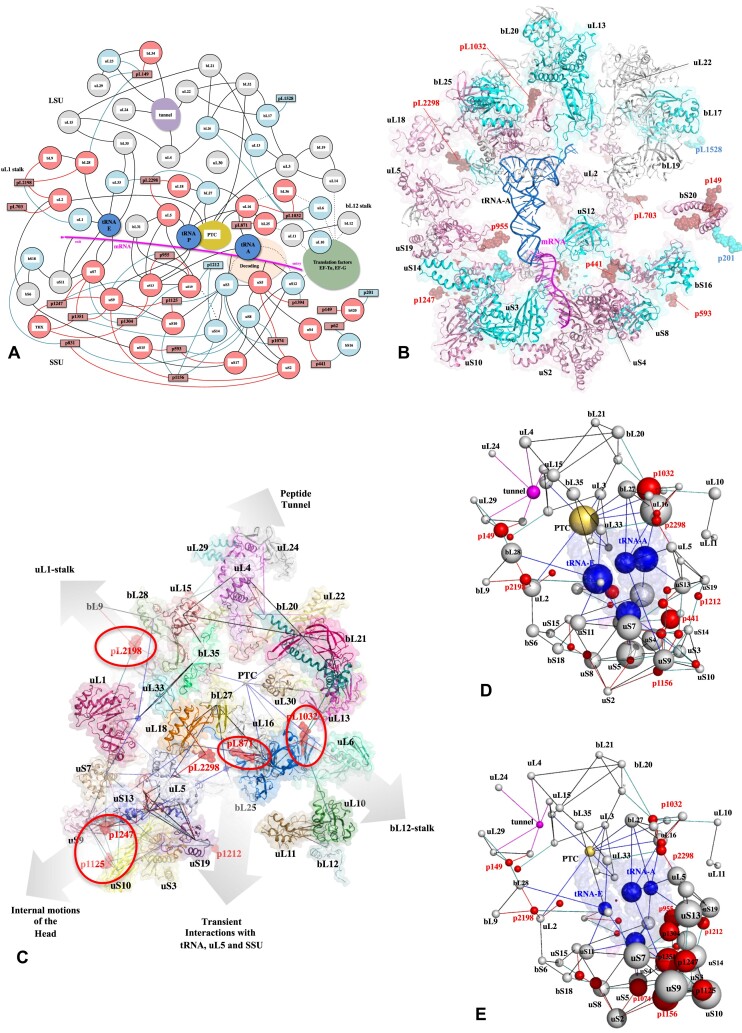
Network between r-proteins, CMs and functional centers of the bacterial ribosome. (**A**) schematic and (**B**) cartoon representations of the connectome linking the bacterial r-proteins to the CMs and the functional center. R-proteins that make direct interactions with CMs are represented in pink. R-proteins that interact indirectly with CMs are represented in cyan. (**C**) Network drawing (black lines) between the r-proteins and CMs, indicating the moving domains controlled by the CMs. (D) and (E) Network of r-proteins (grey spheres), functional centers (yellow spheres) and motion centers (red spheres) in the bacterial ribosome. The diameter of the spheres that correspond to the centers of mass of each component are proportional to the value of the maxima of centrality (**D**) Betweenness centrality (**E**) EigenVector centrality.

Knowing that r-proteins weave a web that runs throughout the ribosome and links r-proteins together as well as functional centers (102) (Supplementary Figures S8–S9 and Supplementary Text 2), it follows that the r-protein–CMs interactions form part of a vast network. For example, uS9, which plays a critical role in contacting directly four CMs in the head domain, interacts with uS7 via its globular domain and uS10 and tRNA-P via its extension. However, uS7 also contacts tRNA-E and uS11, while p1156 contacted by uS9 also interacts with uS2, which in turn contacts uS3 and two other pivots (Figure 2G and H). When integrated to the whole ribosome, all these interactions can be described in a network of 83 nodes (54 proteins, 6 functional centers and 23 CMs) (Figure 3A and B, and Supplementary Table S3 and Supplementary Figure S13). In this network, the r-proteins play a key role in connecting functional sites, such as tRNA binding sites, the PTC or the peptide exit tunnel, on the one hand, and the CMs that control ribosome dynamics, on the other (Figures 1 and 3, and Supplementary Figure S14). The network that is more densely connected in the SSU and in particular in the ‘Head’ domain highlights the diverse roles of r-proteins as intermediaries, facilitating the formation of pathways between the CMs and the functional centers of the ribosome. Figure 3C illustrates how r-proteins interconnect the CMs that control the movements of the LSU stalks and the head/body swivelling to the functional centers. Two opposite situations can be identified. Firstly, a single r-protein may interact with multiple CMs. In the SSU for example, uS2 bridges two CMs from the head domain (p1074 and p1156) with those in the body (p831) and therefore establishes a link between key RNA elements that control both swivelling and the internal mobility of the two domains. In the LSU, uL2 makes direct contact with two very distant CMs. One is via its globular domain (pL2198, located at the base of the uL1-stalk), while the other is via its C-terminal extension (pL703, situated at the base of the helix H34, which in turn contacts uS15 in the SSU). uL16 also interacts with two hinges, namely pL871 and pL1032 that control the mobility of helix H38 (involved in transient contact with tRNAs) and the bL12 stalk, respectively. Second, there are inverse situations, where one CM is at the crossroads of several r-proteins. To illustrate, the CMs situated at the base of the LSU stalks are trapped by a number of proteins: pL2198 at the base of the uL1 stalk is surrounded by uL2, bL28 and bL9, while pL1032 at the base of the bL12 stalk is clamped by uL16 and bL36. The case of CM p593 is of particular interest as it is in direct contact with uS8 and uS17, in close proximity to their connection with the N-terminal extension of uS12, which runs across the SSU (Supplementary Figure S15).

Graph theory reveals nodes with particular properties in terms of centrality (113). Betweenness (BC) that defines the number of times a node is on the shortest path between any two other nodes in the graph is a key determinant of network functionality in that it measures the extent to which a node reflects the information spread on the network. On the other hand, Eigenvector centrality (EC) is recognized as a good measure of a node's influence on others when the network supports an activity (114,115). Previous studies in which rRNA was considered as nucleotide network revealed interesting correlations between graph properties and ribosome functionality (99). In another study, graph theory revealed that in the networks composed of r-proteins and functional centers, the Peptidyl Transferase Center (PTC) which catalyses the peptidyl-transfer reaction during protein synthesis (116) is maximum BC in the three kingdom's ribosomes (102). Here, using the same method as in our previous article (102), we show that adding the 23 CMs to this network has little impact on BC maxima, which remain the PTC and uL16, located in the LSU (Figure 3D; Supplementary Figure S16). Assuming that these transfers take place preferentially via the shortest paths, PTC and uL16 still have a large influence on signal transfers in a network composed by r-protein, functional centers and CMs. In contrast, the inclusion of CM nodes profoundly modifies EigenVector (EV) centrality maxima. uS9, uS3, uS10, uS7 and two CMs, p1156 and p1351, all located in the Head becomes EV maxima, which correspond to the nodes that have the greatest dynamic influence on the network (Figure 3E; Supplementary Figures S16-S17). Graph theory therefore indicates that each r-protein and CMs have distinct roles, in terms of information transfer on the one hand and their physical influence on the network on the other (Supplementary Figure S17).

In summary, the first part of our study shows that the ribosome's major CMs are bound and interconnected by r-proteins with which they form a dense network. The patterns of conserved protein motifs that interact with the CMs raise intriguing questions about the dynamic r-protein/rRNA interplay and their roles in the overall ribosome dynamics. The second part of our study will attempt to elucidate the role of CMs-protein interactions during the translocation.

### Part 2: how does rRNA move around r-proteins?

#### An r-protein centric view of ribosome dynamics during translocation

In order to gain insight into the role of r-proteins in the dynamics of rRNA, a systematic analysis was conducted to examine the mobility of the rRNA helices relative to each r-protein (considered as fixed reference points) within the structure of translocation intermediates. To achieve this, we have conducted a reanalysis of the data from a time-resolved cryo-electron microscopy study (48) which provided a comprehensive yet primarily RNA-centric account of the six steps involved in the translocation process. The rRNA dynamics associated with mRNA translocation involve, on one hand, large-scale conformational changes such as intersubunit rotation, head swiveling and movement of the LSU stalk (Figure 1, and Supplementary Figures S1–S2) and on the other hand, more subtle internal movements within both the LSU and SSU (Supplementary Figures S6–S7). These steps, beginning with the non-rotated classical state ribosome (Step 1), progress to the two hybrid-states (Step 2 and 3), the EF-G-bound stages (Step 4 and 5) and culminate in the back-swivelling stages where tRNAs are moved into its new position (A in the P site and P in the E-site) (Supplementary Table S2 and Supplementary Figures S1–S2).

**Figure 4.**
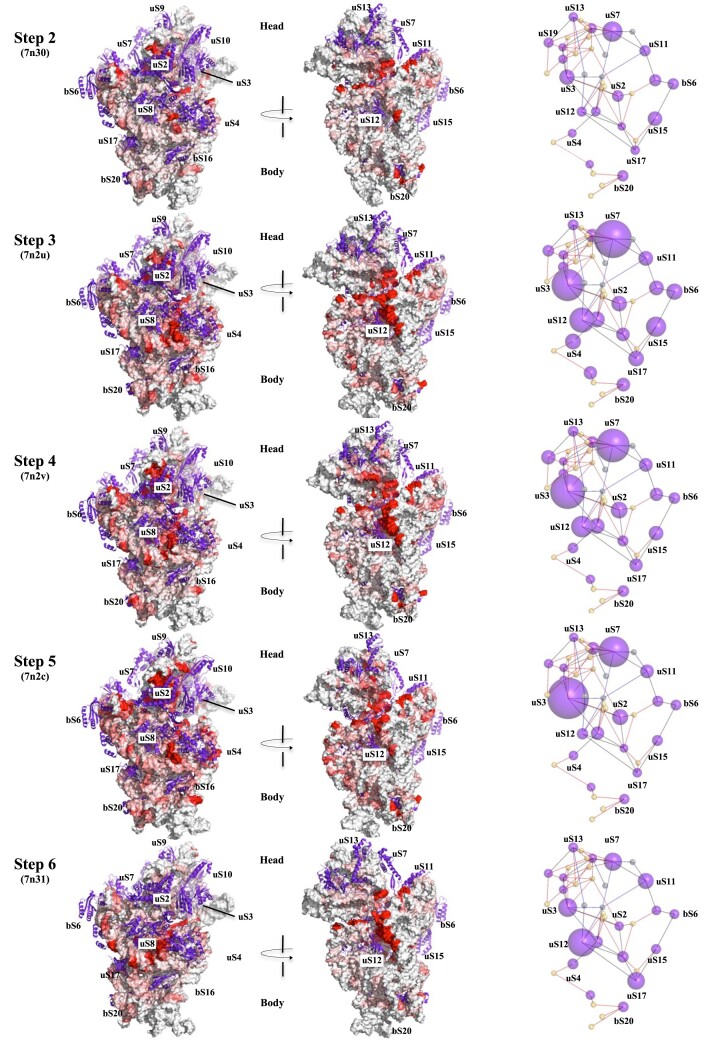
Spatio temporal map of rRNA–r-protein motions in the SSU. During the early translocation steps of *E. coli* ribosome. SSU map of 16S RNA–r-protein movements observed at step 2–6, relatively to step 1 (PDB_id: 7n1p). Surface representations of the 16 RNA coloured from white to red according to the measured phosphorus–phosphorus displacements of each nucleotide, from step 1 to step 6, around each r-protein (represented by violet cartoons). Right panel: the network of CMs (small yellow spheres) – r-proteins (violet spheres whose diameters are proportional to the sum of rRNA displacements around them (within a distance of 15 Å).

To identify the main movements of RNA relative to r-proteins (RNA/protein mobility), we measured the displacements of the phosphorus atoms in the sugar-phosphate backbone during steps 2–6, relative to their initial positions around a given r-protein in step 1, the non-rotated classical-state ribosome (7n1p). Overall, this r-protein centric-view of ribosome dynamics provides a spatio-temporal map of rRNA mobility around the r-proteins in the SSU (Figure 4) and the LSU (Supplementary Figure S18 and Supplementary Data 1 and 2). These cartographic representations offer insights into hitherto unknown aspects of ribosome dynamics during translocation, specifically the timing and manner in which rRNA moves in relation to the various r-proteins. The following sections will seek to provide answers to the following questions. What are the temporal parameters of rRNA/protein mobility during the translocation process? Does rRNA exhibit a tendency to move towards or away from specific r-proteins? What is the impact of localized rRNA–protein motions on the overall translocation processes? What are the role of specific r-protein motifs associated with the observed mobility of rRNA, and what are the underlying molecular mechanisms that govern the mobility of r-proteins and rRNA?

#### Dynamic staging: rRNA moves around each r-protein at different stages of translocation

The first finding of this analysis is that rRNA/protein mobility occurs around >75% of r-proteins during the five-translocation steps. According to the extent and the timing of displacement of the rRNA phosphate groups, r-proteins can be classified into distinct categories (Table 2, Figure 4, and Supplementary Table S4 and Supplementary Figure S18). The r-proteins associated with the greatest rRNA mobility (the higher number of phosphorus atom displacements of >1 Å) are those located at the interface of the two subunits, such as uS7, uL2, bL19, uL14, uL10 and uL11. These r-proteins are transiently in contact with some rRNA regions of the other subunit, which rotates counter-clockwise during translocation (31) (Figure 1, and Supplementary Figure S2). The mobility of rRNA around these proteins therefore essentially reflects the rotation between subunits. Another category is the r-proteins located at the Head/Body interface, such as uS3 that ‘sees’ numerous nucleotides pass along their surfaces during the swivelling process. Proteins such as bL27, uL16 and uL5, which brush against the tRNAs during their transits, also witness numerous displacements of tRNA phosphate groups. Remarkably, rRNA mobility is also significant around most of the r-proteins embedded within each subunit, with the exception of a small fraction of r-proteins (uL22, uL29 and bL34) essentially located in the LSU, around which the rRNA remains unchanged throughout all the steps described by Rundlet *et al.* (48). The initiation of the rRNA mobility around r-proteins occurs in a stepwise manner, analogous to the unfolding of a play. While some rRNA regions are mobile during the five steps of translocation around certain proteins, such as uS7, uL11 and uL2, this mobility may only occur at very specific steps for others, such as uS12, uS17, etc. For example, uL2 and uL5 undergo a change in their RNA environment as early as stage 2. In step 3, uS8 and bS16 are activated in the SSU, whereas in steps 4 and 5, the rRNA is most mobile around uS2, uS9, bS16 and bS20. Interestingly, although there are little RNA/protein movements at step 6 – the back-swivelling step, which corresponds, overall to a return to the unrotated state, there is simultaneous ‘activation’ of the distant but interconnected uS12, uS17 and uS8 r-proteins. It should be noted that while no evidence of RNA mobility was observed near the proteins situated around the tunnel in the ribosome structures examined, it is plausible that this mobility may be initiated during the exit of the newly synthesized peptide (72). Thus, for each r-protein, or for some groups of connected r-proteins within the network, there is a time signature associated with the activation of RNA movement around them.

**Table 2.** Number (Nb) of phosphorus atom displacements >1 Å relative to their initial position in step 1 around each r-protein, in a 15 Å shell at each step 2–6 of the translocation (see also Supplementary Table S1). The ‘rate’ is the ratio of the number of phosphorus atoms with displacements >1Å to the number of phosphorus atoms in the 13Å shell around each r-protein

Protein	Step 2		Step 3		Step 4		Step 5		Step 6	
	Nb	rate	Nb	rate	Nb	rate	Nb	rate	Nb	rate
**uS7**	60	0,56	73	0,76	83	0,89	64	0,81	13	0,12
**uL2**	40	0,13	37	0,12	32	0,1	32	0,1	1	0
**uL11**	37	1	32	0,86	31	0,91	26	0,96	39	1
**uL10**	26	0,79	21	0,6	32	1	32	1	23	0,66
**uL14**	25	0,23	27	0,25	28	0,26	24	0,22	26	0,24
**uL5**	23	0,32	17	0,24	8	0,11	8	0,12	5	0,07
**uS3**	21	0,21	20	0,2	22	0,26	35	0,5	19	0,19
**bL19**	20	0,19	17	0,16	21	0,2	24	0,22	27	0,25
**bL31**	18	0,69	11	0,46	10	0,42	6	0,3	10	0,37
**bL9**	16	0,36	18	0,4	10	0,22	10	0,21	7	0,15
**uL6**	13	0,14	8	0,08	23	0,24	15	0,16	10	0,11
**uS5**	10	0,1	20	0,19	27	0,26	28	0,28	13	0,12
**uS15**	9	0,11	9	0,11	9	0,11	7	0,08	8	0,09
**uS2**	8	0,14	12	0,2	26	0,44	30	0,57	0	0
**bL36**	8	0,09	4	0,04	18	0,21	12	0,14	8	0,09
**uS12**	7	0,04	65	0,38	73	0,44	25	0,15	92	0,56
**uS11**	7	0,07	5	0,05	11	0,12	24	0,27	7	0,08
**bS20**	7	0,05	5	0,04	8	0,06	11	0,09	4	0,03
**uL16**	6	0,04	6	0,04	8	0,06	7	0,05	6	0,04
**bL21**	6	0,06	4	0,04	6	0,06	7	0,07	3	0,03
**uS19**	5	0,07	0	0	3	0,06	0	0	6	0,08
**uL15**	5	0,02	3	0,01	2	0,01	2	0,01	3	0,01
**bL27**	5	0,03	8	0,05	8	0,05	4	0,03	2	0,01
**uS9**	4	0,03	7	0,06	6	0,06	30	0,35	4	0,03
**uS13**	4	0,05	0	0	2	0,03	1	0,02	6	0,07
**bS18**	4	0,08	5	0,1	7	0,14	11	0,22	0	0
**uS4**	3	0,02	9	0,07	7	0,06	12	0,1	11	0,1
**uL4**	2	0,01	2	0,01	1	0,01	2	0,01	1	0,01
**uL33**	2	0,04	0	0	1	0,02	1	0,02	0	0
**uS8**	1	0,01	9	0,1	8	0,09	7	0,08	10	0,12
**uS14**	1	0,01	0	0	1	0,01	3	0,04	1	0,01
**uL29**	1	0,02	0	0	0	0	1	0,02	1	0,02
**uL23**	1	0,01	5	0,05	2	0,02	4	0,04	1	0,01
**uL18**	1	0,01	4	0,05	3	0,04	1	0,01	0	0
**uL13**	1	0,01	2	0,01	4	0,03	3	0,02	2	0,01
**bL35**	1	0,01	1	0,01	0	0	0	0	1	0,01
**bL20**	1	0,01	1	0,01	1	0,01	3	0,02	1	0,01
**uS17**	0	0	7	0,07	18	0,19	4	0,04	33	0,35
**uS10**	0	0	1	0,01	2	0,02	6	0,09	2	0,02
**uL30**	0	0	0	0	0	0	0	0	0	0
**uL3**	0	0	0	0	1	0	0	0	1	0
**uL24**	0	0	0	0	0	0	0	0	1	0,01
**uL22**	0	0	0	0	0	0	0	0	0	0
**bS6**	0	0	2	0,07	0	0	7	0,23	0	0
**bS16**	0	0	4	0,04	2	0,02	5	0,04	1	0,01
**bL34**	0	0	0	0	0	0	0	0	0	0
**bL32**	0	0	0	0	0	0	0	0	1	0,01
**bL28**	0	0	0	0	1	0,01	0	0	0	0
**bL25**	0	0	0	0	4	0,07	5	0,09	0	0
**bL17**	0	0	0	0	0	0	0	0	0	0

#### r-protein–rRNA distance/approach cycles

**Figure 5.**
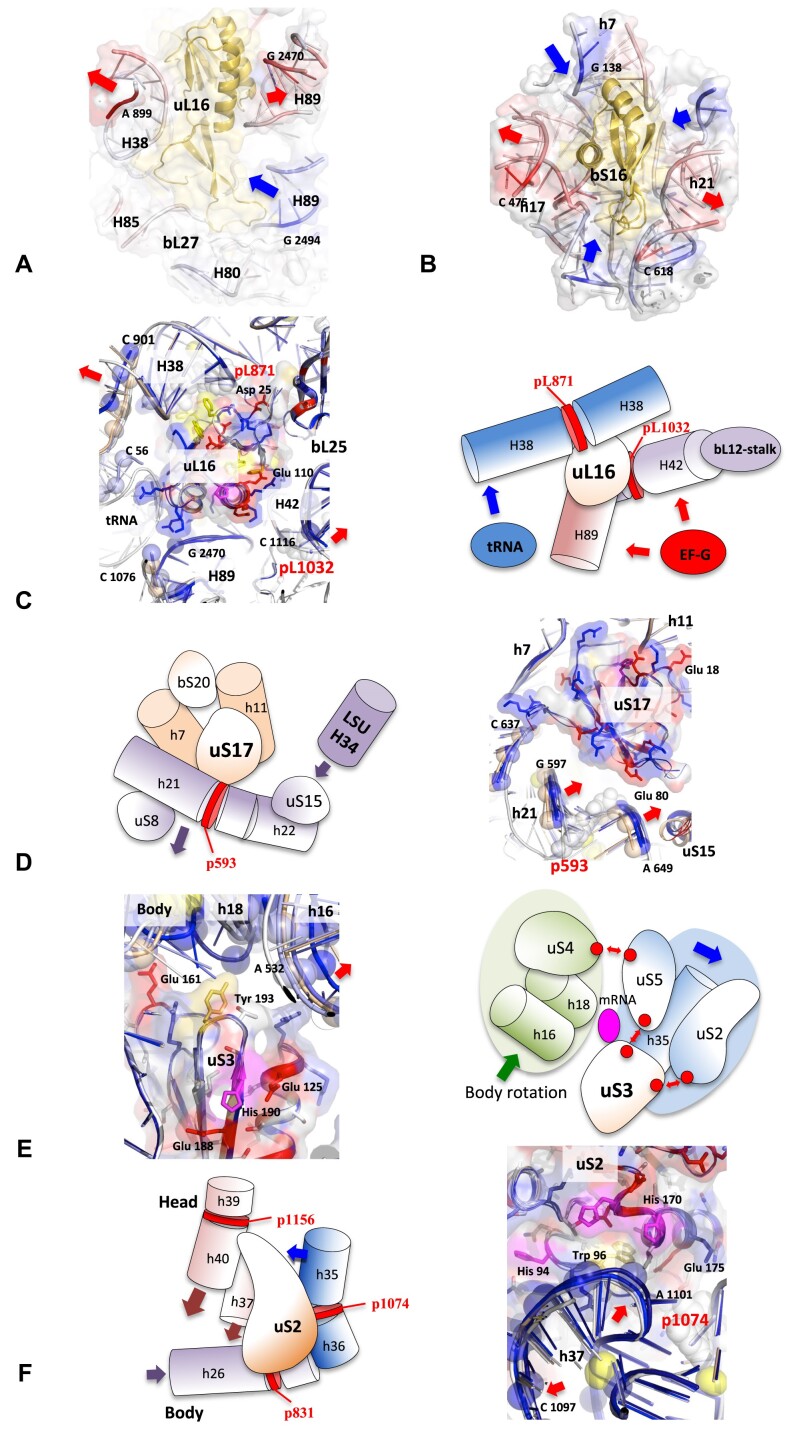
How does rRNA move around the r-proteins during translocation? RNA–protein distance approach cycles trapped at step 5 (7n1c) around uL16 (**A**) and bS16 (**B**). This is a graphic representation of the values reported in the tables of Supplementary Data 1. The r-proteins are represented as yellow cartoons surrounded by rRNA helices represented by ribbon and surfaces. The RNA is coloured according to whether it has undergone a shift towards (Max = blue) or away (Max = red) from the the Center of Mass of protein in step 5, in comparison to its initial position relative to the protein in step 1. Cartoons and schematic representation of the motions of the rRNA helices around representative r-proteins (**C**) uL16, rRNA motion induced by external factors: tRNA step 3, EF-G step 4. (**D**) uS17, the moving away of H21 from uS17 induced by LH34 (LSU). (**E**) uS3, uS3 and mRNA channel: A combination of rRNA–protein and protein–protein motions. (**F**) uS2, rRNA/r-protein motion away from the transit of external factors. White cartoons represent the structures of the r-proteins and its RNA layer at step 1 (7n1p). Light blue: step 2 (7n30); Slate: step 3 (7n2u); Blue: step 4 (7n2v); Deep blue: step 5 (7n2c) and Wheat: step 6 (7n31). The structures of the r-protein (at step 1) are represented by cartoons and surfaces. The phosphate groups of rRNA helices observed in steps 2–5 which have moved >1 Å from their position in step 1 are represented by spheres.

Visualizing and quantifying the rRNA mobility around r-proteins at different stages reveals a wide variety of rRNA dynamics around each r-protein. Two main types of movement are observed: local motions, affecting one or two nucleotides and global motions, when an entire rRNA helix moves towards or away from an r-protein. Overall, the majority of RNA–protein interactions in the ribosome undergo what might be compared to ‘breathing’, i.e. a ‘distance/approach cycle’ during translocation (Supplementary Data 1 and 2). During the course of the translocation process, rRNA helices approach and move away from the majority of r-proteins in a period that depends on specific areas of the ribosome. Figure 5A-B depict the movement of rRNA helices in respect to uL16 and bS16 at step 5 (EF-G bound after GTP hydrolysis). Three principal external factors influence the RNA/protein mobility during the process of translocation: the passage of EF-G, the movement of tRNAs and the relative motion of rRNA domains (Figure 5C–F, and Supplementary Text 3). Complex RNA tectonic patterns have been observed in the vicinity of r-proteins inserted within multiple rRNA helices. The uS2, uS3 and uS17 proteins are encircled by helices that move in opposing directions at various translocation steps. It is worth noting that while some r-proteins, such as uS3-uS10-uS14, remain anchored together in a rigid block throughout the translocation process, others, such as uS7-uS9 or uS3-uS5-uS2, undergo a rearrangement of their interaction surfaces. This contributes to significant reconfigurations of the ribosome's functional sites. It can therefore be concluded that the r-proteins are not simply anchored to rRNA and do not move passively, transported by the RNA helices to which they are anchored. Rather, they regulate the movements of rRNA or other proteins in their vicinity and are integral to the dynamics of the ribosome.

#### How does the rRNA/r-protein mobility influence the translocation?

**Figure 6.**
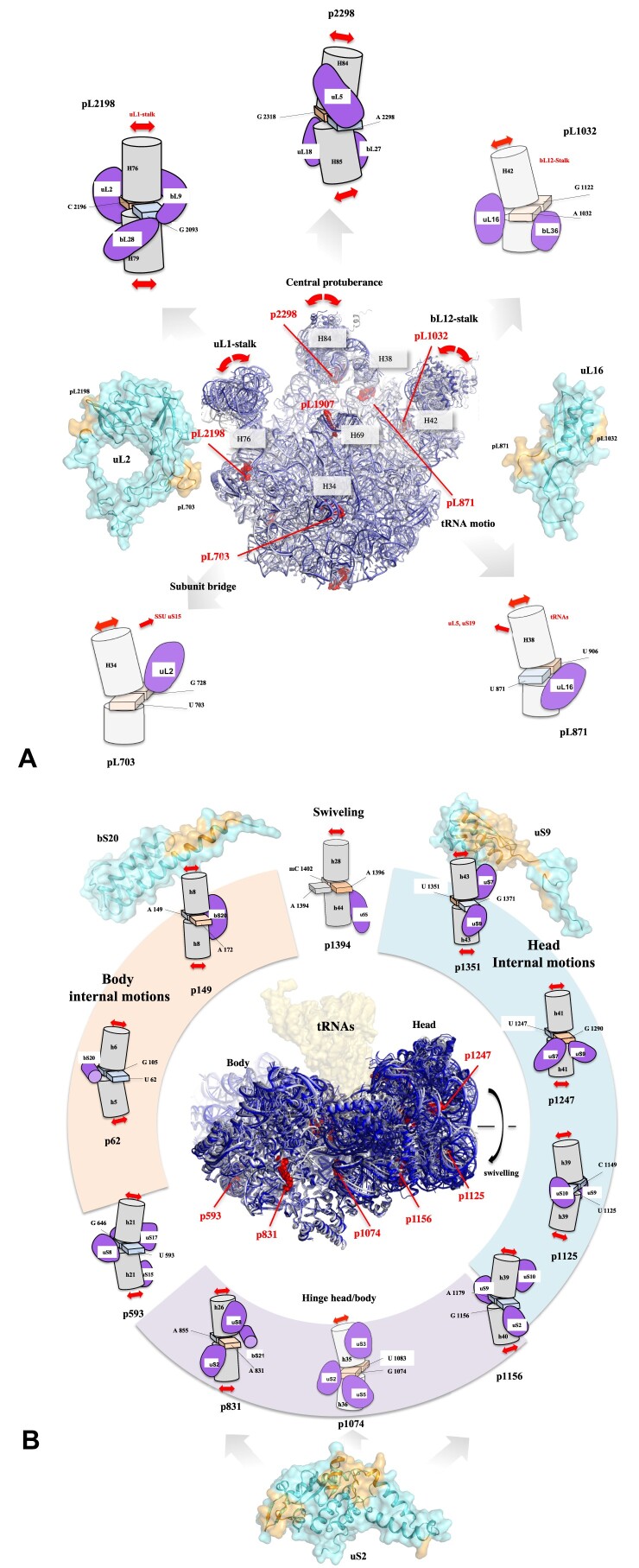
Global view of the CM–r-protein interactions in the context of the ribosome dynamics during translocation. The schematic figures represent the main CMs (represented as coloured squares) bound by the r-proteins and the corresponding motions during the translocation in the LSU (**A**) and the SSU (**B**). The r-proteins that mediate bridges between two or several CMs are represented by surfaces coloured in cyan. The interaction surfaces are represented in orange.

**Figure 7.**
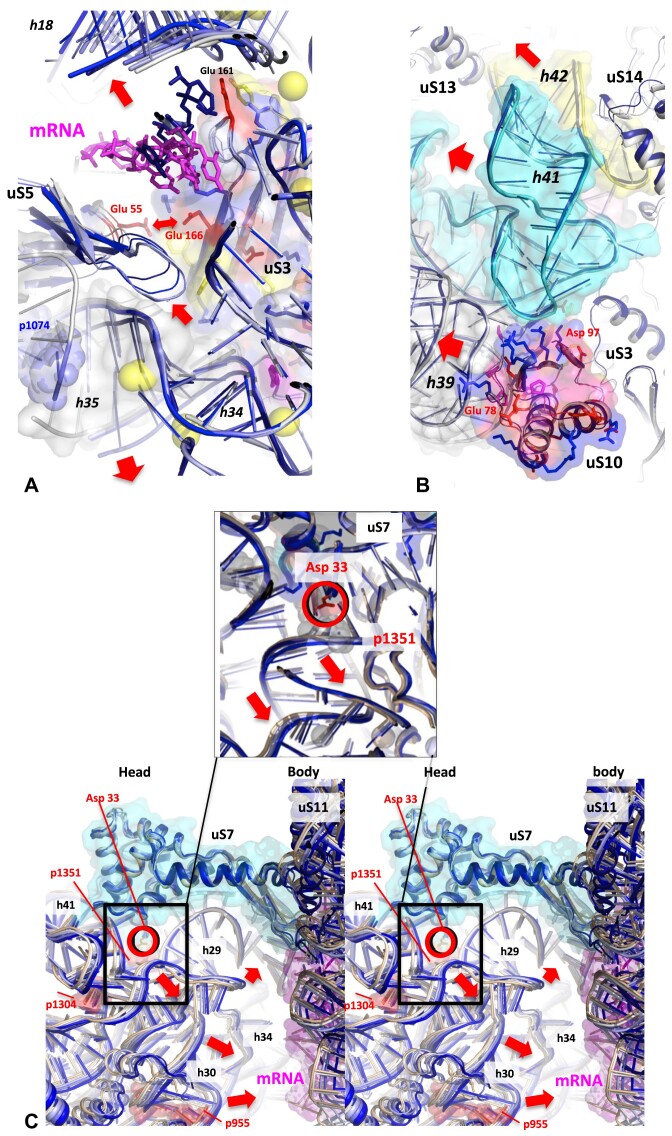
Influence of the local rRNA–protein mobility on the dynamics of the head during translocation. The sum of the localized rRNA–r-protein motions around each r-protein rearranges the internal structure of the head and modulates the sizes of the entry and exit mRNA channel in the head (A–C) during the head swivelling (occurring from steps 3–5). Motions of the r-proteins and rRNA helices around uS3 (**A**), uS10 (**B**) and uS7 (**C**) (each one is fixed during superimposition of the structures observed from steps 2–5). The red arrows highlight the motions during the translocation steps (same colour code as in Figure 1). See also Supplementary Figures S7 and S20–S23 for details and stereo views.

The extent to which RNA–protein mobility and the CM-protein network contribute to the overall dynamics is different in the two subunits. In the LSU, the r-proteins essentially play a punctual role in controlling the movement of stalks or key helices involved in the intersubunit bridges (H34) or in contacts with tRNA (H38) (Figure 6A, and Supplementary Figure S19 and Supplementary Text 3B). The dynamics of SSU are considerably more intricate, depending on an elaborate network of interactions (Figure 6B, and Supplementary Figure S20). The head and body do not function as rigid blocks, but rather undergo internal structural rearrangements during swivelling (Step 4–5) and back-swivelling (Step 6) (Supplementary Figure S7). The r-proteins exert a pivotal influence on these internal motions, orchestrating subtle control over their dynamics during translocation. They collectively control the mobility of the head/body junction, facilitate the head and body internal rearrangements and modulate the entry and exit size of the mRNA channel. The head rotates around the two hinges located at the head/body interface, p1394 and p1074, which are bound by uS5 and uS2 and uS5 respectively (Figure 6B). uS2, uS3 and uS5 play a key role in accommodating the rRNA tectonic around them (Figures 5E and F and Figure 7A, and Supplementary Figure S21A and B). The triad uS2-uS3-uS5 also exhibits relative motions with respect to each other, mainly due to repulsive interactions between key acidic residues at their interfaces (Supplementary Figure S21C–E). The movement of rRNA around uS7 has repercussions on the tRNA-E sites and the size of the exit tunnel (Figure 7C, and Supplementary Figure S22). The uS7-uS9 interface also undergoes reconfiguration during swivelling, thereby facilitating adaptation of the region to the newly established contact with the body.

**Figure 8.**
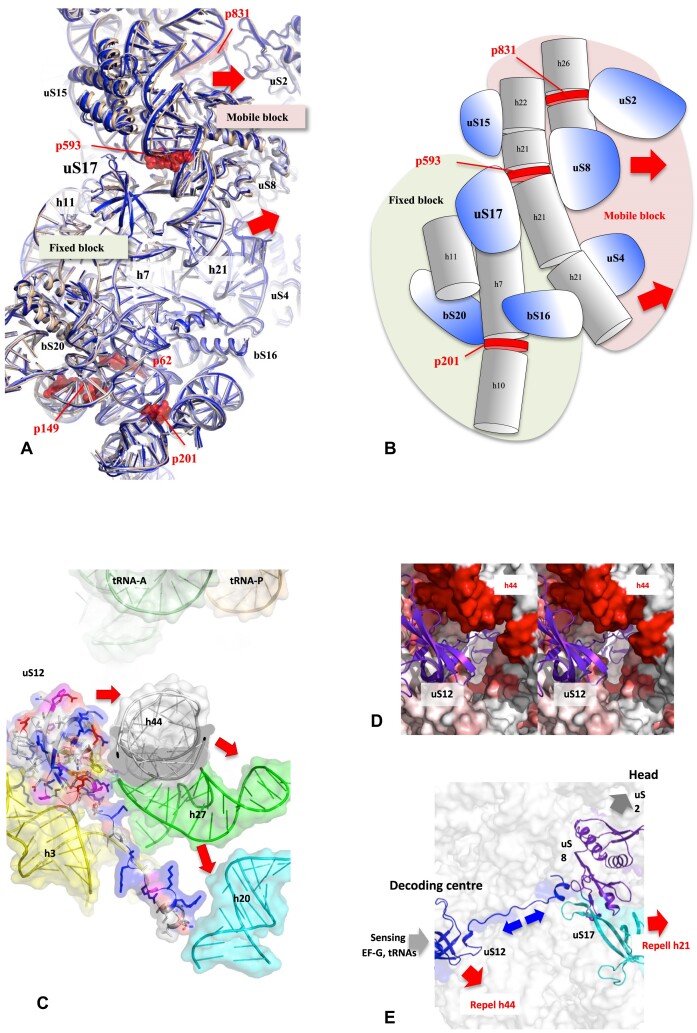
Influence of the local rRNA–protein mobility on the dynamics of the body during translocation. (**A**) The uS17 protein plays a pivotal role in orchestrating a significant reorganization of the body during the back-swivelling movement (step 6) in repelling h21 in front of p593. (**B**) The schematic view of the body represented in A illustrates the splitting of the body into mobile and moving blocks on both sides of uS17. (**C**) A further reorganization is initiated by uS12 on the opposite side of the body, resulting in the repulsion of h44 at step 6. (**D**) Stereo view focusing on uS12 in the SSU map (Figure 4, step 6). The 16S RNA surface around uS12 is coloured from white to red according to the phosphorus-phosphorus displacements at step 6. This view also shows how the long extension of uS12 passes right through the body to reach uS17 on the other side. (**E**) A model showing how uS12 and uS17 may be synchronized through a process of ‘electrostatic dominoes’ allostery (blue arrows), in their repulsion of the large helices h44 and h21 during back-swivelling.

Protein/RNA mobility also controls the body internal rearrangement during the back-swivelling (Step 6). The uS17 and uS12 proteins, situated on opposite sides of the SSU, play a crucial role in the process of fracturing the body into two blocs. This is achieved by controlling their distance from two large helices, h21 and h44, respectively (Figure 8A–C). Remarkably, the two connected r-proteins are synchronized in their repelling state at step 6 (Table 2, Figure 8D–E, and Supplementary Figures S15 and S23). This provides an interesting insight to understand how the network may coordinate rRNA/protein mobility at distance. It seems reasonable to postulate that a signal traversing the extension of uS12, which is itself sensitive to the electrostatic state of the decoding center, propagates towards uS17 and exerts an influence on the electrostatic potential of its surface in contact with h21. Overall, section demonstrates that the r-proteins are placed in strategic positions in the ribosome that allow modulating and orienting the interactions between large rRNA domains through electrostatic changes in their surfaces.

#### r-protein motifs associated with rRNA motions: modulated electrostatic lever arms

**Figure 9.**
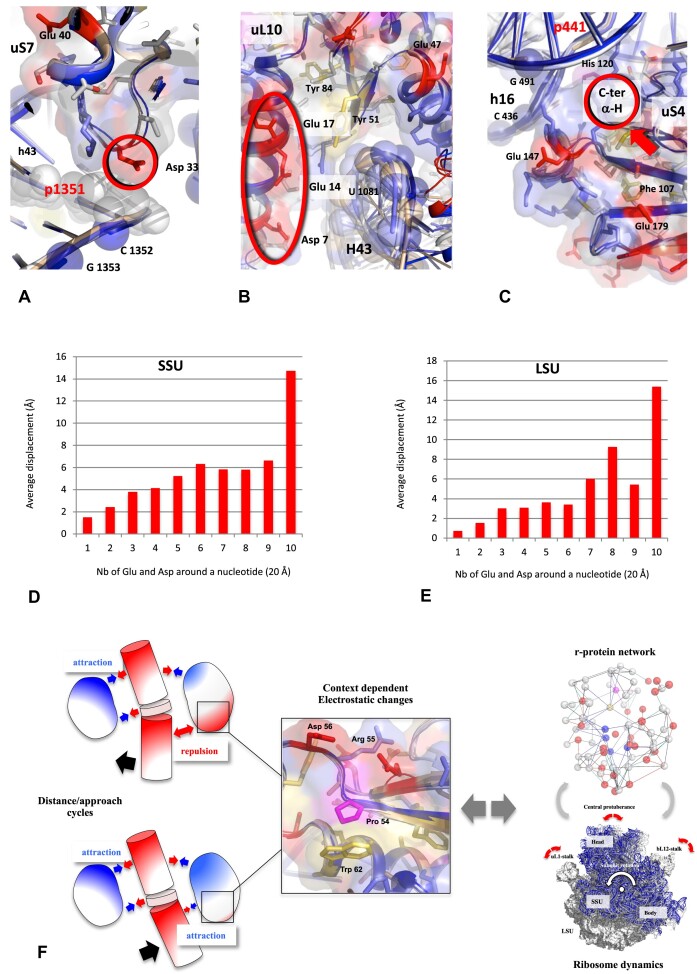
r-protein motifs observed at the vicinity of rRNA moving regions. (**A**) uS7-p1351: The conserved Asp 33 interact directly with the moving base pair of p1351. (**B**) uL10-H43 at the base of the bL12-stalk: Cluster of negatively charged residues is observed just in front of the highly moving H43 helix. (**C**) uS4-h16: The moving residues of the 16S are located in front of a C-ter extremity of an alpha helix of uS4. The structures of the r-proteins (at step 1) are represented by cartoons and surfaces, the phosphate groups which have moved more than 1 Å from their position in step 1 are represented by spheres. (D–E) Averages of displacement of a nucleotide (P-P distance) of rRNA in the SSU (**D**) and in the LSU (**E**) in function of the number of Glu and Asp around it. This is a graphic representation of Supplementary Table S5A and B and Supplementary Data 2. (**F**) A model of a ‘modulated electrostatic lever arm’. The context-induced electrostatic changes of the r-protein surfaces modulate the interaction with the rRNA and control the cycles of repulsion-attraction between rRNA and protein in function of electrostatic signals transmitted through the network.

The systematic survey of the rRNA/protein mobility around r-proteins reveals the r-protein motifs most often associated with rRNA mobility and those, on the contrary that are associated with immobile regions. In both SSU and LSU, the largest rRNA displacements are most often faced with negatively charged surface such as clusters of acidic residues or C-termini of α-helices (Figure 9A–E, and Supplementary Figure S24). This indicates that phosphate-repelling protein surfaces play a key role in the rRNA dynamics. These findings offer a logical rationale for the atypical electrostatic properties of r-protein motifs interacting with CMs (see part 1, 2) (Figure 2E and F, and Supplementary Figures S11, S12; Supplementary Text 3C) and fit well with previous studies showing that negative charges in nucleic acids (117–121) and protein destabilise, repel or control the nucleic acid dynamics (122–128). While basic grips or aromatic clamps immobilise rRNA strands, RNA/protein mobility occurs opposite acidic residues. It should be noted, however, that the RNA/protein mobility is cyclical and varies according to the translocation steps. This indicates that the affinity of r-proteins for their partners is subject to modulation by periodic fluctuations in their surface potentials. Accordingly, distance/approach cycles should be in alignment with repulsion/attraction cycles between the r-proteins and rRNA. We infer that subtle changes in the charge of the r-protein ionisable groups play a key role in this process.

A basis for this mechanism can be found in the existing literature. It is well established that the charges of proteins can vary according to their structural context (129–138). Furthermore, it has been demonstrated that these charges may be influenced by ribosome dynamics (139). The charges of Glu, Asp and Lys can be modulated by their electrostatic contexts (131–135). Furthermore, long range coupling between ligand binding and pKa changes supports the idea of the propagation of electrostatic changes at distant sites in the ribosome (136,137). Recent studies have shown indeed that transient electrostatic changes in proteins can modulate their binding affinities to DNA (140–142).

Integrating these findings supports an ‘electrostatic lever-arm’ model where r-proteins on one hand immobilise the hinge bases and on the other hand repel or attract of the rRNA on either side of the hinge (Figure 9F). The diversity of r-protein motifs found at the vicinity of CMs is conducive to various mechanisms controlling their context-dependent charge changes (Supplementary Figures S11, S12 and S24). Most of the Glu and Asp residues that interact with the mobile regions of the rRNA are located in ABA motifs of great diversity. They range from the simplest, when they are composed of a single acidic, basic and aromatic residue to the most complex as for example in uS2 or uL2 when they contain tryptophan, histidine or series of proline amino acids. These motifs could contribute to modulate the charge of the protein surface in function of the context. It seems plausible that electrostatic changes may be either transmitted through the network or directly triggered by rRNA motions around them.

## Discussion

During protein synthesis, ribosome dynamics regulate the movement of key macromolecules -including mRNA, translation factors, tRNAs and newly synthesized peptides- through its functional sites. This process involves a range of conformational changes, from local to large-scale, that ensure high selectivity and accurate translocation of substrates (Figure 1, and Supplementary Figures S1, S2 and Supplementary Text 1). The dynamics of translocation have been extensively studied both experimentally and theoretically (48,49), with studies converging on the concept of the ribosome as a ‘Brownian nanomachine’ powered by GTP, where rRNA plays a central role in its dynamics (15,16,22,51). It is hypothesized that the intrinsic dynamic properties of rRNA have have evolved through the distribution of CMs along its sequence, partitioning rRNA into flexible and more rigid domains (Supplementary Figures S3-S7). While these studies have provided valuable insights, two key questions remain: (i) how are signals transmitted between distant ribosomal functional sites, and what is their nature? (ii) How are ribosomal dynamics synchronized?

Among the various modes of communication between the functional sites of the ribosome (61–64) that involve RNA (65–71) or proteins (85,88–92,143) (Supplementary Text 2), —r-protein networks deserve special attention for several reasons (Figure 1, and Supplementary Figures S8, S9). First, the role of r-protein networks in translocation dynamics has not been thoroughly explored. Second, the minute yet remarkably well-conserved interactions, which are difficult to reconcile with a straightforward stabilizing function, strongly support the hypothesis that they play a role in allosteric communication (100). The increasing complexity of these networks, mirroring the evolving tasks of the ribosome, further supports their involvement in signaling (102). Third, a comprehensive comparison of these networks in ribosomes at different translation stages failed to identify conventional allosteric switches but proposed an alternative electrostatic mechanism for signal transmission, such as charge transfer between proteins (100). These transmission modes, initially described as electrostatic dominoes (144), were subsequently observed in the bL20 protein crystal structure (80,145) and then by molecular simulations in myosin and other systems (146,147). The protein–protein interfaces in r-protein networks are well suited to integrate a variety of allosteric mechanisms, including classical allostery (148–150), allostery without a conformational switch (151), charge transfer allostery (144,152,153) and even proton or electron transfer (140,154–162).

The present study offers new insights into the role of r-protein networks in coordinating ribosome dynamics. We first demonstrate that the major CMs in the bacterial ribosome are bound by r-proteins through highly conserved motifs, forming a dense network. Graph theory analysis reveals that this network architecture has evolved to optimize both information flow between functional centers and the specialized roles of each node in controlling ribosomal dynamics. Next, we reanalyze data from a previously published time-resolved cryo-electron microscopy study (48) to offer a protein-centric perspective on translocation dynamics. This reveals a universal ‘breathing’ or distance/approach cycles- in rRNA/r-protein and protein-protein interactions in the whole ribosome. Each r-protein plays a distinct role at specific stages, influencing both the surrounding rRNA and the overall ribosomal dynamics. While individual r-proteins affect localized elements of the LSU, together they contribute to the complex rearrangement of rRNA subdomains in the SSU. The cumulative effect of these localized actions influences the broader dynamics of translocation.

An ‘electrostatic lever arm’ mechanism could explain how the r-proteins modulate periodically their interactions with rRNA (Figure 9F). This hypothesis aligns with the concept of ‘electrostatic domino’ allostery, offering new insights into how the r-protein network transmits electrostatic ‘events’ between proteins, directly influencing rRNA mobility. A set of connected r-proteins (uS12, uS17 and uS8) in the SSU (Figure 8D and E, Supplementary Figure S15) provides initial clues on how the network could synchronize rRNA motions. Specifically, uS12 and uS17 coordinate the repulsion of large rRNA helices h44 and h21 during back-swivelling (step 6), driving the large-scale reorganization of the body.

Thus, among the multiple roles of r-proteins, one may be to help synchronize ribosomal dynamics. The diversity of motifs displayed on their surfaces enables r-proteins to provide the ribosome with a broader and more subtle repertoire of mechanisms for controlling rRNA dynamics. These findings could explain why the RNA world early on incorporated proteins during evolution. They also highlight the continuity between the role of r-proteins in ribosome assembly (97,163) and translocation dynamics. Of particular interest for understanding how r-proteins can integrate multiple signals from the network, are the synergistic allosteric systems involving multiple ligand-binding sites as seen in certain enzymes or kinases (103,164–167). The recent discovery of anionic G•U pairs (168) is particularly intriguing in this context, where negative charges play a critical role in the relative mobility of RNA with respect to proteins. It is possible that these charged G•U pairs also contribute to the regulation of ribosomal dynamics. Overall, this model offers a framework for understanding how evolution has shaped complex r-protein networks that could regulate ribosomal dynamics.

## Conclusion and perspectives

During translocation, r-proteins are not merely passive passengers on the rRNA helices to which they are bound. Instead, they could actively contribute to significant RNA–protein mobility that influences the overall ribosome dynamics. These findings represent a potential paradigm shift in our understanding of r-protein roles in translation and, when complemented by biochemical evidence, could further open new avenues for exploring how protein networks control the dynamics of large nucleic acid/protein assemblies, such as spliceosomes and chromatin. Several experimental approaches can be pursued to validate our hypotheses. We predict that mutations in the acidic residues near CMs will disrupt ribosome dynamics and synchronization. Mutations that disrupt network connectivity or r-protein interactions with CMs should also impair ribosome signaling and dynamics. Additionally, our study provides a novel conceptual framework for understanding ribosome heterogeneity (169–171). It can be hypothesized that changes in r-protein composition, and the resulting alterations in network connectivity, may regulate ribosome dynamics. These insights also offer potential applications in the design of antibiotics and the study of ribosomopathies (172). Despite the conserved motifs interacting with CMs, sequence variations between species suggest that specific charge distributions in r-proteins may lead to species-specific ribosome dynamics. Understanding these differences could help develop antibiotics targeting the ribosome dynamics of pathogenic species.

Finally, the concept of allostery, initially linked to the catalytic activity of enzymes or receptors, can be extended to the sensori-motor control of supramolecular assemblies. The analogy between networks operating at different scales—from supramolecular assemblies to organisms (103,173–175), —illustrates that the control of information flow in networks has, since the origin of life, been key to synchronizing dynamic processes.

## Supplementary Material

gkae1308_Supplemental_Files

## Data Availability

The data underlying this article are available in the article and in its online supplementary material.

## References

[1] Melnikov S., Ben-Shem A., Garreau de Loubresse N., Jenner L., Yusupova G., Yusupov M. One core, two shells: bacterial and eukaryotic ribosomes. Nat. Struct. Mol. Biol. 2012; 19:560–567. 22664983 10.1038/nsmb.2313

[2] Bashan A., Yonath A. Correlating ribosome function with high-resolution structures. Trends Microbiol. 2008; 16:326–335. 18547810 10.1016/j.tim.2008.05.001

[3] Steitz T.A. A structural understanding of the dynamic ribosome machine. Nat. Rev. Mol. Cell Biol. 2008; 9:242–253. 18292779 10.1038/nrm2352

[4] Schmeing T.M., Ramakrishnan V. What recent ribosome structures have revealed about the mechanism of translation. Nature. 2009; 461:1234–1242. 19838167 10.1038/nature08403

[5] Sheng K., Li N., Rabuck-Gibbons J.N., Dong X., Lyumkis D., Williamson J.R. Assembly landscape for the bacterial large ribosomal subunit. Nat. Commun. 2023; 14:5220. 37633970 10.1038/s41467-023-40859-w PMC10460392

[6] Rodgers M.L., Woodson S.A. A roadmap for rRNA folding and assembly during transcription. Trends Biochem. Sci. 2021; 46:889–901. 34176739 10.1016/j.tibs.2021.05.009 PMC8526401

[7] Peña C., Hurt E., Panse V.G. Eukaryotic ribosome assembly, transport and quality control. Nat. Struct. Mol. Biol. 2017; 24:689–699. 28880863 10.1038/nsmb.3454

[8] Wilson D.N., Arenz S., Beckmann R. Translation regulation via nascent polypeptide-mediated ribosome stalling. Curr. Opin. Struct. Biol. 2016; 37:123–133. 26859868 10.1016/j.sbi.2016.01.008

[9] Samatova E., Daberger J., Liutkute M., Rodnina M.V. Translational control by ribosome pausing in bacteria: how a non-uniform pace of translation affects protein production and folding. Front. Microbiol. 2021; 11:619430. 33505387 10.3389/fmicb.2020.619430 PMC7829197

[10] Kim K.Q., Zaher H.S. Canary in a coal mine: collided ribosomes as sensors of cellular conditions. Trends Biochem. Sci. 2022; 47:82–97. 34607755 10.1016/j.tibs.2021.09.001 PMC8688274

[11] Bose T., Fridkin G., Davidovich C., Krupkin M., Dinger N., Falkovich A.H., Peleg Y., Agmon I., Bashan A., Yonath A. Origin of life: protoribosome forms peptide bonds and links RNA and protein dominated worlds. Nucleic Acids Res. 2022; 50:1815–1828. 35137169 10.1093/nar/gkac052 PMC8886871

[12] Tanoz I., Timsit Y. Protein fold usages in ribosomes: another glance to the past. Int. J. Mol. Sci. 2024; 25:8806. 39201491 10.3390/ijms25168806 PMC11354259

[13] Grosjean H., Westhof E. An integrated, structure- and energy-based view of the genetic code. Nucleic Acids Res. 2016; 44:8020–8040. 27448410 10.1093/nar/gkw608 PMC5041475

[14] Tirumalai M.R., Rivas M., Tran Q., Fox G.E. The peptidyl transferase center: a window to the past. Microbiol. Mol. Biol. Rev. 2021; 85:e0010421. 34756086 10.1128/MMBR.00104-21 PMC8579967

[15] Frank J., Gonzalez R.L. Structure and dynamics of a processive brownian motor: the translating ribosome. Annu. Rev. Biochem. 2010; 79:381–412. 20235828 10.1146/annurev-biochem-060408-173330 PMC2917226

[16] Rodnina M.V., Wintermeyer W. Protein elongation, co-translational folding and targeting. J. Mol. Biol. 2016; 428:2165–2185. 27038507 10.1016/j.jmb.2016.03.022

[17] Korostelev A.A. The structural dynamics of translation. Annu. Rev. Biochem. 2022; 91:245–267. 35287473 10.1146/annurev-biochem-071921-122857 PMC10389292

[18] Belardinelli R., Sharma H., Caliskan N., Cunha C.E., Peske F., Wintermeyer W., Rodnina M.V. Choreography of molecular movements during ribosome progression along mRNA. Nat. Struct. Mol. Biol. 2016; 23:342–348. 26999556 10.1038/nsmb.3193

[19] Gulay S.P., Bista S., Varshney A., Kirmizialtin S., Sanbonmatsu K.Y., Dinman J.D. Tracking fluctuation hotspots on the yeast ribosome through the elongation cycle. Nucleic Acids Res. 2017; 45:4958–4971. 28334755 10.1093/nar/gkx112 PMC5416885

[20] Petrychenko V., Peng B.-Z., Schwarzer A.C.d.A.P., Peske F., Rodnina M.V., Fischer N. Structural mechanism of GTPase-powered ribosome-tRNA movement. Nat. Commun. 2021; 12:5933. 34635670 10.1038/s41467-021-26133-x PMC8505512

[21] Belardinelli R., Sharma H., Caliskan N., Cunha C.E., Peske F., Wintermeyer W., Rodnina M.V. Choreography of molecular movements during ribosome progression along mRNA. Nat. Struct. Mol. Biol. 2016; 23:342–348. 26999556 10.1038/nsmb.3193

[22] Jobe A., Liu Z., Gutierrez-Vargas C., Frank J. New insights into ribosome structure and function. Cold Spring Harb. Perspect. Biol. 2019; 11:a032615. 29903714 10.1101/cshperspect.a032615 PMC6314068

[23] Zimmermann M.T., Jia K., Jernigan R.L. Ribosome mechanics informs about mechanism. J. Mol. Biol. 2016; 428:802–810. 26687034 10.1016/j.jmb.2015.12.003 PMC4789072

[24] Berisio R., Schluenzen F., Harms J., Bashan A., Auerbach T., Baram D., Yonath A. Structural insight into the role of the ribosomal tunnel in cellular regulation. Nat. Struct. Biol. 2003; 10:366–370. 12665853 10.1038/nsb915

[25] Kurkcuoglu O., Kurkcuoglu Z., Doruker P., Jernigan R.L. Collective dynamics of the ribosomal tunnel revealed by elastic network modeling. Proteins. 2009; 75:837–845. 19004020 10.1002/prot.22292 PMC2774139

[26] Noller H.F., Lancaster L., Mohan S., Zhou J. Ribosome structural dynamics in translocation: yet another functional role for ribosomal RNA. Q. Rev. Biophys. 2017; 50:e12. 29233224 10.1017/S0033583517000117

[27] Adio S., Sharma H., Senyushkina T., Karki P., Maracci C., Wohlgemuth I., Holtkamp W., Peske F., Rodnina M.V. Dynamics of ribosomes and release factors during translation termination in E. coli. eLife. 2018; 7:e34252. 29889659 10.7554/eLife.34252 PMC5995542

[28] Sharma H., Adio S., Senyushkina T., Belardinelli R., Peske F., Rodnina M.V. Kinetics of spontaneous and EF-G-accelerated rotation of ribosomal subunits. Cell Rep. 2016; 16:2187–2196. 27524615 10.1016/j.celrep.2016.07.051

[29] Whitford P.C., Blanchard S.C., Cate J.H.D., Sanbonmatsu K.Y. Connecting the kinetics and energy landscape of tRNA translocation on the ribosome. PLoS Comput. Biol. 2013; 9:e1003003. 23555233 10.1371/journal.pcbi.1003003 PMC3605090

[30] Bock L.V., Kolář M.H., Grubmüller H. Molecular simulations of the ribosome and associated translation factors. Curr. Opin. Struct. Biol. 2018; 49:27–35. 29202442 10.1016/j.sbi.2017.11.003

[31] Bock L.V., Blau C., Vaiana A.C., Grubmüller H. Dynamic contact network between ribosomal subunits enables rapid large-scale rotation during spontaneous translocation. Nucleic Acids Res. 2015; 43:6747–6760. 26109353 10.1093/nar/gkv649 PMC4538834

[32] Bock L.V., Gabrielli S., Kolář M.H., Grubmüller H. Simulation of complex biomolecular systems: the ribosome challenge. Annu. Rev. Biophys. 2023; 52:361–390. 36719969 10.1146/annurev-biophys-111622-091147

[33] Loveland A.B., Demo G., Korostelev A.A. Cryo-EM of elongating ribosome with EF-Tu•GTP elucidates tRNA proofreading. Nature. 2020; 584:640–645. 32612237 10.1038/s41586-020-2447-x PMC7483604

[34] Rodnina M.V., Fischer N., Maracci C., Stark H. Ribosome dynamics during decoding. Philos. Trans. R. Soc. B Biol. Sci. 2017; 372:20160182. 10.1098/rstb.2016.0182 PMC5311926 28138068

[35] Schmeing T.M., Voorhees R.M., Kelley A.C., Gao Y.-G., Murphy F.V., Weir J.R., Ramakrishnan V. The crystal structure of the ribosome bound to EF-tu and aminoacyl-tRNA. Science. 2009; 326:688–694. 19833920 10.1126/science.1179700 PMC3763470

[36] Ogle J.M., Ramakrishnan V. Structural insights into translational fidelity. Annu. Rev. Biochem. 2005; 74:129–177. 15952884 10.1146/annurev.biochem.74.061903.155440

[37] Voorhees R.M., Ramakrishnan V. Structural basis of the translational elongation cycle*. Annu. Rev. Biochem. 2013; 82:203–236. 23746255 10.1146/annurev-biochem-113009-092313

[38] Valle M., Zavialov A., Sengupta J., Rawat U., Ehrenberg M., Frank J. Locking and unlocking of ribosomal motions. Cell. 2003; 114:123–134. 12859903 10.1016/s0092-8674(03)00476-8

[39] Schuwirth B.S., Borovinskaya M.A., Hau C.W., Zhang W., Vila-Sanjurjo A., Holton J.M., Cate J.H.D. Structures of the bacterial ribosome at 3.5 A resolution. Science. 2005; 310:827–834. 16272117 10.1126/science.1117230

[40] Noeske J., Cate J.H. Structural basis for protein synthesis: snapshots of the ribosome in motion. Curr. Opin. Struct. Biol. 2012; 22:743–749. 22871550 10.1016/j.sbi.2012.07.011 PMC3513557

[41] Wimberly B.T., Brodersen D.E., Clemons W.M., Morgan-Warren R.J., Carter A.P., Vonrhein C., Hartsch T., Ramakrishnan V. Structure of the 30S ribosomal subunit. Nature. 2000; 407:327–339. 11014182 10.1038/35030006

[42] Ling C., Ermolenko D.N. Structural insights into ribosome translocation. Wiley Interdiscip. Rev. RNA. 2016; 7:620–636. 27117863 10.1002/wrna.1354 PMC4990484

[43] Ratje A.H., Loerke J., Mikolajka A., Brünner M., Hildebrand P.W., Starosta A.L., Dönhöfer A., Connell S.R., Fucini P., Mielke T. et al. Head swivel on the ribosome facilitates translocation by means of intra-subunit tRNA hybrid sites. Nature. 2010; 468:713–716. 21124459 10.1038/nature09547 PMC3272701

[44] Frank J., Agrawal R.K. A ratchet-like inter-subunit reorganization of the ribosome during translocation. Nature. 2000; 406:318–322. 10917535 10.1038/35018597

[45] Horan L.H., Noller H.F. Intersubunit movement is required for ribosomal translocation. Proc. Natl Acad. Sci. U.S.A. 2007; 104:4881–4885. 17360328 10.1073/pnas.0700762104 PMC1829233

[46] Ermolenko D.N., Majumdar Z.K., Hickerson R.P., Spiegel P.C., Clegg R.M., Noller H.F. Observation of intersubunit movement of the ribosome in solution using FRET. J. Mol. Biol. 2007; 370:530–540. 17512008 10.1016/j.jmb.2007.04.042

[47] Hassan A., Byju S., Freitas F.C., Roc C., Pender N., Nguyen K., Kimbrough E.M., Mattingly J.M., Gonzalez R.L., de Oliveira R.J. et al. Ratchet, swivel, tilt and roll: a complete description of subunit rotation in the ribosome. Nucleic Acids Res. 2023; 51:919–934. 36583339 10.1093/nar/gkac1211 PMC9881166

[48] Rundlet E.J., Holm M., Schacherl M., Natchiar S.K., Altman R.B., Spahn C.M.T., Myasnikov A.G., Blanchard S.C. Structural basis of early translocation events on the ribosome. Nature. 2021; 595:741–745. 34234344 10.1038/s41586-021-03713-x PMC8318882

[49] Carbone C.E., Loveland A.B., Gamper H.B., Hou Y.-M., Demo G., Korostelev A.A. Time-resolved cryo-EM visualizes ribosomal translocation with EF-G and GTP. Nat. Commun. 2021; 12:7236. 34903725 10.1038/s41467-021-27415-0 PMC8668904

[50] Spirin A.S. The ribosome as an RNA-based molecular machine. RNA Biol. 2004; 1:2–8. 17194938

[51] Dashti A., Schwander P., Langlois R., Fung R., Li W., Hosseinizadeh A., Liao H.Y., Pallesen J., Sharma G., Stupina V.A. et al. Trajectories of the ribosome as a Brownian nanomachine. Proc. Natl Acad. Sci. U.S.A. 2014; 111:17492–17497. 25422471 10.1073/pnas.1419276111 PMC4267381

[52] Paci M., Fox G.E. Major centers of motion in the large ribosomal RNAs. Nucleic Acids Res. 2015; 43:4640–4649. 25870411 10.1093/nar/gkv289 PMC4482067

[53] Paci M., Fox G.E. Centers of motion associated with EF-Tu binding to the ribosome. RNA Biol. 2016; 13:524–530. 26786136 10.1080/15476286.2015.1114204 PMC4962796

[54] Mohan S., Donohue J.P., Noller H.F. Molecular mechanics of 30S subunit head rotation. Proc. Natl Acad. Sci. U.S.A. 2014; 111:13325–13330. 25187561 10.1073/pnas.1413731111 PMC4169957

[55] Mohan S., Noller H.F. Recurring RNA structural motifs underlie the mechanics of L1 stalk movement. Nat. Commun. 2017; 8:14285. 28176782 10.1038/ncomms14285 PMC5309774

[56] Varani G., McClain W.H. The G x U wobble base pair. A fundamental building block of RNA structure crucial to RNA function in diverse biological systems. EMBO Rep. 2000; 1:18–23. 11256617 10.1093/embo-reports/kvd001 PMC1083677

[57] Masquida B., Westhof E. On the wobble GoU and related pairs. RNA. 2000; 6:9–15. 10668794 10.1017/s1355838200992082 PMC1369889

[58] Westhof E., Yusupov M., Yusupova G. The multiple flavors of GoU pairs in RNA. J. Mol. Recognit. 2019; 32:e2782. 31033092 10.1002/jmr.2782 PMC6617799

[59] Lescoute A., Westhof E. Topology of three-way junctions in folded RNAs. RNA. 2006; 12:83–93. 16373494 10.1261/rna.2208106 PMC1370888

[60] Réblová K., Sponer J., Lankas F. Structure and mechanical properties of the ribosomal L1 stalk three-way junction. Nucleic Acids Res. 2012; 40:6290–6303. 22451682 10.1093/nar/gks258 PMC3401443

[61] Chen C., Stevens B., Kaur J., Smilansky Z., Cooperman B.S., Goldman Y.E. Allosteric vs. spontaneous exit-site (E-site) tRNA dissociation early in protein synthesis. Proc. Natl Acad. Sci. U.S.A. 2011; 108:16980–16985. 21969541 10.1073/pnas.1106999108 PMC3193197

[62] Fei J., Bronson J.E., Hofman J.M., Srinivas R.L., Wiggins C.H., Gonzalez R.L. Allosteric collaboration between elongation factor G and the ribosomal L1 stalk directs tRNA movements during translation. Proc. Natl Acad. Sci. U.S.A. 2009; 106:15702–15707. 19717422 10.1073/pnas.0908077106 PMC2747183

[63] Fei J., Kosuri P., MacDougall D.D., Gonzalez R.L. Coupling of ribosomal L1 stalk and tRNA dynamics during translation elongation. Mol. Cell. 2008; 30:348–359. 18471980 10.1016/j.molcel.2008.03.012

[64] Sengupta A., Rice G.M., Weeks K.M. Single-molecule correlated chemical probing reveals large-scale structural communication in the ribosome and the mechanism of the antibiotic spectinomycin in living cells. PLoS Biol. 2019; 17:e3000393. 31487286 10.1371/journal.pbio.3000393 PMC6748448

[65] Walker A.S., Russ W.P., Ranganathan R., Schepartz A. RNA sectors and allosteric function within the ribosome. Proc. Natl Acad. Sci. U.S.A. 2020; 117:19879–19887. 32747536 10.1073/pnas.1909634117 PMC7443888

[66] Guzel P., Kurkcuoglu O. Identification of potential allosteric communication pathways between functional sites of the bacterial ribosome by graph and elastic network models. Biochim. Biophys. Acta Gen. Subj. 2017; 1861:3131–3141. 28917952 10.1016/j.bbagen.2017.09.005

[67] Rakauskaite R., Dinman J.D. rRNA mutants in the yeast peptidyltransferase center reveal allosteric information networks and mechanisms of drug resistance. Nucleic Acids Res. 2008; 36:1497–1507. 18203742 10.1093/nar/gkm1179 PMC2275155

[68] Rakauskaite R., Dinman J.D. An arc of unpaired ‘hinge bases’ facilitates information exchange among functional centers of the ribosome. Mol. Cell. Biol. 2006; 26:8992–9002. 17000775 10.1128/MCB.01311-06 PMC1636827

[69] Makarov G.I., Golovin A.V., Sumbatyan N.V., Bogdanov A.A. Molecular dynamics investigation of a mechanism of allosteric signal transmission in ribosomes. Biochem. Biokhimiia. 2015; 80:1047–1056. 10.1134/S0006297915080106 26547073

[70] Guzel P., Yildirim H.Z., Yuce M., Kurkcuoglu O. exploring allosteric signaling in the exit tunnel of the bacterial ribosome by molecular dynamics simulations and residue network model. Front. Mol. Biosci. 2020; 7:586075. 33102529 10.3389/fmolb.2020.586075 PMC7545307

[71] Chan Y.-L., Dresios J., Wool I.G. A pathway for the transmission of allosteric signals in the ribosome through a network of RNA tertiary interactions. J. Mol. Biol. 2006; 355:1014–1025. 16359709 10.1016/j.jmb.2005.11.037

[72] Lu J., Deutsch C. Regional discrimination and propagation of local rearrangements along the ribosomal exit tunnel. J. Mol. Biol. 2014; 426:4061–4073. 25308341 10.1016/j.jmb.2014.10.006 PMC4979075

[73] Arenz S., Meydan S., Starosta A.L., Berninghausen O., Beckmann R., Vázquez-Laslop N., Wilson D.N. Drug sensing by the ribosome induces translational arrest via active site perturbation. Mol. Cell. 2014; 56:446–452. 25306253 10.1016/j.molcel.2014.09.014 PMC4252717

[74] O’Connor M., Gregory S.T., Dahlberg A.E. Multiple defects in translation associated with altered ribosomal protein L4. Nucleic Acids Res. 2004; 32:5750–5756. 15509870 10.1093/nar/gkh913 PMC528798

[75] Denks K., Sliwinski N., Erichsen V., Borodkina B., Origi A., Koch H.-G. The signal recognition particle contacts uL23 and scans substrate translation inside the ribosomal tunnel. Nat. Microbiol. 2017; 2:16265. 28134917 10.1038/nmicrobiol.2016.265

[76] Brodersen D.E., Clemons W.M., Carter A.P., Wimberly B.T., Ramakrishnan V. Crystal structure of the 30 s ribosomal subunit from *Thermus thermophilus*: structure of the proteins and their interactions with 16 s RNA1. J. Mol. Biol. 2002; 316:725–768. 11866529 10.1006/jmbi.2001.5359

[77] Klein D.J., Moore P.B., Steitz T.A. The roles of ribosomal proteins in the structure assembly, and evolution of the large ribosomal subunit. J. Mol. Biol. 2004; 340:141–177. 15184028 10.1016/j.jmb.2004.03.076

[78] Alva V., Söding J., Lupas A.N. A vocabulary of ancient peptides at the origin of folded proteins. eLife. 2015; 4:e09410. 26653858 10.7554/eLife.09410 PMC4739770

[79] Wilson D.N., Nierhaus K.H. Ribosomal proteins in the spotlight. Crit. Rev. Biochem. Mol. Biol. 2005; 40:243–267. 16257826 10.1080/10409230500256523

[80] Timsit Y., Acosta Z., Allemand F., Chiaruttini C., Springer M. The role of disordered ribosomal protein extensions in the early steps of eubacterial 50 S ribosomal subunit assembly. Int. J. Mol. Sci. 2009; 10:817–834. 19399222 10.3390/ijms10030817 PMC2672003

[81] Calidas D., Lyon H., Culver G.M. The N-terminal extension of S12 influences small ribosomal subunit assembly in *Escherichia coli*. RNA. 2014; 20:321–330. 24442609 10.1261/rna.042432.113 PMC3923127

[82] Lawrence M.G., Shamsuzzaman M., Kondopaka M., Pascual C., Zengel J.M., Lindahl L. The extended loops of ribosomal proteins uL4 and uL22 of *Escherichia coli* contribute to ribosome assembly and protein translation. Nucleic Acids Res. 2016; 44:5798–5810. 27257065 10.1093/nar/gkw493 PMC4937340

[83] Gamalinda M., Woolford J.L. Paradigms of ribosome synthesis: lessons learned from ribosomal proteins. Transl. Austin Tex. 2015; 3:e975018. 10.4161/21690731.2014.975018 PMC4682805 26779413

[84] Sulima S.O., Gülay S.P., Anjos M., Patchett S., Meskauskas A., Johnson A.W., Dinman J.D. Eukaryotic rpL10 drives ribosomal rotation. Nucleic Acids Res. 2014; 42:2049–2063. 24214990 10.1093/nar/gkt1107 PMC3919601

[85] Bowen A.M., Musalgaonkar S., Moomau C.A., Gulay S.P., Mirvis M., Dinman J.D. Ribosomal protein uS19 mutants reveal its role in coordinating ribosome structure and function. Translation. 2015; 3:e1117703. 26824029 10.1080/21690731.2015.1117703 PMC4721500

[86] Rhodin M.H.J., Dinman J.D. An extensive network of information flow through the B1b/c intersubunit bridge of the yeast ribosome. PLoS One. 2011; 6:e20048. 21625514 10.1371/journal.pone.0020048 PMC3098278

[87] Jindal S., Ghosh A., Ismail A., Singh N., Komar A.A. Role of the uS9/yS16 C-terminal tail in translation initiation and elongation in *Saccharomyces cerevisiae*. Nucleic Acids Res. 2019; 47:806–823. 30481328 10.1093/nar/gky1180 PMC6344880

[88] Mailliot J., Garreau de Loubresse N., Yusupova G., Meskauskas A., Dinman J.D., Yusupov M. Crystal structures of the uL3 mutant ribosome: illustration of the importance of ribosomal proteins for translation efficiency. J. Mol. Biol. 2016; 428:2195–2202. 26906928 10.1016/j.jmb.2016.02.013 PMC5331904

[89] Rhodin M.H.J., Dinman J.D. A flexible loop in yeast ribosomal protein L11 coordinates P-site tRNA binding. Nucleic Acids Res. 2010; 38:8377–8389. 20705654 10.1093/nar/gkq711 PMC3001080

[90] Meskauskas A., Dinman J.D. A molecular clamp ensures allosteric coordination of peptidyltransfer and ligand binding to the ribosomal A-site. Nucleic Acids Res. 2010; 38:7800–7813. 20660012 10.1093/nar/gkq641 PMC2995063

[91] Singh N., Jindal S., Ghosh A., Komar A.A. Communication between RACK1/Asc1 and uS3 (Rps3) is essential for RACK1/Asc1 function in yeast *Saccharomyces cerevisiae*. Gene. 2019; 706:69–76. 31054365 10.1016/j.gene.2019.04.087 PMC6538471

[92] Meskauskas A., Russ J.R., Dinman J.D. Structure/function analysis of yeast ribosomal protein L2. Nucleic Acids Res. 2008; 36:1826–1835. 18263608 10.1093/nar/gkn034 PMC2330241

[93] Cukras A.R., Green R. Multiple effects of S13 in modulating the strength of intersubunit interactions in the ribosome during translation. J. Mol. Biol. 2005; 349:47–59. 15876367 10.1016/j.jmb.2005.03.075 PMC1687178

[94] Takyar S., Hickerson R.P., Noller H.F. mRNA helicase activity of the ribosome. Cell. 2005; 120:49–58. 15652481 10.1016/j.cell.2004.11.042

[95] Bock L.V., Blau C., Schröder G.F., Davydov I.I., Fischer N., Stark H., Rodnina M.V., Vaiana A.C., Grubmüller H. Energy barriers and driving forces in tRNA translocation through the ribosome. Nat. Struct. Mol. Biol. 2013; 20:1390–1396. 24186064 10.1038/nsmb.2690

[96] Demirci H., Wang L., Murphy F.V., Murphy E.L., Carr J.F., Blanchard S.C., Jogl G., Dahlberg A.E., Gregory S.T. The central role of protein S12 in organizing the structure of the decoding site of the ribosome. RNA. 2013; 19:1791–1801. 24152548 10.1261/rna.040030.113 PMC3884664

[97] Kim H., Abeysirigunawarden S.C., Chen K., Mayerle M., Ragunathan K., Luthey-Schulten Z., Ha T., Woodson S.A. Protein-guided RNA dynamics during early ribosome assembly. Nature. 2014; 506:334–338. 24522531 10.1038/nature13039 PMC3968076

[98] Lescoute A., Westhof E. The interaction networks of structured RNAs. Nucleic Acids Res. 2006; 34:6587–6604. 17135184 10.1093/nar/gkl963 PMC1747187

[99] David-Eden H., Mandel-Gutfreund Y. Revealing unique properties of the ribosome using a network based analysis. Nucleic Acids Res. 2008; 36:4641–4652. 18625614 10.1093/nar/gkn433 PMC2504294

[100] Poirot O., Timsit Y. Neuron-like networks between ribosomal proteins within the ribosome. Sci. Rep. 2016; 6:26485. 27225526 10.1038/srep26485 PMC4881015

[101] Timsit Y., Bennequin D. Nervous-like circuits in the ribosome facts, hypotheses and perspectives. Int. J. Mol. Sci. 2019; 20:2911. 31207893 10.3390/ijms20122911 PMC6627100

[102] Timsit Y., Sergeant-Perthuis G., Bennequin D. Evolution of ribosomal protein network architectures. Sci. Rep. 2021; 11:625. 33436806 10.1038/s41598-020-80194-4 PMC7804294

[103] Timsit Y., Grégoire S.-P. Towards the idea of molecular brains. Int. J. Mol. Sci. 2021; 22:11868. 34769300 10.3390/ijms222111868 PMC8584932

[104] Yutin N., Puigbò P., Koonin E.V., Wolf Y.I. Phylogenomics of prokaryotic ribosomal proteins. PLoS One. 2012; 7:e36972. 22615861 10.1371/journal.pone.0036972 PMC3353972

[105] Ban N., Beckmann R., Cate J.H.D., Dinman J.D., Dragon F., Ellis S.R., Lafontaine D.L.J., Lindahl L., Liljas A., Lipton J.M. et al. A new system for naming ribosomal proteins. Curr. Opin. Struct. Biol. 2014; 24:165–169. 24524803 10.1016/j.sbi.2014.01.002 PMC4358319

[106] Schrodinger L.L.C. The PyMOL Molecular Graphics System, Version 2.0. 2017; https://www.pymol.org/.

[107] Hagberg A., Swart P.J., Schult D.A. Exploring network structure, dynamics, and function using NetworkX. 2008; Los Alamos, NM, USA Los Alamos National Laboratory (LANL).

[108] Shazman S., Mandel-Gutfreund Y. Classifying RNA-binding proteins based on electrostatic properties. PLOS Comput. Biol. 2008; 4:e1000146. 18716674 10.1371/journal.pcbi.1000146 PMC2518515

[109] Lunde B.M., Moore C., Varani G. RNA-binding proteins: modular design for efficient function. Nat. Rev. Mol. Cell Biol. 2007; 8:479–490. 17473849 10.1038/nrm2178 PMC5507177

[110] Schottel B.L., Chifotides H.T., Dunbar K.R. Anion–π interactions. Chem. Soc. Rev. 2007; 37:68–83. 18197334 10.1039/b614208g

[111] Frontera A., Gamez P., Mascal M., Mooibroek T.J., Reedijk J. Putting anion–π interactions into perspective. Angew. Chem. Int. Ed. 2011; 50:9564–9583. 10.1002/anie.201100208 21928463

[112] Dougherty D.A. The cation–π interaction. Acc. Chem. Res. 2013; 46:885–893. 23214924 10.1021/ar300265y PMC3957424

[113] Mason O., Verwoerd M. Graph theory and networks in biology. IET Syst. Biol. 2007; 1:89–119. 17441552 10.1049/iet-syb:20060038

[114] Fletcher J.M., Wennekers T. From structure to activity: using centrality measures to predict neuronal activity. Int. J. Neural Syst. 2018; 28:1750013. 28076982 10.1142/S0129065717500137

[115] Bonacich P. Factoring and weighting approaches to status scores and clique identification. J. Math. Sociol. 1972; 2:113–120.

[116] Polacek N., Mankin A.S. The ribosomal peptidyl transferase center: structure, function, evolution, inhibition. Crit. Rev. Biochem. Mol. Biol. 2005; 40:285–311. 16257828 10.1080/10409230500326334

[117] Timsit Y., Moras D. Self-fitting and self-modifying properties of the B-DNA molecule. J. Mol. Biol. 1995; 251:629–647. 7666416 10.1006/jmbi.1995.0461

[118] Timsit Y. DNA-directed base pair opening. Mol. Basel Switz. 2012; 17:11947–11964. 10.3390/molecules171011947 PMC6268293 23060287

[119] Timsit Y., Westhof E., Fuchs R.P.P., Moras D. Unusual helical packing in crystals of DNA bearing a mutation hot spot. Nature. 1989; 341:459–462. 2797169 10.1038/341459a0

[120] Timsit Y., Moras D. Cruciform structures and functions. Q. Rev. Biophys. 1996; 29:279–307. 9080546 10.1017/s0033583500005862

[121] Várnai P., Timsit Y. Differential stability of DNA crossovers in solution mediated by divalent cations. Nucleic Acids Res. 2010; 38:4163–4172. 20215439 10.1093/nar/gkq150 PMC2896531

[122] Song J., Rechkoblit O., Bestor T.H., Patel D.J. Structure of DNMT1–DNA complex reveals a role for autoinhibition in maintenance DNA methylation. Science. 2011; 331:1036–1040. 21163962 10.1126/science.1195380 PMC4689315

[123] Jeltsch A., Jurkowska R.Z. Allosteric control of mammalian DNA methyltransferases – a new regulatory paradigm. Nucleic Acids Res. 2016; 44:8556–8575. 27521372 10.1093/nar/gkw723 PMC5062992

[124] Panja S., Santiago-Frangos A., Schu D.J., Gottesman S., Woodson S.A. Acidic residues in the Hfq chaperone increase the selectivity of sRNA binding and annealing. J. Mol. Biol. 2015; 427:3491–3500. 26196441 10.1016/j.jmb.2015.07.010 PMC4624489

[125] Hossain K.A., Kogut M., Słabońska J., Sappati S., Wieczór M., Czub J. How acidic amino acid residues facilitate DNA target site selection. Proc. Natl Acad. Sci. U.S.A. 2023; 120:e2212501120. 36634135 10.1073/pnas.2212501120 PMC9934023

[126] Chou C.-C., Wang A.H.-J. Structural D/E-rich repeats play multiple roles especially in gene regulation through DNA/RNA mimicry. Mol. Biosyst. 2015; 11:2144–2151. 26088262 10.1039/c5mb00206k

[127] Zaharias S., Zhang Z., Davis K., Fargason T., Cashman D., Yu T., Zhang J. Intrinsically disordered electronegative clusters improve stability and binding specificity of RNA-binding proteins. J. Biol. Chem. 2021; 297:100945. 34246632 10.1016/j.jbc.2021.100945 PMC8348266

[128] Rollins M.G., Shasmal M., Meade N., Astar H., Shen P.S., Walsh D. Negative charge in the RACK1 loop broadens the translational capacity of the human ribosome. Cell Rep. 2021; 36:109663. 34496247 10.1016/j.celrep.2021.109663 PMC8451006

[129] Liao S.-M., Du Q.-S., Meng J.-Z., Pang Z.-W., Huang R.-B. The multiple roles of histidine in protein interactions. Chem. Cent. J. 2013; 7:44. 23452343 10.1186/1752-153X-7-44 PMC3599372

[130] Baran K.L., Chimenti M.S., Schlessman J.L., Fitch C.A., Herbst K.J., Garcia-Moreno B.E. Electrostatic effects in a network of polar and ionizable groups in staphylococcal nuclease. J. Mol. Biol. 2008; 379:1045–1062. 18499123 10.1016/j.jmb.2008.04.021

[131] Di Russo N.V., Estrin D.A., Martí M.A., Roitberg A.E. pH-dependent conformational changes in proteins and their effect on experimental pK(a)s: the case of nitrophorin 4. PLoS Comput. Biol. 2012; 8:e1002761. 23133364 10.1371/journal.pcbi.1002761 PMC3486867

[132] Zhang H., Eerland J., Horn V., Schellevis R., van Ingen H. Mapping the electrostatic potential of the nucleosome acidic patch. Sci. Rep. 2021; 11:23013. 34837025 10.1038/s41598-021-02436-3 PMC8626509

[133] Karp D.A., Stahley M.R., García-Moreno B. Conformational consequences of ionization of Lys, Asp, and Glu buried at position 66 in staphylococcal nuclease. Biochemistry. 2010; 49:4138–4146. 20329780 10.1021/bi902114m PMC3373020

[134] Fitch C.A., Karp D.A., Lee K.K., Stites W.E., Lattman E.E., García-Moreno E.B. Experimental pK(a) values of buried residues: analysis with continuum methods and role of water penetration. Biophys. J. 2002; 82:3289–3304. 12023252 10.1016/s0006-3495(02)75670-1 PMC1302117

[135] Isom D.G., Castañeda C.A., Cannon B.R., García-Moreno B. Large shifts in pKa values of lysine residues buried inside a protein. Proc. Natl Acad. Sci. U.S.A. 2011; 108:5260–5265. 21389271 10.1073/pnas.1010750108 PMC3069169

[136] Bas D.C., Rogers D.M., Jensen J.H. Very fast prediction and rationalization of pKa values for protein–ligand complexes. Proteins. 2008; 73:765–783. 18498103 10.1002/prot.22102

[137] Aguilar B., Anandakrishnan R., Ruscio J.Z., Onufriev A.V. Statistics and physical origins of pK and ionization state changes upon protein–ligand binding. Biophys. J. 2010; 98:872–880. 20197041 10.1016/j.bpj.2009.11.016 PMC2830434

[138] Cruz-Gallardo I., Del Conte R., Velázquez-Campoy A., García-Mauriño S.M., Díaz-Moreno I. A non-invasive NMR method based on histidine imidazoles to analyze the pH-modulation of protein–nucleic acid interfaces. Chemistry. 2015; 21:7588–7595. 25846236 10.1002/chem.201405538

[139] Trylska J., Konecny R., Tama F., Brooks C.L., McCammon J.A. Ribosome motions modulate electrostatic properties. Biopolymers. 2004; 74:423–431. 15274086 10.1002/bip.20093

[140] Crack J.C., Amara P., Volbeda A., Mouesca J.-M., Rohac R., Pellicer Martinez M.T., Huang C.-Y., Gigarel O., Rinaldi C., Le Brun N.E. et al. Electron and proton transfers modulate DNA binding by the transcription regulator RsrR. J. Am. Chem. Soc. 2020; 142:5104–5116. 32078310 10.1021/jacs.9b12250

[141] Deochand D.K., Meariman J.K., Grove A. pH-dependent DNA distortion and repression of gene expression by pectobacterium atrosepticum PecS. ACS Chem. Biol. 2016; 11:2049–2056. 27213700 10.1021/acschembio.6b00168

[142] Deochand D.K., Pande A., Meariman J.K., Grove A. Redox sensing by PecS from the plant pathogen pectobacterium atrosepticum and its effect on gene expression and the conformation of PecS-bound promoter DNA. Biochemistry. 2019; 58:2564–2575. 31046241 10.1021/acs.biochem.9b00288

[143] Arora S., Bhamidimarri S.P., Bhattacharyya M., Govindan A., Weber M.H.W., Vishveshwara S., Varshney U. Distinctive contributions of the ribosomal P-site elements m 2 G966, m 5 C967 and the C-terminal tail of the S9 protein in the fidelity of initiation of translation in *Escherichia coli*. Nucleic Acids Res. 2013; 41:4963–4975. 23530111 10.1093/nar/gkt175 PMC3643588

[144] Sebban P., Maróti P., Schiffer M., Hanson D.K. Electrostatic dominoes: long distance propagation of mutational effects in photosynthetic reaction centers of *Rhodobacter capsulatus*. Biochemistry. 1995; 34:8390–8397. 7599129 10.1021/bi00026a021

[145] Timsit Y., Allemand F., Chiaruttini C., Springer M. Coexistence of two protein folding states in the crystal structure of ribosomal protein L20. EMBO Rep. 2006; 7:1013–1018. 16977336 10.1038/sj.embor.7400803 PMC1618378

[146] Sato T., Ohnuki J., Takano M. Long-range coupling between ATP-binding and lever-arm regions in myosin via dielectric allostery. J. Chem. Phys. 2017; 147:215101. 29221399 10.1063/1.5004809

[147] Sato T., Ohnuki J., Takano M. Dielectric allostery of protein: response of myosin to ATP binding. J. Phys. Chem. B. 2016; 120:13047–13055. 28030954 10.1021/acs.jpcb.6b10003

[148] del Sol A., Tsai C.-J., Ma B., Nussinov R. The origin of allosteric functional modulation: multiple pre-existing pathways. Structure. 2009; 17:1042–1050. 19679084 10.1016/j.str.2009.06.008 PMC2749652

[149] Arantes P.R., Patel A.C., Palermo G. Emerging methods and applications to decrypt allostery in proteins and nucleic acids. J. Mol. Biol. 2022; 434:167518. 35240127 10.1016/j.jmb.2022.167518 PMC9398933

[150] Liu J., Nussinov R. Allostery: an overview of its history, concepts, methods, and applications. PLOS Comput. Biol. 2016; 12:e1004966. 27253437 10.1371/journal.pcbi.1004966 PMC4890769

[151] Nechushtai R., Lammert H., Michaeli D., Eisenberg-Domovich Y., Zuris J.A., Luca M.A., Capraro D.T., Fish A., Shimshon O., Roy M. et al. Allostery in the ferredoxin protein motif does not involve a conformational switch. Proc. Natl Acad. Sci. U.S.A. 2011; 108:2240–2245. 21266547 10.1073/pnas.1019502108 PMC3038707

[152] Banerjee-Ghosh K., Ghosh S., Mazal H., Riven I., Haran G., Naaman R. Long-range charge reorganization as an allosteric control signal in proteins. J. Am. Chem. Soc. 2020; 142:20456–20462. 33211484 10.1021/jacs.0c10105 PMC7735699

[153] Ghosh S., Banerjee-Ghosh K., Levy D., Scheerer D., Riven I., Shin J., Gray H.B., Naaman R., Haran G. Control of protein activity by photoinduced spin polarized charge reorganization. Proc. Natl Acad. Sci. U.S.A. 2022; 119:e2204735119. 35994638 10.1073/pnas.2204735119 PMC9436351

[154] Corbella M., Voityuk A.A., Curutchet C. How abasic sites impact hole transfer dynamics in GC-rich DNA sequences. Phys. Chem. Chem. Phys. 2018; 20:23123–23131. 30168547 10.1039/c8cp03572e

[155] Sosorev A.Y. Walking around ribosomal small subunit: a possible “tourist map” for electron holes. Molecules. 2021; 26:5479. 34576950 10.3390/molecules26185479 PMC8467113

[156] Sosorev A., Kharlanov O. Organic nanoelectronics inside us: charge transport and localization in RNA could orchestrate ribosome operation. Phys. Chem. Chem. Phys. 2021; 23:7037–7047. 33448272 10.1039/d0cp04970k

[157] Wang P., Leontyev I., Stuchebrukhov A.A. Mechanical allosteric couplings of redox-induced conformational changes in Respiratory complex I. J. Phys. Chem. B. 2022; 126:4080–4088. 35612955 10.1021/acs.jpcb.2c00750

[158] Isom D.G., Sridharan V., Baker R., Clement S.T., Smalley D.M., Dohlman H.G. Protons as second messenger regulators of G protein signaling. Mol. Cell. 2013; 51:531–538. 23954348 10.1016/j.molcel.2013.07.012 PMC3770139

[159] Kapolka N.J., Rowe J.B., Taghon G.J., Morgan W.M., O’Shea C.R., Isom D.G. Proton-gated coincidence detection is a common feature of GPCR signaling. Proc. Natl Acad. Sci. U.S.A. 2021; 118:e2100171118. 34260394 10.1073/pnas.2100171118 PMC8285908

[160] Harris R.C., Tsai C.-C., Ellis C.R., Shen J. Proton-coupled conformational allostery modulates the inhibitor selectivity for β-secretase. J. Phys. Chem. Lett. 2017; 8:4832–4837. 28927275 10.1021/acs.jpclett.7b02309 PMC5713904

[161] Tsai C.-C., Yue Z., Shen J. How electrostatic coupling enables conformational plasticity in a tyrosine kinase. J. Am. Chem. Soc. 2019; 141:15092–15101. 31476863 10.1021/jacs.9b06064 PMC6933753

[162] Dempsey J.L., Winkler J.R., Gray H.B. Proton-coupled electron flow in protein redox machines. Chem. Rev. 2010; 110:7024–7039. 21082865 10.1021/cr100182b PMC3005815

[163] Abeysirigunawardena S.C., Kim H., Lai J., Ragunathan K., Rappé M.C., Luthey-Schulten Z., Ha T., Woodson S.A. Evolution of protein-coupled RNA dynamics during hierarchical assembly of ribosomal complexes. Nat. Commun. 2017; 8:492. 28887451 10.1038/s41467-017-00536-1 PMC5591316

[164] Ghode A., Gross L.Z.F., Tee W.-V., Guarnera E., Berezovsky I.N., Biondi R.M., Anand G.S. Synergistic allostery in multiligand–protein interactions. Biophys. J. 2020; 119:1833–1848. 33086047 10.1016/j.bpj.2020.09.019 PMC7677135

[165] Shin H., Ren Z., Zeng X., Bandara S., Yang X. Structural basis of molecular logic OR in a dual-sensor histidine kinase. Proc. Natl Acad. Sci. U.S.A. 2019; 116:19973–19982. 31527275 10.1073/pnas.1910855116 PMC6778262

[166] Jiao W., Hutton R.D., Cross P.J., Jameson G.B., Parker E.J. Dynamic cross-talk among remote binding sites: the molecular basis for unusual synergistic allostery. J. Mol. Biol. 2012; 415:716–726. 22154807 10.1016/j.jmb.2011.11.037

[167] Webby C.J., Jiao W., Hutton R.D., Blackmore N.J., Baker H.M., Baker E.N., Jameson G.B., Parker E.J. Synergistic allostery, a sophisticated regulatory network for the control of aromatic amino acid biosynthesis in mycobacterium tuberculosis*. J. Biol. Chem. 2010; 285:30567–30576. 20667835 10.1074/jbc.M110.111856 PMC2945551

[168] Westhof E., Watson Z.L., Zirbel C.L., Cate J.H.D. Anionic G•U pairs in bacterial ribosomal rRNAs. RNA. 2023; 29:1069–1076. 37068913 10.1261/rna.079583.123 PMC10275268

[169] Genuth N.R., Barna M. The discovery of ribosome heterogeneity and its implications for gene regulation and organismal life. Mol. Cell. 2018; 71:364–374. 30075139 10.1016/j.molcel.2018.07.018 PMC6092941

[170] Dinman J.D. Pathways to specialized ribosomes: the Brussels lecture. J. Mol. Biol. 2016; 428:2186–2194. 26764228 10.1016/j.jmb.2015.12.021 PMC4884523

[171] Chen Y.-X., Xu Z., Ge X., Hong J.-Y., Sanyal S., Lu Z.J., Javid B. Selective translation by alternative bacterial ribosomes. Proc. Natl Acad. Sci. U.S.A. 2020; 117:19487–19496. 32723820 10.1073/pnas.2009607117 PMC7431078

[172] Sulima S.O., Hofman I.J.F., De Keersmaecker K., Dinman J.D. How ribosomes translate cancer. Cancer Discov. 2017; 7:1069–1087. 28923911 10.1158/2159-8290.CD-17-0550 PMC5630089

[173] Levin M. Molecular bioelectricity: how endogenous voltage potentials control cell behavior and instruct pattern regulation *in vivo*. Mol. Biol. Cell. 2014; 25:3835–3850. 25425556 10.1091/mbc.E13-12-0708 PMC4244194

[174] Levin M. Bioelectric signaling: reprogrammable circuits underlying embryogenesis, regeneration, and cancer. Cell. 2021; 184:1971–1989. 33826908 10.1016/j.cell.2021.02.034

[175] Levin M. Bioelectric networks: the cognitive glue enabling evolutionary scaling from physiology to mind. Anim. Cogn. 2023; 26:1865–1891. 37204591 10.1007/s10071-023-01780-3 PMC10770221

