# Supplementary material for: The role of ribosomal protein networks in ribosome dynamics
Source: Nucleic Acids Res. 2025 Jan 10;53(1):gkae1308. doi: 10.1093/nar/gkae1308 (PMC11711686; doi:10.1093/nar/gkae1308)
Supplement: gkae1308_Supplemental_Files [file gkae1308_supplemental_files.zip › Supplementary_TEXT_TABLE_FIG_revised_36.pdf]

The role of ribosomal protein networks in ribosome dynamics

Supplementary Texts, Tables and Figures

Youri Timsit[1, 2, *], Grégoire Sergeant-Perthuis[3] and Daniel Bennequin[4]

1.Aix Marseille Univ, Université de Toulon, CNRS, IRD, MIO UM110, 13288 Marseille, France;
2. Research Federation for the Study of Global Ocean Systems Ecology and Evolution, FR2022/Tara GOSEE, 3 Rue Michel-Ange, 75016 Paris, France

3.Laboratory of Computational and Quantitative Biology (LCQB, Sorbonne Université Paris)
4. Institut de Mathématiques de Jussieu - Paris Rive Gauche (IMJ-PRG), UMR 7586, CNRS, Université Paris Diderot, Paris, France

*** Corresponding author : youri.timsit@mio.osupytheas.fr**

Supplementary Text 1: The dynamics of the bacterial ribosome during the elongation phase

A. Large and small-scale rearrangements during the decoding step

Elongation begins with the decoding -a two-step process including the initial selection and the proofreading that achieves the high fidelity of decoding. The minimization of miscoding events results from the continuous verification of tRNA throughout its trajectory (1, 2). Initial selection discriminates correct tRNA before GTP hydrolysis by EF-Tu and leads to the GTPase activation. The correct codon-anticodon pairing induces both a local conformational change in the decoding centre: the flipping of A1492 and A1493, G530 that fasten the codon-anticodon helix and the global closure of the SSU head and body domains (3–6). The three bases shield the codon-anticodon base pair, prevent its interaction with the solvent and increase the free-energy difference between the cognate and near cognate (7, 8). Upon the EF-Tu binding the bL12 stalk curls inward the Peptidyl Transfer Center (PTC) and a large conformational change is observed in tRNA (9). Once the cognate tRNA is selected, the GTPase centre of the ribosome is activated and EF-Tu is released from the ribosome. The conformational dynamics is also crucial for GTPase activation: the GTP hydrolysis is more rapid for a cognate than a near cognate codon. The SSU closure drags the tRNA and the EF-Tu toward the SRL and activates the GTPase of the EF-Tu (10–12). After hydrolysis and before the peptidyl transfer, the proofreading rejects incorrectly selected tRNAs. The aminoacyl end of the tRNA is then accommodated into the A-site of the 50S peptidyl transferase center, where the peptide bond is formed (13–16). The peptide bond is then created between the peptide chain of the P-site tRNA-P and the amino acid of the A-tRNA (17, 18). At this step, the tRNA substrate plays an active role in the conformation of the ribosome active site, which remains inactive in the absence of substrate (19). This is the structure of the PTC that act as an entropic catalyst to accelerate the peptide bond formation (20). The interplay of rigid and mobile regions also play a critical role in peptide bond formation (21, 22). This results in formation of a peptidyl tRNA which occupies the ribosomal A-site.

B. Ribosome dynamics during translocation

Ribosomes engage in a complex choreography involving both large- and small-scale movements during the translocation of the peptidylated tRNA-A and deacylated tRNA-P into the P and E sites respectively. A complex dynamics ensures that tRNAs and mRNAs move together while avoiding the loss of the reading frame. This dynamics combines the intersubunit rotations (23–25), the 30S head/body swivelling (26) and the 50S stalk movements. Finer movements of a set of rRNA helices collectively regulate the functional sites during the directional translocation of the substrates (27–30). The head/body swivelling (26, 30–33) - the rotation of the 30S subunit head relatively to the body- accompanies the tRNA from the P to the E site of the SSU. Head swivelling is essential because it opens a constriction (13 Å) in the path between the 30S P and E sites (34) that forms a steric barrier to the tRNA movement during translocation (A790 and G1378 and U1341) (35). During rotation of the 30S subunit head domain, the P-sites contacts between the head and the ASL (G1338, A1339 and G966 of the 16 rRNA) and the C-ter tails of uS13 and uS9 are maintained while the P-site contacts with the 30S body are disrupted (35, 36). The P-site ASL moves precisely with rotation of 30S domain while the A-site ASL move further than the head rotation to avoid clash with domain IV of EF-G. The tRNAs are then translocated into the classic P/P and E/E state by reverse subunit rotation and back-swivelling. Translocation is accompanied by conformation change of EF-G and coupled to the docking of domain IV of EF-G into the empty A-site of the SSU. Then, GTP hydrolysis and Pi release trigger EF-G dissociation form the ribosome (37, 38). However, GTP hydrolysis and translocation

do not appear to be directly coupled and its role is still controversial. The uL1 stalk (composed of uL1, H76, H77 and H78 of 23S rRNA) also undergoes large-scale movements coupled to the translocation of deacylated tRNA and the intersubunit rotation (39–45). The stalk moves according to the binding state of the deacylated tRNA (46, 47). In the classical A/A, P/P state, uL1-stalk is open while in the rotated A/P, P/E hybrid state, uL1-stalk is closed and interact with the elbow of P/E tRNA. It then follows movement the tRNA through the pe/E chimeric hybrid (48) and classical E/E state (49). In the absence of EF-G, uL1-stalk spontaneously fluctuates between closed and open position. Beyond large-scale motions and domain movements, there are many small adjustments of helices that are indicated by the many hinges found by the comparison of ribosome structures in different functional states (50, 51). The head rotation is a complex movement obtained by several movements of helices (33). According to Mohan and collaborators, the movement of head swivelling results from the rotation around two helical axes. They have identified two hinges in the 16S rRNA responsible for the head swivelling. Hinge 1 is located in the neck (h28) at the point of contact of mRNA. Hinge 2 is located in the coaxially stacked h35 and h36, which form an A-minor interaction between the head and body domain. The hinges 1 and 2 correspond roughly to p1394 and p1074 described by Paci and Fox. Interestingly, the antibiotic spectinomycin traps the head in a partially swivelled state and drastically reduces the rate of translocation. The spectinomycin binding sites is localized to G1064, 1066, 1068, A1191 and C1192 and G1193 at the h34/h35 junction (52–55). Thus, the hinge 2 area (p1074) that plays a critical role in head swivelling may be modulated by external ligands. This supports the view that rRNA mobility can be controlled by external factors such as antibiotics or r-proteins.

Supplementary Text 2: Properties of the r-protein networks

A. r-protein extensions and networking

The r-proteins exhibit long filamentous extensions that still remain both an evolutionary and functional mystery. Due to their charged and dynamic nature, it has been proposed that they could contribute to ribosome assembly (56–61). However, in addition to their interaction with rRNA, r-protein extensions systematically form r-protein networks through tiny interactions, within the three kingdom ribosomes (62–64) (Supplementary Figure S8). Unlike most protein networks that occur transiently in the cells (65–67), r-protein networks are particular in that they are woven by permanent interactions in mature ribosomes, once the stages of their biogenesis is complete (68–70).

The structural comparison of the three kingdom's ribosomes (A: archael, B: bacterial and E: Eukaryal) has inferred that a universal r-protein network (ABE) probably existed in LUCA before the radiation of the three kingdoms (71–73) (Supplementary Figure S9). It has been proposed that this ABE r-protein network was already well connected to functional sites such as the PTC, mRNA, the tRNAs and the peptide exit tunnel. The connectivity has then increased from this common core, until it reached its peak in the eukaryotic ribosomes. Each kingdom's network has followed its own pathway of evolution leading to graphs with both common and distinct mathematical properties. The networks are remarkably well conserved among kingdom and despite their differences in complexity, they share a common architecture that has been probably shaped by both divergent and convergent evolution.

B. Co-evolution for networking

A remarkable finding was that the extensions have been concurrently acquired on remote r-proteins for establishing new connections between them. This coordinated evolutionary changes between constituents termed co-evolution (74, 75) has been found significantly amplified in the archaea -> eukaryote transition, in agreement with the finding that the acquisition of new protein insertions correlate with network rewiring (76). Co-evolution has been also observed at the amino acid level. For example, the appearance of new motifs that constitute the signature of r-protein-r-protein contacts correlate with the appearance of new inter-protein interactions. In the *M. smegmatis* and *E. coli ribosomes*, these new types of motifs correlate at the vicinity of the interaction points of the new extensions (Supplementary Figure S8, E and F). Interestingly, unlike the growing involvement of extensions into networking, extension-rRNA interactions only slightly increase from prokaryotes to eukaryotes. Moreover, in the eukaryotic ribosomes, the eukaryotic specific extensions rather interact with other r-proteins than with eukaryotic specific RNA expansion segments. These studies demonstrated that the acquisition of new extensions during evolution are mainly linked to their roles protein-protein interactions. The phenomena of co-evolution, which are expressed at two levels thus reinforces the idea that r-protein networks are under a strong selective pressure.

C. Graph theory and r-protein network functions

Interesting correspondences between the graph theory and the ribosome biology have been noticed. Graph theory has first demonstrated that the node inter-connectivity is non-randomly distributed in the three kingdom's networks. The betweenness centrality of each nodes (that measures the extent to which a node influences the information spread on the network) strikingly correlates with functional studies. For example, "without knowing ribosome biology", mathematics have highlighted a set of particular nodes, the maxima of betweenness centrality in the three kingdom's networks. Intriguingly, these nodes correspond to the PTC, which is one of the most important ribosome functional sites. This reveals that bacterial and archaeal networks have evolved convergently so that the PTC becomes a major player in information spread, in perfect agreement with its central role in ribosome catalysis (77) and allostery (78–80). In eukaryotic ribosomes, network control is also distributed to other actors including uL16, tRNA-A, eL8 and uL4. Interestingly, uL16 has been also highlighted by biochemical studies as a key player in information transmission of the eukaryotic ribosome (81). These findings converge towards the idea that during evolution, the r-protein networks play a growing role in information transmission in ribosomes (64, 82).

D. R-protein networks and ribosome signalling

Growing experimental evidences have shown indeed that distant ribosomal functional sites not only continuously "sense" incoming molecular signals but also "transmit" them to each other. For example, long-range signalling between the decoding centre monitors the correct geometry of the codon-anticodon and other distant sites such as the Sarcin Ricin Loop (SRL) or the E-tRNA site (83, 84). The r-protein uL3 also plays a key role in the allosteric coordination of the PTC (77) and the A-site (85). Similarly, the r-proteins that sense the nascent peptide within the exit tunnel participate in the regulation of co-translational folding and communicate with remote functional sites such as the PTC (86, 87). Communication processes also coordinate the complex ribosomal movements during translation, such as the ratchet-like motion between the two subunits (81, 88–91).

Because it is experimentally difficult to disentangle their respective roles in ribosome assembly and in translation, the functions of r-proteins or rRNA expansion segments still remain a matter of debate (60, 92). Finding that ribosome are ribozymes (93) has long relegated r-proteins to the rank of auxiliaries in translation mainly invested in ribosome biogenesis (68–70, 94–98). Extensions have been proposed to participate in the organization of rRNA domains during ribosome assembly (56, 57, 59–61). Mutagenic studies have shown, for example, that the N-terminal extension of uS12, eL8, uL29 and uL30 play a predominant role in the assembly of the bacterial SSU (99) or the eukaryotic LSU (100). Conversely, it has been shown that extensions also participate in both translation and ribosomal assembly (101–104). Many studies of the Dinman's group have demonstrated that r-proteins and their extensions contribute in long-range communication between functional sites (85, 105–111). Several studies have also highlighted the role of r-protein communication for efficient translation and its link to yeast signalling pathways (112, 113). The dramatic expansion of r-protein networks in the tree of life provides a conceptual framework that integrates these data in the light of evolution. Our previous study suggested that r-proteins evolved collectively towards multiple functions and were progressively dedicated to share and process information coming from remote functional sites during the protein synthesis. Previous studies have revealed that rRNA can also participate in remote communication between functional sites (78, 80, 114, 115). It is therefore possible that rRNA and r-protein allosteric networks have also co-evolved for exchanging information (82). The expansion of network connectivity remarkably parallels the increasing accuracy and complexity of the ribosome's tasks from prokaryotes to eukaryotes (116–118). Since any changes that affect network connectivity should modify the translation efficiency and accuracy, the concept of r-protein network in signalling provides new hints to understand how ribosome heterogeneity regulates translation (119, 120) and diseases (121). Furthermore, the finding of new pathways and shortcuts in the networks of parasitic and pathogenic species such as *plasmodium* or *mycobacterium* opens new therapeutic perspectives.

Supplementary text 3: details of relative RNA/protein movements

A. Factors that influence the rRNA/r-protein mobility

The movement of rRNA around r-proteins is influenced by a number of factors, both external and internal. In the LSU, the passage of the tRNA from the A site to the P site causes H38 to move away from uL16 in step 3. At step 4, EF-G pushes the bL12-stalk that induces H42 to move away from the other side of the protein (Figure 5C). Another example shows that at step 6, helix h21 moves significantly away from uS17 that remains anchored to helices h7 and h11 (Figure 5D). The movement of h21 relatively to uS17 appears to be indirectly caused by the LSU h34 pushing on uS15 during the subunit rotation. Another type of situation occurs at the SSU head/body interface, where swivelling gradually moves the h16 and h18 helices away from uS3 which remains firmly attached to h34, uS10 and uS14, from step 2 to step 6 (Figure 5E). Interestingly, More complex is the movement of the h35 helix relative to uS3. Unlike h16 and h18 helices driven by head/body swivelling, h35 does not move away progressively from uS3 but together with uS2 and uS5, makes a major leap forward specifically at stage 5 (EF-G bound after GTP hydrolysis) (Supplementary Figure S21A). It's as if uS3 accumulated motion energy during steps 2-4 and released it abruptly at step 5. This motion depends on a range of factors including the head/body swivelling but also the remodelling of the interfaces uS3-uS2, uS4-uS5 and uS3-uS5 (Supplementary Figure S21, B-E). These r-protein interfaces are mediated by repulsive interactions involving well-conserved pairs of Glu or Asp.

Far from areas where tRNAs pass through, and at the interfaces of rotating subunits, movements between r-proteins and rRNA are also observed. For example, RNA helix h26 moves slightly along uS2 in steps 5 and 6, while helices h37 and h40 move significantly closer towards uS2 in step 5 (Figure 5F, Supplementary Figure S21B). In addition, around bS16, the RNA mobility exhibits a different behaviour. Instead of a gradual movement in one direction, the rRNA helices "come and go" during each step (Figure 5B). For example, helix h21 sees its groove contract in steps 3 and 4, expand in step 5, and contract again in step 6. During the translocation steps, helix h17 displays the same oscillation around its original position relative to bS16. In summary, most r-proteins participate in a specific dynamic with their rRNA environment and play a key role in the modulation of the RNA domain tectonic during the translocation steps. Reciprocally, they undergo periodical electrostatic changes due to the "distance/approach" cycles with rRNA.

B. How distance approach cycles control the overal dynamics

The diameter of the mRNA channel entrance and exit is subject to temporal variation, which is dependent on a complex network of interactions. A series of protein-protein and protein-RNA distance/approach cycles modulate, like an electrostatic diaphragm, its diameter during the different stages of translocation (Figure 7A-C; Supplementary S21-S22). On the head side, uS3 controls its distance from the h35 helix. Between the body and the head, uS2 acts on the h35/h36 helices around the p1074 pivot, while on the body side, uS4 acts on the h17 helices around the p441 pivot (Figure 5E-F, Supplementary S21). The size of the mRNA channel is also controlled by distant/approach cycles between the r-proteins uS4-uS5, uS3-uS5 and uS2-uS3 (see above). The possible repulsion between the strictly conserved Glu and Asp residues located at their interfaces probably plays a key role in this process (Supplementary Figure S21). uS3 therefore both controls and perceives several movements at once, the body/head rotation on the one hand, and the internal head movements on the other. uS10 is unusual in that most of the helices around it undergo local deformation, particularly at step 4 (Figure 7B). For example, the major groove of helix h39 is compressed and h41 and uS13 are moved away. uS10 is also linked to uS9, uS3 and uS14 throughout the steps and subtly controls movements around strategic pivots such as p1125, p1156 and p1247. Further on, local deformations of helix h41 (cyan) (pivot p1247) are reflected in helix h42 (yellow) linked to uS13 and uS19, which are involved in LSU/SSU rotation. These subtle movements contribute to a lateral displacement of the part of the Head in contact with the LSU, towards the tRNA-E site, driving uS13 and uS19 (Figure 7B and Supplementary Figure S7).

In the body, at step 6 (back-swivelling), the large helices h21 and h44 move away from uS17 and uS12, respectively (Figure 8A-C). This rearrangement also involves the helices h27, h20 and h14/h3 and splits the body into two blocks. One is formed by the group of h1-h5, h12 and h18 helices firmly anchored to the "outer" face of uS12 (in yellow) (Figure 8C). The other consists of the group of helices h8, h11, h13, h14, h20, h27 and h44, which move away from uS12 (cyan), following the same direction that the one of trajectory of the tRNAs. The fracture between uS12 and this region of the body spreads to uS15, uS17 and bS20 and displaces the uS12 extension where it interacts with uS17 and uS8.

C. CMs protein interactions and motifs that repel RNA

While rRNA immobile regions are generally stabilized by basic, polar or sometimes aromatic residue clamps, the moving arms are located opposite motifs rich in acidic, histidine or tryptophan residues. Emblematic cases of residues observed near moving pivot bases are, for example, Asp 33 (uS7) - U 1351, Glu 175 and Trp 96

(uS2)-G1074 or His 15, 70 and Asp 14 and 19 of uS10 near the moving strand 1152-1155 (CM 1225) (Figure 9; Supplementary Figure 24). Thus, as seen above, motifs opposite moving regions have electrostatic components that can repel rRNA from the r-proteins. They can either play a passive role, facilitating RNA removal from proteins when moved by external factors, or a more active role, if we consider that their charges vary and can modulate their affinities for RNA during the different stages of translocation. The r-proteins attached to the CMs therefore play antagonistic roles, immobilising certain regions of the RNA by "steric clamps" and facilitating the movement of others by "electrostatic lever arms".

The special r-protein motifs observed at the vicinity of CMs would act as "molecular transistor" for controlling local rRNA motions. They share some analogy with those involved in r-protein interactions in the network (122–124). These properties are conducive to combine various mechanisms such as the classical allostery with (125–127), or without molecular switches (128) charge transfer allostery (129–134), or even proton or electron transfer (135) (136–145). The combination of these various mechanisms may therefore transmit multiple signals that reflect the electrostatic status of remote ribosome regions to modulate both local and global rRNA motions, either for coordinating the ribosome dynamic or for acting in functional sites. In addition, of particular interest for understanding how r-proteins can integrate multiple signals from the network, are the synergistic allosteric systems involving multiple ligand-binding sites observed in certain enzymes or kinases (146–150). These works that show how proteins may integrate multiple signals can be useful for understanding the behaviours of r-proteins within the network. The integration of these cues in time and space coming from distant functional sites may contribute to coordinate the overall ribosome dynamics. This model both provides a new conceptual framework to understand why the evolution has woven such complex r-protein networks (122, 123) and the electrostatic mechanisms that can contribute to synchronize ribosome dynamics.

Several experimental studies have shown that the negatively charged RNA or DNA phosphate groups or the acidic residues in proteins can either destabilize or push nucleic acid helices away. In the ribosome, the compact packing RNA helices forms a tense system dominated by the repulsion between the tightly packed negatively charged phosphate groups, similar in density to a nucleic acid crystal (151). In these critical systems, it has been shown that small electrostatic disturbances can have major consequences. For example, it has been shown that the tight compaction of DNA helices can reveal fragile zones –the compaction responsive sequences- that are deformed by repulsive electrostatic interactions (152–154) and whose dynamics are modulated by divalent cation binding (154, 155). These regions (156) are therefore reminiscent to the ribosomal CMs that also constitute flexible zones of the rRNA. In another hand, it has been shown that a linker containing a cluster of acidic residues repel the non-cognate unmethylated DNA from the DNMT1 methylase (157, 158). Several studies have also shown that protein negatively charged residues play a critical role in the selectivity of RNA or DNA sequences (159–162).

D. Context dependent electrostatic changes in proteins

It is well known that protein charges can vary according to their structural or their electrostatic context (163, 164). Numerous experimental and theoretical studies have shown that the pKa and/or the redox potentials of protein groups may change according to their environment or their interactions with different ligands, including nucleic acids (165–170). Histidines in particular are well known to easily switch from the neutral to the positive state, depending on subtle differences in their molecular environment (171–173). But the charge of other amino acids such as Glu, Asp and Lys can be also modulated by their electrostatic contexts or protein stability (174–178). In contrast, Arg remains charged in various environments and even when it is buried in internal position in proteins

(179, 180). Experiments demonstrating long range coupling between ligand binding and pKa changes is particularly relevant for supporting the idea of the propagation of electrostatic changes at distant sites in the ribosome. It has been shown that substantial pKa changes may occurs well outside the binding interfaces, far as 24 Å of the ligand and even further (181, 182).

What we observe in the ribosome has also been described in protein-DNA interactions. Recent studies have shown indeed that transient electrostatic changes in proteins can modulate their binding to DNA (136, 183, 184). For example, in the transcription regulator RsrR, the electron and proton transfer drive redox-dependent protein conformational changes that modulate DNA binding (136). Such a mechanism provides precious insights for understanding how r-protein electrostatic can influence at distance, the mobility of rRNA.

References

1. Loveland,A.B., Demo,G., Grigorieff,N. and Korostelev,A.A. (2017) Ensemble cryo-EM elucidates the mechanism of translation fidelity. 10.1038/nature22397.

2. Loveland,A.B., Demo,G. and Korostelev,A.A. (2020) Cryo-EM of elongating ribosome with EF-Tu•GTP elucidates tRNA proofreading. *Nature*, **584**, 640–645.

3. Ogle,J.M., Brodersen,D.E., Clemons,W.M., Tarry,M.J., Carter,A.P. and Ramakrishnan,V. (2001) Recognition of cognate transfer RNA by the 30S ribosomal subunit. 10.1126/science.1060612.

4. Ogle,J.M., Murphy,F.V., Tarry,M.J. and Ramakrishnan,V. (2002) Selection of tRNA by the Ribosome Requires a Transition from an Open to a Closed Form. 10.1016/S0092-8674(02)01086-3.

5. Ogle,J.M. and Ramakrishnan,V. (2005) Structural insights into translational fidelity. 10.1146/annurev.biochem.74.061903.155440.

6. Schmeing,T.M., Voorhees,R.M., Kelley,A.C., Gao,Y.-G., Murphy,F.V., Weir,J.R. and Ramakrishnan,V. (2009) The crystal structure of the ribosome bound to EF-Tu and aminoacyl-tRNA. *Science*, **326**, 688–694.

7. Satpati,P., Sund,J. and Aqvist,J. (2014) Structure-based energetics of mRNA decoding on the ribosome. *Biochemistry*, **53**, 1714–1722.

8. Bock,L.V., Kolář,M.H. and Grubmüller,H. (2018) Molecular simulations of the ribosome and associated translation factors. *Curr Opin Struct Biol*, **49**, 27–35.

9. Frank,J., Sengupta,J., Gao,H., Li,W., Valle,M., Zavialov,A. and Ehrenberg,M. (2005) The role of tRNA as a molecular spring in decoding, accommodation, and peptidyl transfer. *FEBS Lett*, **579**, 959–962.

10. Fischer,N., Neumann,P., Konevega,A.L., Bock,L.V., Ficner,R., Rodnina,M.V. and Stark,H. (2015) Structure of the E. coli ribosome-EF-Tu complex at <3 Å resolution by Cs-corrected cryo-EM. 10.1038/nature14275.

11. Noel,J.K. and Whitford,P.C. (2016) How EF-Tu can contribute to efficient proofreading of aa-tRNA by the ribosome. *Nat Commun*, **7**, 13314.

12. Schuette,J.-C., Murphy,F.V., Kelley,A.C., Weir,J.R., Giesebrecht,J., Connell,S.R., Loerke,J., Mielke,T., Zhang,W., Penczek,P.A., *et al.* (2009) GTPase activation of elongation factor EF-Tu by the ribosome during decoding. *EMBO J*, **28**, 755–765.

13. Rodnina,M.V., Fischer,N., Maracci,C. and Stark,H. (2017) Ribosome dynamics during decoding. *Philosophical Transactions of the Royal Society B: Biological Sciences*, **372**, 20160182.

14. Wohlgemuth,I., Pohl,C., Mittelstaet,J., Konevega,A.L. and Rodnina,M.V. (2011) Evolutionary optimization of speed and accuracy of decoding on the ribosome. 10.1098/rstb.2011.0138.

15. Demeshkina,N., Jenner,L., Westhof,E., Yusupov,M. and Yusupova,G. (2012) A new understanding of the decoding principle on the ribosome. *Nature*, **484**, 256–259.

16. Sanbonmatsu,K.Y., Joseph,S. and Tung,C.-S. (2005) Simulating movement of tRNA into the ribosome during decoding. *Proc Natl Acad Sci U S A*, **102**, 15854–15859.

17. Voorhees,R.M., Weixlbaumer,A., Loakes,D., Kelley,A.C. and Ramakrishnan,V. (2009) Insights into substrate stabilization from snapshots of the peptidyl transferase center of the intact 70S ribosome. 10.1038/nsmb.1577.

18. Voorhees,R.M. and Ramakrishnan,V. (2013) Structural basis of the translational elongation cycle. 10.1146/annurev-biochem-113009-092313.

19. Steitz,T.A. (2008) A structural understanding of the dynamic ribosome machine. *Nat Rev Mol Cell Biol*, **9**, 242–253.

20. Rodnina,M.V., Beringer,M. and Wintermeyer,W. (2006) Mechanism of peptide bond formation on the ribosome. 10.1017/S003358350600429X.

21. Trobro,S. and Aqvist,J. (2005) Mechanism of peptide bond synthesis on the ribosome. *Proc Natl Acad Sci U S A*, **102**, 12395–12400.

22. Sievers,A., Beringer,M., Rodnina,M.V. and Wolfenden,R. (2004) The ribosome as an entropy trap. *Proc Natl Acad Sci U S A*, **101**, 7897–7901.

23. Noeske,J. and Cate,J.H.D. (2012) Structural basis for protein synthesis: snapshots of the ribosome in motion. 10.1016/j.sbi.2012.07.011.

24. Schuwirth,B.S., Borovinskaya,M.A., Hau,C.W., Zhang,W., Vila-Sanjurjo,A., Holton,J.M. and Cate,J.H.D. (2005) Structures of the bacterial ribosome at 3.5 A resolution. 10.1126/science.1117230.

25. Valle,M., Zavialov,A., Sengupta,J., Rawat,U., Ehrenberg,M. and Frank,J. (2003) Locking and unlocking of ribosomal motions. 10.1016/s0092-8674(03)00476-8.

26. Wimberly,B.T., Brodersen,D.E., Clemons,W.M., Morgan-Warren,R.J., Carter,A.P., Vonrhein,C., Hartsch,T. and Ramakrishnan,V. (2000) Structure of the 30S ribosomal subunit. 10.1038/35030006.

27. Belardinelli,R., Sharma,H., Caliskan,N., Cunha,C.E., Peske,F., Wintermeyer,W. and Rodnina,M.V. (2016) Choreography of molecular movements during ribosome progression along mRNA. 10.1038/nsmb.3193.

28. Noller,H.F., Lancaster,L., Mohan,S. and Zhou,J. (2017) Ribosome structural dynamics in translocation: yet another functional role for ribosomal RNA. 10.1017/S0033583517000117.

29. Ling,C. and Ermolenko,D.N. (2016) Structural insights into ribosome translocation. *Wiley Interdiscip Rev RNA*, **7**, 620–636.

30. Ratje,A.H., Loerke,J., Mikolajka,A., Brünner,M., Hildebrand,P.W., Starosta,A.L., Dönhöfer,A., Connell,S.R., Fucini,P., Mielke,T., *et al.* (2010) Head swivel on the ribosome facilitates translocation by means of intra-subunit tRNA hybrid sites. 10.1038/nature09547.

31. Hassan,A., Byju,S., Freitas,F.C., Roc,C., Pender,N., Nguyen,K., Kimbrough,E.M., Mattingly,J.M., Gonzalez,R.L., de Oliveira,R.J., *et al.* (2023) Ratchet, swivel, tilt and roll: a complete description of subunit rotation in the ribosome. *Nucleic Acids Res*, **51**, 919–934.

32. Guo,Z. and Noller,H.F. (2012) Rotation of the head of the 30S ribosomal subunit during mRNA translocation. *Proc Natl Acad Sci U S A*, **109**, 20391–20394.

33. Mohan,S., Donohue,J.P. and Noller,H.F. (2014) Molecular mechanics of 30S subunit head rotation. *Proc Natl Acad Sci U S A*, **111**, 13325–13330.

34. Schuwirth,B.S., Borovinskaya,M.A., Hau,C.W., Zhang,W., Vila-Sanjurjo,A., Holton,J.M. and Cate,J.H.D. (2005) Structures of the bacterial ribosome at 3.5 A resolution. *Science*, **310**, 827–834.

35. Noller,H.F., Lancaster,L., Zhou,J. and Mohan,S. (2017) The ribosome moves: RNA mechanics and translocation. *Nat Struct Mol Biol*, **24**, 1021–1027.

36. Zhou,J., Lancaster,L., Donohue,J.P. and Noller,H.F. (2014) How the ribosome hands the A-site tRNA to the P site during EF-G-catalyzed translocation. *Science*, **345**, 1188–1191.

37. Savelsbergh,A., Katunin,V.I., Mohr,D., Peske,F., Rodnina,M.V. and Wintermeyer,W. (2003) An elongation factor G-induced ribosome rearrangement precedes tRNA-mRNA translocation. *Mol Cell*, **11**, 1517–1523.

38. Savelsbergh,A., Mohr,D., Kothe,U., Wintermeyer,W. and Rodnina,M.V. (2005) Control of phosphate release from elongation factor G by ribosomal protein L7/12. *EMBO J*, **24**, 4316–4323.

39. Bock,L.V., Blau,C., Schröder,G.F., Davydov,I.I., Fischer,N., Stark,H., Rodnina,M.V., Vaiana,A.C. and Grubmüller,H. (2013) Energy barriers and driving forces in tRNA translocation through the ribosome. *Nat Struct Mol Biol*, **20**, 1390–1396.

40. Cornish,P.V., Ermolenko,D.N., Staple,D.W., Hoang,L., Hickerson,R.P., Noller,H.F. and Ha,T. (2009) Following movement of the L1 stalk between three functional states in single ribosomes. *Proc Natl Acad Sci U S A*, **106**, 2571–2576.

41. Mohan,S. and Noller,H.F. (2017) Recurring RNA structural motifs underlie the mechanics of L1 stalk movement. 10.1038/ncomms14285.

42. Munro,J.B., Altman,R.B., O'Connor,N. and Blanchard,S.C. (2007) Identification of two distinct hybrid state intermediates on the ribosome. *Mol Cell*, **25**, 505–517.

43. Munro,J.B., Wasserman,M.R., Altman,R.B., Wang,L. and Blanchard,S.C. (2010) Correlated conformational events in EF-G and the ribosome regulate translocation. *Nat Struct Mol Biol*, **17**, 1470–1477.

44. Fei,J., Kosuri,P., MacDougall,D.D. and Gonzalez,R.L. (2008) Coupling of ribosomal L1 stalk and tRNA dynamics during translation elongation. *Mol Cell*, **30**, 348–359.

45. Trabuco,L.G., Schreiner,E., Eargle,J., Cornish,P., Ha,T., Luthey-Schulten,Z. and Schulten,K. (2010) The role of L1 stalk-tRNA interaction in the ribosome elongation cycle. *J Mol Biol*, **402**, 741–760.

46. Dunkle,J.A., Wang,L., Feldman,M.B., Pulk,A., Chen,V.B., Kapral,G.J., Noeske,J., Richardson,J.S., Blanchard,S.C. and Cate,J.H.D. (2011) Structures of the bacterial ribosome in classical and hybrid states of tRNA binding. *Science*, **332**, 981–984.

47. Tourigny,D.S., Fernández,I.S., Kelley,A.C. and Ramakrishnan,V. (2013) Elongation factor G bound to the ribosome in an intermediate state of translocation. *Science*, **340**, 1235490.

48. Zhou,J., Lancaster,L., Donohue,J.P. and Noller,H.F. (2013) Crystal structures of EF-G-ribosome complexes trapped in intermediate states of translocation. *Science*, **340**, 1236086.

49. Korostelev,A., Trakhanov,S., Laurberg,M. and Noller,H.F. (2006) Crystal structure of a 70S ribosome-tRNA complex reveals functional interactions and rearrangements. *Cell*, **126**, 1065–1077.

50. Paci,M. and Fox,G.E. (2015) Major centers of motion in the large ribosomal RNAs. *Nucleic Acids Research*, **43**, 4640–4649.

51. Paci,M. and Fox,G.E. (2016) Centers of motion associated with EF-Tu binding to the ribosome. 10.1080/15476286.2015.1114204.

52. Borovinskaya,M.A., Shoji,S., Holton,J.M., Fredrick,K. and Cate,J.H.D. (2007) A steric block in translation caused by the antibiotic spectinomycin. *ACS Chem Biol*, **2**, 545–552.

53. Carter,A.P., Clemons,W.M., Brodersen,D.E., Morgan-Warren,R.J., Wimberly,B.T. and Ramakrishnan,V. (2000) Functional insights from the structure of the 30S ribosomal subunit and its interactions with antibiotics. *Nature*, **407**, 340–348.

54. Moazed,D. and Noller,H.F. (1987) Interaction of antibiotics with functional sites in 16S ribosomal RNA. *Nature*, **327**, 389–394.

55. Peske,F., Savelsbergh,A., Katunin,V.I., Rodnina,M.V. and Wintermeyer,W. (2004) Conformational changes of the small ribosomal subunit during elongation factor G-dependent tRNA-mRNA translocation. *J Mol Biol*, **343**, 1183–1194.

56. Timsit,Y., Acosta,Z., Allemand,F., Chiaruttini,C. and Springer,M. (2009) The role of disordered ribosomal protein extensions in the early steps of eubacterial 50 S ribosomal subunit assembly. *Int J Mol Sci*, **10**, 817–834.

57. Timsit,Y., Allemand,F., Chiaruttini,C. and Springer,M. (2006) Coexistence of two protein folding states in the crystal structure of ribosomal protein L20. *EMBO Rep.*, **7**, 1013–1018.

58. Wilson,D.N. and Nierhaus,K.H. (2005) Ribosomal proteins in the spotlight. *Crit. Rev. Biochem. Mol. Biol.*, **40**, 243–267.

59. Klein,D.J., Moore,P.B. and Steitz,T.A. (2004) The roles of ribosomal proteins in the structure assembly, and evolution of the large ribosomal subunit. *J. Mol. Biol.*, **340**, 141–177.

60. Brodersen,D.E., Clemons,W.M., Carter,A.P., Wimberly,B.T. and Ramakrishnan,V. (2002) Crystal structure of the 30 S ribosomal subunit from Thermus thermophilus: structure of the proteins and their interactions with 16 S RNA. *J. Mol. Biol.*, **316**, 725–768.

61. Peng,Z., Oldfield,C.J., Xue,B., Mizianty,M.J., Dunker,A.K., Kurgan,L. and Uversky,V.N. (2014) A creature with a hundred waggly tails: intrinsically disordered proteins in the ribosome. *Cell. Mol. Life Sci.*, **71**, 1477–1504.

62. Klinge,S., Voigts-Hoffmann,F., Leibundgut,M. and Ban,N. (2012) Atomic structures of the eukaryotic ribosome. *Trends Biochem. Sci.*, **37**, 189–198.

63. Melnikov,S., Ben-Shem,A., Garreau de Loubresse,N., Jenner,L., Yusupova,G. and Yusupov,M. (2012) One core, two shells: bacterial and eukaryotic ribosomes. *Nat. Struct. Mol. Biol.*, **19**, 560–567.

64. Poirot,O. and Timsit,Y. (2016) Neuron-Like Networks Between Ribosomal Proteins Within the Ribosome. *Sci Rep*, **6**, 26485.

65. Strogatz,S.H. (2001) Exploring complex networks. *Nature*, **410**, 268–276.

66. Barabási,A.-L. and Oltvai,Z.N. (2004) Network biology: understanding the cell's functional organization. *Nat. Rev. Genet.*, **5**, 101–113.

67. Mason,O. and Verwoerd,M. (2007) Graph theory and networks in Biology. *IET Syst Biol*, **1**, 89–119.

68. Shajani,Z., Sykes,M.T. and Williamson,J.R. (2011) Assembly of bacterial ribosomes. *Annu. Rev. Biochem.*, **80**, 501–526.

69. Konikkat,S. and Woolford,J.L. (2017) Principles of 60S ribosomal subunit assembly emerging from recent studies in yeast. *Biochem. J.*, **474**, 195–214.

70. Peña,C., Hurt,E. and Panse,V.G. (2017) Eukaryotic ribosome assembly, transport and quality control. *Nat. Struct. Mol. Biol.*, **24**, 689–699.

71. Woese,C. (1998) The universal ancestor. *Proc. Natl. Acad. Sci. U.S.A.*, **95**, 6854–6859.

72. Forterre,P. (2015) The universal tree of life: an update. *Front Microbiol*, **6**, 717.

73. Hug,L.A., Baker,B.J., Anantharaman,K., Brown,C.T., Probst,A.J., Castelle,C.J., Butterfield,C.N., Hernsdorf,A.W., Amano,Y., Ise,K., *et al.* (2016) A new view of the tree of life. *Nat Microbiol*, **1**, 16048.

74. Pazos,F. and Valencia,A. (2008) Protein co-evolution, co-adaptation and interactions. *EMBO J.*, **27**, 2648–2655.

75. Lovell,S.C. and Robertson,D.L. (2010) An integrated view of molecular coevolution in protein-protein interactions. *Mol. Biol. Evol.*, **27**, 2567–2575.

76. Hormozdiari,F., Salari,R., Hsing,M., Schönhuth,A., Chan,S.K., Sahinalp,S.C. and Cherkasov,A. (2009) The effect of insertions and deletions on wirings in protein-protein interaction networks: a large-scale study. *J. Comput. Biol.*, **16**, 159–167.

77. Polacek,N. and Mankin,A.S. (2005) The ribosomal peptidyl transferase center: structure, function, evolution, inhibition. *Crit. Rev. Biochem. Mol. Biol.*, **40**, 285–311.

78. David-Eden,H. and Mandel-Gutfreund,Y. (2008) Revealing unique properties of the ribosome using a network based analysis. *Nucleic Acids Res.*, **36**, 4641–4652.

79. Rakauskaite,R. and Dinman,J.D. (2008) rRNA mutants in the yeast peptidyltransferase center reveal allosteric information networks and mechanisms of drug resistance. *Nucleic Acids Res.*, **36**, 1497–1507.

80. Makarova,T.M. and Bogdanov,A.A. (2017) The Ribosome as an Allosterically Regulated Molecular Machine. *Biochemistry Mosc.*, **82**, 1557–1571.

81. Sulima,S.O., Gülay,S.P., Anjos,M., Patchett,S., Meskauskas,A., Johnson,A.W. and Dinman,J.D. (2014) Eukaryotic rpL10 drives ribosomal rotation. *Nucleic Acids Res.*, **42**, 2049–2063.

82. Timsit,Y. and Bennequin,D. (2019) Nervous-Like Circuits in the Ribosome Facts, Hypotheses and Perspectives. *Int J Mol Sci*, **20**.

83. Voorhees,R.M. and Ramakrishnan,V. (2013) Structural basis of the translational elongation cycle. *Annu. Rev. Biochem.*, **82**, 203–236.

84. Zaher,H.S. and Green,R. (2009) Fidelity at the molecular level: lessons from protein synthesis. *Cell*, **136**, 746–762.

85. Meskauskas,A. and Dinman,J.D. (2010) A molecular clamp ensures allosteric coordination of peptidyltransfer and ligand binding to the ribosomal A-site. *Nucleic Acids Res.*, **38**, 7800–7813.

86. Pechmann,S., Willmund,F. and Frydman,J. (2013) The ribosome as a hub for protein quality control. *Mol. Cell*, **49**, 411–421.

87. Wilson,D.N., Arenz,S. and Beckmann,R. (2016) Translation regulation via nascent polypeptide-mediated ribosome stalling. *Curr. Opin. Struct. Biol.*, **37**, 123–133.

88. Korostelev,A., Ermolenko,D.N. and Noller,H.F. (2008) Structural dynamics of the ribosome. *Curr Opin Chem Biol*, **12**, 674–683.

89. Gulay,S.P., Bista,S., Varshney,A., Kirmizialtin,S., Sanbonmatsu,K.Y. and Dinman,J.D. (2017) Tracking fluctuation hotspots on the yeast ribosome through the elongation cycle. *Nucleic Acids Res.*, **45**, 4958–4971.

90. Fei,J., Kosuri,P., MacDougall,D.D. and Gonzalez,R.L. (2008) Coupling of ribosomal L1 stalk and tRNA dynamics during translation elongation. *Mol. Cell*, **30**, 348–359.

91. Fei,J., Bronson,J.E., Hofman,J.M., Srinivas,R.L., Wiggins,C.H. and Gonzalez,R.L. (2009) Allosteric collaboration between elongation factor G and the ribosomal L1 stalk directs tRNA movements during translation. *Proc. Natl. Acad. Sci. U.S.A.*, **106**, 15702–15707.

92. Fujii,K., Susanto,T.T., Saurabh,S. and Barna,M. (2018) Decoding the Function of Expansion Segments in Ribosomes. *Mol. Cell*, **72**, 1013-1020.e6.

93. Nissen,P., Hansen,J., Ban,N., Moore,P.B. and Steitz,T.A. (2000) The structural basis of ribosome activity in peptide bond synthesis. *Science*, **289**, 920–930.

94. Kaczanowska,M. and Rydén-Aulin,M. (2007) Ribosome biogenesis and the translation process in Escherichia coli. *Microbiol. Mol. Biol. Rev.*, **71**, 477–494.

95. Gamalinda,M. and Woolford,J.L. (2015) Paradigms of ribosome synthesis: Lessons learned from ribosomal proteins. *Translation (Austin)*, **3**, e975018.

96. Ghosh,A. and Komar,A.A. (2015) Eukaryote-specific extensions in ribosomal proteins of the small subunit: Structure and function. *Translation (Austin)*, **3**, e999576.

97. Abeysirigunawardena,S.C., Kim,H., Lai,J., Ragunathan,K., Rappé,M.C., Luthey-Schulten,Z., Ha,T. and Woodson,S.A. (2017) Evolution of protein-coupled RNA dynamics during hierarchical assembly of ribosomal complexes. *Nat Commun*, **8**, 492.

98. Mallik,S., Akashi,H. and Kundu,S. (2015) Assembly constraints drive co-evolution among ribosomal constituents. *Nucleic Acids Res.*, **43**, 5352–5363.

99. Calidas,D., Lyon,H. and Culver,G.M. (2014) The N-terminal extension of S12 influences small ribosomal subunit assembly in Escherichia coli. *RNA*, **20**, 321–330.

100. Tutuncuoglu,B., Jakovljevic,J., Wu,S., Gao,N. and Woolford,J.L. (2016) The N-terminal extension of yeast ribosomal protein L8 is involved in two major remodeling events during late nuclear stages of 60S ribosomal subunit assembly. *RNA*, **22**, 1386–1399.

101. Galkin,O., Bentley,A.A., Gupta,S., Compton,B.-A., Mazumder,B., Kinzy,T.G., Merrick,W.C., Hatzoglou,M., Pestova,T.V., Hellen,C.U.T., *et al.* (2007) Roles of the negatively charged N-terminal extension of Saccharomyces cerevisiae ribosomal protein S5 revealed by characterization of a yeast strain containing human ribosomal protein S5. *RNA*, **13**, 2116–2128.

102. Voorhees,R.M., Weixlbaumer,A., Loakes,D., Kelley,A.C. and Ramakrishnan,V. (2009) Insights into substrate stabilization from snapshots of the peptidyl transferase center of the intact 70S ribosome. *Nat. Struct. Mol. Biol.*, **16**, 528–533.

103. Fernández-Pevida,A., Martín-Villanueva,S., Murat,G., Lacombe,T., Kressler,D. and de la Cruz,J. (2016) The eukaryote-specific N-terminal extension of ribosomal protein S31 contributes to the assembly and function of 40S ribosomal subunits. *Nucleic Acids Res.*, **44**, 7777–7791.

104. Lawrence,M.G., Shamsuzzaman,M., Kondopaka,M., Pascual,C., Zengel,J.M. and Lindahl,L. (2016) The extended loops of ribosomal proteins uL4 and uL22 of Escherichia coli contribute to ribosome assembly and protein translation. *Nucleic Acids Res.*, **44**, 5798–5810.

105. Meskauskas,A., Harger,J.W., Jacobs,K.L.M. and Dinman,J.D. (2003) Decreased peptidyltransferase activity correlates with increased programmed -1 ribosomal frameshifting and viral maintenance defects in the yeast Saccharomyces cerevisiae. *RNA*, **9**, 982–992.

106. Meskauskas,A., Russ,J.R. and Dinman,J.D. (2008) Structure/function analysis of yeast ribosomal protein L2. *Nucleic Acids Res.*, **36**, 1826–1835.

107. Rhodin,M.H.J. and Dinman,J.D. (2010) A flexible loop in yeast ribosomal protein L11 coordinates P-site tRNA binding. *Nucleic Acids Res.*, **38**, 8377–8389.

108. Rhodin,M.H.J. and Dinman,J.D. (2011) An extensive network of information flow through the B1b/c intersubunit bridge of the yeast ribosome. *PLoS ONE*, **6**, e20048.

109. Bowen,A.M., Musalgaonkar,S., Moomau,C.A., Gulay,S.P., Mirvis,M. and Dinman,J.D. (2015) Ribosomal protein uS19 mutants reveal its role in coordinating ribosome structure and function. *Translation (Austin)*, **3**, e1117703.

110. Kisly,I., Gulay,S.P., Mäeorg,U., Dinman,J.D., Remme,J. and Tamm,T. (2016) The Functional Role of eL19 and eB12 Intersubunit Bridge in the Eukaryotic Ribosome. *J. Mol. Biol.*, **428**, 2203–2216.

111. Mailliot,J., Garreau de Loubresse,N., Yusupova,G., Meskauskas,A., Dinman,J.D. and Yusupov,M. (2016) Crystal Structures of the uL3 Mutant Ribosome: Illustration of the Importance of Ribosomal Proteins for Translation Efficiency. *J. Mol. Biol.*, **428**, 2195–2202.

112. Jindal,S., Ghosh,A., Ismail,A., Singh,N. and Komar,A.A. (2019) Role of the uS9/yS16 C-terminal tail in translation initiation and elongation in Saccharomyces cerevisiae. *Nucleic Acids Res.*, **47**, 806–823.

113. Singh,N., Jindal,S., Ghosh,A. and Komar,A.A. (2019) Communication between RACK1/Asc1 and uS3 (Rps3) is essential for RACK1/Asc1 function in yeast Saccharomyces cerevisiae. *Gene*, **706**, 69–76.

114. Chan,Y.-L., Dresios,J. and Wool,I.G. (2006) A pathway for the transmission of allosteric signals in the ribosome through a network of RNA tertiary interactions. *J. Mol. Biol.*, **355**, 1014–1025.

115. Guzel,P. and Kurkcuoglu,O. (2017) Identification of potential allosteric communication pathways between functional sites of the bacterial ribosome by graph and elastic network models. *Biochim Biophys Acta Gen Subj*, **1861**, 3131–3141.

116. Wohlgemuth,I., Pohl,C., Mittelstaet,J., Konevega,A.L. and Rodnina,M.V. (2011) Evolutionary optimization of speed and accuracy of decoding on the ribosome. *Philos. Trans. R. Soc. Lond., B, Biol. Sci.*, **366**, 2979–2986.

117. Balchin,D., Hayer-Hartl,M. and Hartl,F.U. (2016) In vivo aspects of protein folding and quality control. *Science*, **353**, aac4354.

118. Joshi,K., Cao,L. and Farabaugh,P.J. (2019) The problem of genetic code misreading during protein synthesis. *Yeast*, **36**, 35–42.

119. Dinman,J.D. (2016) Pathways to Specialized Ribosomes: The Brussels Lecture. *J. Mol. Biol.*, **428**, 2186–2194.

120. Genuth,N.R. and Barna,M. (2018) The Discovery of Ribosome Heterogeneity and Its Implications for Gene Regulation and Organismal Life. *Mol. Cell*, **71**, 364–374.

121. Sulima,S.O., Hofman,I.J.F., De Keersmaecker,K. and Dinman,J.D. (2017) How Ribosomes Translate Cancer. *Cancer Discov*, **7**, 1069–1087.

122. Timsit,Y., Sergeant-Perthuis,G. and Bennequin,D. (2021) Evolution of ribosomal protein network architectures. 10.1038/s41598-020-80194-4.

123. Poirot,O. and Timsit,Y. (2016) Neuron-Like Networks Between Ribosomal Proteins Within the Ribosome. 10.1038/srep26485.

124. Timsit,Y. and Bennequin,D. (2019) Nervous-Like Circuits in the Ribosome Facts, Hypotheses and Perspectives. 10.3390/ijms20122911.

125. Nussinov,R., Tsai,C.-J. and Liu,J. (2014) Principles of allosteric interactions in cell signaling. 10.1021/ja510028c.

126. del Sol,A., Tsai,C.-J., Ma,B. and Nussinov,R. (2009) The origin of allosteric functional modulation: multiple pre-existing pathways. *Structure*, **17**, 1042–1050.

127. Arantes,P.R., Patel,A.C. and Palermo,G. (2022) Emerging Methods and Applications to Decrypt Allostery in Proteins and Nucleic Acids. *Journal of Molecular Biology*, **434**, 167518.

128. Nechushtai,R., Lammert,H., Michaeli,D., Eisenberg-Domovich,Y., Zuris,J.A., Luca,M.A., Capraro,D.T., Fish,A., Shimshon,O., Roy,M., *et al.* (2011) Allostery in the ferredoxin protein motif does not involve a conformational switch. *Proceedings of the National Academy of Sciences*, **108**, 2240–2245.

129. Sebban,P., Maróti,P., Schiffer,M. and Hanson,D.K. (1995) Electrostatic dominoes: long distance propagation of mutational effects in photosynthetic reaction centers of Rhodobacter capsulatus. *Biochemistry*, **34**, 8390–8397.

130. Timsit,Y., Allemand,F., Chiaruttini,C. and Springer,M. (2006) Coexistence of two protein folding states in the crystal structure of ribosomal protein L20. 10.1038/sj.embor.7400803.

131. Sato,T., Ohnuki,J. and Takano,M. (2016) Dielectric Allostery of Protein: Response of Myosin to ATP Binding. 10.1021/acs.jpcb.6b10003.

132. Sato,T., Ohnuki,J. and Takano,M. (2017) Long-range coupling between ATP-binding and lever-arm regions in myosin via dielectric allostery. 10.1063/1.5004809.

133. Banerjee-Ghosh,K., Ghosh,S., Mazal,H., Riven,I., Haran,G. and Naaman,R. (2020) Long-Range Charge Reorganization as an Allosteric Control Signal in Proteins. *J Am Chem Soc*, **142**, 20456–20462.

134. Ghosh,S., Banerjee-Ghosh,K., Levy,D., Scheerer,D., Riven,I., Shin,J., Gray,H.B., Naaman,R. and Haran,G. (2022) Control of protein activity by photoinduced spin polarized charge reorganization. *Proc Natl Acad Sci U S A*, **119**, e2204735119.

135. Dempsey,J.L., Winkler,J.R. and Gray,H.B. (2010) Proton-Coupled Electron Flow in Protein Redox Machines. 10.1021/cr100182b.

136. Crack,J.C., Amara,P., Volbeda,A., Mouesca,J.-M., Rohac,R., Pellicer Martinez,M.T., Huang,C.-Y., Gigarel,O., Rinaldi,C., Le Brun,N.E., *et al.* (2020) Electron and Proton Transfers Modulate DNA Binding by the Transcription Regulator RsrR. 10.1021/jacs.9b12250.

137. Corbella,M., Voityuk,A.A. and Curutchet,C. (2015) Single Amino Acid Mutation Controls Hole Transfer Dynamics in DNA-Methyltransferase HhaI Complexes. 10.1021/acs.jpclett.5b01683.

138. Corbella,M., Voityuk,A.A. and Curutchet,C. (2018) How abasic sites impact hole transfer dynamics in GC-rich DNA sequences. 10.1039/c8cp03572e.

139. Sosorev,A.Y. (2021) Walking around Ribosomal Small Subunit: A Possible 'Tourist Map' for Electron Holes. 10.3390/molecules26185479.

140. Sosorev,A. and Kharlanov,O. (2021) Organic nanoelectronics inside us: charge transport and localization in RNA could orchestrate ribosome operation. 10.1039/D0CP04970K.

141. Wang,P., Leontyev,I. and Stuchebrukhov,A.A. (2022) Mechanical Allosteric Couplings of Redox-Induced Conformational Changes in Respiratory Complex I. *J. Phys. Chem. B*, **126**, 4080–4088.

142. Isom,D.G., Sridharan,V., Baker,R., Clement,S.T., Smalley,D.M. and Dohlman,H.G. (2013) Protons as second messenger regulators of G protein signaling. 10.1016/j.molcel.2013.07.012.

143. Kapolka,N.J., Rowe,J.B., Taghon,G.J., Morgan,W.M., O'Shea,C.R. and Isom,D.G. (2021) Proton-gated coincidence detection is a common feature of GPCR signaling. 10.1073/pnas.2100171118.

144. Harris,R.C., Tsai,C.-C., Ellis,C.R. and Shen,J. (2017) Proton-Coupled Conformational Allostery Modulates the Inhibitor Selectivity for β-Secretase. *J Phys Chem Lett*, **8**, 4832–4837.

145. Tsai,C.-C., Yue,Z. and Shen,J. (2019) How Electrostatic Coupling Enables Conformational Plasticity in a Tyrosine Kinase. *J Am Chem Soc*, **141**, 15092–15101.

146. Ghode,A., Gross,L.Z.F., Tee,W.-V., Guarnera,E., Berezovsky,I.N., Biondi,R.M. and Anand,G.S. (2020) Synergistic Allostery in Multiligand-Protein Interactions. 10.1016/j.bpj.2020.09.019.

147. Webby,C.J., Jiao,W., Hutton,R.D., Blackmore,N.J., Baker,H.M., Baker,E.N., Jameson,G.B. and Parker,E.J. (2010) Synergistic allostery, a sophisticated regulatory network for the control of aromatic amino acid biosynthesis in Mycobacterium tuberculosis. 10.1074/jbc.M110.111856.

148. Jiao,W., Hutton,R.D., Cross,P.J., Jameson,G.B. and Parker,E.J. (2012) Dynamic cross-talk among remote binding sites: the molecular basis for unusual synergistic allostery. 10.1016/j.jmb.2011.11.037.

149. Shin,H., Ren,Z., Zeng,X., Bandara,S. and Yang,X. (2019) Structural basis of molecular logic OR in a dual-sensor histidine kinase. 10.1073/pnas.1910855116.

150. Timsit,Y. and Grégoire,S.-P. (2021) Towards the Idea of Molecular Brains. 10.3390/ijms222111868.

151. Timsit,Y. and Moras,D. (1992) Crystallization of DNA. *Methods Enzymol*, **211**, 409–429.

152. Timsit,Y. and Moras,D. (1995) Self-fitting and self-modifying properties of the B-DNA molecule. *J Mol Biol*, **251**, 629–647.

153. Timsit,Y. (2012) DNA-directed base pair opening. *Molecules*, **17**, 11947–11964.

154. Várnai,P. and Timsit,Y. (2010) Differential stability of DNA crossovers in solution mediated by divalent cations. 10.1093/nar/gkq150.

155. Timsit,Y., Westhof,E., Fuchs,R.P. and Moras,D. (1989) Unusual helical packing in crystals of DNA bearing a mutation hot spot. 10.1038/341459a0.

156. Timsit,Y. and Moras,D. (1996) Cruciform structures and functions. *Q Rev Biophys*, **29**, 279–307.

157. Song,J., Rechkoblit,O., Bestor,T.H. and Patel,D.J. (2011) Structure of DNMT1-DNA complex reveals a role for autoinhibition in maintenance DNA methylation. *Science*, **331**, 1036–1040.

158. Jeltsch,A. and Jurkowska,R.Z. (2016) Allosteric control of mammalian DNA methyltransferases - a new regulatory paradigm. *Nucleic Acids Res*, **44**, 8556–8575.

159. Panja,S., Santiago-Frangos,A., Schu,D.J., Gottesman,S. and Woodson,S.A. (2015) Acidic Residues in the Hfq Chaperone Increase the Selectivity of sRNA Binding and Annealing. *J Mol Biol*, **427**, 3491–3500.

160. Hossain,K.A., Kogut,M., Słabońska,J., Sappati,S., Wieczór,M. and Czub,J. (2023) How acidic amino acid residues facilitate DNA target site selection. *Proceedings of the National Academy of Sciences*, **120**, e2212501120.

161. Chou,C.-C. and Wang,A.H.-J. (2015) Structural D/E-rich repeats play multiple roles especially in gene regulation through DNA/RNA mimicry. 10.1039/c5mb00206k.

162. Zaharias,S., Zhang,Z., Davis,K., Fargason,T., Cashman,D., Yu,T. and Zhang,J. (2021) Intrinsically disordered electronegative clusters improve stability and binding specificity of RNA-binding proteins. 10.1016/j.jbc.2021.100945.

163. Warshel,A., Sharma,P.K., Kato,M. and Parson,W.W. (2006) Modeling electrostatic effects in proteins. *Biochim Biophys Acta*, **1764**, 1647–1676.

164. Gitlin,I., Carbeck,J.D. and Whitesides,G.M. (2006) Why are proteins charged? Networks of charge-charge interactions in proteins measured by charge ladders and capillary electrophoresis. *Angew Chem Int Ed Engl*, **45**, 3022–3060.

165. Peng,Y. and Alexov,E. (2017) Computational investigation of proton transfer, pKa shifts and pH-optimum of protein-DNA and protein-RNA complexes. 10.1002/prot.25221.

166. Pahari,S., Sun,L. and Alexov,E. (2019) PKAD: a database of experimentally measured pKa values of ionizable groups in proteins. 10.1093/database/baz024.

167. Onufriev,A.V. and Alexov,E. (2013) Protonation and pK changes in protein-ligand binding. *Q Rev Biophys*, **46**, 181–209.

168. Harris,T.K. and Turner,G.J. (2002) Structural basis of perturbed pKa values of catalytic groups in enzyme active sites. *IUBMB Life*, **53**, 85–98.

169. Grimsley,G.R., Scholtz,J.M. and Pace,C.N. (2009) A summary of the measured pK values of the ionizable groups in folded proteins. *Protein Sci*, **18**, 247–251.

170. Moutevelis,E. and Warwicker,J. (2004) Prediction of pKa and redox properties in the thioredoxin superfamily. *Protein Sci*, **13**, 2744–2752.

171. Cruz-Gallardo,I., Del Conte,R., Velázquez-Campoy,A., García-Mauriño,S.M. and Díaz-Moreno,I. (2015) A Non-Invasive NMR Method Based on Histidine Imidazoles to Analyze the pH-Modulation of Protein-Nucleic Acid Interfaces. 10.1002/chem.201405538.

172. Liao,S.-M., Du,Q.-S., Meng,J.-Z., Pang,Z.-W. and Huang,R.-B. (2013) The multiple roles of histidine in protein interactions. *Chemistry Central Journal*, **7**, 44.

173. Baran,K.L., Chimenti,M.S., Schlessman,J.L., Fitch,C.A., Herbst,K.J. and Garcia-Moreno,B.E. (2008) Electrostatic effects in a network of polar and ionizable groups in staphylococcal nuclease. *J Mol Biol*, **379**, 1045–1062.

174. Di Russo,N.V., Estrin,D.A., Martí,M.A. and Roitberg,A.E. (2012) pH-Dependent conformational changes in proteins and their effect on experimental pK(a)s: the case of Nitrophorin 4. *PLoS Comput Biol*, **8**, e1002761.

175. Zhang,H., Eerland,J., Horn,V., Schellevis,R. and van Ingen,H. (2021) Mapping the electrostatic potential of the nucleosome acidic patch. *Sci Rep*, **11**, 23013.

176. Karp,D.A., Stahley,M.R. and García-Moreno,B. (2010) Conformational consequences of ionization of Lys, Asp, and Glu buried at position 66 in staphylococcal nuclease. *Biochemistry*, **49**, 4138–4146.

177. Fitch,C.A., Karp,D.A., Lee,K.K., Stites,W.E., Lattman,E.E. and García-Moreno E,B. (2002) Experimental pK(a) values of buried residues: analysis with continuum methods and role of water penetration. *Biophys J*, **82**, 3289–3304.

178. Isom,D.G., Castañeda,C.A., Cannon,B.R. and García-Moreno,B. (2011) Large shifts in pKa values of lysine residues buried inside a protein. *Proc Natl Acad Sci U S A*, **108**, 5260–5265.

179. Harms,M.J., Schlessman,J.L., Sue,G.R. and García-Moreno E.,B. (2011) Arginine residues at internal positions in a protein are always charged. *Proceedings of the National Academy of Sciences*, **108**, 18954–18959.

180. Fitch,C.A., Platzer,G., Okon,M., Garcia-Moreno,B.E. and McIntosh,L.P. (2015) Arginine: Its pKa value revisited. *Protein Sci*, **24**, 752–761.

181. Bas,D.C., Rogers,D.M. and Jensen,J.H. (2008) Very fast prediction and rationalization of pKa values for protein-ligand complexes. *Proteins*, **73**, 765–783.

182. Aguilar,B., Anandakrishnan,R., Ruscio,J.Z. and Onufriev,A.V. (2010) Statistics and physical origins of pK and ionization state changes upon protein-ligand binding. *Biophys J*, **98**, 872–880.

183. Deochand,D.K., Pande,A., Meariman,J.K. and Grove,A. (2019) Redox Sensing by PecS from the Plant Pathogen Pectobacterium atrosepticum and Its Effect on Gene Expression and the Conformation of PecS-Bound Promoter DNA. 10.1021/acs.biochem.9b00288.

184. Deochand,D.K., Meariman,J.K. and Grove,A. (2016) pH-Dependent DNA Distortion and Repression of Gene Expression by Pectobacterium atrosepticum PecS. *ACS Chem. Biol.*, **11**, 2049–2056.

Captions to the Supplementary Tables

Table S1. List of the CMs and their bound r-proteins
BC-C: Calculated values of the betweenness centrality of the CM in the network.
EV-C: Calculated values of the eigenvector centrality of the CM in the network.
Deg: Calculated values of the degree (Nb of connections of the node) centrality of the CM in the network.
Base pairs: paired bases of the CM at the hinge point.
Direct: list of the r-proteins and conserved motifs interacting directly with the CM. Indirect: list of the r-proteins and their conserved motifs interacting indirectly with the CM.

Table S2. Cryo-EM structures from Rundlet et al (2021) [48] (main text) used to extract rRNA-r-protein movements during the early steps of translocation.

Table S3. List of the interactions between r-protein, CMs and functional sites in the network in the SSU (A) and the LSU (B) according to distance criteria. The r-protein-functional centre interactions have been described in details in previous works [100-103] (main text)

Table S4. Sum of the phosphorus atom displacements of the nucleotides located in a radius of < 15 Å around each r-protein, at step 2-6, relatively to step 1.
Nb= nb of nucleotide at a distance of 15 Å of the protein. Σd = sum of displacement of P around the protein. Σd pond = Σd/Nb.

Table S5. Sum of all the phosphorus atom displacements during steps 2-6, relatively to step 1. (A) SSU. (B) LSU. This Sum provides an estimate of the total quantity of movement of a given nucleotide located in a radius of < 15 Å around each Glu or Asp residues of r-proteins. Σdispl : sum of all the P-P displacements measured from steps 2 to step 6, relatively to step 1. MinDist: minimum distance between the Calpha of the Glu or Asp and the phosphorus atom of the set nucleotides within a sphere of 15 Å around them. Nb_nucl: number of nucleotides within a sphere of 15 Å around each Glu or Asp Residue. Av_displ: Σdispl /Nb_nucl.

Captions to the Supplementary Figures

Figure S1. Summary of the 6 steps of the translocation observed in the time-resolved cryo-electromicroscopic study of Rundlet etal (2021) [48] (main text). For each translocation step, the structure of the SSU and LSU are represented in cartoon and the position of mRNA, tRNA and factors are represented by coloured surfaces.

Figure S2. Summary of the main motions of the bacterial ribosome observed during the translocation including the translocation of the tRNAs from the A-site to the E-site, the subunit rotation between the SSU and the LSU, the head/body swivelling in the SSU and the stalk motions in the LSU.
The LSU in the classic unrotated state is represented with a white surface (PDB_id: 7n1p). The subunit rotation (the counter clockwise rotation of the 30S subunit relatively to the 50S subunit) is illustrated by a set of cartoons of the SSU during the 6 steps of the

translocation described in the study of Rundlet et al. (2021) [48] with different colours. The colour code is maintained throughout the manuscript: white: step 1 (7n1p); light blue: step 2 (7n30); slate: step 3 (7n2u); blue: step 4 (7n2v); deep blue: step 5 (7n2c) and wheat: step 6 (7n31). Top right. Superimposition of the SSU observed in the 6 steps to highlight the swivelling motion. The direction of the translocation is represented by red arrows.

Figure S3. Localisation of the major centre of motions (CMs) in the 2D maps of the 16S (A) and 23S rRNA (B) and their corresponding locations in the ribosome 3D structures. Stereo views of the CM (red spheres) in the SSU (C-E) and the LSU (F-G). This figure is a synthesis of the works described in [52-55] (main text). The red lines within the 2D rRNA maps indicate the moving helices (adapted from the figures of Paci and Fox, 2015 [52] (main text).

Figure S4. Structural properties of the major CMs of the bacterial ribosomes. They contribute to form flexible zones in the rRNA. The base pair of the hinge is generally a weak non-canonical base pair. The CMs are grouped into clusters (Head: p1247, p1351 and p1304) (Body: p62, p149 and p201) and often observed in 3-way junctions (A-C) [59] (main text), they form special tertiary RNA-RNA interactions (D-H). p1247 and p1351 are involved in unusual RNA-RNA contacts such as the stacking of guanine G1371 on the edge of 3 adenines (A1287-A1289). The CMs are represented by red spheres.

Figure S5. Functional properties of CMs
(A-F) Localisation of the CMs of the bacterial ribosomes at the interfaces of subdomains of the SSU. The rainbow display (blue 5'- red 3') of the rRNA chain highlights the distinct domains of the 16S rRNA (PDB_id : 4v9h). (G-H) CMs are located close to ribosome functional centres. (G) Stereo view of the EF-G binding site, close to CM p441. (H) stereo view of codon-anticodon pairing in the vicinity of the uS9, uS13, uS5 proteins and the CM p1394-p1396-p1402. (PDB_id : 4v9h). The CMs are represented by spheres and r-proteins by ribbons.

Figure S6. Localisation of the CMs in the context of the dynamic of the subunit during the translocation. The CMs represented by red spheres are shown the superimposed structures of each subunit, of the 6 steps of the translocation (same colour code as in Figure 1 and Supplementary Figure S2). The CMs are observed at the base of moving stalks and helices in the LSU. They play a more complex and collective role in the SSU (described in details in figure S7).

Figure S7. Complex internal motions of the head during swivelling. The rRNA of the head (residues 932-1385) has been superimposed to highlight ist internal motion and rearrangements during the swivelling. The ribosome structures compared are: *T. thermophilus* (PDB_id : 4v9h and 4v5f) orange and grey, respectively and *E. coli* ribosomes : 7n1p, 7n30, 7n2u, 7n2v, 7n2c, 7n31.

Figure S8. Summary of the main properties of r-protein networks.
(A) The r-proteins of the ribosome of *T. thermophilus* (PDB_id : 4v9h) are represented by cartoon, the r-protein interactions are represented by surfaces. The rRNA is omitted to highlight the tiny interactions between the extensions. (B) Surface representation of the interactions between r-proteins showing the alternate aromatic (yellow) and basic (blue) residues. (C and D) Focus on a tiny interaction between the extension of uL3 and uL13

showing the conserved residues at the interface. (E and F) Examples of interspecies variations in network connectivity. In *E. coli*, uS14 has an elongated structure that forms an additional contact with uS19 (7n1p1). In *M. smegmatis,* b16 has an additional extension that contact uS4 (PDB id: 5o61).

Figure S9. Evolution of r-protein networks.
(A and B) Congruent growing number of extensions in r-protein and contact in the networks during the evolution (ABE=inferred universal network; B=bacterial, AE= archaeal, E=eukaryotic r-protein network. C. Schematic tree summarizing the evolution of the r-protein network highlighting the growing connectivity between the functional modules in passing from bacteria to eukarya.

Figure S10. Interactions between the major CMs of the bacterial ribosome and r-proteins.
(A) CMs are represented by red spheres and r-proteins by blue cartoons. The coordinates of *T. thermophilus* (PDB_id : 4v9h) have been used to detect the rRNA-r-proteins around the hinges according distance criteria. (B and C) The interactions CMs-r-proteins are shown in the rRNA 2D maps of the SSU and the LSU, respectively.

Figure S11. Phylogenetically conserved r-protein motifs interacting with the CMs. Strictly conserved residues are represented by red stick. Similar residues (polar, hydrophobic, aromatic and charged) are represented by orange sticks (PDB_id : 4v9h).

Figure S12. Conserved acidic residues at the interface of r-protein interactions
Stereo views of (A) uL2-pL1298 (B) uL2-pL703 (C) uL16-pL871. The conserved acidic residues are represented by red spheres and mRNA by magenta sticks (PDB_id : 4v9h).

Figure S13. r-protein - CMs network
(A) Schematic representation of the network formed by r-proteins (circles), CMs (rectangles) and functional centres (tRNA: blue, PTC: orange, Tunnel: magenta). The r-proteins that interact directly with the CMs are coloured in pink, those that interact indirectly (within one helix turn) are represented in cyan. The CMs contacted directly by r-proteins are represented in pink, the CMs contacted indirectly by r-protein are represented in cyan. (B) Stereo view of the r-protein network in the *E. coli* ribosome (7n1p) with the same colour code as in (A).

Figure S14. Focus and 3D representations of network regions involved in key ribosome movements during translocation. CMs are represented by red spheres and r-proteins by cartoons.

Figure S15. Global stereo view of the CMs-r-proteins in the body (A) and close-up of CM p593 located opposite the interaction of the triad uS8, uS12 and uS17 (B).

Figure S16. Graph theory and r-protein-CM networks.
The connected spheres correspond to the centres of mass of each node. R-proteins are depicted in grey, functional centres in yellow spheres and CMs in red. The diameters of the spheres are proportional to the centrality values of each node. (A) Betweenness centrality (B) EigenVector centrality. The centrality values of each node are also represented by histogram with the same colour code. The Betweenness centrality measures how many central paths pass through the node and is defined as $C^B(u) =$ [illegible] $\sigma_{v,v1} (u) / \sigma_{v,v1}$. Eigenvector centrality is a normalized eigenvector of the adjacency matrix of G

with eigenvalue 1, $C^B(u) = x(u)$ where $Ax = x$. Both centralities are computed using the Python package NetworkX [107] (main text).

Figure S17. Other mode of representation of the r-protein-CM networks.
(A) Stereo view of the r-protein represented by cartoons and CMs represented by red spheres (without rRNA) (This is a stereo view of Figure 3C (main text). The centres of mass of each node are connected by thin black lines. (B) Close up on the maxima of EigenVector centrality in the head of the SSU (represented in Figure S16 B).

Figure S18. Spatio temporal map of rRNA-r-protein motions in the LSU. During the early translocation steps of *E. coli* ribosome.
LSU map of 23S RNA-r-protein movements observed at step 2-6, relatively to step 1 (PDB_id: 7n1p). Surface representations of the 16 RNA coloured from white to red according to the measured phosphorus-phosphorus displacements of each nucleotide, from step 1 to step 6, around each r-protein (represented by violet cartoons). (Right): the network of CMs (small yellow spheres) – r-proteins (violet spheres whose diameters are proportional to the sum of rRNA displacements around them (within a distance of 15 Å).

Figure S19. rRNA/r-protein movements around the main CMs of the LSU at step 5 (EF-G bound bound, before GTP hydrolysis). Three modes of representation illustrate the relative movements of the rRNA with respect to the r-protein in the vicinity of the CM at step 5 (7n2c) (EF-G bound, after GTP hydrolysis).
On the left, the rRNA is coloured by a colour gradient from white to red, proportional to the amount of displacement relative to its initial position at step 1 (classic non-rotated state).
The middle section presents a stereo view, which superimposes the area around the CM in step 1 (7n1p, white) and step 5 (7n2c, deep blue). (The protein from step 5 is superimposed on the protein from step 1 to show the relative movement of the RNA around it). CMs are represented by white spheres and r-proteins by purple cartoons.
On the right, a schematic view of the CM-protein interaction and an axial view of the CM base pair are shown.
(A) CM pL1298 at the base of uL1-stalk. (B) CM pL2298 at the base of the central protuberance (C) CM pL1032 at the base of the bL12-stalk. The red arrows indicate the direction of movement from step 1 to 5. (D) CM pL703 at the base of H34 that mediates an interaction with uS15 in the SSU. (E) CM pL871 at the base of H38 that mediates transient interactions with uL5, uS19 and tRNAs.

Figure S20. rRNA/r-protein movements around the main CMs of the SSU at step 5 (EF-G bound, before GTP hydrolysis). Three modes of representation illustrate the relative movements of the rRNA with respect to the r-protein in the vicinity of the CM at step 5.
On the left, the rRNA is coloured by a colour gradient from white to red, proportional to the amount of displacement relative to its initial position at step 1 (classic non-rotated state).
The middle section presents a stereo view, which superimposes the area around the CM in step 1 (7n1p, white) and step 5 (7n2c, deep blue) (The protein from step 5 is superimposed on the protein from step 1 to show the relative movement of the RNA around it). CMs are represented by white spheres and r-proteins by purple cartoons.
On the right, a schematic view of the CM-protein interaction and an axial view of the CM base pair are represented. (A) **CM p1074** corresponding to one of the rotation axis of the head/body swivelling (corresponding roughly to **Hinge 2** of Mohan et al [54] (main text).

(B-E) CMs playing a role in regulating the internal motions of the head and the size of the mRNA channel entry and exit points during swivelling.
(F) **CM p1394** corresponding to the "neck" pivot, or the first rotation axis (**Hinge 1** of Mohan et al. [54]) during head/body. (G-J) CMs involved in the internal reorganisation of the body during swivelling. The red arrows indicate the direction of movement from step 1 to 5.

Figure S21. rRNA/r-protein and r-protein/r-protein movements rearrange the internal structure of the head and modulate the size of the mRNA entry channel.
(A) Movements of rRNA helices of the head around uS3 (fixed). (B) Movements of rRNA helices around uS2 (fixed). The acidic residues are represented by red stick. White cartoons represent the structures of the r-proteins and their rRNA layer of the *E. coli* ribosome at step 1 (7n1p). Light blue: step 2 (7n30); Slate: step 3 (7n2u); Blue: step 4 (7n2v); Deep blue: step 5 (7n2c) and Wheat: step 6 (7n31).
Conserved acidic residues at the interaction surfaces of (C) uS2-uS3 (D) uS3-uS5 and (E) uS4-uS5. The conserved Glu and Asp residues are depicted by red spheres and the other conserved residues are depicted by sticks (*T. thermophilus* ribosome pdb_id 4v9h*)*

Figure S22. rRNA/r-protein and r-protein/r-protein movements rearrange the internal structure of the head and modulate the size of the mRNA exit channel. (A-C) Three stereo views of the rRNA and r-proteins around uS7 (fixed) represented by a cyan surface, during the 6 steps of the translocation (Figure S1 and S2).

Figure S23. rRNA/r-protein and r-protein/r-protein movements rearrange the internal structure of the body. (A, C) and (B) stereo and schematic views of the movements of the rRNA helices around uS17 (fixed) during the 6 translocation steps (same colour code as Figure S21). During the process of translocation, the mobility of rRNA around uS17 results in the division of the body into two distinct blocks. One of these blocks remains fixed, and it is attached to bS16 and bS20. The other block is mobile and contains uS8 and uS15.
(D-F). Three stereo views showing the relationship between uS17 and uS12 located at the opposite faces of the SSU during the translocation. In particular, uS12 and uS17 repel the long helices h44 and h21 during the back-swivelling occurring ate step 6 (7n31) coloured in wheat.

Figure S24. R-protein residues at the vicinity of rRNA-r-protein moving regions in the *E. coli* ribosome. (A-P) LSU. (Q-Y) SSU.
White cartoons represent the structures of the r-proteins and its rRNA layer at step 1 (7n1p). Light blue: step 2 (7n30). Slate: step 3 (7n2u). Blue: step 4 (7n2v). Deep blue: step 5 (7n2c). Wheat: step 6 (7n31). The structures of the r-proteins (at step 1) are represented by cartoons and surfaces. The phosphate groups which have moved more than 1 Å from their position in step 1 are represented by spheres. C-ter-alpha helices are highlighted with red circles.

CM	BC-C	EV-C	Deg	Helix	base pair	Direct			Indirect		
**SSU-head**											
**p955**	0.01	**0.44**	3	H30 (T)	U955-A1225	**uS13-ext C**	**uS19-ext C**		**uS9-ext-C**	US13-uS19	
						R78.104.102-A1225	R78-A1125		R128-G1231		
						Y87/R91-π	basic/cat-π		basic/cat-π		
**p1074**	0.01	**0.46**	3	H36 (L)	G1074-U1083	**uS2-G**	**uS5-G**		**uS3**	uS2-uS8-uS5	
						W97. E176-G1074	cat-π > π G 1074		H176.P174-C1109		
						Nter α-helix>P1074	α-helix // bck		SBA:R179/E206/F203		
						2 aroms F152.105					
						No basic residues	basic > bck				
**p1125**	0.02	**0.54**	3	H39 (L)	U1125-C1149	**uS10-G**	**uS9-G**		**uS3**		
					tetrad	P37.39.41	R9.16(S)>P1149		E206-R179>C1112	uS10-uS9-uS14-uS3	
						D73- R5> P(U 1125)					
						D17-R70>p(A1151)					
						H68-p(A1152)					
						special SYM *					
**p1156**	0.1	**0.77**	5	H40 (L)	A1179-G1156	**uS2-dom**	**uS9**		**uS3**	**uS10**	**uS14**
						K132.133(S)>P(A1159)	K97.R93(B)>P(A1179)		E206-R179>C1208	D17-R70>p(A1151)	W61..C1114
							D105-R10 ...A 1179			H68-p(A1152)	
**p1212**	0.03	**0.23**	2	H34 (T)	U1212 . U992				**uS19**	**uS14**	
									W34. Y52 > G987	α-helix//bck	
										F16R19> U1219	
**p1247**	0.04	**0.58**	4	H41 (L)	U 1247-G 1290	**uS9-G**	**THX**	**uS7**	**uS7**	**uS13**	
						NC res in loop	basic classic	K35(S)>P(G1290)	R115-A1239	K13> U1301	
						F33.Y36 > p (C1249)			R119-E113> U1239	R44-D47>C1296	
**p1304**	0.09	**0.49**	4	H42 (T)	G1304>P(A1332)	**THX**	**uS13-G**		**uS7**	**uS19**	
						D5 > G1304	Y23.R71.E67>U1330		R119-E113> U1239	K6.K7(S)>C1314	
							K13> U1301				
							R44-D47>C1296				
							R88.93.99.110>U1308				
							H92>p (U 1308)				
**p1351**	0.1	**0.73**	5	H43 (T)	U 1351 -G 1371	**uS7**	**uS9**	**THX**	**uS9**	**uS10**	**uS14**
						NC loop. K35>U1351	K11(S)> G1371	K3>P C 1352	E110-R120.	H62.R60.E61> C1367	W61> 1368
							D75.105-R10.107> G1373		K113>U1348		
**p1394**	0	**0.09**	1	H28 (L)	A 1394, A 1396, C 1402	**uS5**	**mRNA**		**uS12**		

**1396,1402**						R24 > A 1396	C 1402		D92.K46.47 > 1491		
									pro 45.48. 94 >1491		
**SSU-body**											
**p62**	0.34	**0**	**2**	H6 (T)	U 62 - G105	**bS20**			**bS16**		
						K14.R15 (S)>G105			D23.R25 > G 110		
									D68> G380. G105		
**p149**	0	**0**	**1**	H8 (T)	A 172 -A 149	**bS20**			**bS20**		
						K21, R25 > A149			D64.K 65 > C 177		
**p201**	0	**0**	**1**	H10 (T)	C 201-G216				**bS20**		
									D64.K 65 > C 177		
**p441**	0.55	**0.02**	**2**	H17 (L)	A441- G493	**uS4**			bS16		
						E150-K151)>491			Y17.Y39. P41> G450		
						his 123 > 439.					
						Cter αHel >439					
**p593**	0.03	**0.13**	**3**	H21 (T)	G 593-U 646	**uS15**	**uS17**		**uS8**		
						Y69.D74.R77..A753	E24>U 646)/U646		E132.R92.Y94>G644		
						H42> C764			F31>C643		
**p831**	0.06	**0.21**	**3**	H26/H26a (T)	U 831-G 855	**uS2**			**uS8**	**bS18**	
						R21, K22 >U831			D8.R12>C826	K61.64> U835	
						W24/P26> G829				N-ter α-hel> U 835	
						cluster ED... U831					
						ED pathway					
**LSU**											
**pL703**	0	0.01	1	H34	U703-G728	**uL2 ext N and C-ter**					
						T10. K208 > G728					
						W 214 > > G728					
**L1 stalk**											
**pL2198**	0.15	0.02	3	H79	A2198. C2196-G2093	**uL2**	**bL9**	**bL28**			
						Y172 > C2196	R33>A2198. K22 >G2093	R50 (NC) > A2199			
							Y25/Y29 > A2198	I67> U2091			
**central protuberance**											
**pL2298**	0.09	0.02	3	H84	A2298-G2318	**uL5**	**uL18**		**uL18**	**uL33**	
						K74.K75> A2298	R13> G2318		R13>U2296	K25.K27 > C2285	
						K36> G2315	K11 (NC) >G2318		Y92>C2293	N26>A2286	
						R128 >C2316					

						D126 > G2303					
						D45>C2306					
						F80>A2311					
L12-Stalk											
**pL1032**	0.71	0.11	7	H42	A1032-G1122	**bL36**	**uL16**		**bL25-dom1**	**uL6**	
						R8>A1032	E116..A1032		K46 (NC)> C1041	R59(NC)> G1036	
						R18>G1122	E111...C1116			N-ter α-hel> G1036	
						V16.V23. Q36>A1032	K128> A1029		**uL10**	**uL13**	
									R56> G1047	K65>G1022>G1122	
									R62> A 1046	bL20	
									R8> C1044	R70.N75>H41>G1122	
									L3 > C1043		
**pL871**	0	0	2	H38	U871-G906	**uL16**	**bL25**				
						F29>G906	E168.H151(NC) > G875				
						F69. P4 >U871					
						R61>G906					
						R5 >U871.R6> G869					
						K8/Y9cat-p> A911					
**pL149**	0.06	0.01	1	H9.H10	A149-G177	**bL34**			**uL29**	**uL23**	
						R19>G117.R28>G179			NC basic>U112>G12>A149	T35> C143. K40>G139	
						F18 > G117					
						M22 >A119					
**pL1528**	0	0	1	H59	A1528-G1540				**bL17**		
									R63.R64>U1453..G1448>A1542		
**pL1907**	-	-	-	H69	G1907-U1923	Helix D of tRNA-P					

Table S1

**Steps**		**tRNAs**	**EF-G**	**Domain motions**	**PDB_id**
**Step 1**	PRE-C	aA/A ; pP/P	-		7n1p
**Step 2**	Pre H2*	aA/P; pP/E	-	Subunit rotation	7n30
**Step 3**	Pre H1	aA/P ; pP/E	-	Subunit rotation	7n2u
**Step 4**	INT1-SPC	aA/P ; pP/E	EF-G (GTP)	Head swivel	7n2v
**Step 5**	INT2-FA	aP/P ; pE/E	EF-G	Back rotation/swivel	7n2c
**Step 6**	POST	pP/P ; eE/E	-	Reverse swivel	7n31

Table S2

Protein	Protein	Fonctional centre	Centre of motion
**uS2**	uS3		
	uS8		
			P831
			P1074
			P1156
**uS3**	uS2		
	uS5		
	uS10		
	uS14		
		mRNA	
			(P1074)
			(P1125)
			(P1156)
**uS4**	uS5		
			(p441)
**uS5**	uS3		
	uS4		
	uS8		
		mRNA	
			P1074
			P1394
**bS6**	uS15		
	uL2		
	bS18		
**uS7**	uS9		
	uS11		
		mRNA	
		tRNA-E	
			P1247
			P1351
			(P1304)
**uS8**	uS2		
	uS5		
	uS12		
	uS17		
			(P593) *
			(P831)
			(P1074)
**uS9**	uS7		
	uS10		
		tRNA-P	
			P1125
			P1156
			P1247
			P1351
			(P955)
**uS10**	uS3		
	uS9		
	uS14		
			P1125
			(P1156)
			(P1351)
**uS11**	uS7		
	bS18		
		tRNA-E	
		mRNA	
**uS12**	uS8		
	uS17		
		mRNA	
		tRNA-A	
			(P1412)
**uS13**	uL5		
	uS19		
	bL31		
		tRNA-A	

		tRNA-P	
			P955
			(P1247)
			P1304
**uS14**	uS3		
	uS10		
			(P1156)
			(P1212)
			(P1351)
**uS15**	uS17		
	bS6		
			(P593)
**bS16**			(P441)
			(P62)
**uS17**	uS8		
	uS12		
	uS15		
			P593
**bS18**	bS6		
	uS11		
			(P831)
**uS19**	uS13		
	uL5		
	bL31		
			P955
			(P1304)
			(P1212)
**bS20**			P62
			P149
			(P201)
**THX**			P1351
			P1304
			(P1247)

Table S3 (A)

Protein	Protein	Fonctional centre	CM
**uL1**		tRNA-E	
**uL2**	bS6		
		PTC	
			pL703
			pL2198
**uL3**	uL14		
	uL13		
	bL17		
	bL19		
		PTC	
**uL4**	uL15		
	bL20		
		tunnel	
**uL5**	uS13		
	uS19		
	bL31		
		tRNA-P	
			pL2298
**uL6**		PTC	
			(PL1032)
**bL9**	bL28		
			PL2198
**uL10**	uL11		
			(PL1032)
**uL11**	uL10		
	bL12		
**bL12**	uL11		
**uL13**	uL3		
	bL20		
	bL21		
			(PL1032)
**uL14**	uL3		
	bL19		
		PTC	
**uL15**	uL4		
	bL21		
	bL35		
**uL16**	bL25		
	bL27		
		tRNA-A	
		tRNA-P	
		tRNA-P	
		PTC	
			pL871
			pL1032
**bL17**	uL3		
	uL22		
	bL32		
			(pL1528)
**uL18**	bL27		
			(PL2298)
**bL19**	uL3		

	uL14		
**bL20**	uL4		
	uL13		
	bL21		
	bL32		
			(PL1032)
**bL21**	uL13		
	uL15		
	bL20		
**uL22**	bL17		
	bL32		
		tunnel	
**uL23**	uL29		
	bL34		
		tunnel	
			(PL149)
**uL24**		tunnel	
**bL25**	uL16		
			PL871
			(PL1032)
**bL27**	uL16		
	uL18		
		tRNA-A	
		tRNA-P	
		PTC	
			(PL2298)
**bL28**	bL9		
		tRNA-E	
			PL2198
			(pL149)
**uL29**	uL23		
**uL30**			
**bL31**	uL5		
	uS13		
	uS19		
**bL32**	bL17		
	bL20		
	uL22		
		PTC	
**uL33**	bL35		
		tRNA-E	
			(PL2298)
**bL34**	uL23		
		tunnel	
			PL149
**bL35**	uL15		
	uL33		
		tRNA-E	

Table S3 (B)

	step2		step3		step4		step5		step6		Nb
	Σd	Σd pond	Σd	Σd pond	Σd	Σd pond	Σd	Σd pond	Σd	Σd pond	
**uL2**	140.3	4.5	158.1	5.1	156.8	5.1	143.1	4.6	72.2	2.3	309
**uL14**	78.2	7.2	84.9	7.9	87.3	8.1	70.6	6.5	58.7	5.4	108
**uS7**	75.0	11.0	108.8	16.0	144.5	21.2	227.3	33.4	64.2	9.4	68
**uL11**	62.2	27.0	37.5	16.3	41.3	18.0	71.0	30.9	62.8	27.3	23
**bL19**	60.8	5.7	62.1	5.8	74.4	7.0	72.3	6.8	58.9	5.5	107
**bL9**	58.9	13.1	58.7	13.0	46.5	10.3	44.2	9.8	32.4	7.2	45
**uS15**	56.4	6.8	60.0	7.2	57.7	6.9	59.0	7.1	28.9	3.5	83
**uL10**	53.3	18.4	29.2	10.1	80.5	27.7	77.5	26.7	45.7	15.8	29
**uS12**	50.5	3.1	150.0	9.3	186.8	11.5	115.1	7.1	262.7	16.2	162
**bS20**	49.9	3.9	47.1	3.7	53.4	4.1	72.3	5.6	43.7	3.4	129
**uS3**	49.2	7.7	86.9	13.6	147.4	23.0	313.8	49.0	66.6	10.4	64
**uL5**	48.3	7.1	41.4	6.1	35.5	5.2	39.4	5.8	21.4	3.1	68
**uL15**	45.3	1.8	42.8	1.7	43.6	1.8	53.3	2.2	41.4	1.7	246
**uL3**	43.7	1.8	48.4	2.0	51.7	2.1	53.6	2.2	41.7	1.7	245
**uL6**	42.2	4.6	34.0	3.7	69.2	7.5	79.1	8.6	35.5	3.9	92
**uL16**	40.8	2.9	55.8	4.0	54.5	3.9	57.9	4.1	41.7	3.0	141
**bL27**	40.8	2.6	50.0	3.2	48.0	3.1	46.3	3.0	35.6	2.3	154
**uL4**	36.8	1.9	41.4	2.2	39.9	2.1	48.1	2.5	32.3	1.7	192
**uS11**	36.5	4.2	39.2	4.5	53.6	6.2	92.6	10.6	67.1	7.7	87
**bL31**	34.2	18.0	34.3	18.0	36.9	19.4	49.1	25.8	12.8	6.7	19
**bL28**	32.2	2.5	36.5	2.9	36.6	2.9	33.7	2.7	24.0	1.9	127
**bL36**	31.4	3.7	29.6	3.5	51.7	6.1	52.5	6.2	32.3	3.8	85
**uS5**	31.0	3.3	50.5	5.4	75.4	8.0	101.7	10.8	48.8	5.2	94
**uS4**	29.9	2.6	60.0	5.2	45.7	4.0	63.2	5.5	47.3	4.1	115
**uS9**	27.6	3.9	29.9	4.2	35.6	5.0	70.9	10.0	22.6	3.2	71
**bL21**	27.4	2.6	25.0	2.4	40.0	3.8	37.9	3.6	22.0	2.1	104
**uS2**	27.4	5.2	28.8	5.4	49.1	9.3	80.2	15.1	17.6	3.3	53
**uS17**	27.1	2.9	45.5	4.8	53.6	5.7	46.4	4.9	82.8	8.8	94
**uL13**	26.8	1.8	29.6	2.0	43.6	3.0	42.1	2.9	29.1	2.0	147
**uL18**	26.7	3.2	29.0	3.5	27.2	3.2	28.1	3.3	20.2	2.4	84
**bL20**	26.6	1.4	29.4	1.5	33.4	1.7	42.7	2.2	30.6	1.6	193
**bS18**	25.3	5.1	23.4	4.7	30.2	6.0	36.3	7.3	15.9	3.2	50
**bL34**	24.3	1.7	28.2	1.9	28.1	1.9	30.3	2.1	26.7	1.8	145
**bL35**	24.1	1.7	26.6	1.8	24.8	1.7	28.3	2.0	23.8	1.7	144
**bS16**	23.5	2.1	42.4	3.8	31.8	2.8	46.9	4.2	32.2	2.9	112
**uL23**	23.3	2.3	29.0	2.8	27.8	2.7	29.2	2.9	24.1	2.4	102
**bL32**	21.5	1.6	19.5	1.5	22.6	1.7	23.9	1.8	26.1	2.0	133
**uL22**	20.6	1.5	23.5	1.7	25.8	1.9	26.8	2.0	21.7	1.6	137
**uS8**	20.3	2.4	36.0	4.2	38.7	4.5	38.8	4.5	41.2	4.8	86
**uS14**	18.8	2.8	16.9	2.5	20.8	3.1	28.8	4.2	16.2	2.4	68
**bL17**	18.3	1.3	21.5	1.5	24.5	1.7	25.7	1.8	19.8	1.4	140
**uL24**	14.0	1.7	15.8	1.9	18.4	2.2	26.1	3.1	19.4	2.3	84
**uS10**	13.7	2.4	12.5	2.2	14.7	2.6	27.0	4.7	13.9	2.4	57
**bS6**	13.1	4.4	15.7	5.2	13.6	4.5	20.5	6.8	9.7	3.2	30
**uL30**	12.3	1.6	12.7	1.6	17.3	2.2	20.5	2.7	17.0	2.2	77
**bL25**	12.1	2.2	12.1	2.2	18.3	3.4	22.9	4.2	12.3	2.3	54
**uL33**	11.9	2.3	14.4	2.8	14.7	2.8	21.5	4.1	12.7	2.4	52
**uS13**	11.9	2.8	10.3	2.5	15.6	3.7	18.7	4.5	12.8	3.1	42
**uL29**	10.7	2.2	13.5	2.8	12.3	2.6	16.5	3.4	14.1	2.9	48
**uS19**	7.6	3.2	6.7	2.8	14.8	6.2	11.0	4.6	5.5	2.3	24

Table S4

Prot	Res		Σdispl	MinDist	Nb_nucl	Av_displ
**uS2**	**GLU**	**78**	2.4	16.9	2	1.2
**uS2**	**ASP**	**116**	3.6	14.4	4	0.9
**uS2**	**GLU**	**118**	4.0	16.0	4	1.0
**uS2**	**ASP**	**127**	6.9	18.4	1	6.9
**uS2**	**GLU**	**133**	53.3	11.5	18	3.0
**uS2**	**GLU**	**140**	31.4	9.0	15	2.1
**uS2**	**GLU**	**142**	30.4	9.8	12	2.5
**uS2**	**GLU**	**145**	16.1	11.8	8	2.0
**uS2**	**ASP**	**153**	1.0	17.1	1	1.0
**uS2**	**ASP**	**159**	0.9	18.3	1	0.9
**uS2**	**ASP**	**165**	4.9	14.8	2	2.4
**uS2**	**ASP**	**167**	12.3	12.5	11	1.1
**uS2**	**GLU**	**169**	15.3	9.5	11	1.4
**uS2**	**GLU**	**175**	13.8	7.7	13	1.1
**uS2**	**ASP**	**188**	4.5	16.3	5	0.9
**uS2**	**ASP**	**192**	11.3	11.8	11	1.0
**uS2**	**ASP**	**194**	8.8	11.8	9	1.0
**uS2**	**ASP**	**197**	2.4	18.9	3	0.8
**uS2**	**ASP**	**204**	3.5	19.1	2	1.8
**uS2**	**ASP**	**205**	2.5	18.3	2	1.2
**uS3**	**GLU**	**28**	4.6	11.2	7	0.7
**uS3**	**ASP**	**31**	2.6	14.3	5	0.5
**uS3**	**ASP**	**34**	2.3	17.1	4	0.6
**uS3**	**GLU**	**46**	2.2	18.3	2	1.1
**uS3**	**ASP**	**93**	0.5	17.1	1	0.5
**uS3**	**ASP**	**112**	0.8	18.8	2	0.4
**uS3**	**ASP**	**118**	2.4	14.1	5	0.5
**uS3**	**GLU**	**125**	19.4	12.2	9	2.2
**uS3**	**GLU**	**152**	13.0	8.8	14	0.9
**uS3**	**GLU**	**161**	67.3	6.2	16	4.2
**uS3**	**GLU**	**166**	16.0	8.1	15	1.1
**uS3**	**GLU**	**170**	24.1	9.2	12	2.0
**uS3**	**ASP**	**181**	7.2	13.2	13	0.6
**uS3**	**ASP**	**183**	6.2	14.5	10	0.6
**uS3**	**GLU**	**188**	3.2	8.0	7	0.5
**uS3**	**GLU**	**206**	9.4	12.4	12	0.8
**uS4**	**GLU**	**15**	11.9	9.6	15	0.8
**uS4**	**ASP**	**18**	11.8	12.9	15	0.8
**uS4**	**ASP**	**29**	15.3	10.1	15	1.0
**uS4**	**GLU**	**35**	12.8	11.4	15	0.9
**uS4**	**ASP**	**50**	20.6	8.1	17	1.2
**uS4**	**GLU**	**57**	7.8	10.7	11	0.7
**uS4**	**GLU**	**69**	26.6	4.1	30	0.9
**uS4**	**GLU**	**78**	44.5	9.6	31	1.4
**uS4**	**GLU**	**88**	8.4	16.4	5	1.7
**uS4**	**GLU**	**95**	5.0	14.8	8	0.6
**uS4**	**ASP**	**99**	12.9	9.9	17	0.8
**uS4**	**GLU**	**113**	18.1	5.2	19	1.0
**uS4**	**ASP**	**141**	6.5	14.0	8	0.8
**uS4**	**GLU**	**147**	6.1	14.0	6	1.0
**uS4**	**GLU**	**160**	6.2	13.3	5	1.2
**uS4**	**GLU**	**163**	2.7	16.1	3	0.9
**uS4**	**GLU**	**166**	0.9	18.3	1	0.9
**uS4**	**GLU**	**187**	2.7	19.2	2	1.3
**uS4**	**ASP**	**194**	0.3	19.6	1	0.3
**uS4**	**GLU**	**197**	13.3	16.6	7	1.9
**uS4**	**GLU**	**202**	38.0	11.2	21	1.8
**uS5**	**GLU**	**13**	1.6	19.4	2	0.8
**uS5**	**GLU**	**55**	18.7	9.9	13	1.4
**uS5**	**GLU**	**65**	8.1	8.7	8	1.0
**uS5**	**GLU**	**101**	27.5	12.3	9	3.1
**uS5**	**GLU**	**116**	4.5	15.6	4	1.1
**uS5**	**ASP**	**142**	12.0	12.7	18	0.7
**uS5**	**GLU**	**145**	4.0	15.8	6	0.7
**bS6**	**GLU**	**5**	7.9	9.2	10	0.8
**bS6**	**ASP**	**13**	0.7	19.9	1	0.7
**bS6**	**GLU**	**33**	1.3	18.2	2	0.6
**bS6**	**GLU**	**40**	0.2	19.9	1	0.2
**bS6**	**GLU**	**65**	7.5	12.1	7	1.1
**bS6**	**GLU**	**69**	9.9	10.6	7	1.4
**bS6**	**ASP**	**72**	11.2	10.0	9	1.2
**bS6**	**GLU**	**73**	8.4	13.6	7	1.2
**bS6**	**GLU**	**75**	9.0	11.3	9	1.0

**bS6**	**ASP**	**82**	5.2	13.4	6	0.9
**bS6**	**GLU**	**98**	1.0	19.8	1	1.0
**uS7**	**ASP**	**15**	1.7	11.7	2	0.8
**uS7**	**GLU**	**21**	3.2	13.0	2	1.6
**uS7**	**ASP**	**33**	26.0	7.0	24	1.1
**uS7**	**GLU**	**40**	8.8	12.0	9	1.0
**uS7**	**GLU**	**48**	2.3	16.4	1	2.3
**uS7**	**GLU**	**74**	7.3	10.7	4	1.8
**uS7**	**GLU**	**90**	2.3	14.2	2	1.1
**uS7**	**GLU**	**106**	19.6	9.5	10	2.0
**uS7**	**ASP**	**113**	6.4	9.5	5	1.3
**uS7**	**GLU**	**123**	5.0	14.5	3	1.7
**uS7**	**GLU**	**139**	2.5	17.2	1	2.5
**uS7**	**ASP**	**140**	8.9	15.7	3	3.0
**uS8**	**ASP**	**5**	15.1	7.0	30	0.5
**uS8**	**ASP**	**9**	15.7	9.2	27	0.6
**uS8**	**GLU**	**42**	0.5	19.6	1	0.5
**uS8**	**GLU**	**47**	0.6	19.1	1	0.6
**uS8**	**ASP**	**48**	0.5	19.0	1	0.5
**uS8**	**GLU**	**52**	8.3	14.1	8	1.0
**uS8**	**ASP**	**54**	11.4	11.3	11	1.0
**uS8**	**GLU**	**58**	12.2	11.5	14	0.9
**uS8**	**GLU**	**60**	6.5	13.0	9	0.7
**uS8**	**GLU**	**73**	6.6	12.0	9	0.7
**uS8**	**ASP**	**90**	36.0	7.5	16	2.2
**uS8**	**GLU**	**91**	35.5	8.9	17	2.1
**uS8**	**ASP**	**113**	5.3	13.2	9	0.6
**uS8**	**GLU**	**124**	17.9	8.5	25	0.7
**uS9**	**GLU**	**36**	4.9	13.7	4	1.2
**uS9**	**GLU**	**42**	11.3	14.2	7	1.6
**uS9**	**GLU**	**89**	4.1	13.2	4	1.0
**uS9**	**ASP**	**91**	1.8	18.7	2	0.9
**uS9**	**GLU**	**92**	1.7	16.7	2	0.8
**uS9**	**GLU**	**97**	1.8	14.0	2	0.9
**uS9**	**ASP**	**107**	11.5	8.3	17	0.7
**uS9**	**GLU**	**112**	23.2	4.9	27	0.9
**uS10**	**ASP**	**14**	7.5	8.6	11	0.7
**uS10**	**ASP**	**19**	7.2	8.3	7	1.0
**uS10**	**GLU**	**24**	2.9	16.5	2	1.4
**uS10**	**GLU**	**27**	2.7	19.5	2	1.3
**uS10**	**GLU**	**47**	12.7	5.0	20	0.6
**uS10**	**ASP**	**60**	12.7	7.3	26	0.5
**uS10**	**ASP**	**63**	14.2	7.5	22	0.6
**uS10**	**GLU**	**66**	16.0	10.3	22	0.7
**uS10**	**ASP**	**75**	7.1	14.3	9	0.8
**uS10**	**GLU**	**78**	1.3	18.7	1	1.3
**uS10**	**ASP**	**97**	7.2	11.0	8	0.9
**uS11**	**ASP**	**18**	5.2	15.7	6	0.9
**uS11**	**ASP**	**36**	7.9	10.7	11	0.7
**uS11**	**GLU**	**68**	5.8	15.3	6	1.0
**uS11**	**ASP**	**72**	5.6	12.8	5	1.1
**uS11**	**GLU**	**76**	3.7	12.3	4	0.9
**uS11**	**GLU**	**83**	3.6	12.5	5	0.7
**uS11**	**GLU**	**94**	2.1	17.0	4	0.5
**uS11**	**ASP**	**112**	5.8	12.1	10	0.6
**uS12**	**GLU**	**25**	51.3	7.8	31	1.7
**uS12**	**GLU**	**62**	70.8	7.5	19	3.7
**uS12**	**GLU**	**70**	16.1	4.2	22	0.7
**uS12**	**GLU**	**76**	9.5	16.5	4	2.4
**uS12**	**ASP**	**103**	7.8	11.5	12	0.6
**uS12**	**ASP**	**109**	14.9	7.5	20	0.7
**uS13**	**ASP**	**11**	0.2	18.7	1	0.2
**uS13**	**GLU**	**41**	2.7	14.4	4	0.7
**uS13**	**ASP**	**42**	2.1	15.0	3	0.7
**uS13**	**ASP**	**58**	1.2	17.9	2	0.6
**uS13**	**GLU**	**59**	1.8	14.9	3	0.6
**uS13**	**GLU**	**66**	5.4	9.5	7	0.8
**uS13**	**ASP**	**68**	3.9	13.1	5	0.8
**uS13**	**GLU**	**72**	4.8	9.5	6	0.8
**uS13**	**ASP**	**82**	6.7	13.9	6	1.1
**uS14**	**GLU**	**10**	16.3	8.8	24	0.7
**uS14**	**ASP**	**18**	13.4	11.2	11	1.2
**uS14**	**GLU**	**26**	6.8	11.7	6	1.1
**uS14**	**ASP**	**33**	2.7	13.9	3	0.9
**uS14**	**ASP**	**38**	1.1	16.5	1	1.1
**uS14**	**GLU**	**39**	0.9	17.0	1	0.9
**uS14**	**ASP**	**40**	2.6	15.8	3	0.9

**uS14**	**ASP**	**54**	15.5	8.9	19	0.8
**uS14**	**GLU**	**86**	10.0	9.2	16	0.6
**uS14**	**GLU**	**92**	3.1	16.6	4	0.8
**uS15**	**GLU**	**6**	9.5	8.7	9	1.1
**uS15**	**GLU**	**14**	4.4	15.7	7	0.6
**uS15**	**ASP**	**18**	7.0	10.3	10	0.7
**uS15**	**ASP**	**21**	10.1	6.5	15	0.7
**uS15**	**GLU**	**26**	19.9	10.6	17	1.2
**uS15**	**GLU**	**45**	42.1	10.0	19	2.2
**uS15**	**ASP**	**49**	25.6	6.3	34	0.8
**uS15**	**ASP**	**68**	45.5	9.2	24	1.9
**uS15**	**ASP**	**74**	9.3	9.7	12	0.8
**uS15**	**GLU**	**83**	42.2	14.1	5	8.4
**bS16**	**ASP**	**23**	22.0	6.7	38	0.6
**bS16**	**GLU**	**34**	22.5	10.1	28	0.8
**bS16**	**GLU**	**45**	10.8	9.4	15	0.7
**bS16**	**GLU**	**47**	12.9	7.9	15	0.9
**bS16**	**GLU**	**48**	14.4	10.5	19	0.8
**bS16**	**ASP**	**53**	8.6	10.8	10	0.9
**bS16**	**ASP**	**55**	7.3	15.7	7	1.0
**bS16**	**ASP**	**69**	23.1	6.7	29	0.8
**bS16**	**GLU**	**77**	14.2	8.6	15	0.9
**uS17**	**ASP**	**15**	10.0	7.1	13	0.8
**uS17**	**GLU**	**18**	12.3	7.4	17	0.7
**uS17**	**GLU**	**26**	43.0	10.4	29	1.5
**uS17**	**ASP**	**48**	11.2	9.5	15	0.7
**uS17**	**GLU**	**49**	10.7	9.0	12	0.9
**uS17**	**GLU**	**52**	6.9	15.4	8	0.9
**uS17**	**ASP**	**57**	13.8	13.4	9	1.5
**uS17**	**GLU**	**60**	30.4	13.5	17	1.8
**uS17**	**GLU**	**63**	25.6	8.8	26	1.0
**uS17**	**GLU**	**80**	19.5	11.8	7	2.8
**bS18**	**GLU**	**16**	26.5	7.0	13	2.0
**bS18**	**GLU**	**20**	21.1	9.2	17	1.2
**bS18**	**ASP**	**22**	9.1	12.2	11	0.8
**bS18**	**ASP**	**25**	2.0	16.1	3	0.7
**bS18**	**GLU**	**35**	4.8	14.3	7	0.7
**bS18**	**ASP**	**72**	8.9	10.1	11	0.8
**uS19**	**ASP**	**12**	10.0	9.0	12	0.8
**uS19**	**GLU**	**20**	2.8	12.2	3	0.9
**uS19**	**GLU**	**24**	1.5	15.8	2	0.8
**uS19**	**ASP**	**27**	1.3	15.2	2	0.7
**uS19**	**GLU**	**73**	4.5	11.4	5	0.9
**bS20**	**GLU**	**15**	41.2	8.5	43	1.0
**bS20**	**GLU**	**40**	5.6	12.1	8	0.7
**bS20**	**ASP**	**43**	3.2	17.3	5	0.6
**bS20**	**GLU**	**53**	21.4	9.6	24	0.9
**bS20**	**ASP**	**59**	33.5	7.9	44	0.8

Table S5 (A)

Prot	res		□displ	minDist	Nb_nucl	Av_displ
uL2	GLU	23	17.1	12.1	10	1.7
uL2	GLU	35	18.2	6.7	35	0.5
uL2	ASP	66	11.9	10.3	20	0.6
uL2	ASP	72	7.9	12.3	9	0.9
uL2	GLU	79	7.9	10.5	9	0.9
uL2	GLU	82	11.1	10.9	19	0.6
uL2	ASP	84	10.0	7.8	22	0.5
uL2	ASP	98	15.1	7.1	17	0.9
uL2	GLU	100	15.2	8.1	19	0.8
uL2	ASP	114	4.9	14.1	3	1.6
uL2	GLU	145	8.1	10.2	16	0.5
uL2	ASP	168	25.6	12.5	12	2.1
uL2	GLU	180	35.9	9.5	28	1.3
uL2	GLU	185	20.6	8.3	19	1.1
uL2	ASP	187	8.1	10.4	12	0.7
uL2	GLU	194	24.1	11.2	14	1.7
uL2	GLU	199	25.6	10.6	23	1.1
uL2	ASP	229	28.9	8.8	53	0.5
uL2	GLU	236	46.3	6.4	51	0.9
uL2	ASP	264	67.5	7.4	34	2.0
uL3	GLU	17	1.4	16.5	3	0.5
uL3	ASP	18	2.2	15.2	6	0.4
uL3	GLU	28	4.0	14.4	10	0.4
uL3	GLU	30	3.3	18.1	7	0.5
uL3	ASP	39	5.1	11.1	10	0.5
uL3	ASP	43	7.1	7.1	15	0.5
uL3	GLU	64	9.3	8.9	22	0.4
uL3	GLU	74	6.4	15.7	13	0.5
uL3	GLU	81	8.4	4.9	19	0.4
uL3	GLU	86	2.5	15.2	6	0.4
uL3	GLU	88	2.0	17.1	5	0.4
uL3	GLU	89	2.0	17.7	4	0.5
uL3	ASP	103	0.7	18.7	2	0.3
uL3	ASP	108	6.2	8.9	16	0.4
uL3	ASP	131	22.0	5.1	55	0.4
uL3	GLU	168	13.3	7.0	37	0.4
uL3	ASP	176	6.9	7.5	14	0.5
uL3	ASP	200	11.6	12.2	26	0.4
uL4	GLU	16	0.9	15.3	4	0.2
uL4	ASP	22	0.9	11.1	4	0.2
uL4	GLU	25	5.7	9.3	16	0.4
uL4	GLU	51	17.6	7.7	44	0.4
uL4	ASP	91	14.4	8.1	34	0.4
uL4	GLU	111	2.0	16.4	6	0.3
uL4	ASP	116	0.5	18.7	1	0.5
uL4	GLU	127	2.6	12.5	5	0.5
uL4	ASP	140	4.3	16.5	6	0.7
uL4	GLU	144	7.7	13.2	15	0.5
uL4	ASP	145	4.6	10.6	12	0.4
uL4	GLU	152	29.7	11.7	5	5.9
uL4	ASP	154	5.6	15.0	7	0.8
uL4	GLU	155	6.0	12.2	10	0.6
uL4	ASP	168	7.4	12.0	14	0.5
uL4	ASP	171	29.4	9.4	7	4.2
uL4	ASP	176	21.0	8.6	16	1.3
uL4	ASP	184	3.4	9.5	7	0.5
uL4	ASP	191	0.3	19.3	1	0.3
uL4	GLU	197	2.3	12.6	6	0.4
uL4	GLU	198	4.9	11.2	9	0.5
uL5	ASP	6	4.8	11.1	7	0.7
uL5	ASP	10	6.2	14.1	8	0.8
uL5	GLU	11	2.7	16.9	4	0.7
uL5	GLU	19	3.5	18.3	4	0.9
uL5	GLU	32	16.2	9.3	19	0.9
uL5	GLU	42	9.9	12.0	9	1.1
uL5	ASP	46	3.0	17.6	3	1.0
uL5	ASP	51	3.9	13.5	6	0.7
uL5	ASP	56	3.5	16.0	5	0.7
uL5	GLU	94	12.7	9.0	14	0.9
uL5	GLU	98	6.1	14.4	7	0.9
uL5	GLU	101	3.0	18.6	4	0.8
uL5	ASP	113	4.0	15.4	3	1.3
uL5	ASP	123	19.1	6.8	17	1.1
uL5	GLU	134	8.6	11.9	5	1.7
uL5	ASP	153	18.3	7.5	14	1.3
uL5	ASP	163	16.1	12.2	12	1.3

**uL5**	**GLU**	**164**	9.4	15.4	8	1.2
**uL5**	**GLU**	**165**	9.7	15.7	9	1.1
**uL6**	**GLU**	**32**	2.5	18.0	2	1.3
**uL6**	**ASP**	**39**	6.2	10.6	9	0.7
**uL6**	**GLU**	**42**	4.2	16.4	6	0.7
**uL6**	**ASP**	**56**	9.0	15.8	10	0.9
**uL6**	**ASP**	**60**	19.6	10.1	24	0.8
**uL6**	**GLU**	**81**	2.1	15.4	3	0.7
**uL6**	**ASP**	**114**	9.8	14.7	10	1.0
**uL6**	**GLU**	**130**	1.6	19.3	3	0.5
**uL6**	**ASP**	**137**	9.8	10.3	12	0.8
**uL6**	**ASP**	**147**	18.2	10.1	21	0.9
**uL6**	**GLU**	**155**	14.8	9.9	24	0.6
**uL6**	**ASP**	**166**	18.4	13.2	12	1.5
**uL6**	**GLU**	**167**	20.7	11.9	16	1.3
**uL6**	**GLU**	**173**	32.8	8.2	20	1.6
**bL9**	**ASP**	**7**	7.3	15.9	4	1.8
**bL9**	**ASP**	**17**	1.8	16.3	2	0.9
**bL9**	**ASP**	**98**	5.0	11.9	6	0.8
**bL9**	**ASP**	**101**	5.3	14.9	5	1.1
**bL9**	**GLU**	**109**	10.2	16.4	5	2.0
**bL9**	**GLU**	**114**	6.9	12.4	8	0.9
**bL9**	**GLU**	**129**	2.2	19.3	2	1.1
**bL9**	**GLU**	**137**	8.4	16.8	4	2.1
**uL10**	**ASP**	**7**	34.6	10.9	12	2.9
**uL10**	**GLU**	**14**	25.9	13.5	7	3.7
**uL10**	**ASP**	**29**	39.2	11.8	8	4.9
**uL10**	**ASP**	**36**	71.2	7.4	15	4.7
**uL10**	**GLU**	**40**	20.8	14.9	6	3.5
**uL10**	**GLU**	**47**	14.9	13.6	2	7.5
**uL10**	**GLU**	**65**	11.2	15.5	3	3.7
**uL10**	**GLU**	**70**	2.1	18.9	1	2.1
**uL10**	**ASP**	**74**	23.4	15.5	4	5.9
**uL10**	**GLU**	**107**	1.8	19.1	1	1.8
**uL11**	**GLU**	**50**	2.5	16.8	1	2.5
**uL11**	**ASP**	**96**	11.6	13.7	2	5.8
**uL11**	**GLU**	**108**	13.4	14.3	3	4.5
**uL11**	**ASP**	**116**	46.8	6.7	10	4.7
**uL11**	**ASP**	**121**	49.1	7.0	7	7.0
**uL11**	**GLU**	**123**	31.8	9.4	5	6.4
**uL11**	**GLU**	**130**	51.3	10.2	10	5.1
**uL13**	**GLU**	**9**	11.6	7.9	22	0.5
**uL13**	**ASP**	**14**	8.2	12.2	11	0.7
**uL13**	**ASP**	**19**	1.6	16.9	4	0.4
**uL13**	**GLU**	**31**	8.8	8.7	20	0.4
**uL13**	**GLU**	**43**	5.3	14.1	15	0.4
**uL13**	**ASP**	**49**	8.8	9.8	26	0.3
**uL13**	**ASP**	**52**	7.6	11.6	15	0.5
**uL13**	**ASP**	**60**	3.3	14.6	7	0.5
**uL13**	**ASP**	**71**	19.5	8.0	35	0.6
**uL13**	**GLU**	**90**	16.8	10.5	24	0.7
**uL13**	**GLU**	**91**	16.3	10.5	24	0.7
**uL13**	**GLU**	**98**	8.6	11.8	18	0.5
**uL13**	**GLU**	**102**	10.4	8.8	25	0.4
**uL13**	**GLU**	**129**	7.0	13.5	9	0.8
**uL13**	**ASP**	**141**	2.4	16.8	7	0.3
**uL14**	**GLU**	**4**	17.6	8.8	41	0.4
**uL14**	**ASP**	**12**	37.4	8.9	11	3.4
**uL14**	**ASP**	**37**	12.9	10.8	19	0.7
**uL14**	**GLU**	**45**	51.1	9.4	26	2.0
**uL14**	**ASP**	**56**	19.6	7.5	20	1.0
**uL14**	**ASP**	**73**	6.8	10.1	12	0.6
**uL14**	**ASP**	**80**	19.7	8.9	21	0.9
**uL14**	**GLU**	**92**	9.1	12.5	8	1.1
**uL14**	**GLU**	**106**	12.0	11.8	13	0.9
**uL14**	**GLU**	**110**	21.1	11.3	9	2.3
**uL14**	**GLU**	**121**	52.4	6.3	13	4.0
**uL15**	**GLU**	**10**	14.7	7.6	24	0.6
**uL15**	**GLU**	**51**	23.5	7.3	52	0.5
**uL15**	**GLU**	**76**	13.9	10.6	27	0.5
**uL15**	**ASP**	**81**	7.0	9.8	17	0.4
**uL15**	**GLU**	**86**	2.1	15.7	6	0.4
**uL15**	**ASP**	**91**	4.4	16.4	10	0.4
**uL15**	**GLU**	**106**	12.8	9.8	29	0.4
**uL15**	**GLU**	**115**	10.8	11.8	14	0.8
**uL15**	**GLU**	**136**	1.4	14.4	4	0.4
**uL15**	**GLU**	**143**	1.1	15.7	4	0.3

**uL15**	**GLU**	**144**	2.1	13.9	6	0.3
**uL16**	**ASP**	**25**	8.1	8.1	16	0.5
**uL16**	**GLU**	**47**	8.5	7.8	13	0.7
**uL16**	**ASP**	**70**	11.4	8.5	31	0.4
**uL16**	**GLU**	**75**	24.0	4.2	26	0.9
**uL16**	**GLU**	**90**	11.7	10.2	18	0.6
**uL16**	**GLU**	**104**	5.5	12.2	8	0.7
**uL16**	**ASP**	**106**	4.2	12.3	6	0.7
**uL16**	**GLU**	**110**	5.9	15.1	5	1.2
**uL16**	**GLU**	**111**	7.7	13.8	8	1.0
**uL16**	**GLU**	**115**	15.1	10.2	14	1.1
**bL17**	**GLU**	**32**	4.0	10.3	11	0.4
**bL17**	**GLU**	**43**	10.3	8.7	35	0.3
**bL17**	**GLU**	**49**	8.2	5.7	24	0.3
**bL17**	**ASP**	**58**	6.8	10.0	16	0.4
**bL17**	**ASP**	**72**	9.1	8.6	26	0.4
**bL17**	**GLU**	**74**	7.5	10.4	16	0.5
**bL17**	**GLU**	**82**	2.2	12.9	6	0.4
**bL17**	**ASP**	**106**	13.2	7.5	38	0.3
**bL17**	**GLU**	**114**	4.7	10.4	17	0.3
**bL17**	**ASP**	**117**	2.7	10.2	9	0.3
**bL17**	**GLU**	**120**	1.4	14.9	5	0.3
**uL18**	**ASP**	**2**	24.1	8.2	32	0.8
**uL18**	**GLU**	**20**	14.7	9.7	23	0.6
**uL18**	**GLU**	**46**	5.1	7.7	11	0.5
**uL18**	**GLU**	**55**	12.1	6.2	18	0.7
**uL18**	**GLU**	**60**	7.7	13.7	10	0.8
**uL18**	**ASP**	**69**	6.7	7.9	10	0.7
**uL18**	**GLU**	**80**	2.1	15.8	3	0.7
**uL18**	**GLU**	**84**	1.8	15.6	3	0.6
**uL18**	**ASP**	**89**	4.3	10.7	6	0.7
**uL18**	**ASP**	**93**	13.9	10.2	23	0.6
**uL18**	**ASP**	**108**	8.3	12.2	12	0.7
**uL18**	**GLU**	**112**	3.8	14.8	5	0.8
**bL19**	**GLU**	**9**	8.6	11.8	14	0.6
**bL19**	**GLU**	**11**	3.3	16.8	6	0.5
**bL19**	**ASP**	**16**	1.6	16.5	3	0.5
**bL19**	**ASP**	**24**	17.1	14.6	20	0.9
**bL19**	**GLU**	**27**	23.5	13.4	7	3.4
**bL19**	**GLU**	**34**	35.9	10.4	7	5.1
**bL19**	**GLU**	**44**	26.6	11.2	9	3.0
**bL19**	**GLU**	**68**	42.1	12.8	17	2.5
**bL19**	**GLU**	**71**	16.5	10.0	17	1.0
**bL19**	**ASP**	**82**	18.9	15.1	5	3.8
**bL19**	**GLU**	**102**	39.0	11.3	34	1.1
**bL19**	**GLU**	**112**	39.0	8.9	25	1.6
**bL20**	**ASP**	**49**	13.0	7.1	41	0.3
**bL20**	**GLU**	**89**	6.3	8.9	13	0.5
**bL20**	**ASP**	**91**	7.3	4.7	19	0.4
**bL20**	**ASP**	**97**	4.6	12.2	13	0.4
**bL20**	**ASP**	**102**	0.6	19.3	2	0.3
**bL20**	**GLU**	**111**	2.4	17.5	6	0.4
**bL21**	**GLU**	**16**	18.6	14.7	3	6.2
**bL21**	**GLU**	**23**	8.1	7.5	17	0.5
**bL21**	**ASP**	**26**	10.0	8.7	11	0.9
**bL21**	**GLU**	**31**	1.6	18.2	2	0.8
**bL21**	**GLU**	**34**	3.4	15.6	3	1.1
**bL21**	**GLU**	**37**	4.8	13.9	6	0.8
**bL21**	**GLU**	**62**	1.5	18.3	1	1.5
**bL21**	**GLU**	**70**	12.3	8.7	24	0.5
**bL21**	**ASP**	**95**	15.6	14.9	13	1.2
**uL22**	**GLU**	**2**	6.5	9.7	14	0.5
**uL22**	**ASP**	**22**	5.6	8.5	20	0.3
**uL22**	**ASP**	**34**	4.5	14.5	10	0.5
**uL22**	**GLU**	**52**	6.1	7.8	14	0.4
**uL22**	**GLU**	**59**	4.1	10.3	10	0.4
**uL22**	**ASP**	**62**	4.6	9.3	10	0.5
**uL22**	**ASP**	**65**	2.4	16.2	5	0.5
**uL22**	**ASP**	**67**	4.1	17.5	6	0.7
**uL22**	**ASP**	**68**	4.1	15.5	6	0.7
**uL22**	**ASP**	**77**	15.4	7.9	37	0.4
**uL22**	**GLU**	**78**	14.9	7.4	37	0.4
**uL22**	**ASP**	**94**	18.3	6.1	43	0.4
**uL22**	**ASP**	**109**	3.8	16.3	8	0.5
**uL23**	**GLU**	**4**	15.7	11.5	16	1.0
**uL23**	**GLU**	**5**	18.7	10.1	19	1.0
**uL23**	**GLU**	**18**	11.7	7.0	20	0.6

**uL23**	**GLU**	**25**	11.1	13.4	14	0.8
**uL23**	**ASP**	**37**	18.4	7.2	33	0.6
**uL23**	**GLU**	**42**	17.0	9.1	25	0.7
**uL23**	**GLU**	**52**	2.2	17.8	2	1.1
**uL23**	**GLU**	**54**	10.9	13.8	13	0.8
**uL23**	**GLU**	**56**	16.3	9.9	22	0.7
**uL23**	**ASP**	**79**	15.4	6.4	34	0.5
**uL23**	**GLU**	**89**	5.4	10.5	9	0.6
**uL24**	**ASP**	**8**	3.5	7.8	8	0.4
**uL24**	**ASP**	**9**	4.8	9.4	12	0.4
**uL24**	**GLU**	**10**	3.5	12.8	10	0.4
**uL24**	**ASP**	**18**	12.6	8.4	24	0.5
**uL24**	**GLU**	**37**	5.7	14.6	9	0.6
**uL24**	**GLU**	**60**	8.2	10.3	14	0.6
**uL24**	**GLU**	**62**	10.0	9.5	18	0.6
**uL24**	**ASP**	**81**	10.1	7.1	21	0.5
**uL24**	**GLU**	**88**	5.3	12.9	12	0.4
**uL24**	**ASP**	**89**	5.8	14.0	12	0.5
**uL24**	**GLU**	**101**	4.4	13.5	11	0.4
**bL25**	**GLU**	**7**	3.4	13.9	4	0.8
**bL25**	**GLU**	**11**	5.4	8.5	12	0.5
**bL25**	**GLU**	**35**	4.6	13.1	8	0.6
**bL25**	**GLU**	**41**	6.6	10.0	10	0.7
**bL25**	**ASP**	**43**	9.6	11.2	11	0.9
**bL25**	**ASP**	**45**	11.6	7.5	10	1.2
**bL25**	**GLU**	**55**	6.0	16.4	2	3.0
**bL25**	**GLU**	**59**	0.9	17.5	2	0.5
**bL25**	**ASP**	**76**	7.3	9.8	18	0.4
**bL25**	**ASP**	**90**	6.2	10.9	16	0.4
**bL27**	**ASP**	**15**	20.9	8.0	47	0.4
**bL27**	**GLU**	**17**	21.3	4.7	47	0.5
**bL27**	**GLU**	**29**	10.9	7.0	20	0.5
**bL27**	**ASP**	**56**	18.6	5.8	41	0.5
**bL27**	**ASP**	**64**	9.4	11.0	15	0.6
**bL27**	**GLU**	**70**	12.2	7.4	22	0.6
**bL27**	**GLU**	**83**	10.5	13.1	14	0.8
**bL27**	**GLU**	**85**	5.8	15.8	6	1.0
**bL28**	**GLU**	**41**	9.3	15.2	10	0.9
**bL28**	**GLU**	**43**	12.9	10.0	11	1.2
**bL28**	**ASP**	**60**	10.8	10.7	19	0.6
**bL28**	**ASP**	**65**	8.4	13.9	12	0.7
**bL28**	**GLU**	**70**	11.2	12.1	15	0.7
**bL28**	**GLU**	**76**	17.0	9.3	25	0.7
**uL29**	**GLU**	**5**	6.4	10.2	11	0.6
**uL29**	**GLU**	**8**	2.4	12.1	4	0.6
**uL29**	**GLU**	**12**	4.6	16.3	4	1.1
**uL29**	**GLU**	**13**	1.8	18.2	3	0.6
**uL29**	**GLU**	**17**	2.4	14.8	6	0.4
**uL29**	**GLU**	**24**	5.2	14.8	12	0.4
**uL29**	**ASP**	**49**	12.7	8.4	25	0.5
**uL29**	**GLU**	**59**	11.0	9.0	17	0.6
**uL30**	**GLU**	**37**	7.1	9.6	12	0.6
**uL30**	**GLU**	**39**	4.2	8.9	10	0.4
**uL30**	**ASP**	**40**	3.7	12.3	9	0.4
**uL30**	**GLU**	**58**	2.1	18.7	4	0.5
**uL30**	**GLU**	**59**	0.6	18.9	1	0.6
**bL31**	**ASP**	**4**	16.1	10.2	12	1.3
**bL31**	**GLU**	**10**	7.5	15.7	5	1.5
**bL31**	**GLU**	**11**	4.4	17.3	3	1.5
**bL32**	**ASP**	**20**	11.7	7.9	39	0.3
**bL32**	**ASP**	**31**	5.1	13.1	11	0.5
**bL32**	**GLU**	**36**	3.9	15.7	7	0.6
**bL32**	**ASP**	**46**	3.4	14.6	9	0.4
**uL33**	**GLU**	**7**	8.8	8.4	16	0.5
**uL33**	**GLU**	**32**	3.4	10.4	7	0.5
**uL33**	**GLU**	**35**	10.9	10.1	17	0.6
**uL33**	**ASP**	**40**	16.0	9.4	24	0.7
**uL33**	**GLU**	**51**	6.9	14.5	14	0.5
**bL35**	**ASP**	**54**	11.0	7.3	33	0.3
**bL36**	**ASP**	**20**	17.7	4.8	34	0.5
**bL36**	**GLU**	**30**	34.0	7.8	27	1.3

Table S5 (B)

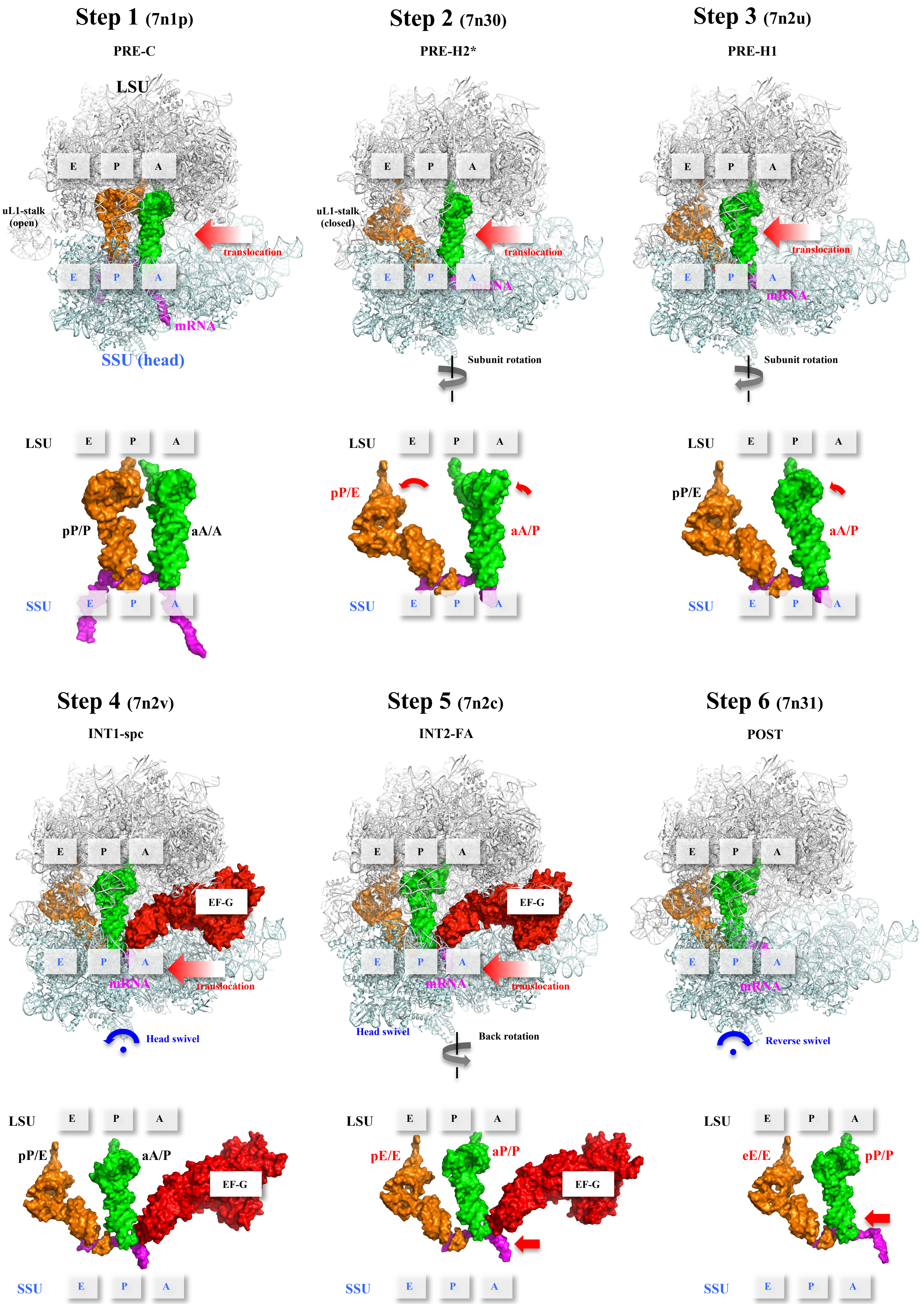

Step 1 (7n1p)
PRE-C
LSU
E P A
uL1-stalk (open)
translocation
E P A
mRNA
SSU (head)
Step 2 (7n30)
PRE-H2*
uL1-stalk (closed)
translocation
mRNA
Subunit rotation
Step 3 (7n2u)
PRE-H1
translocation
mRNA
Subunit rotation
LSU
SSU
pP/P
aA/A
pP/E
aA/P
Step 4 (7n2v)
INT1-spc
EF-G
mRNA
translocation
Head swivel
Step 5 (7n2c)
INT2-FA
Back rotation
Step 6 (7n31)
POST
Reverse swivel
pE/E
aP/P
eE/E

Figure S1

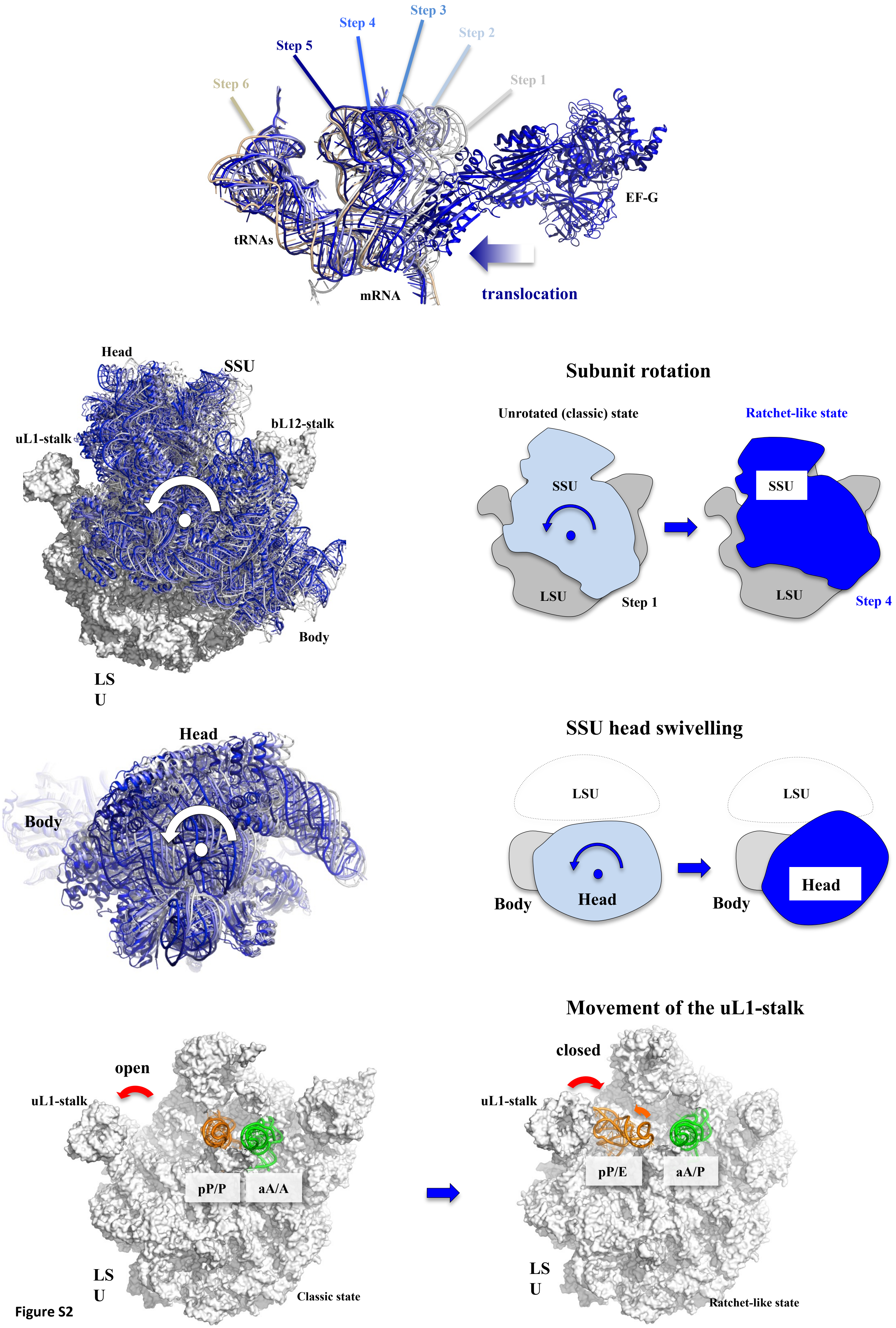

Step 6
Step 5
Step 4
Step 3
Step 2
Step 1
EF-G
tRNAs
mRNA
translocation
Head
SSU
uL1-stalk
bL12-stalk
Body
LS
U
Subunit rotation
Unrotated (classic) state
Ratchet-like state
SSU
LSU
Step 1
SSU
LSU
Step 4
Head
Body
SSU head swivelling
LSU
Body
Head
LSU
Body
Head
Movement of the uL1-stalk
open
uL1-stalk
pP/P
aA/A
LS
U
Classic state
closed
uL1-stalk
pP/E
aA/P
LS
U
Ratchet-like state

Figure S2

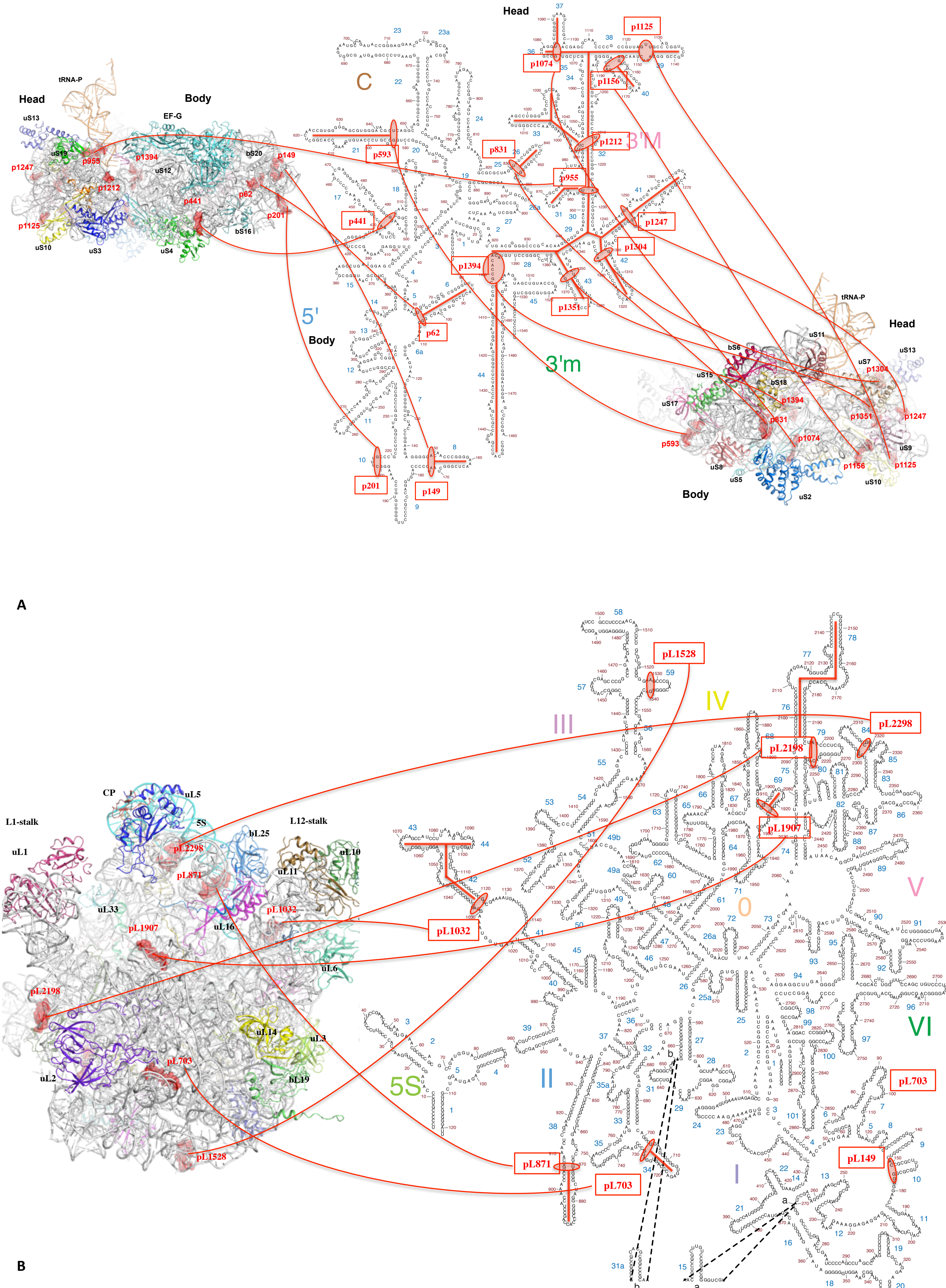
Head
Body
tRNA-P
EF-G
uS13
uS19
p1247
p955
p1394
uS12
bS20
p149
p1212
p441
p62
p201
p1125
bS16
uS10
uS3
uS4
C
3'M
5'
3'm
p593
p831
p1074
p1156
p1304
p1351
p441
p62
p201
p149
uS11
bS6
uS7
uS15
bS18
uS17
uS9
uS8
uS5
uS2
A
CP
uL5
5S
L1-stalk
L12-stalk
uL1
bL25
uL10
uL11
uL33
pL2298
pL871
pL1032
uL16
pL1907
uL6
pL2198
uL14
uL3
uL2
pL703
bL19
pL1528
III
IV
V
VI
0
I
II
5S
pL149
B

Figure S3

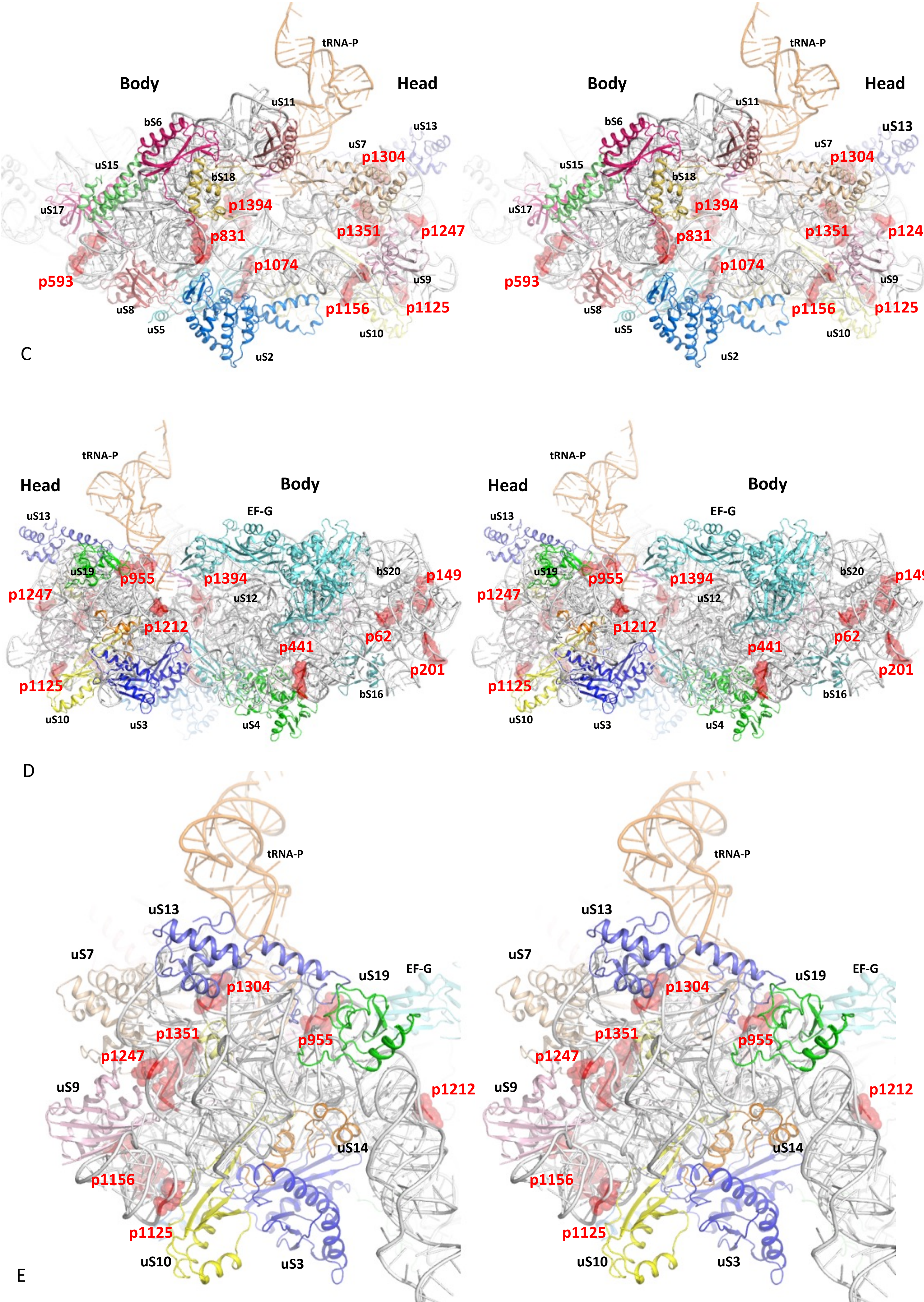

Figure S3 (continued)

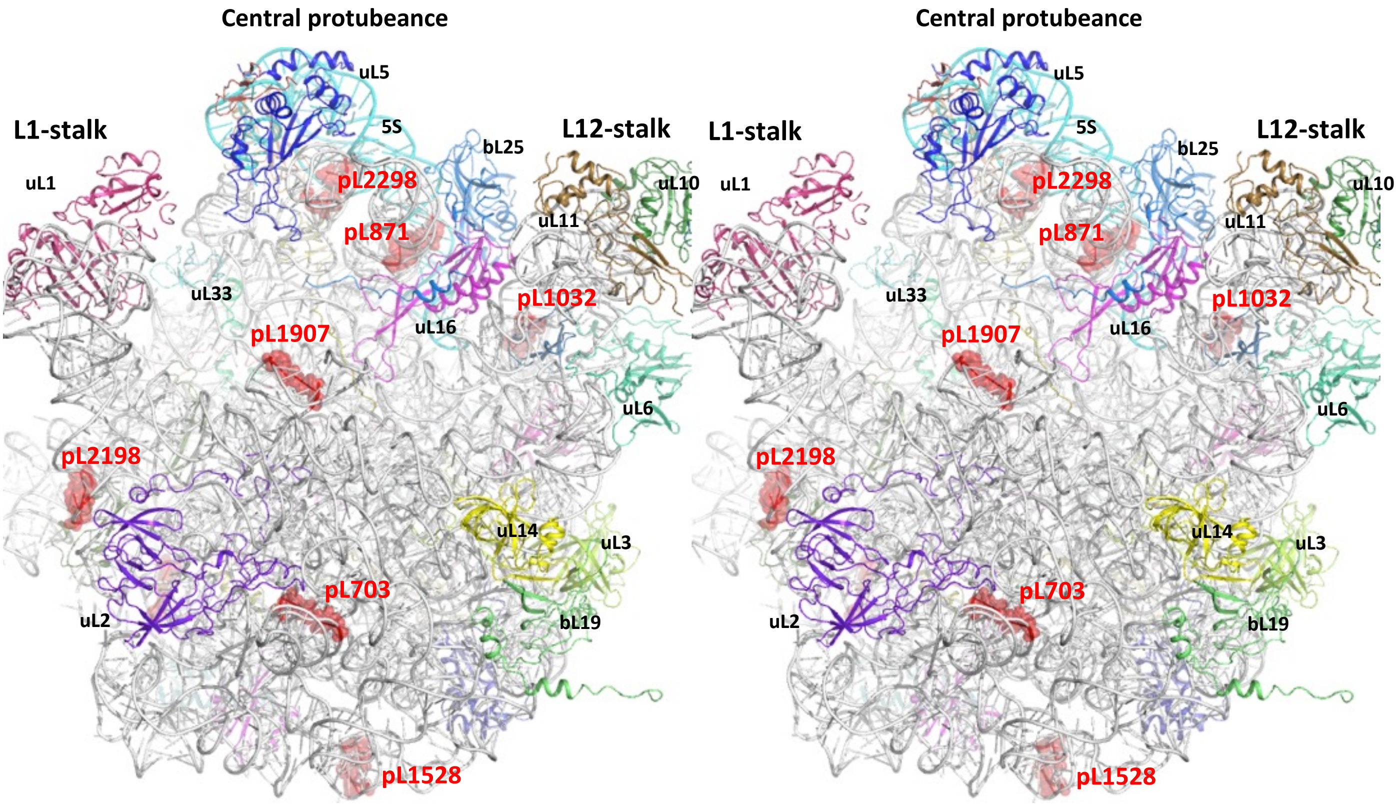

F

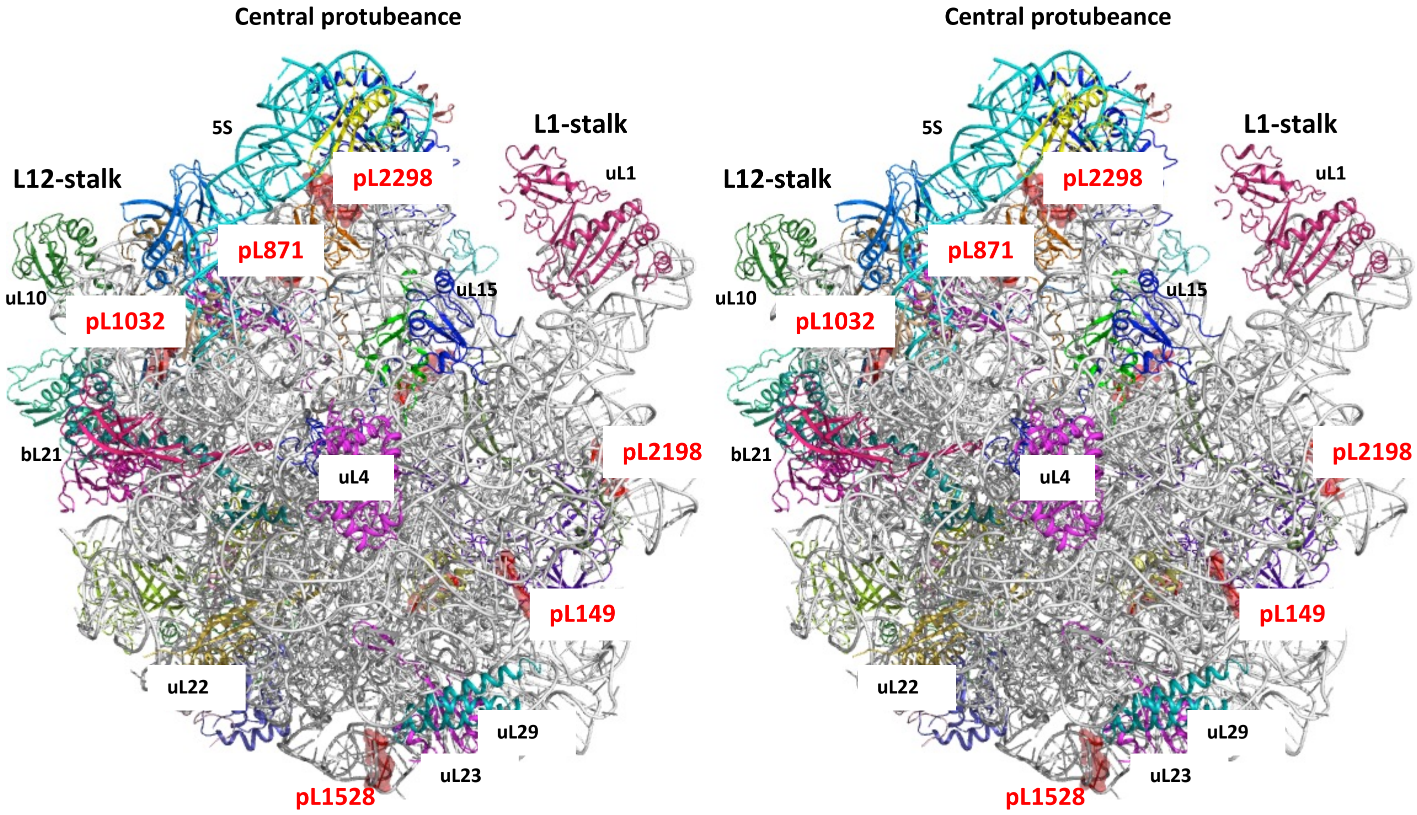

G

Figure S3 (continued)

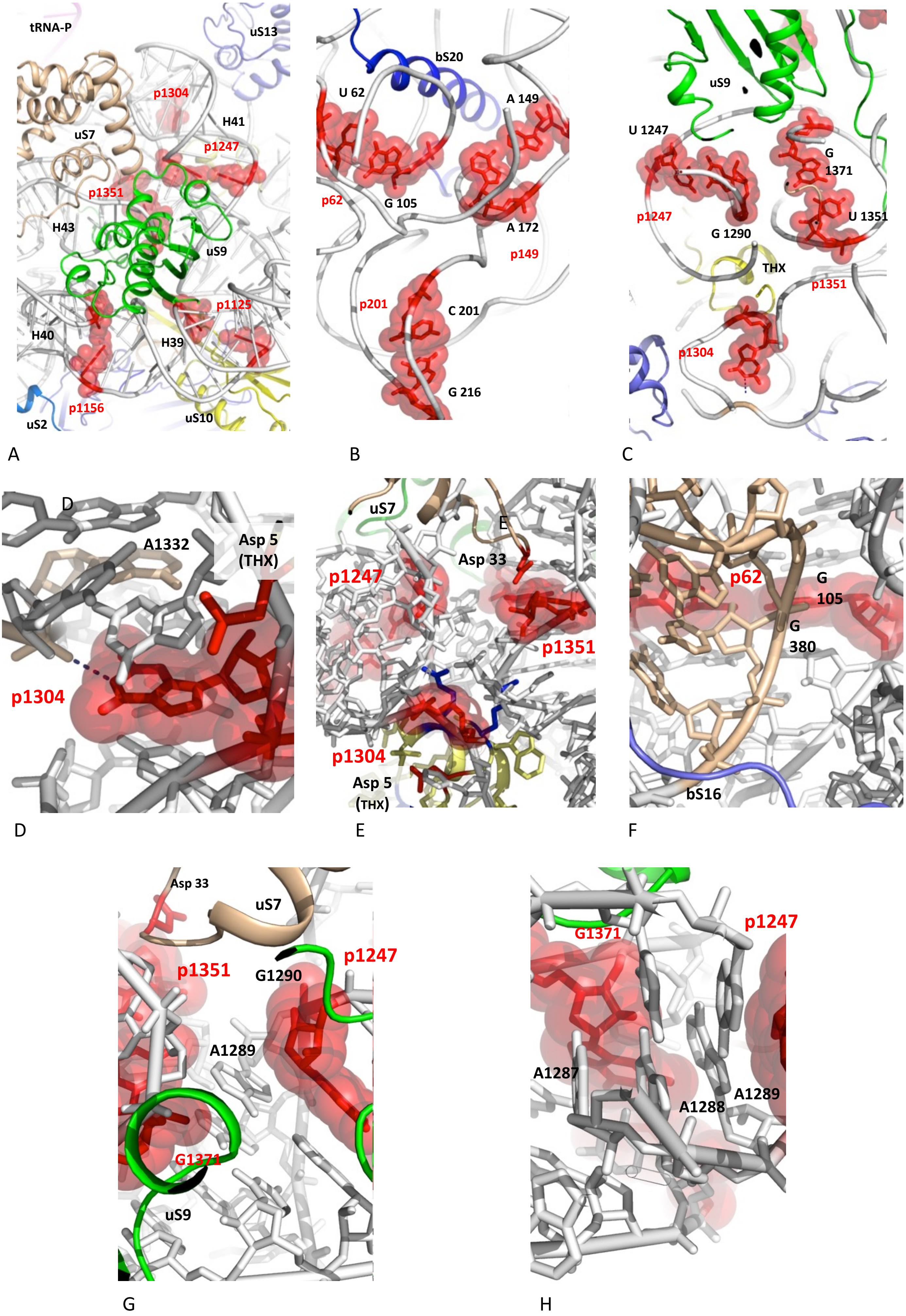

tRNA-P
uS13
p1304
H41
uS7
p1247
p1351
H43
uS9
p1125
H40
H39
p1156
uS2
uS10
A
bS20
U 62
A 149
p62
G 105
A 172
p149
p201
C 201
G 216
B
uS9
U 1247
G
1371
p1247
U 1351
G 1290
THX
p1351
p1304
C
D
A1332
Asp 5
(THX)
p1304
D
uS7
E
Asp 33
p1247
p1351
p1304
Asp 5
(THX)
E
p62
G
105
G
380
bS16
F
Asp 33
uS7
p1351
p1247
G1290
A1289
G1371
uS9
G
G1371
p1247
A1287
A1288
A1289
H

Figure S4

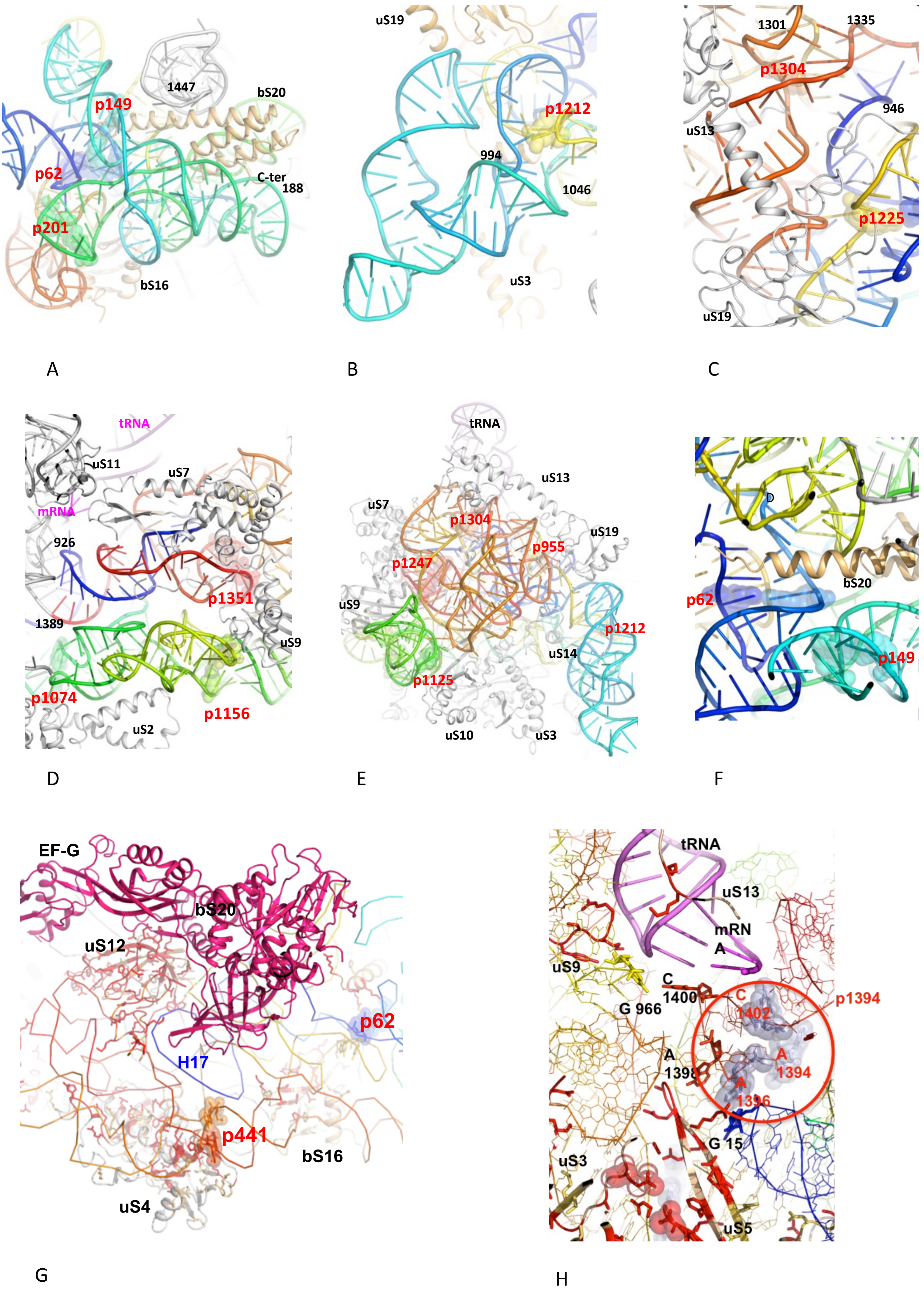
p149
1447
bS20
p62
C-ter
188
p201
bS16
A
uS19
p1212
994
1046
uS3
B
1301
1335
p1304
946
uS13
p1225
uS19
C
tRNA
uS11
uS7
mRNA
926
p1351
1389
uS9
p1074
p1156
uS2
D
tRNA
uS13
uS7
p1304
uS19
p955
p1247
uS9
p1212
uS14
p1125
uS10
uS3
E
D
bS20
p62
p149
F
EF-G
bS20
uS12
p62
H17
p441
bS16
uS4
G
tRNA
uS13
mRN
A
uS9
C
1400
G 966
C
1402
p1394
A
1394
A
1398
A
1396
G 15
uS3
uS5
H

Figure S5

Major centres of motion in the translocation context

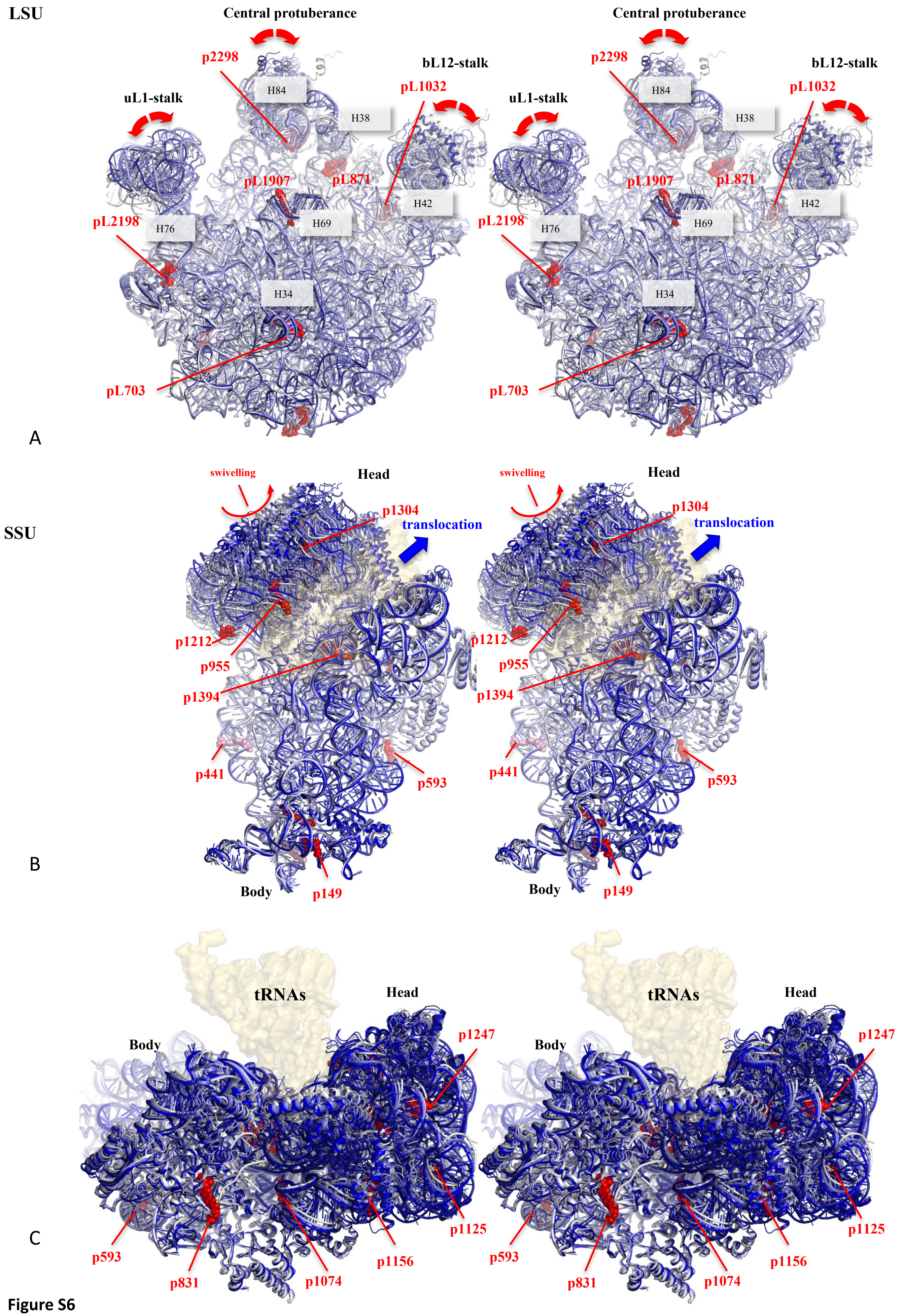

Figure S6

Internal motions of the head during swivelling

Super-imposition of the head domain (residues 932-1385)

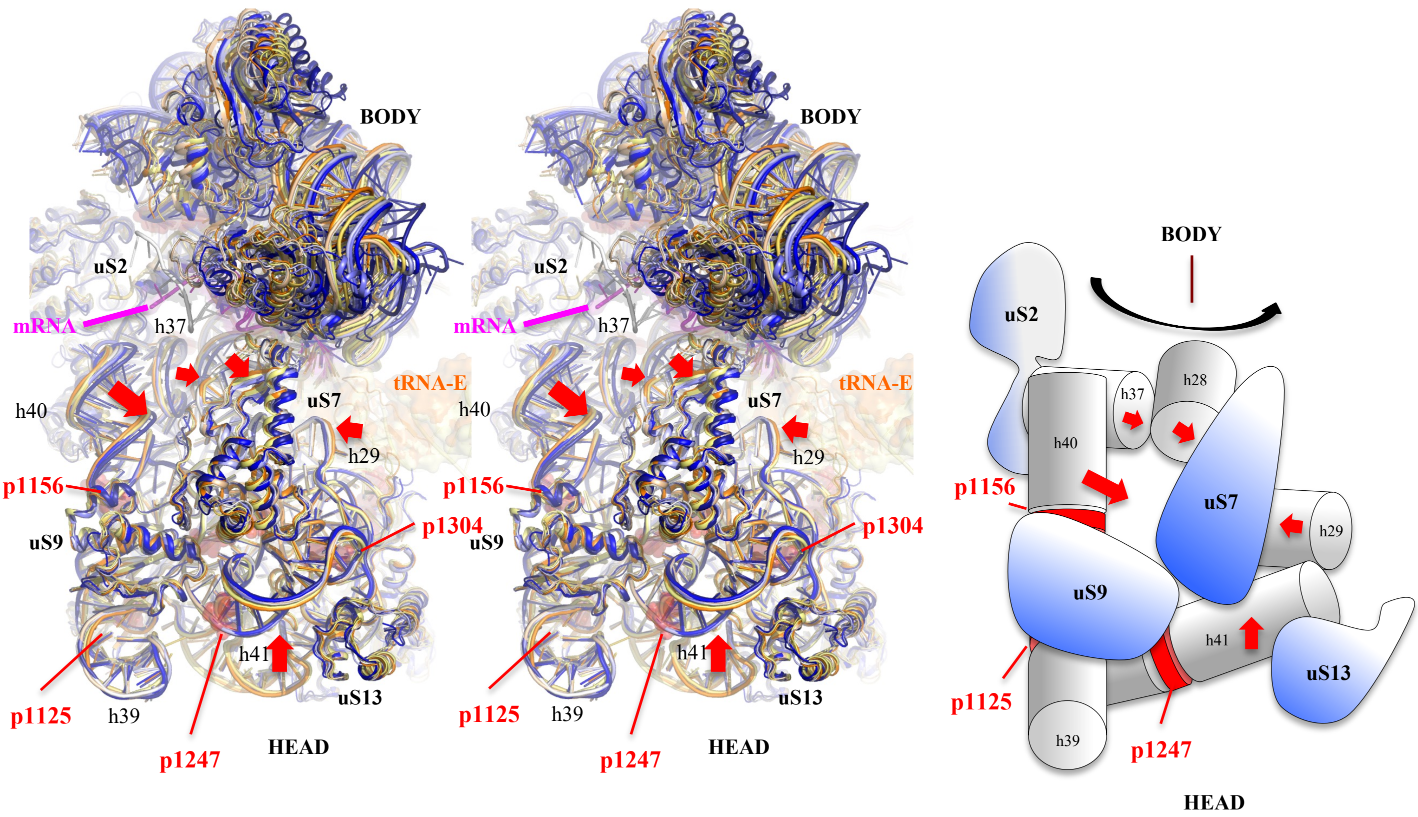

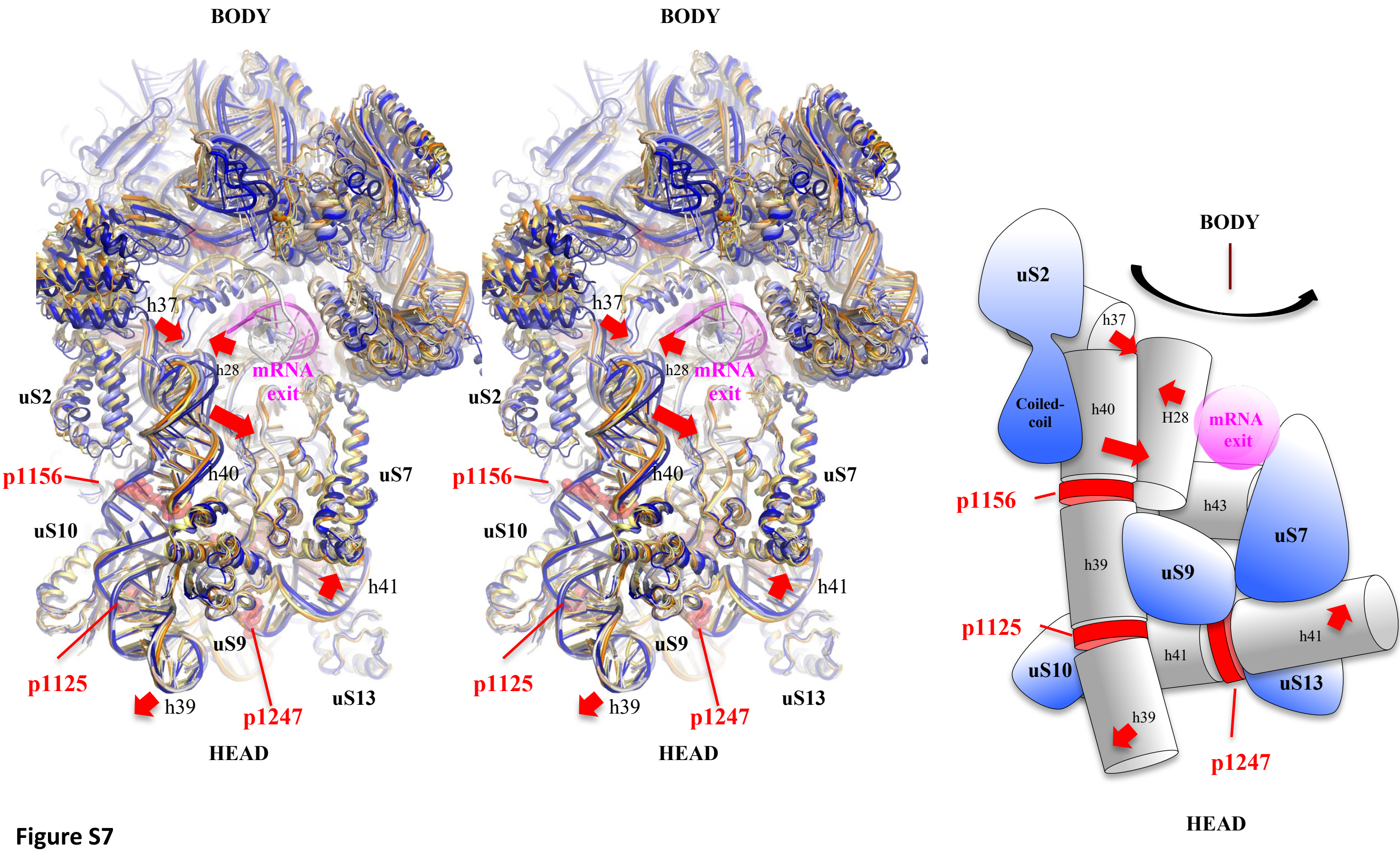

Figure S7

Internal motions of the head during swivelling

Super-imposition of the head domain (residues 932-1385)

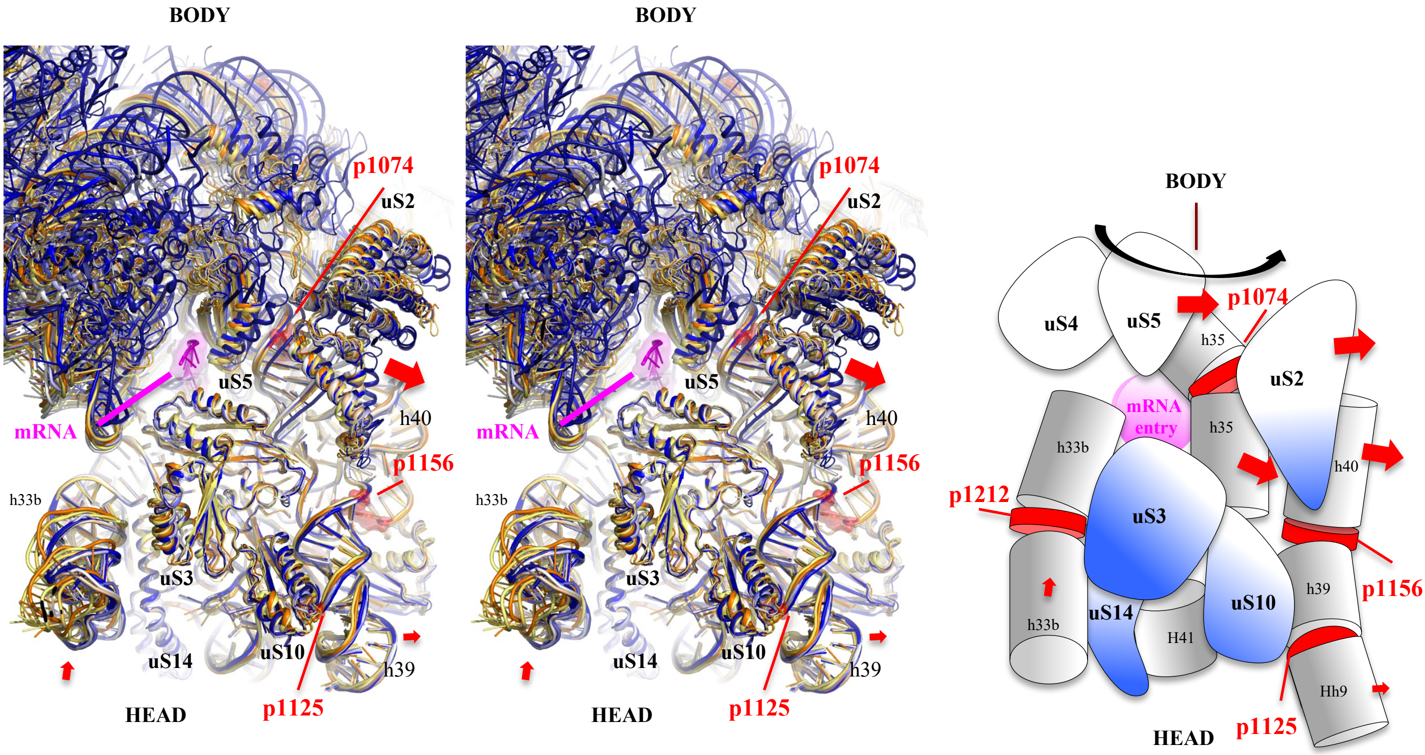

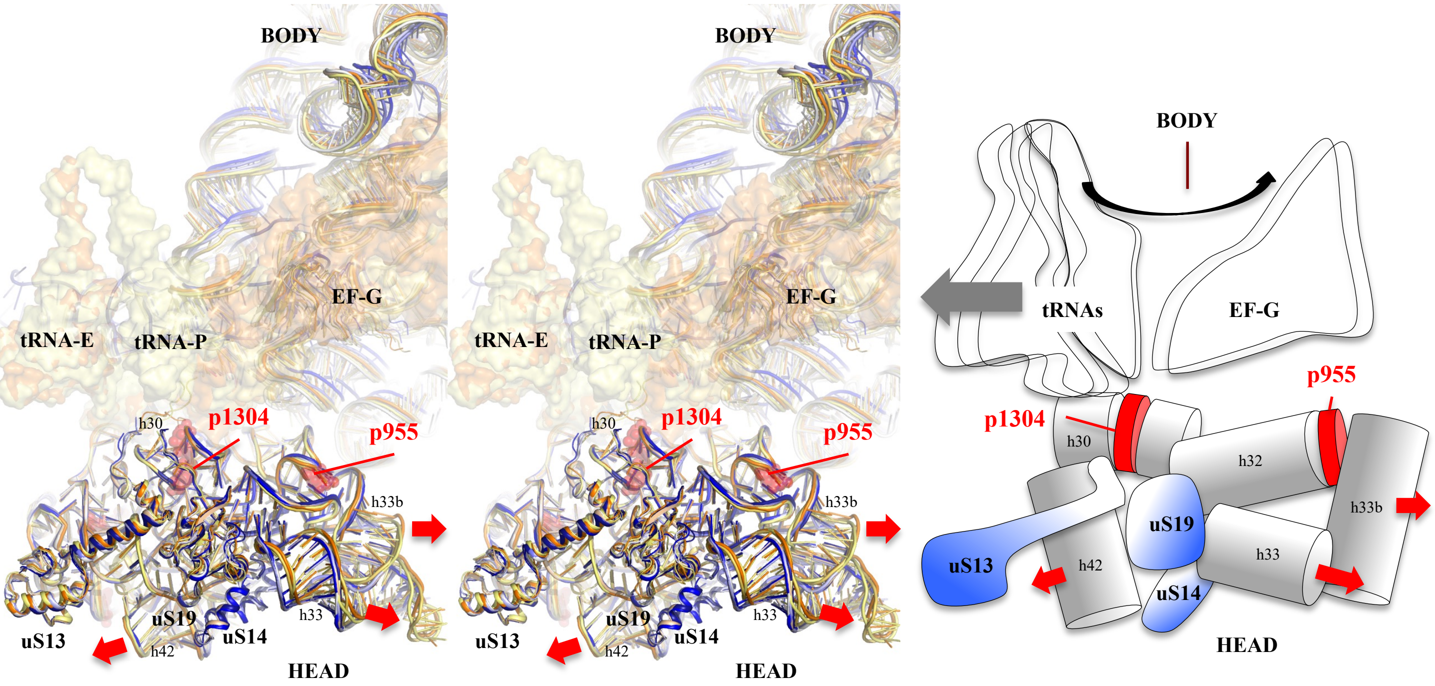

Figure S7 (continued)

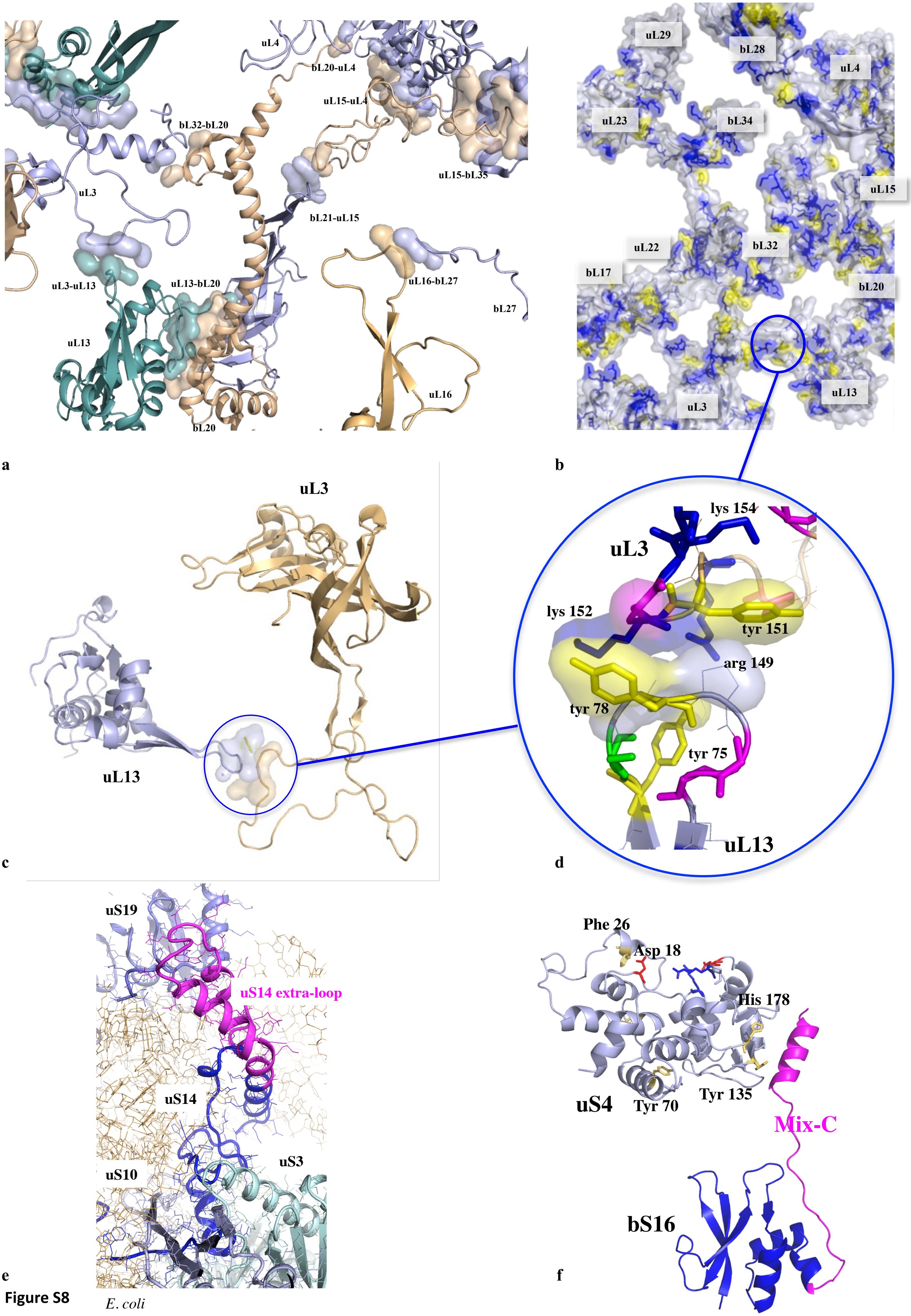

uL4
bL20-uL4
uL15-uL4
bL32-bL20
uL15-bL35
uL3
bL21-uL15
uL3-uL13
uL13-bL20
uL16-bL27
bL27
uL13
uL16
bL20
a
uL29
bL28
uL4
uL23
bL34
uL15
uL22
bL32
bL17
bL20
uL13
uL3
b
uL3
uL13
c
lys 154
uL3
lys 152
tyr 151
arg 149
tyr 78
tyr 75
uL13
d
uS19
uS14 extra-loop
uS14
uS3
uS10
e
E. coli
Phe 26
Asp 18
His 178
uS4
Tyr 70
Tyr 135
Mix-C
bS16
f

Figure S8

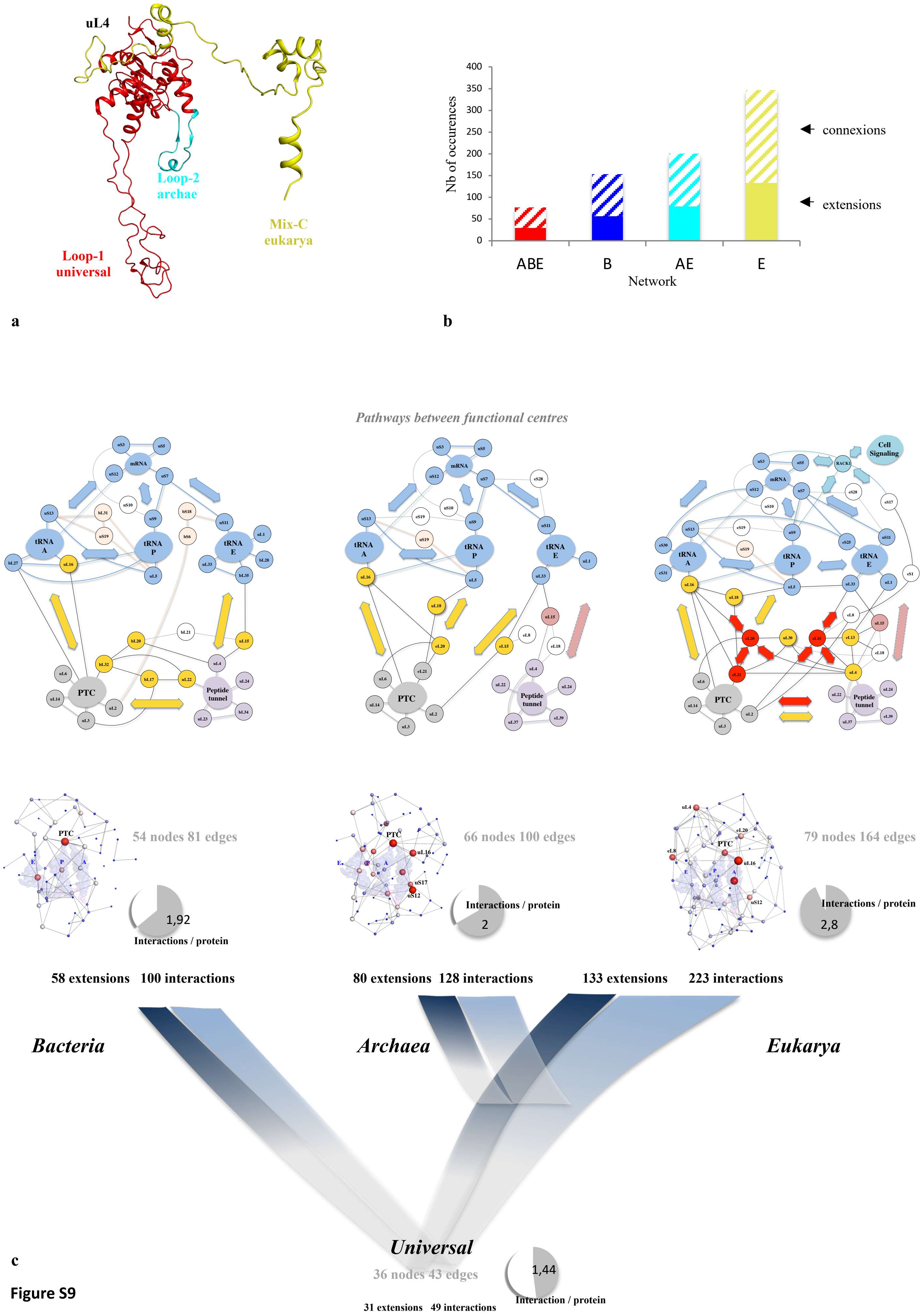

uL4
Loop-2 archae
Mix-C eukarya
Loop-1 universal
a
Nb of occurences
400
350
300
250
200
150
100
50
0
connexions
extensions
ABE
B
AE
E
Network
b
Pathways between functional centres
Cell Signaling
mRNA
tRNA A
tRNA P
tRNA E
PTC
Peptide tunnel
54 nodes 81 edges
1,92
Interactions / protein
66 nodes 100 edges
Interactions / protein
2
79 nodes 164 edges
Interactions / protein
2,8
58 extensions 100 interactions
80 extensions 128 interactions
133 extensions 223 interactions
Bacteria
Archaea
Eukarya
Universal
36 nodes 43 edges
1,44
Interaction / protein
31 extensions 49 interactions
c

Figure S9

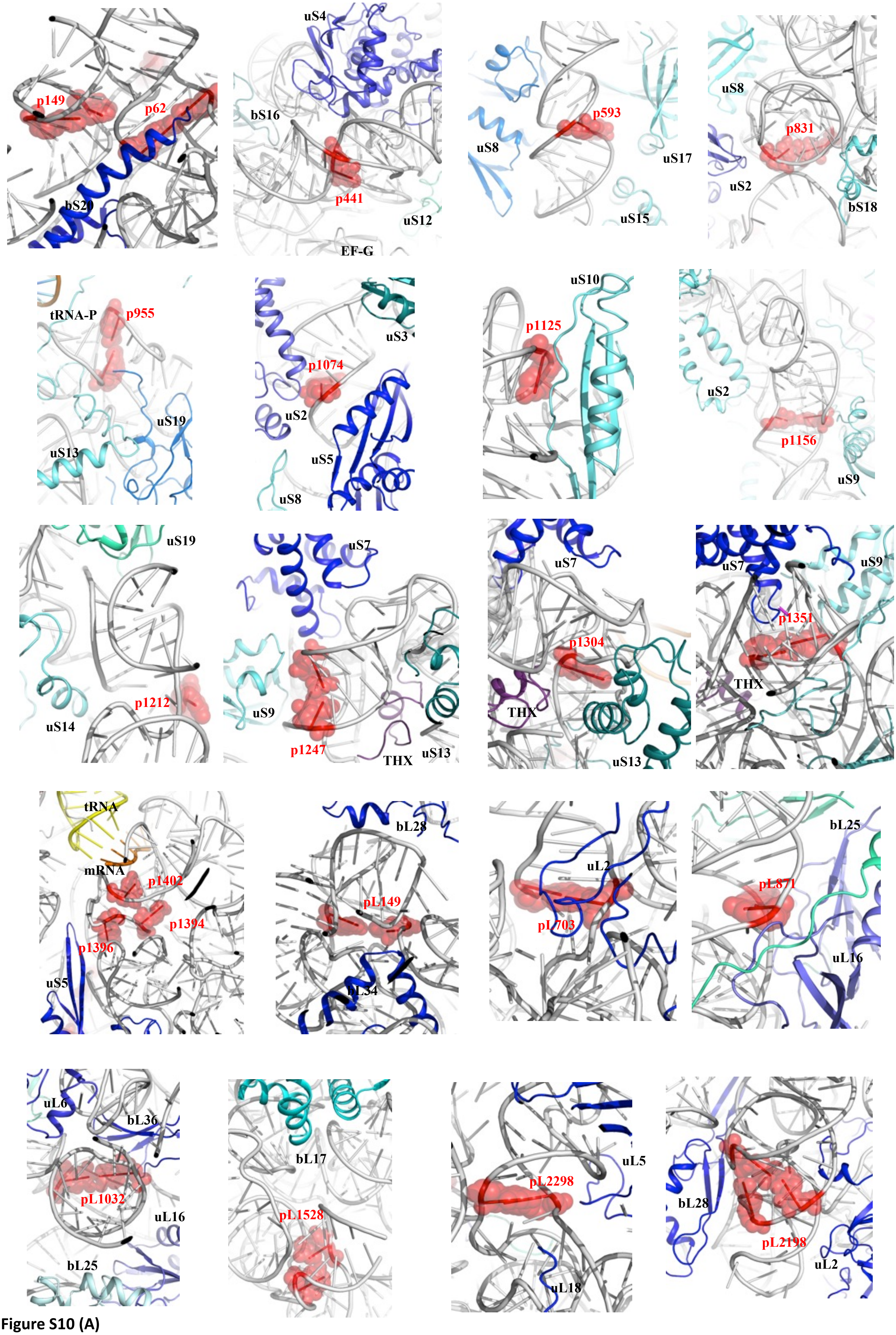
p149
p62
bS20
uS4
bS16
p441
uS12
EF-G
p593
uS8
uS17
uS15
uS8
p831
uS2
bS18
tRNA-P
p955
uS19
uS13
uS3
p1074
uS2
uS5
uS8
uS10
p1125
uS2
p1156
uS9
uS19
p1212
uS14
uS7
uS9
p1247
THX
uS13
uS7
p1304
THX
uS13
uS7
uS9
p1351
THX
tRNA
mRNA
p1402
p1394
p1396
uS5
bL28
pL149
bL34
uL2
pL703
bL25
pL871
uL16
uL6
bL36
pL1032
uL16
bL25
bL17
pL1528
uL5
pL2298
uL18
bL28
pL2198
uL2

Figure S10 (A)

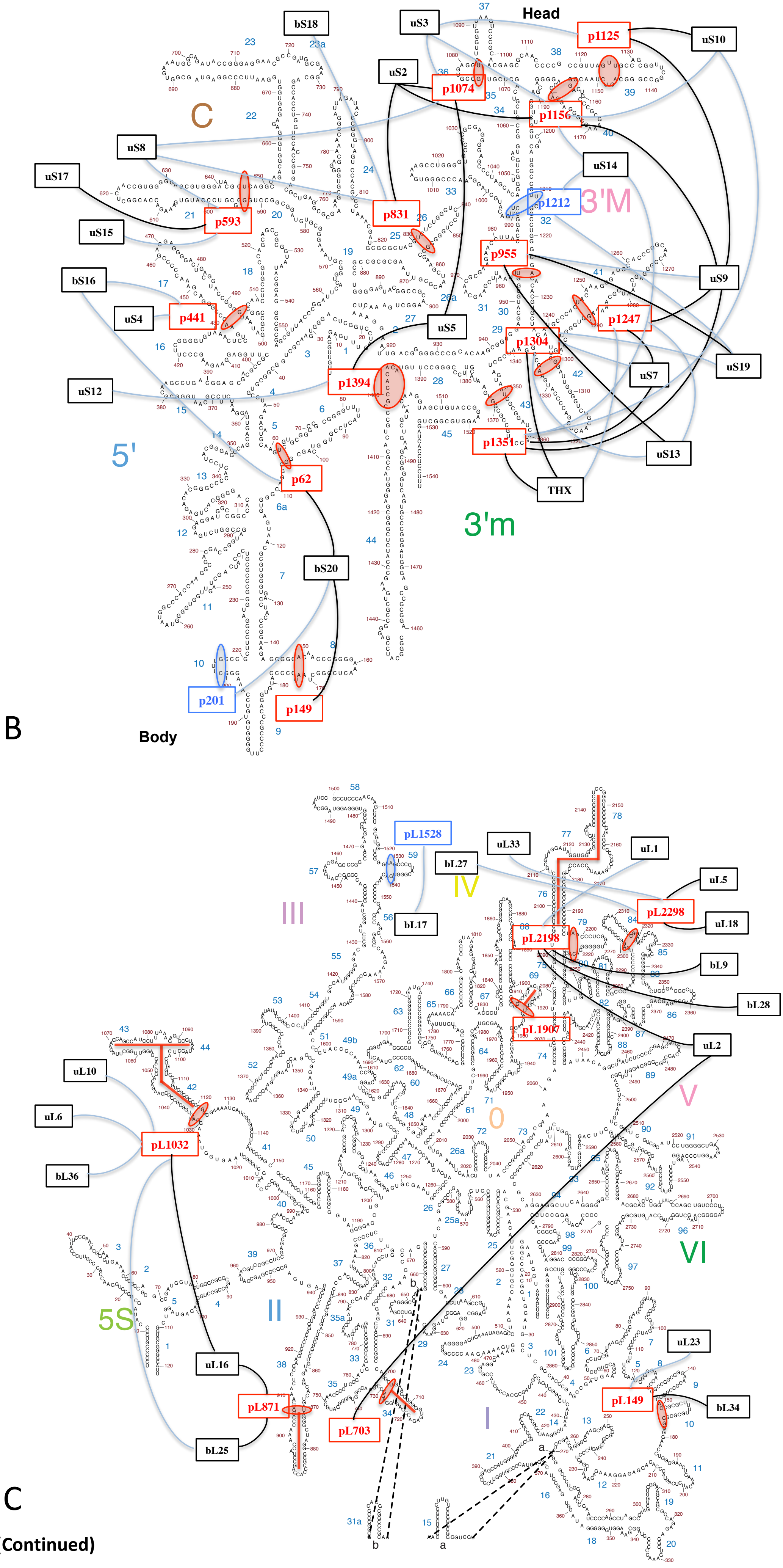

Figure S10 (Continued)

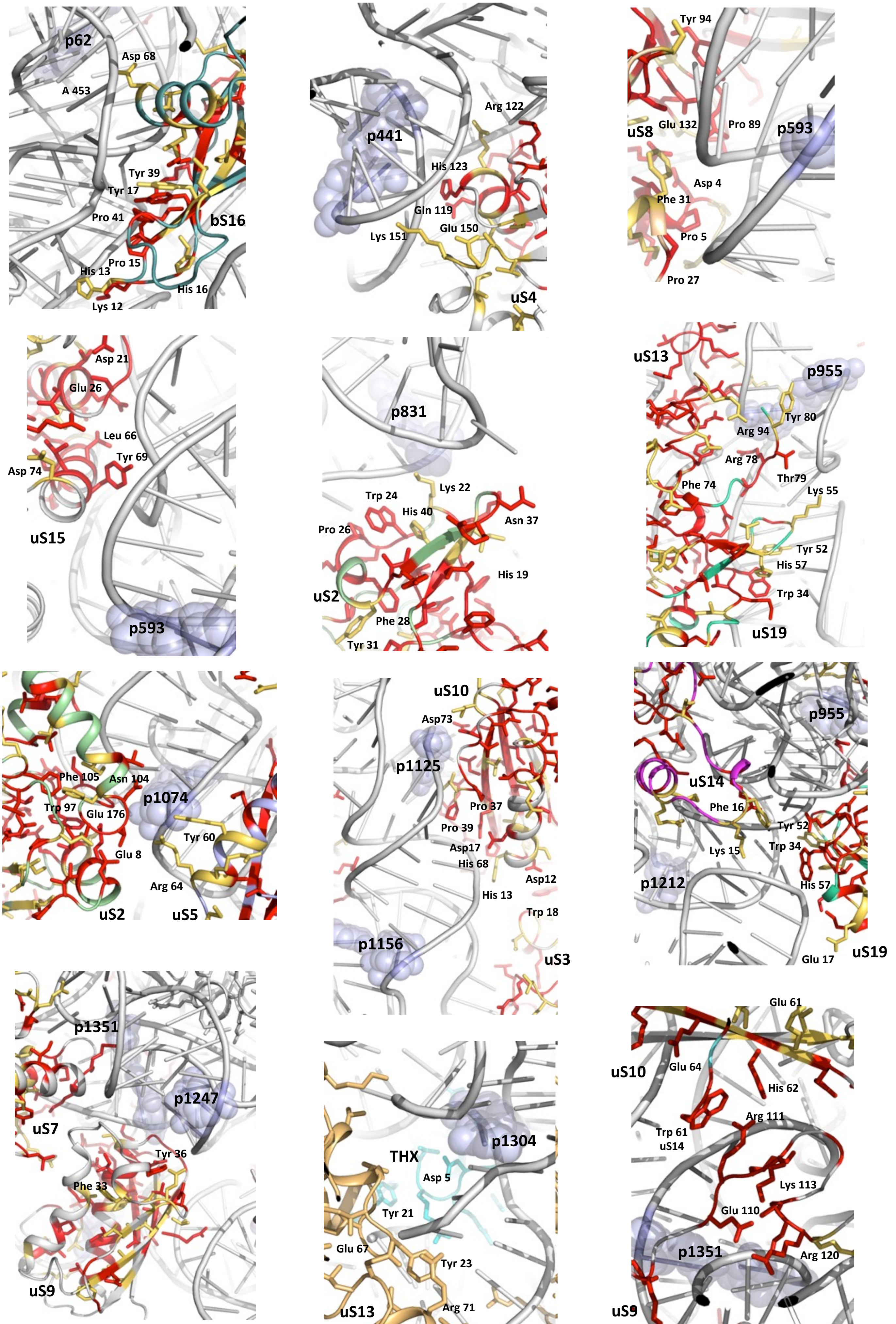

p62
Asp 68
A 453
Tyr 39
Tyr 17
Pro 41
bS16
His 13
Pro 15
His 16
Lys 12
p441
Arg 122
His 123
Gln 119
Glu 150
Lys 151
uS4
Tyr 94
uS8
Glu 132
Pro 89
p593
Asp 4
Phe 31
Pro 5
Pro 27
Asp 21
Glu 26
Leu 66
Tyr 69
Asp 74
uS15
p593
p831
Lys 22
Trp 24
His 40
Asn 37
Pro 26
His 19
uS2
Phe 28
Tyr 31
uS13
p955
Tyr 80
Arg 94
Arg 78
Thr79
Phe 74
Lys 55
Tyr 52
His 57
Trp 34
uS19
Phe 105
Asn 104
p1074
Trp 97
Glu 176
Tyr 60
Glu 8
Arg 64
uS2
uS5
uS10
Asp73
p1125
Pro 37
Pro 39
Asp17
His 68
Asp12
His 13
Trp 18
p1156
uS3
p955
uS14
Phe 16
Tyr 52
Lys 15
Trp 34
p1212
His 57
Glu 17
uS19
p1351
p1247
uS7
Tyr 36
Phe 33
uS9
p1304
THX
Asp 5
Tyr 21
Glu 67
Tyr 23
Arg 71
uS13
Glu 61
uS10
Glu 64
His 62
Arg 111
Trp 61
uS14
Lys 113
Glu 110
p1351
Arg 120
uS9

Figure S11

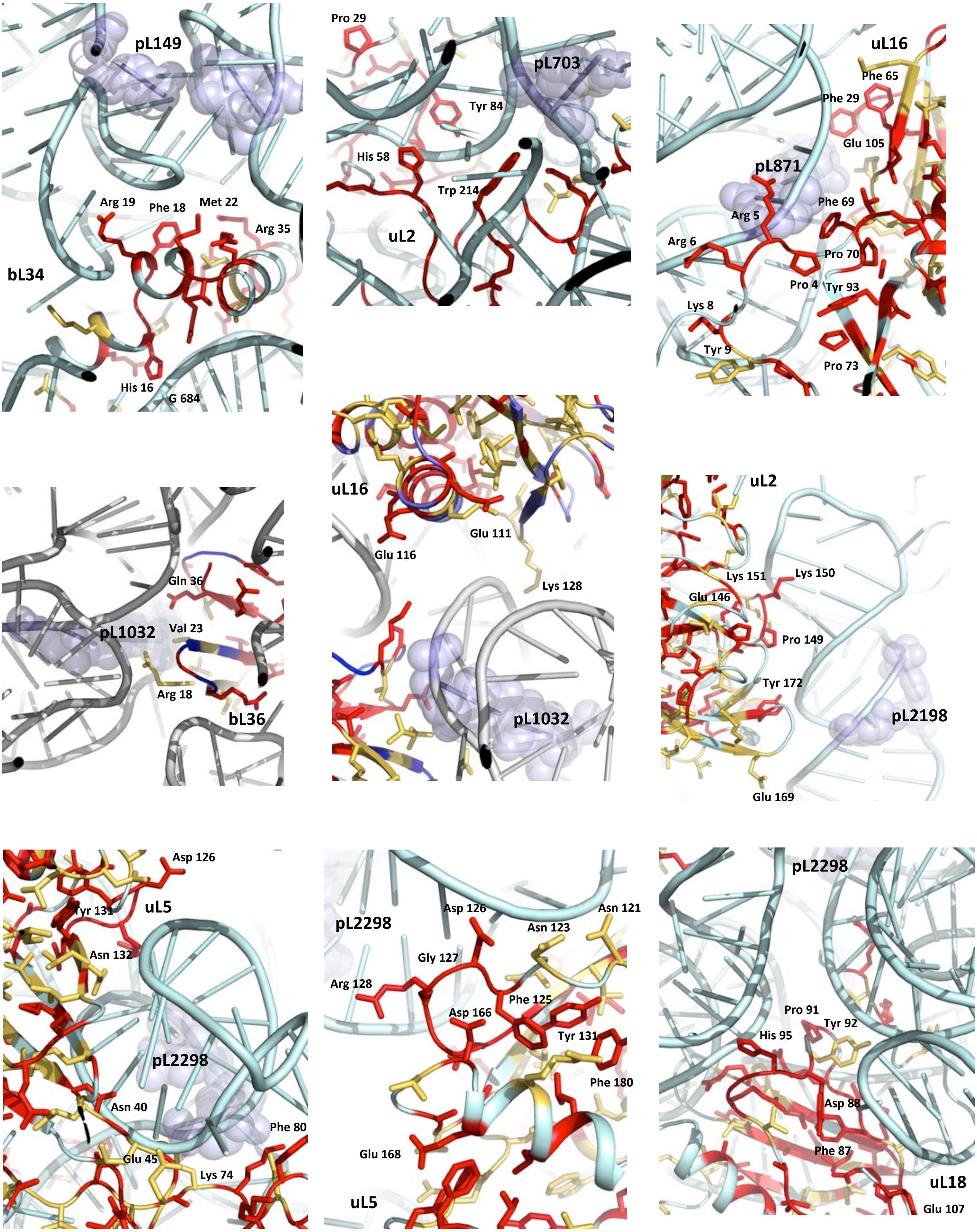

Figure S11 (continued)

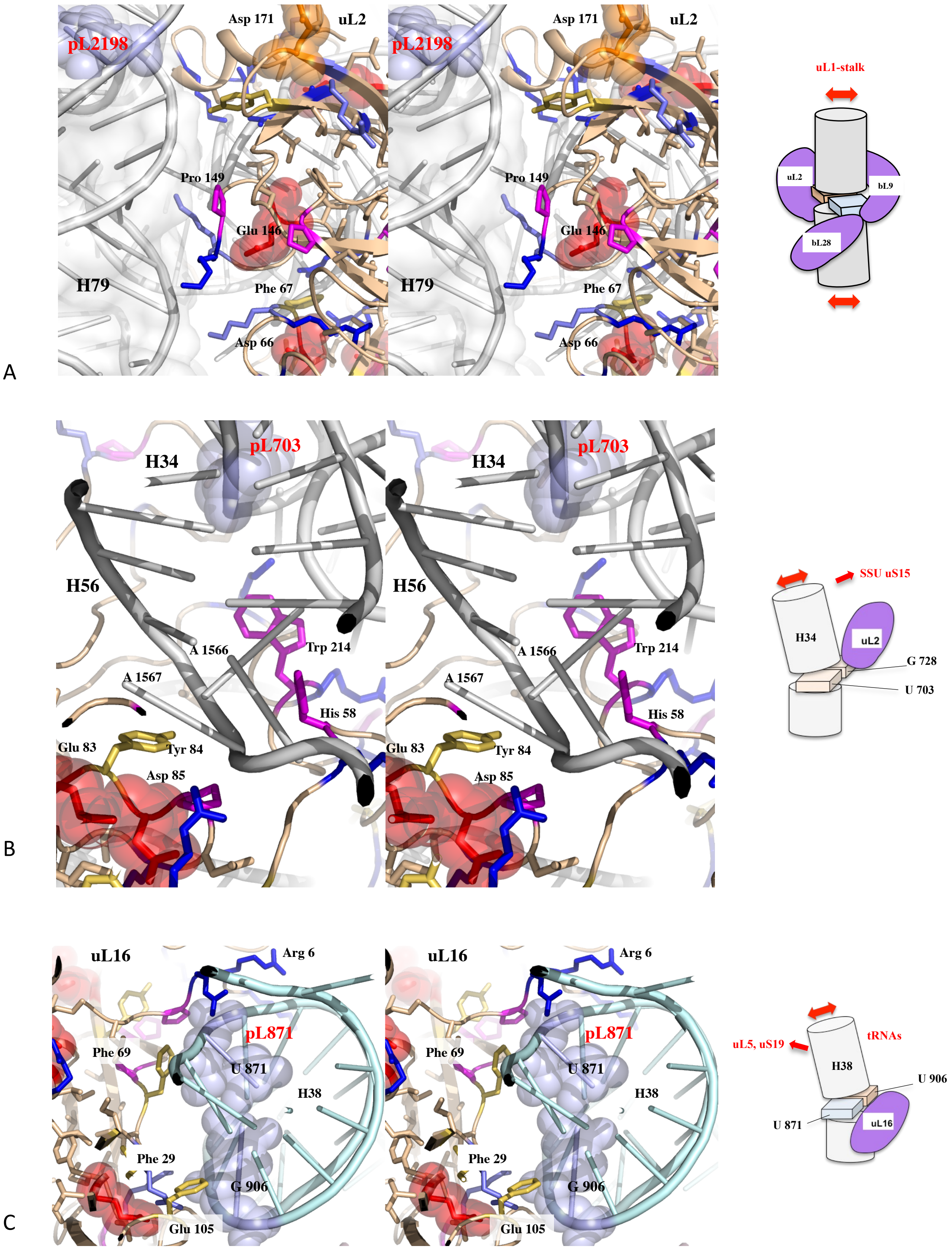
A
pL2198
Asp 171
uL2
Pro 149
Glu 146
H79
Phe 67
Asp 66
uL1-stalk
uL2
bL9
bL28
B
pL703
H34
H56
A 1566
A 1567
Trp 214
His 58
Glu 83
Tyr 84
Asp 85
SSU uS15
H34
uL2
G 728
U 703
C
uL16
Arg 6
pL871
Phe 69
U 871
H38
Phe 29
G 906
Glu 105
tRNAs
uL5, uS19
H38
U 906
U 871
uL16

Figure S12

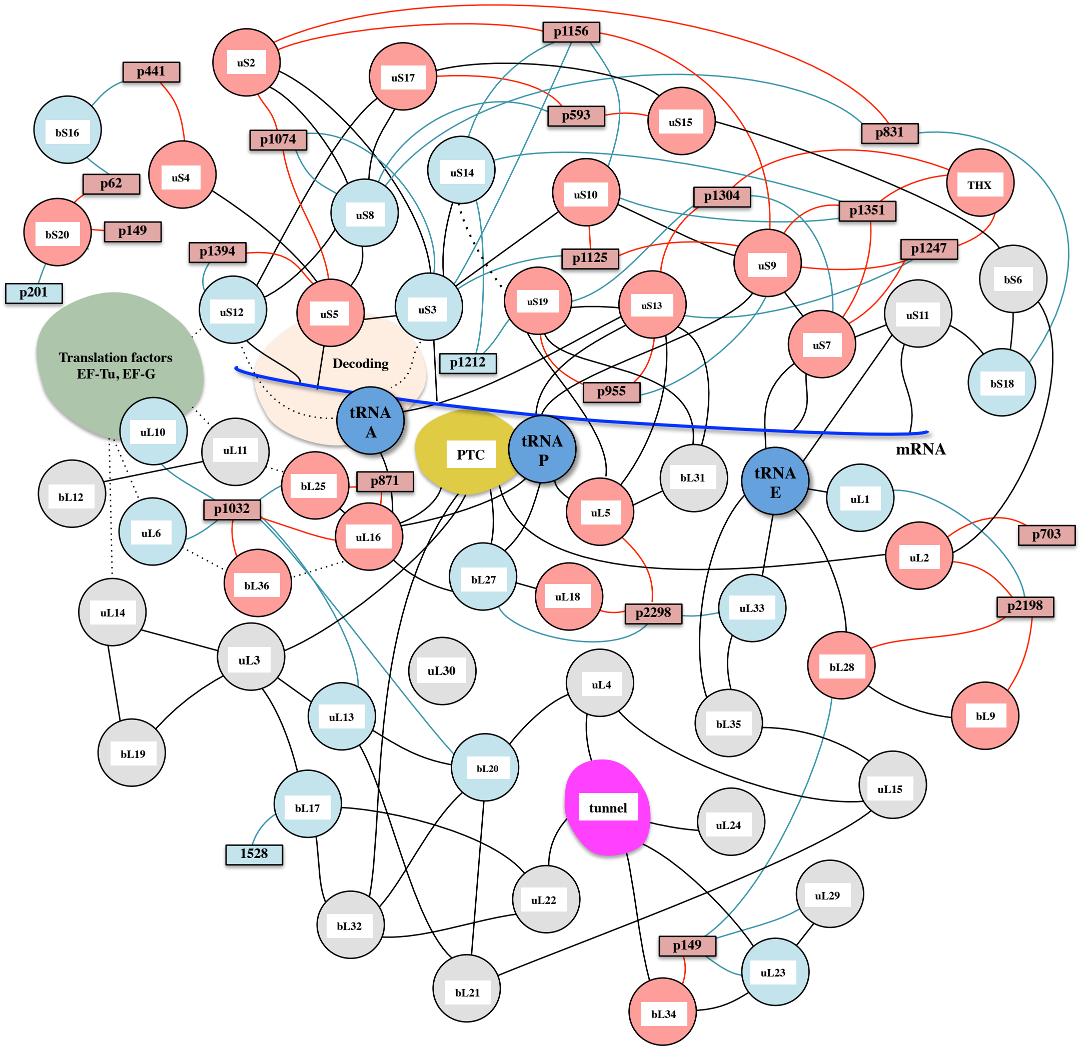

A

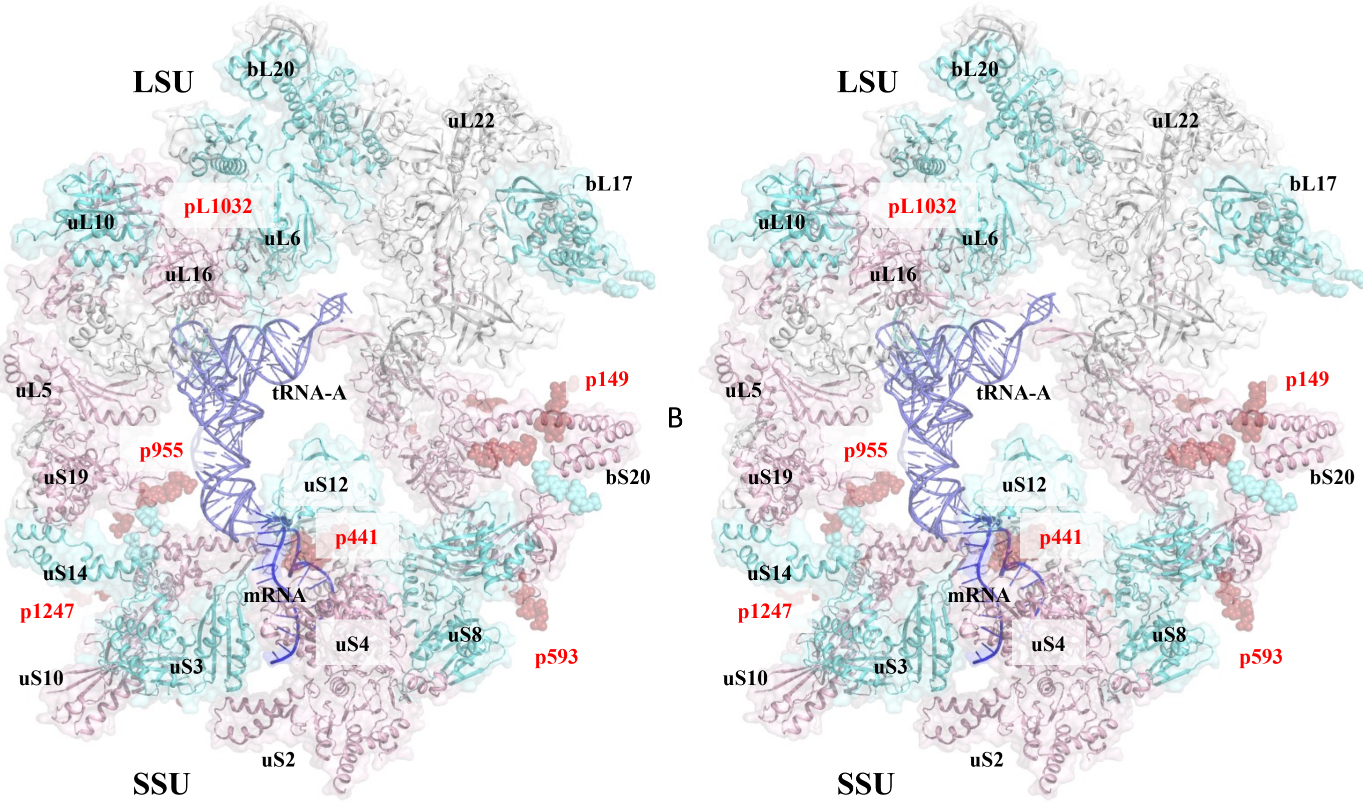

B

Figure S13

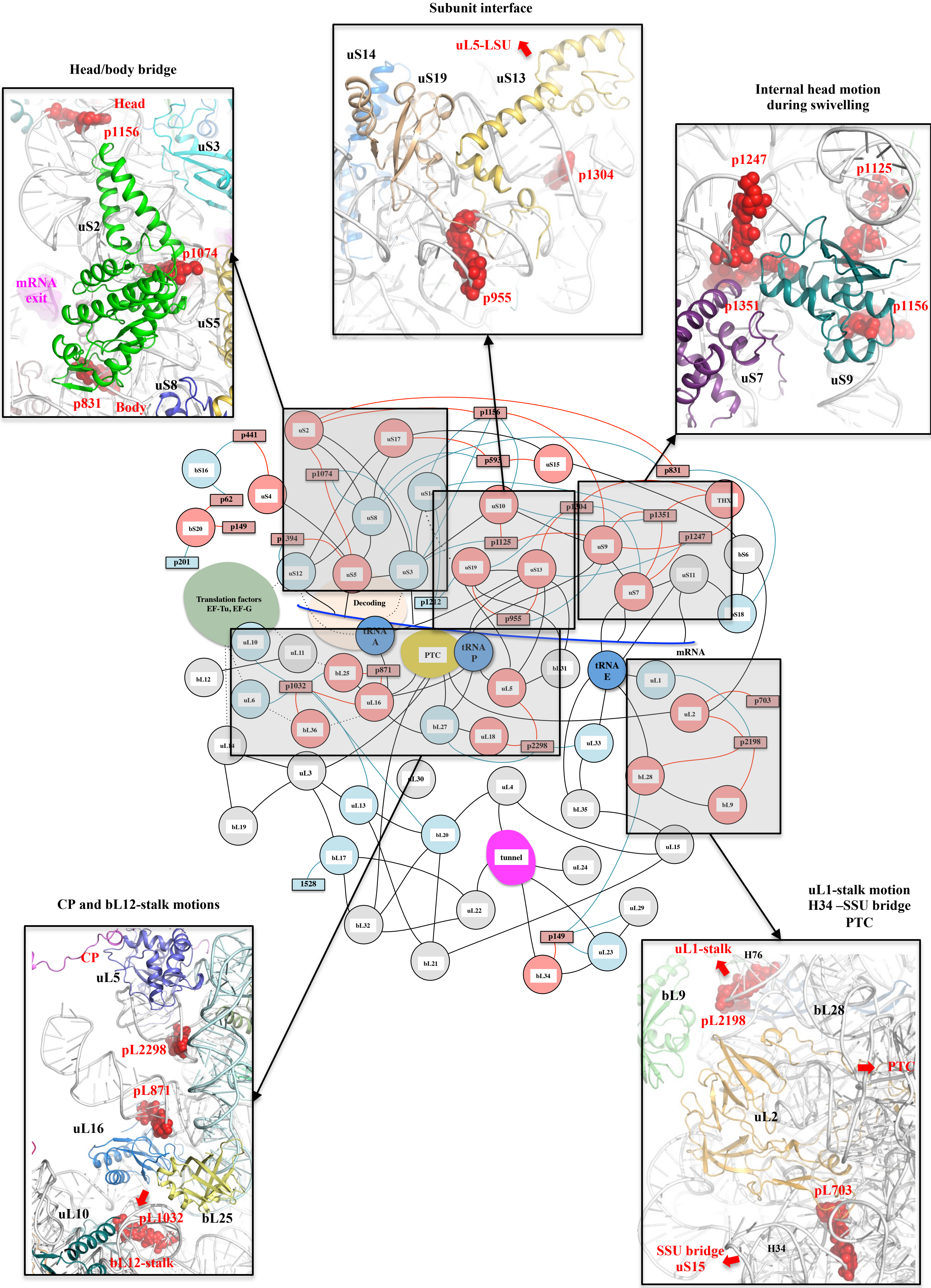

Subunit interface
Head/body bridge
Internal head motion during swivelling
CP and bL12-stalk motions
uL1-stalk motion H34 –SSU bridge PTC
Translation factors EF-Tu, EF-G
Decoding
mRNA
tunnel

Figure S14

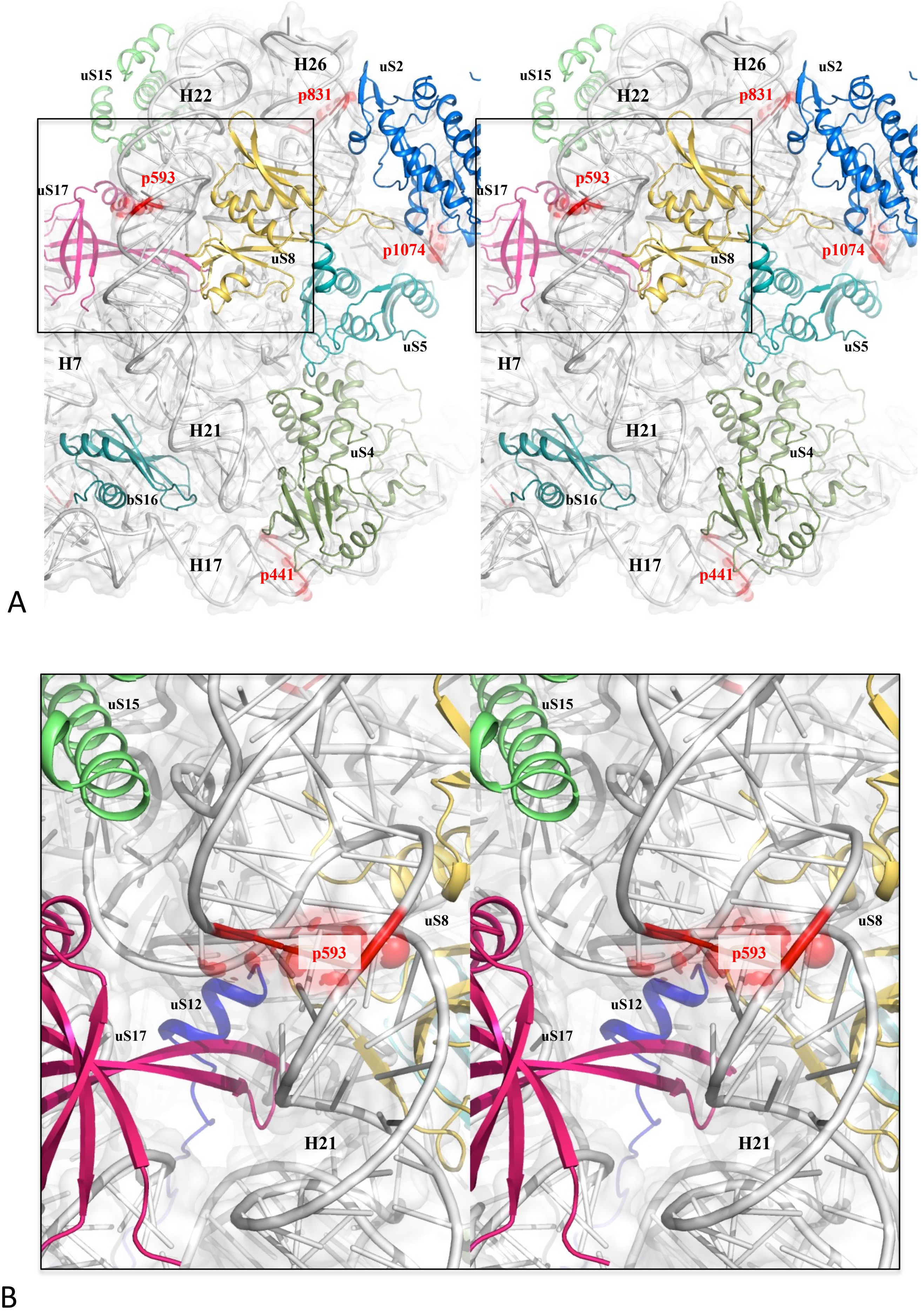
uS15
H26
uS2
H22
p831
p593
uS17
uS8
p1074
uS5
H7
H21
uS4
bS16
H17
p441
A
uS15
uS8
p593
uS12
uS17
H21
B

Figure S15

betweenness

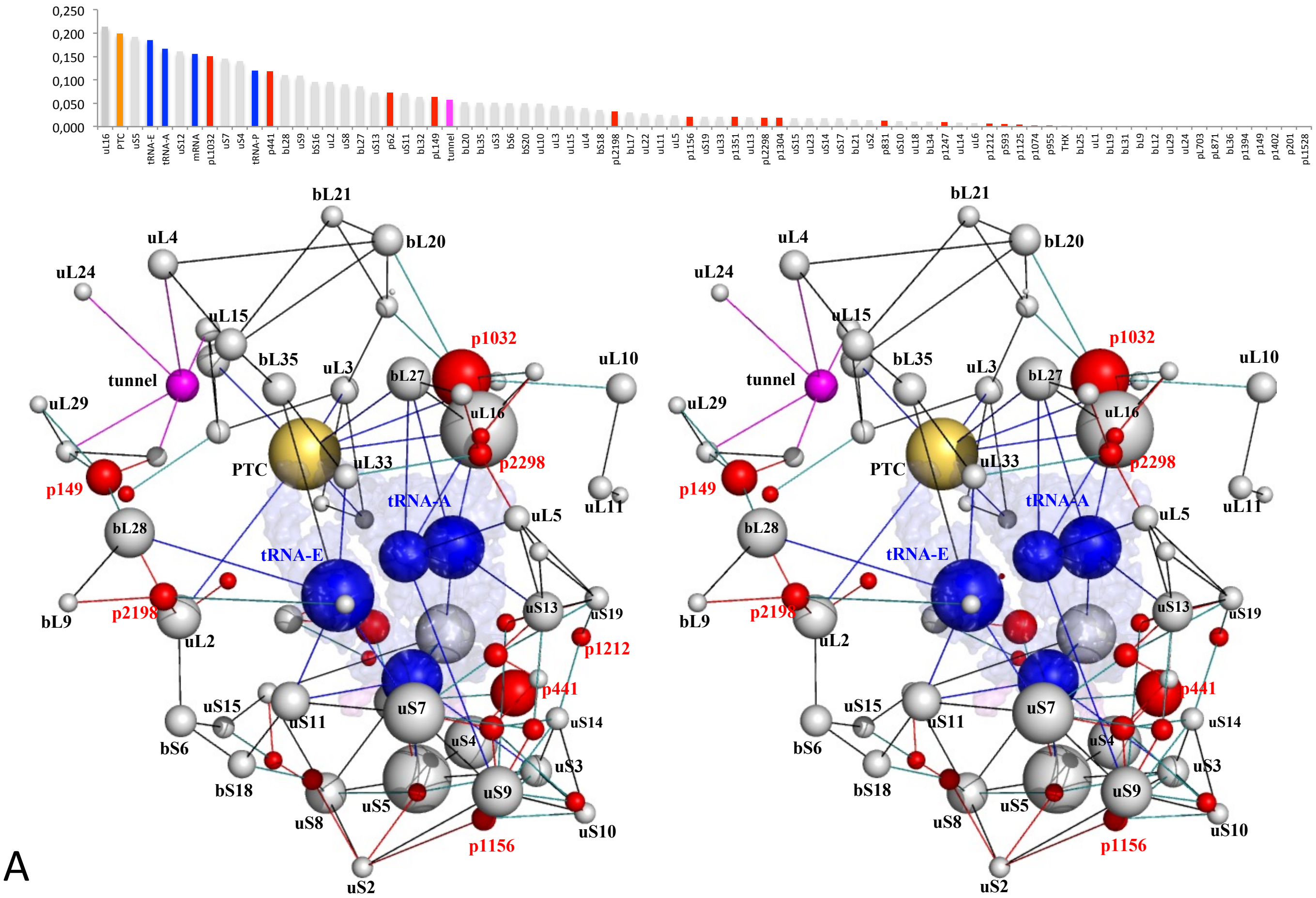

A

eigenvector

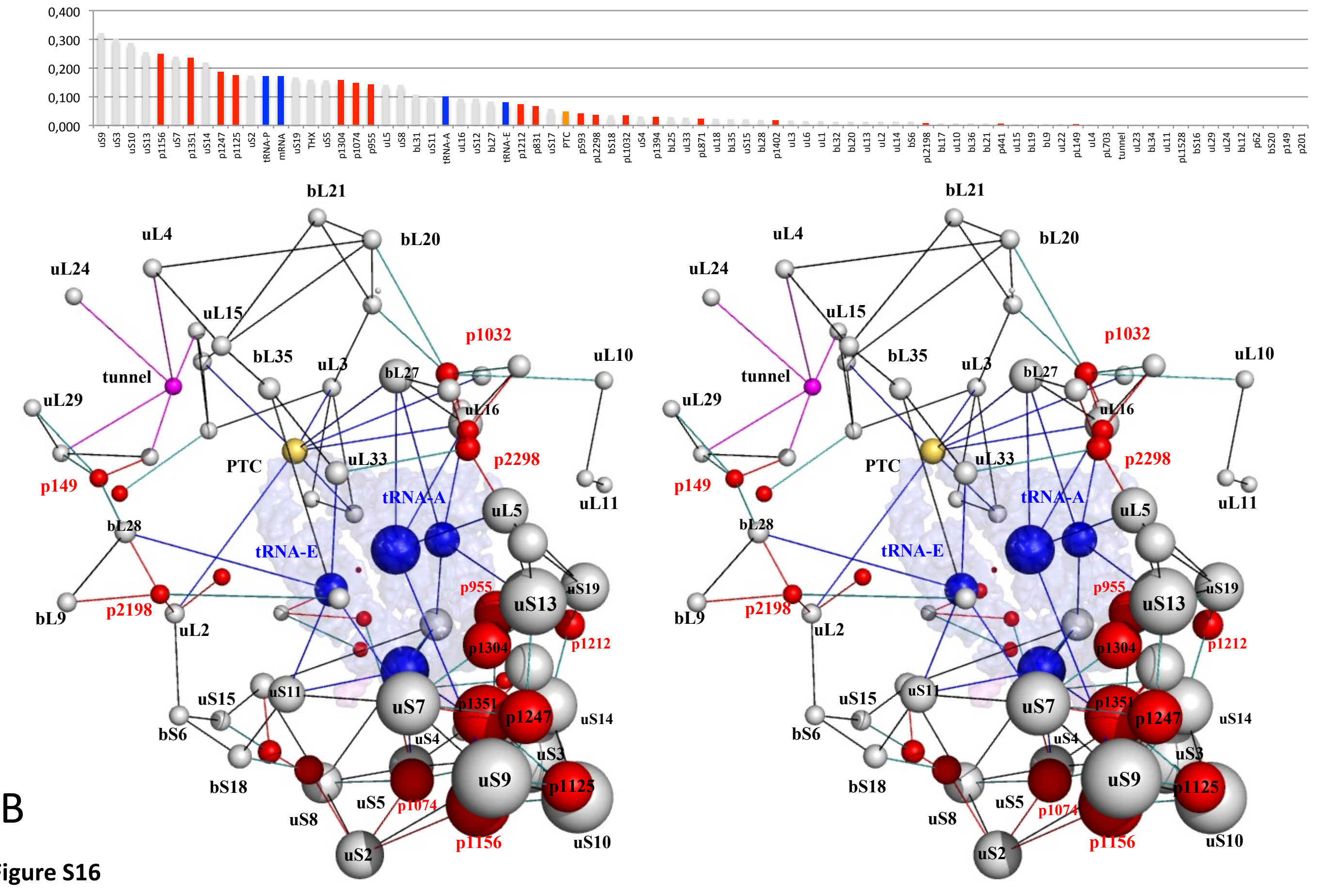

B

Figure S16

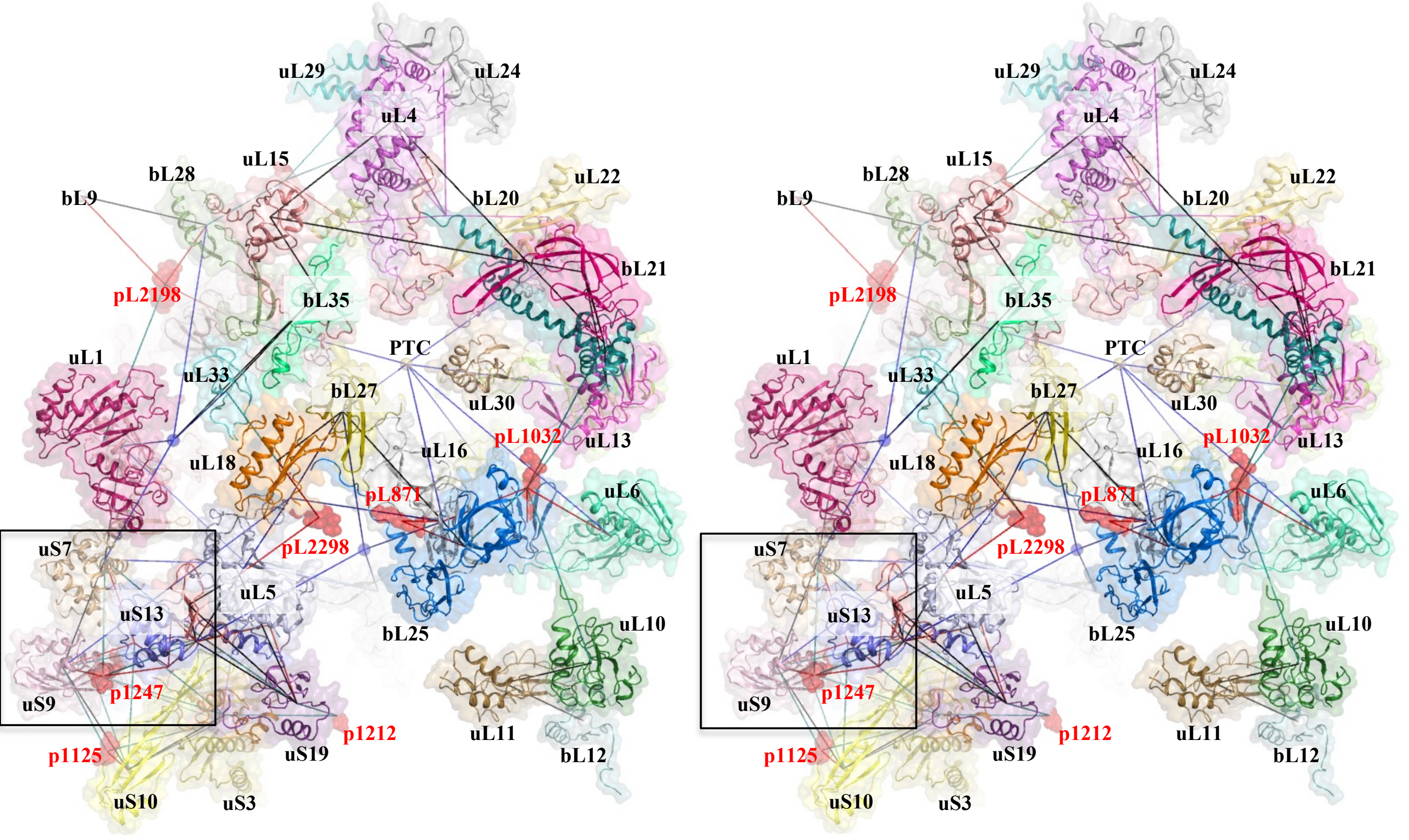

A

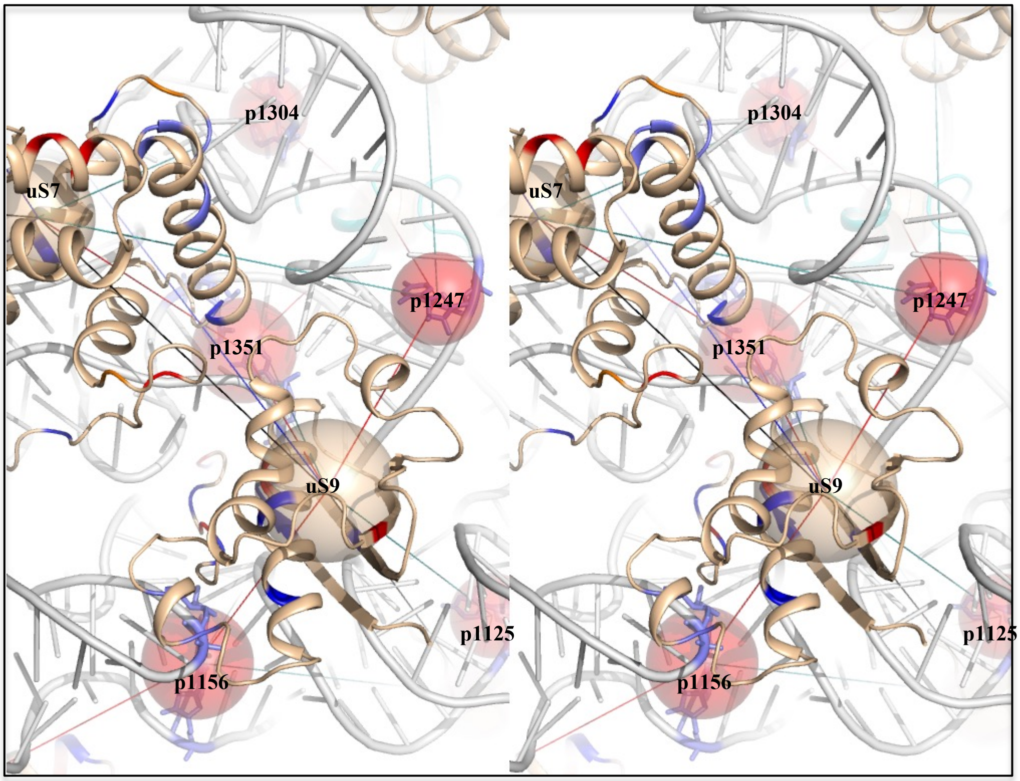

B

Figure S17

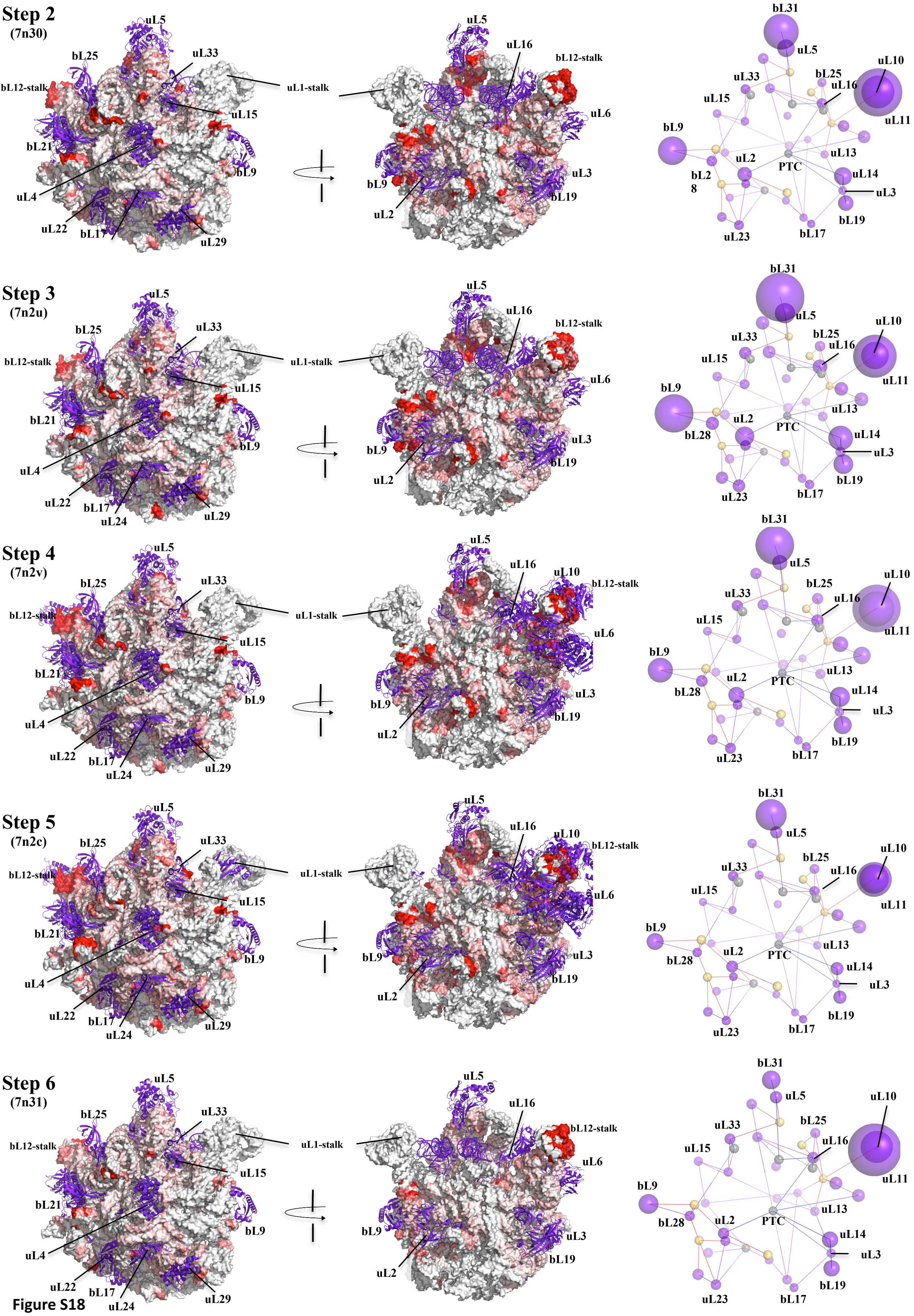

Figure S18

LSU stalks and Central Protuberance

pL2198 (uL2 fixed): LSU, uL1 stalk

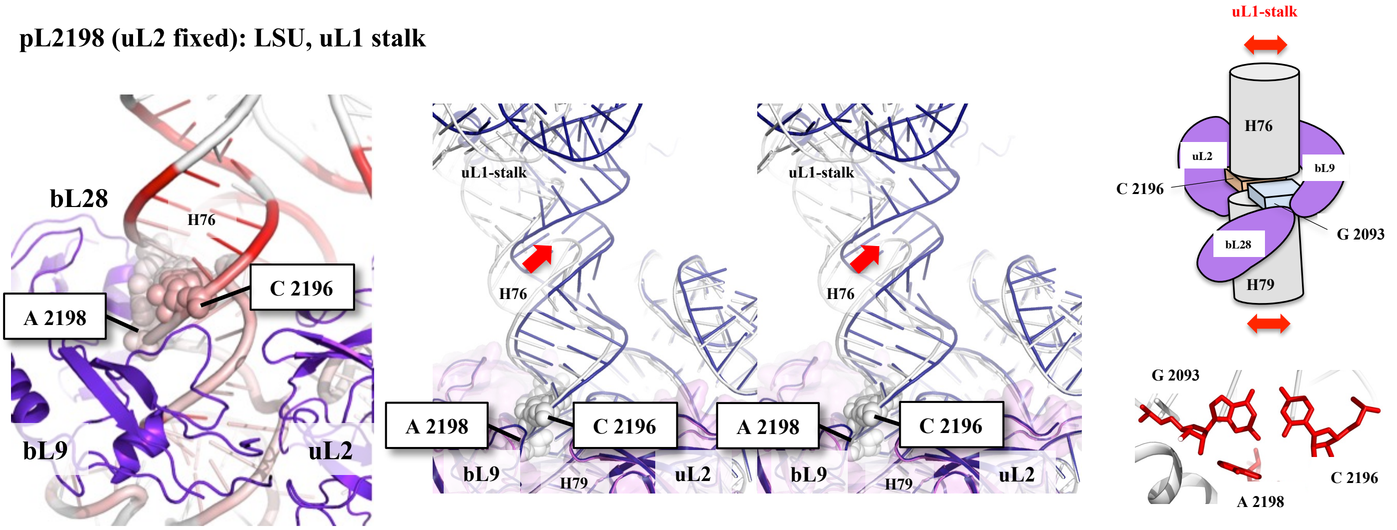

A

pL2298 (uL5 fixed) : Central Protuberance

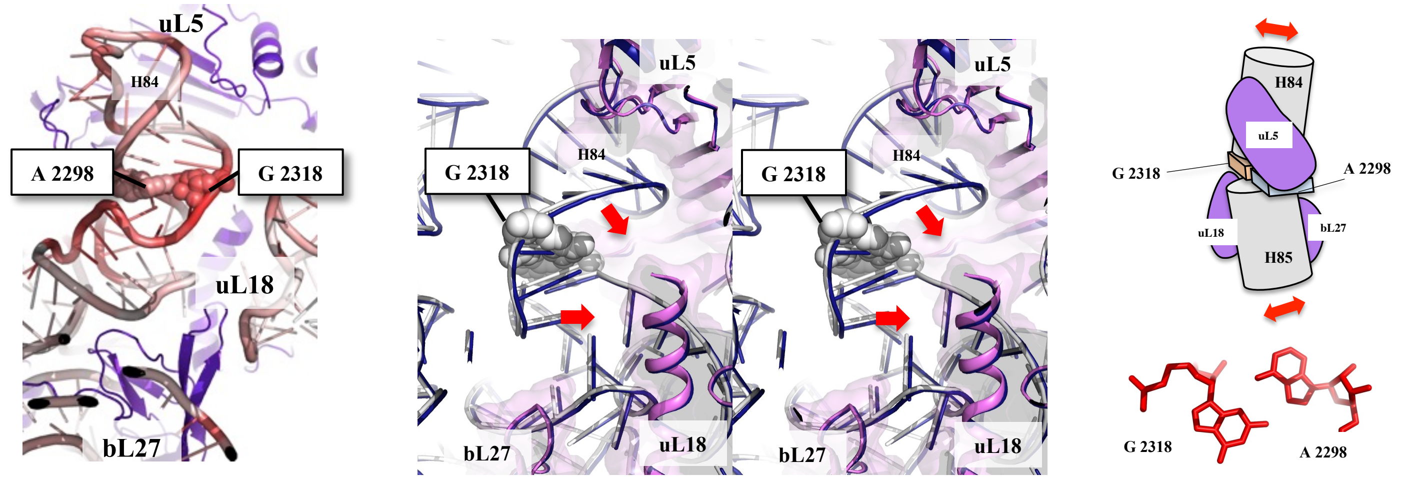

B

pL1032 (uL16 fixed) : bL12-stalk

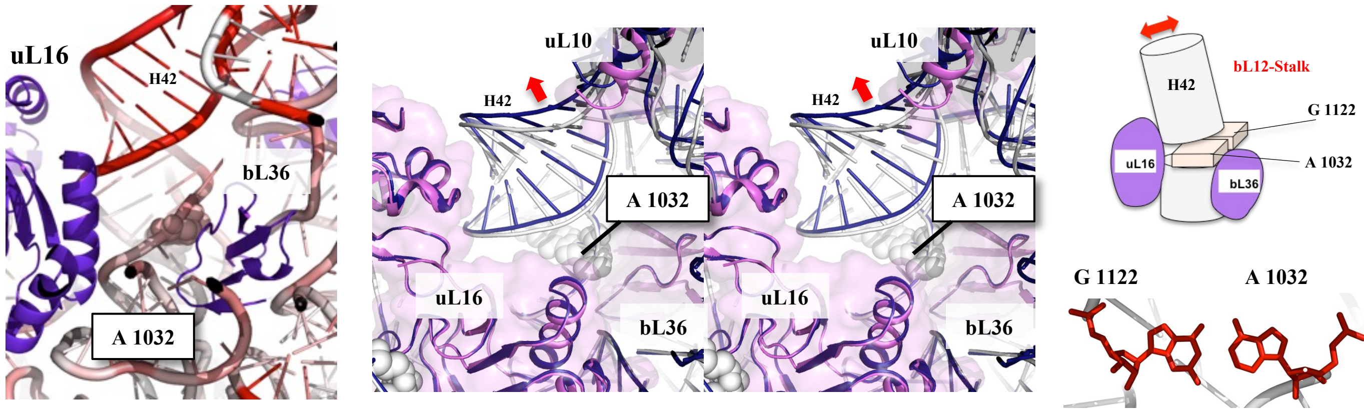

C

Figure S19

LSU helices H34 and H38

pL703 (uL2 fixed) : H34 interface with SSU and uS15

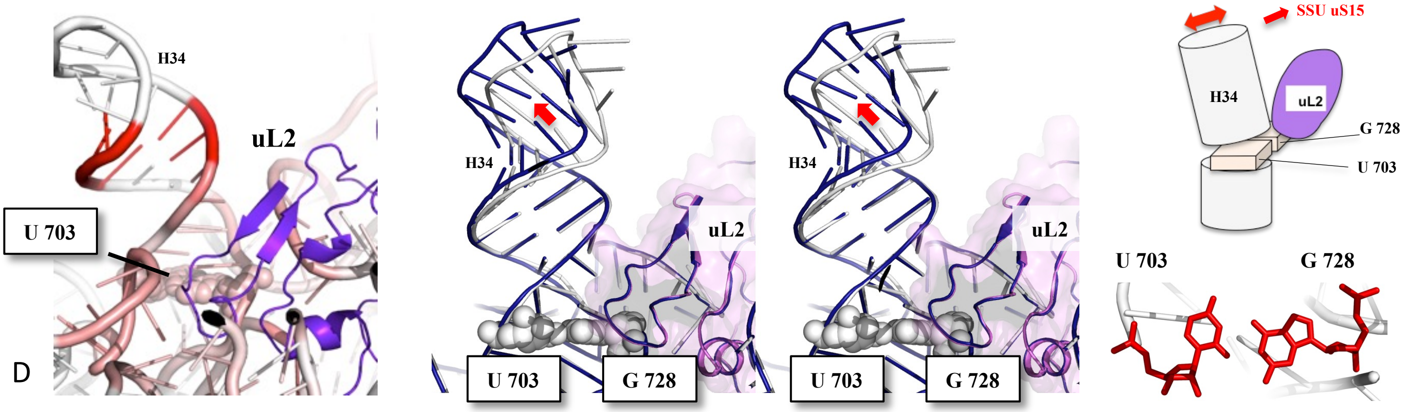

pL871 (uL16 fixed) : H38 transient interactions with tRNAs, uL5 and uS19

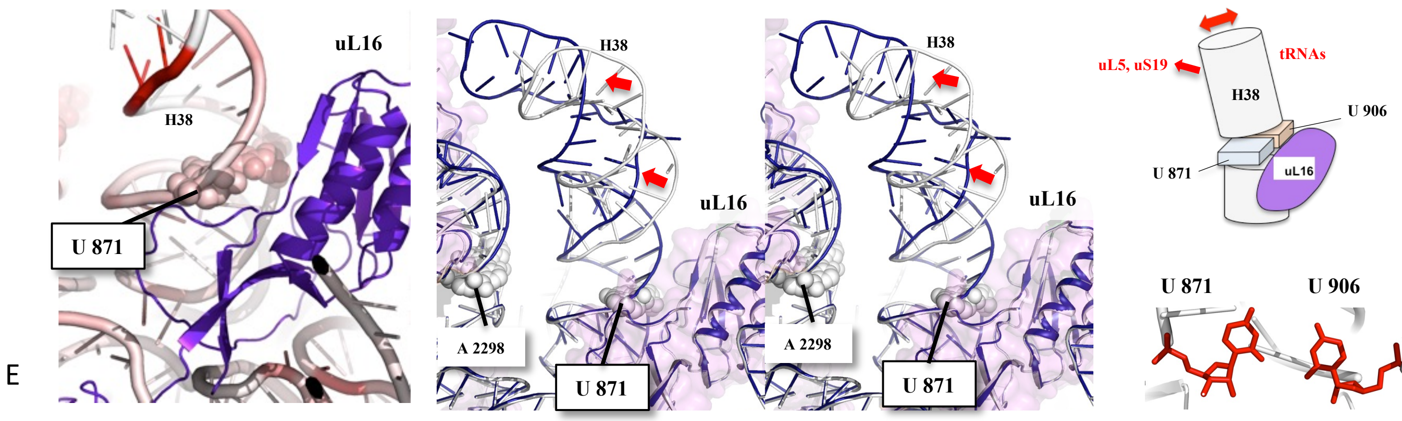

Figure S19 (continued)

SSU internal motions of the head during swivelling

p1074 (uS2 fixed): hinge body-head

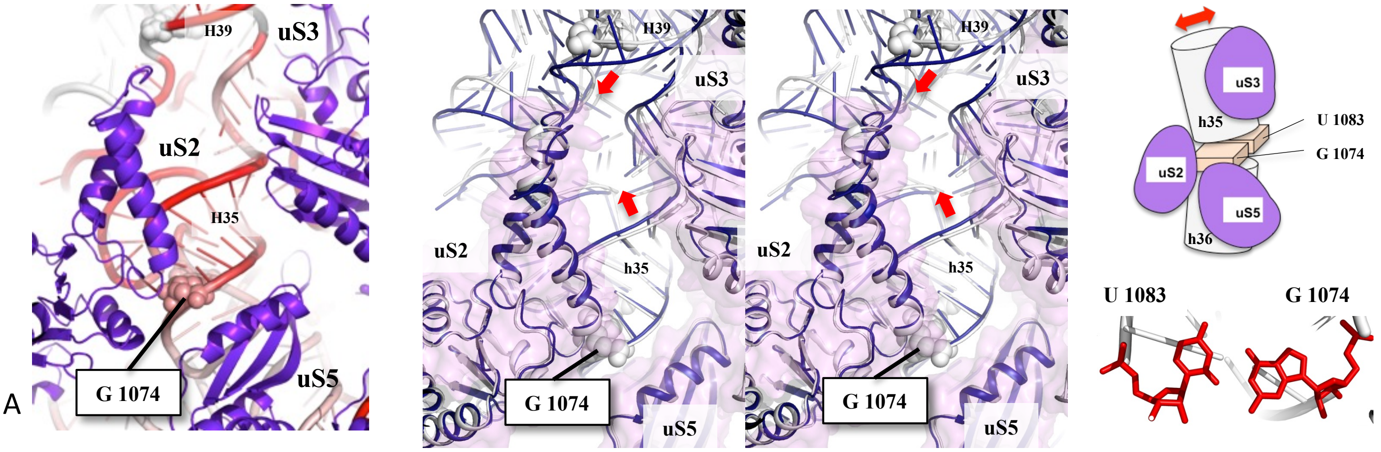

p1225 (uS10 fixed)

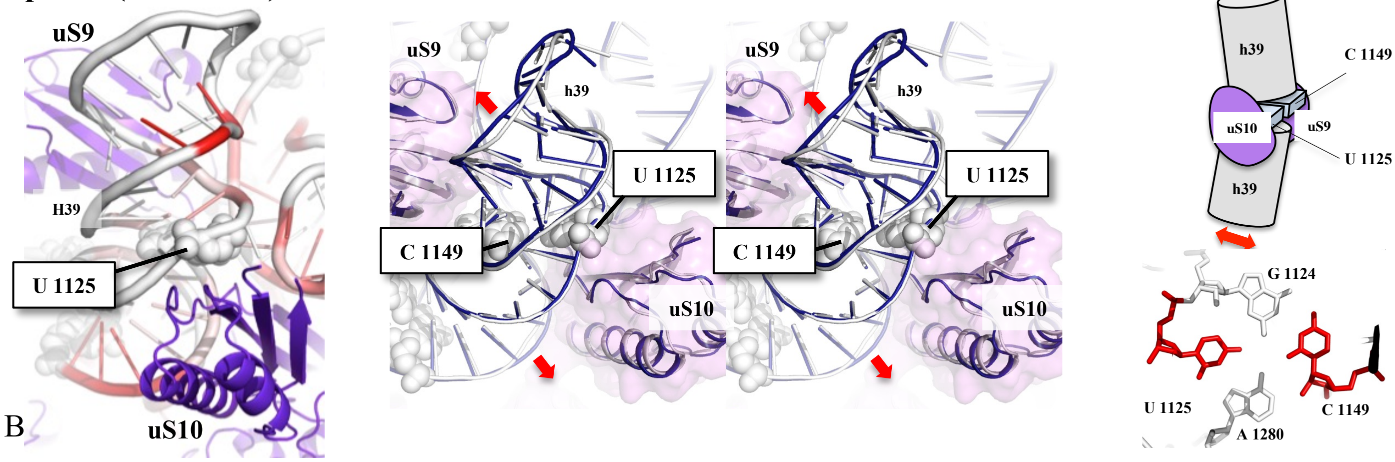

p1156 (uS9 fixed)

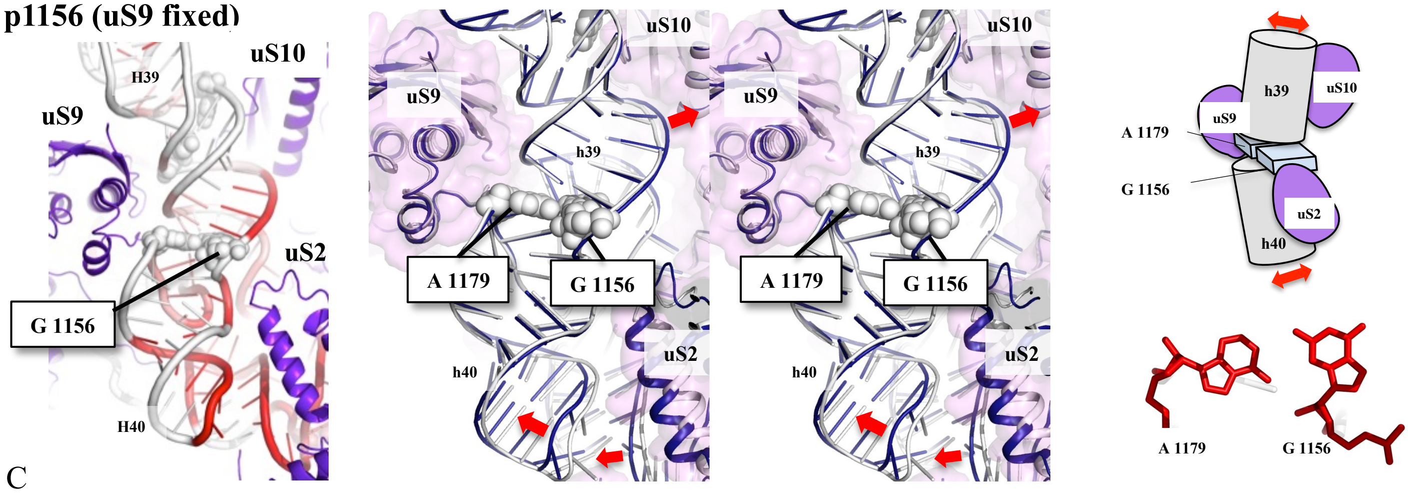

Figure S20

SSU: internal motions of the head during swivelling (continued)

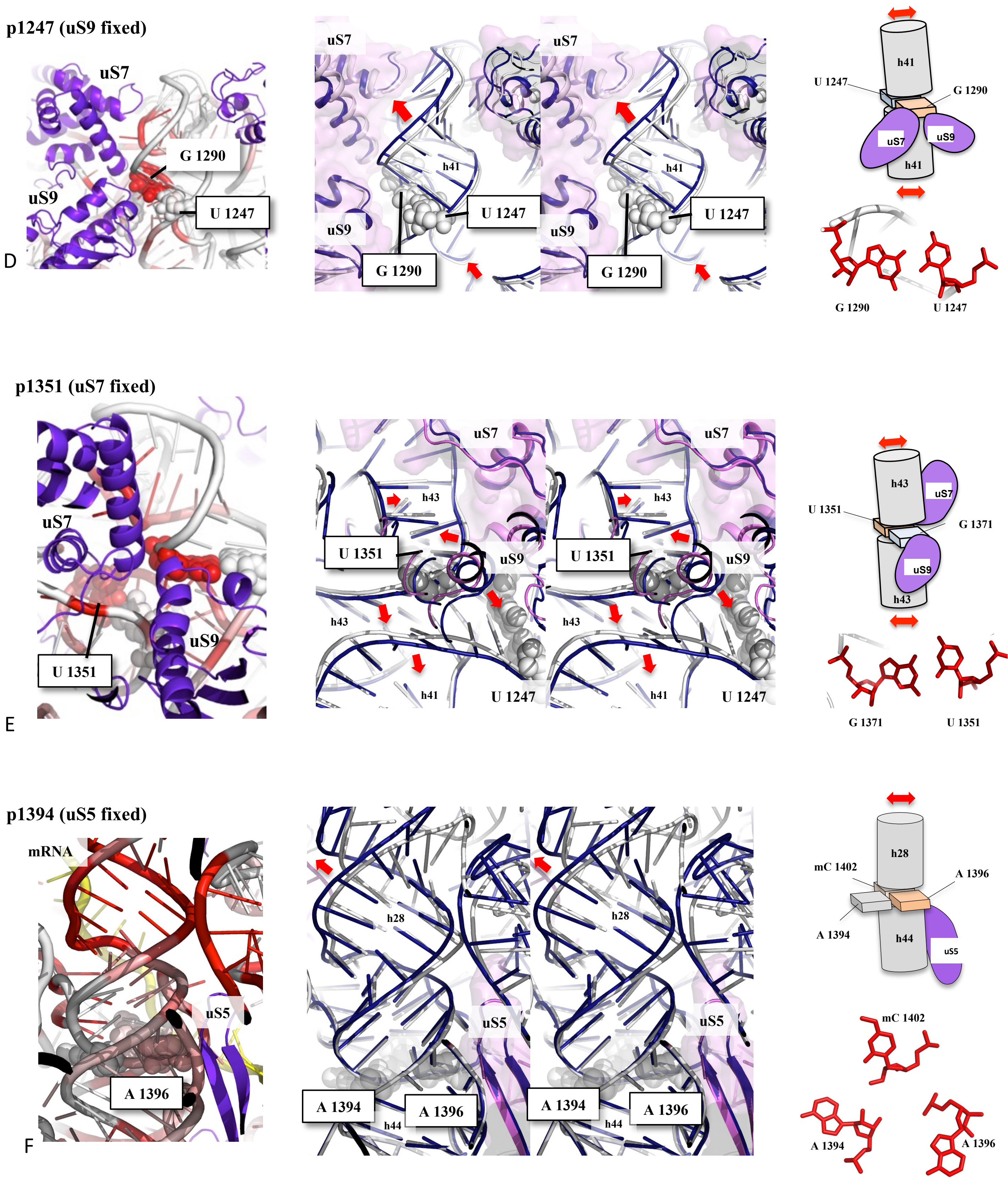

Figure S20 (continued)

SSU: internal motions of the body during swivelling (continued)

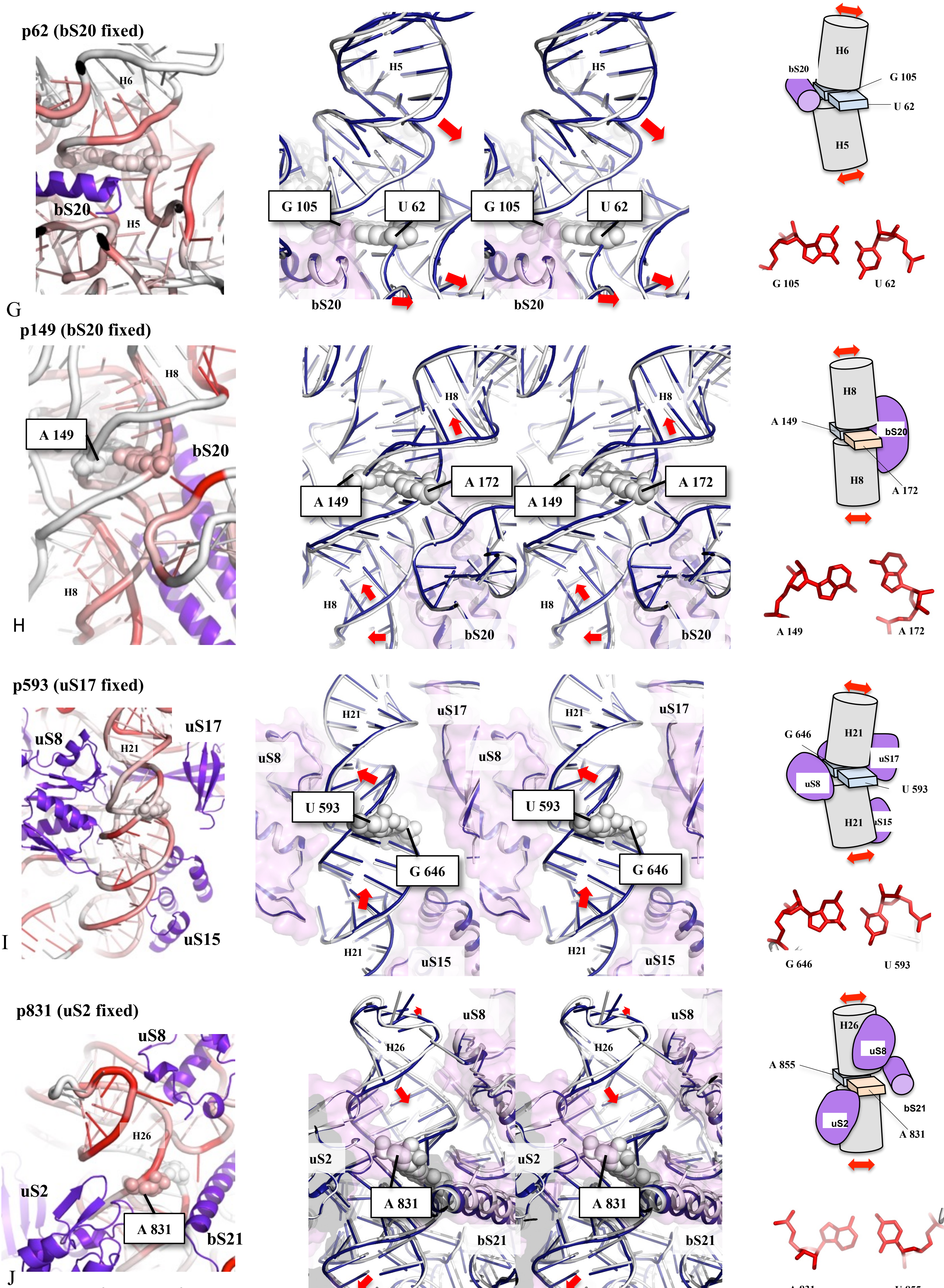

Figure S20 (continued)

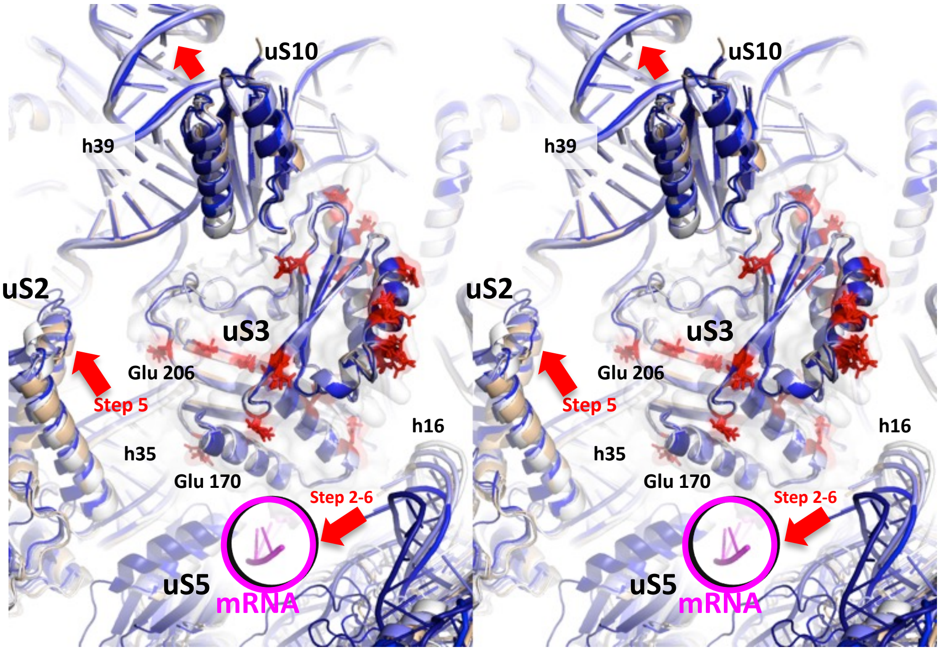

A

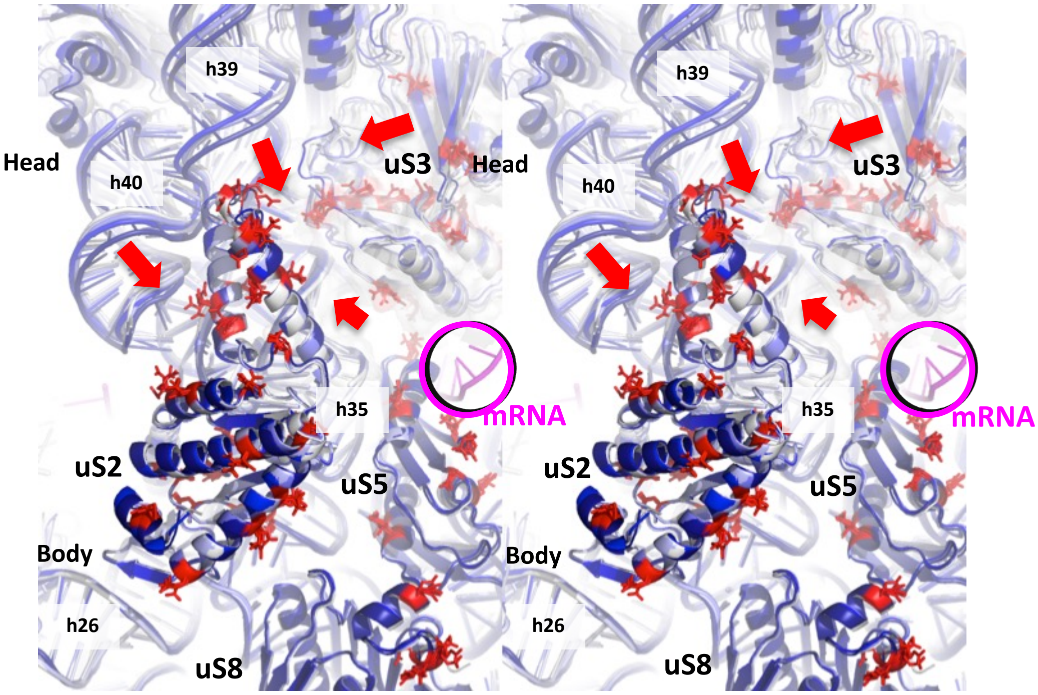

B

Figure S21

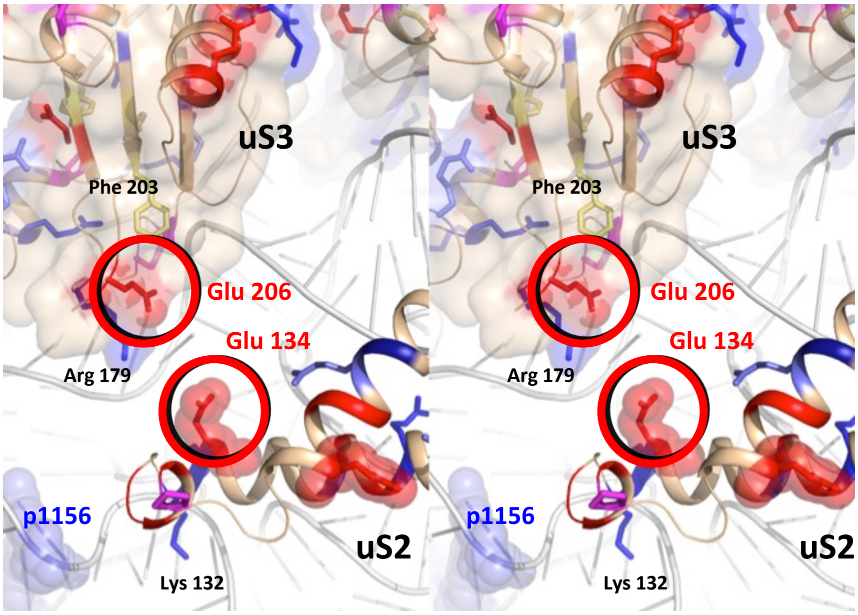

C

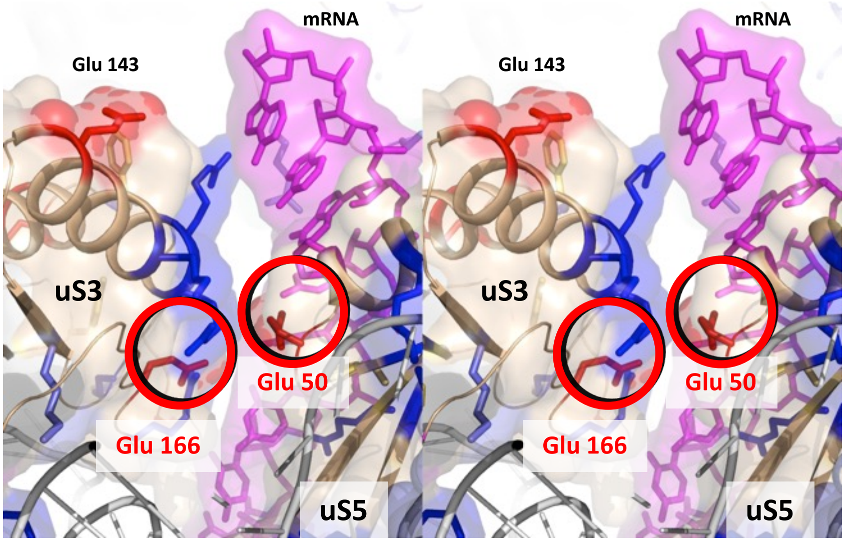

D

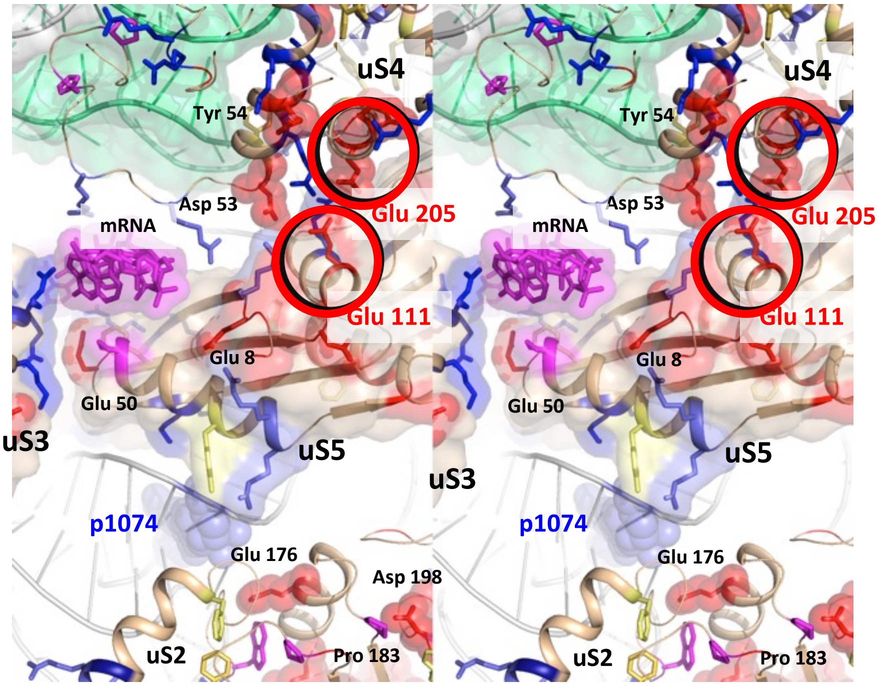

E

Figure S21 (continued)

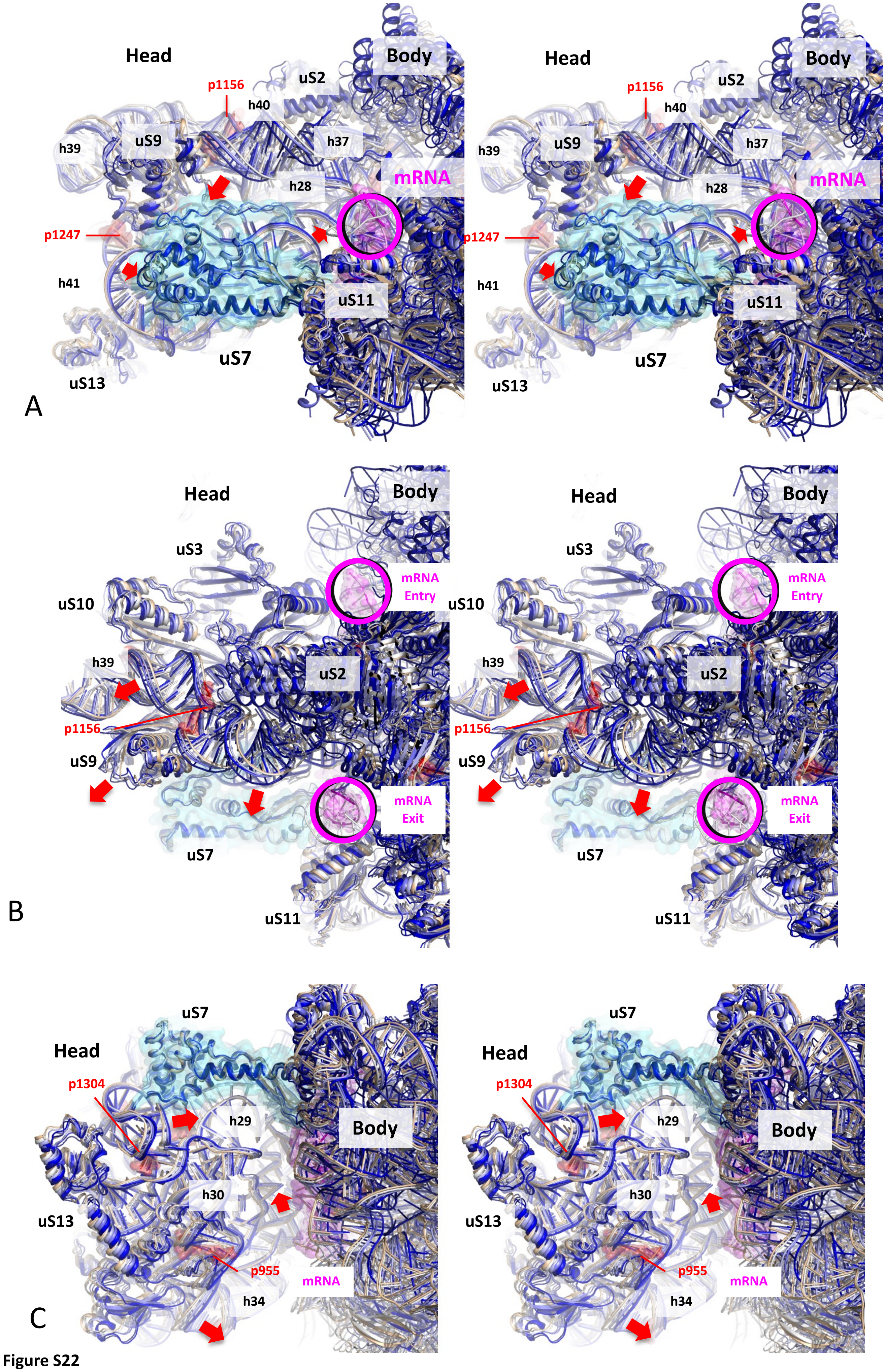
Head
Body
p1156
uS2
h40
uS9
h39
h37
h28
mRNA
p1247
h41
uS11
uS7
uS13
A
uS3
uS10
mRNA
Entry
h39
uS2
p1156
uS9
mRNA
Exit
uS7
uS11
B
uS7
Head
p1304
h29
Body
h30
uS13
p955
mRNA
h34
C

Figure S22

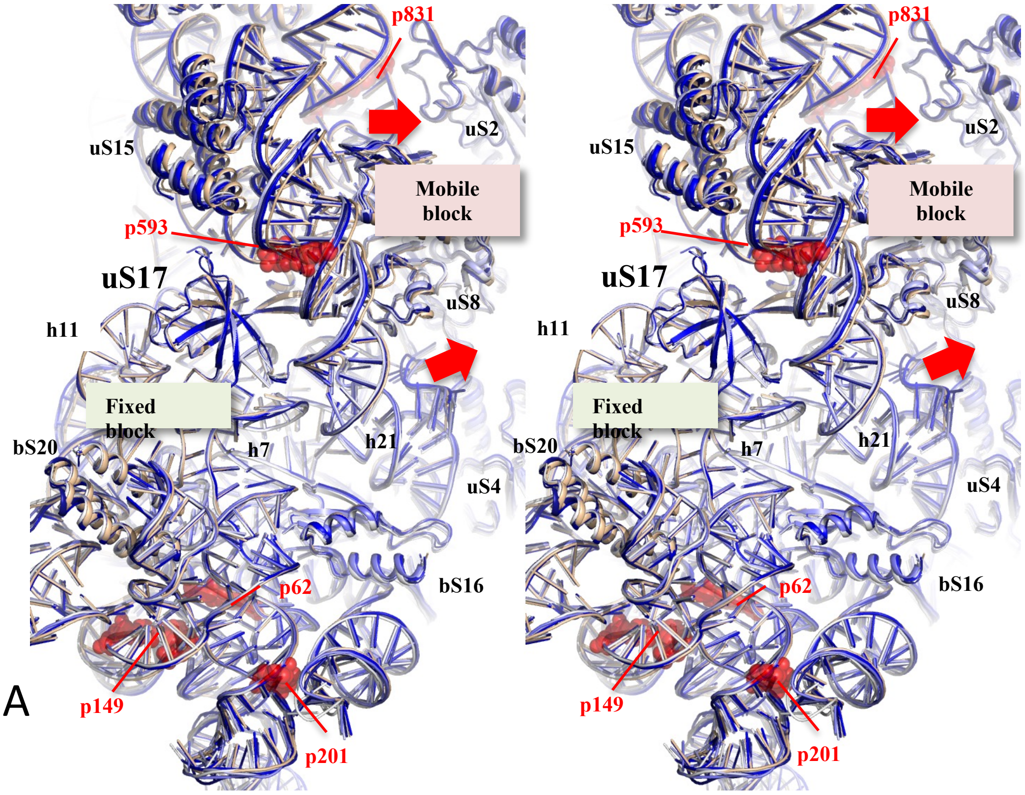

p831
uS15
uS2
Mobile block
p593
uS17
uS8
h11
Fixed block
bS20
h7
h21
uS4
bS16
p62
p149
p201
A

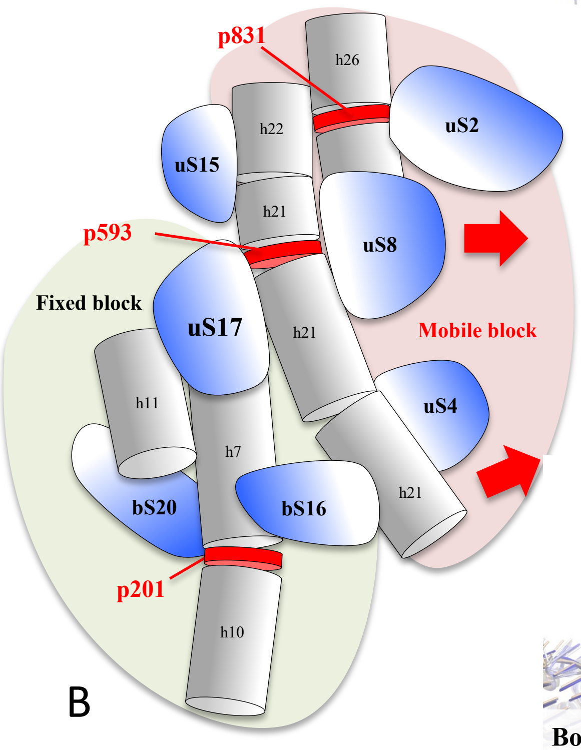

p831
h26
h22
uS2
uS15
h21
p593
uS8
Fixed block
uS17
h21
Mobile block
h11
uS4
h7
bS20
bS16
h21
p201
h10
B

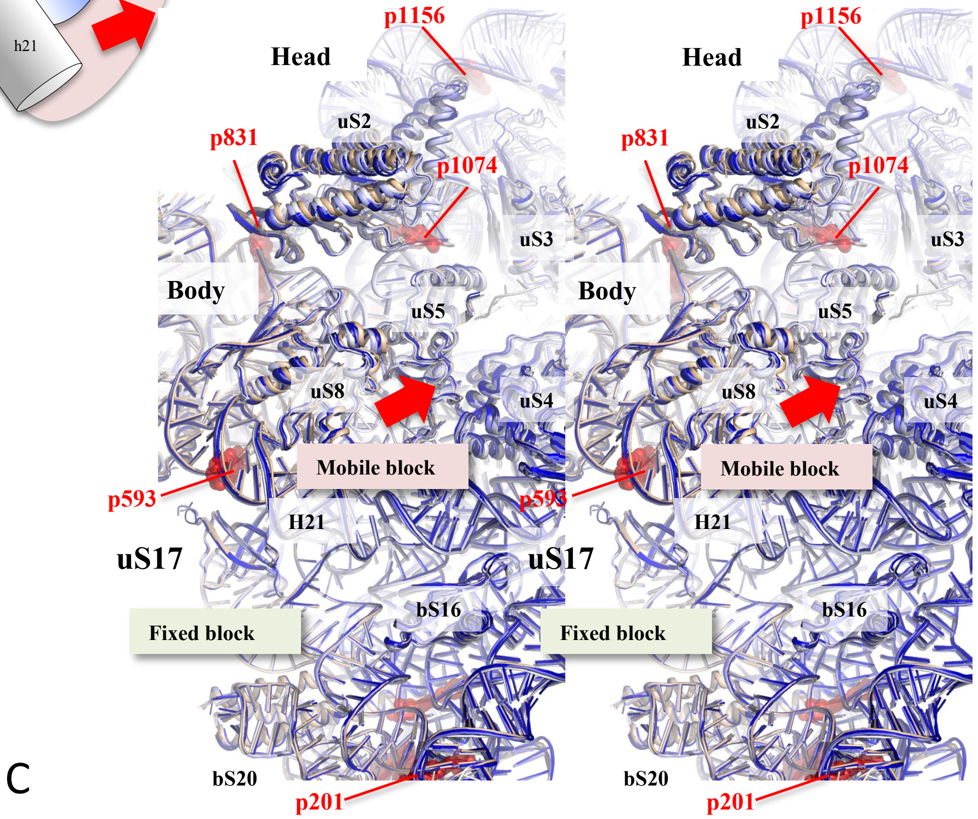

p1156
Head
uS2
p831
p1074
uS3
Body
uS5
uS8
uS4
Mobile block
p593
H21
uS17
bS16
Fixed block
bS20
p201
C

Figure S23

D

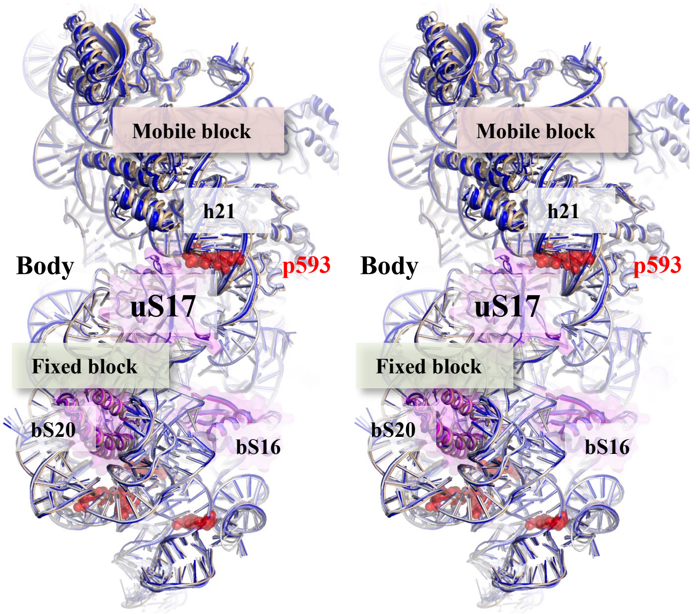

E

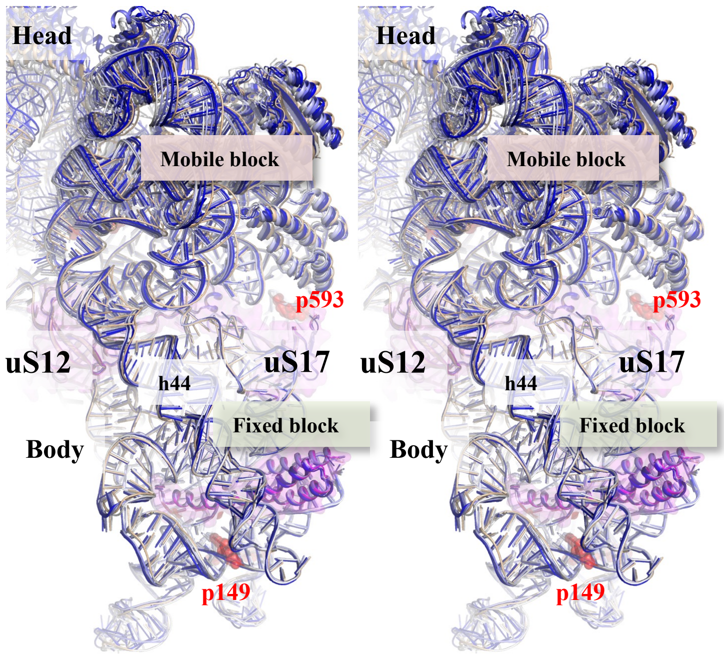

F

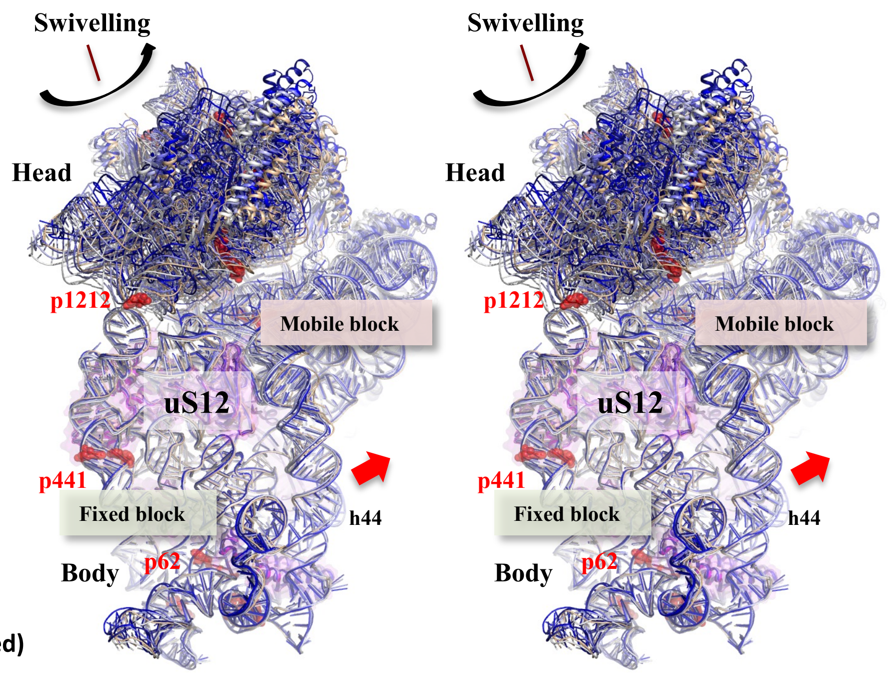

Figure S23 (continued)

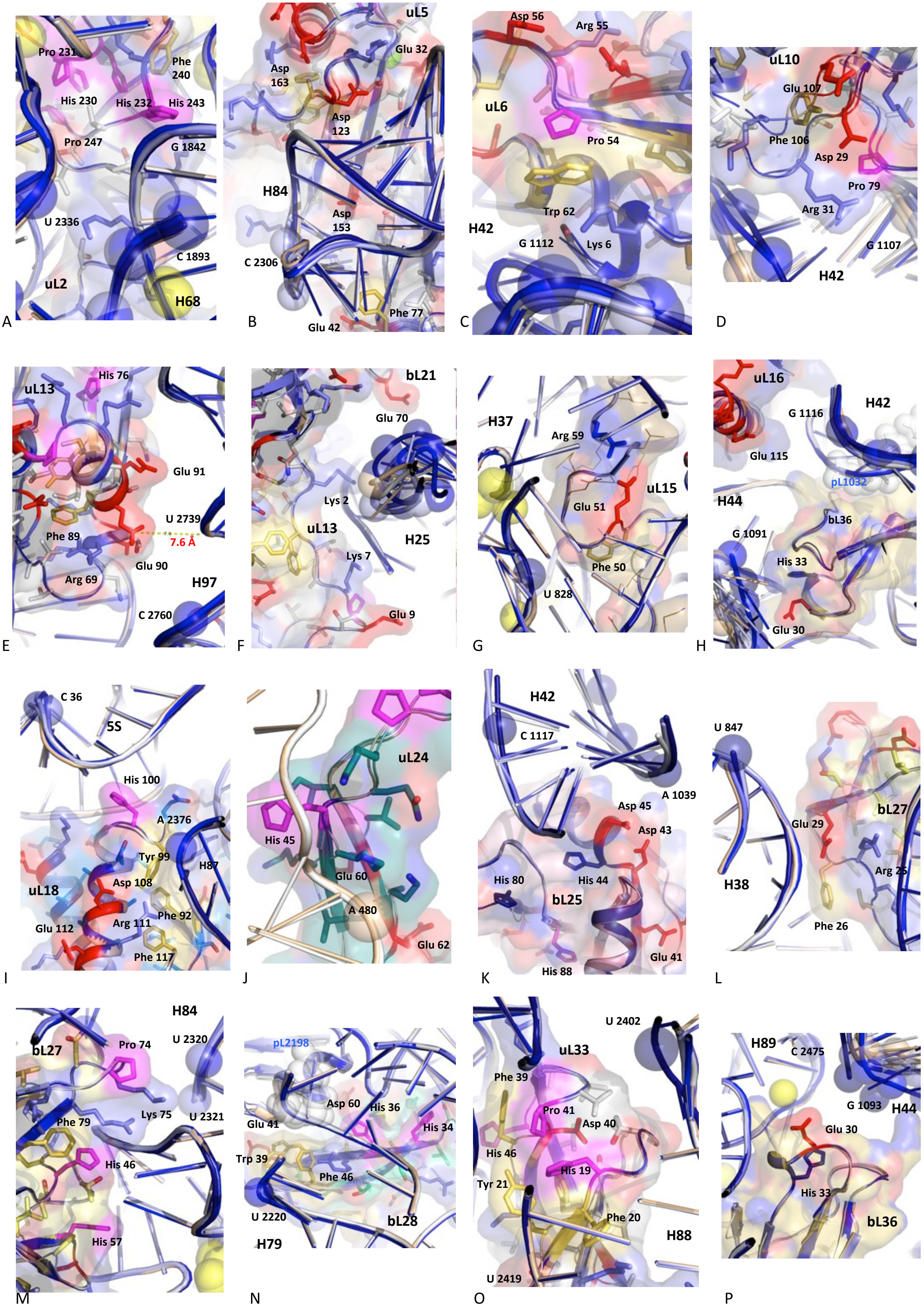

A
Pro 231
Phe 240
His 230
His 232
His 243
Pro 247
G 1842
U 2336
C 1893
uL2
H68
B
uL5
Glu 32
Asp 163
Asp 123
H84
Asp 153
C 2306
Phe 77
Glu 42
C
Asp 56
Arg 55
uL6
Pro 54
Trp 62
H42
G 1112
Lys 6
D
uL10
Glu 107
Phe 106
Asp 29
Pro 79
Arg 31
G 1107
H42
E
uL13
His 76
Glu 91
U 2739
7.6 Å
Phe 89
Glu 90
Arg 69
H97
C 2760
F
bL21
Glu 70
Lys 2
uL13
H25
Lys 7
Glu 9
G
H37
Arg 59
uL15
Glu 51
Phe 50
U 828
H
uL16
H42
G 1116
Glu 115
pL1032
H44
bL36
G 1091
His 33
Glu 30
I
C 36
5S
His 100
A 2376
Tyr 99
H87
uL18
Asp 108
Phe 92
Glu 112
Arg 111
Phe 117
J
uL24
His 45
Glu 60
A 480
Glu 62
K
H42
C 1117
A 1039
Asp 45
Asp 43
His 80
His 44
bL25
Glu 41
His 88
L
U 847
bL27
Glu 29
Arg 26
H38
Phe 26
M
H84
bL27
Pro 74
U 2320
Lys 75
U 2321
Phe 79
His 46
His 57
N
pL2198
Asp 60
His 36
Glu 41
His 34
Trp 39
Phe 46
U 2220
bL28
H79
O
U 2402
uL33
Phe 39
Pro 41
Asp 40
His 46
His 19
Tyr 21
Phe 20
H88
U 2419
P
H89
C 2475
G 1093
H44
Glu 30
His 33
bL36

Figure S24

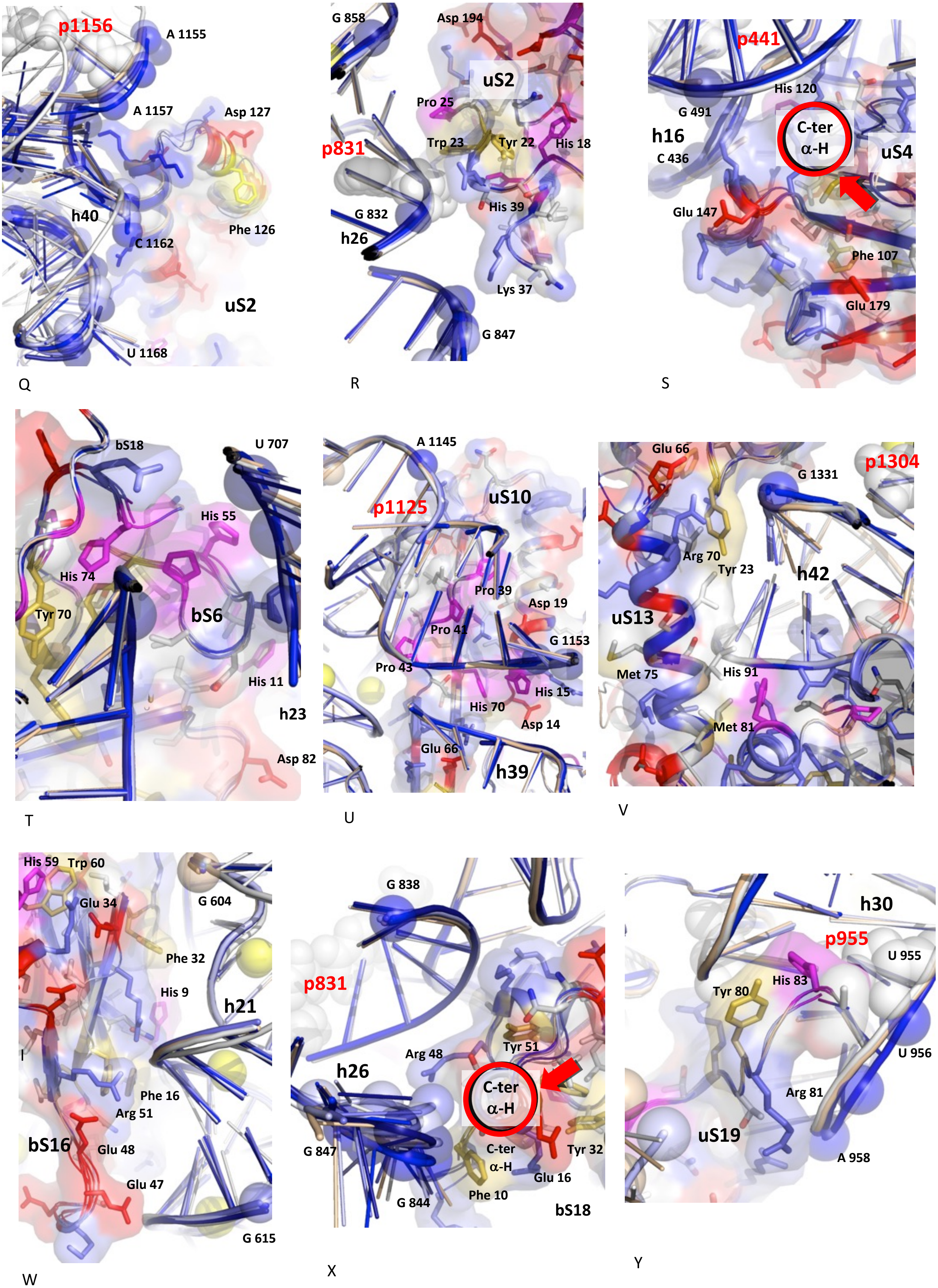

Figure S24 (continued)
